# Formation of exceptional monomeric YPhos–PdCl_2_ complexes with high activities in coupling reactions†

**DOI:** 10.1039/d2sc04523k

**Published:** 2022-10-26

**Authors:** Ilja Rodstein, Leif Kelling, Julian Löffler, Thorsten Scherpf, Abir Sarbajna, Diego M. Andrada, Viktoria H. Gessner

**Affiliations:** Faculty of Chemistry and Biochemistry, Chair of Inorganic Chemistry II, Ruhr University Bochum Universitätsstr. 150 44801 Bochum Germany viktoria.gessner@rub.de; General and Inorganic Chemistry Department, University of Saarland Campus C4.1 66123 Saarbruecken Germany

## Abstract

The use of well-defined palladium(ii) complexes as precatalysts for C–X cross-coupling reactions has improved the use of palladium catalysts in organic synthesis including large-scale processes. Whereas sophisticated Pd(ii) precursors have been developed in the past years to facilitate catalyst activation as well as the handling of systems with more advanced monophosphine ligands, we herein report that simple PdCl_2_ complexes function as efficient precatalysts for ylide-substituted phosphines (YPhos). These complexes are readily synthesized from PdCl_2_ sources and form unprecedented monomeric PdCl_2_ complexes without the need for any additional coligand. Instead, these structures are stabilized through a unique bonding motif, in which the YPhos ligands bind to the metal through the adjacent phosphine and ylidic carbon site. DFT calculations showed that these bonds are both dative interactions with the stronger interaction originating from the electron-rich phosphine donor. This bonding mode leads to a remarkable stability even towards air and moisture. Nonetheless, the complexes readily form monoligated LPd(0) complexes and thus the active palladium(0) species in coupling reactions. Accordingly, the YPhos–PdCl_2_ complexes serve as highly efficient precatalysts for a series of C–C and C–X coupling reactions. Despite their simplicity they can compete with the efficiency of more complex and less stable precatalysts.

## Abstract 2

Ylide-substituted phosphines form monomer, air-stable PdCl_2_ complexes which feature a unique bonding situation between the metal and the phosphine and ylide donor and exhibit high efficiencies as precatalysts in C–C and C–X cross-coupling reactions.

## Graphical abstract

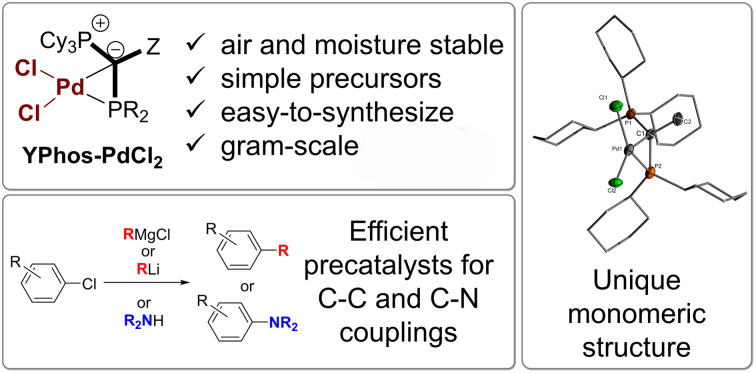

## Introduction

Palladium-catalyzed coupling reactions have become an indispensable tool in organic synthesis for the construction of C–C and C–X bonds under mild reaction conditions.^1^ Over the past two decades, the design of efficient catalysts and the establishment of reliable reaction protocols have turned them into robust methods also for large-scale industrial processes.^2^ Many advances in the field were based on the development of new ligands that allowed the tailoring of the catalyst properties to meet the requirements of different substrates and reaction conditions.^3^ Thus, intense research efforts have focused on the design of new ligands, above all phosphines and carbenes, to improve the reaction protocols with respect to substrate scope, temperature or catalyst loading.^4^ However, besides ligand design also the choice of palladium source has been recognized to have a profound impact on the efficiency of a catalyst system. Therefore, researchers have aimed at developing preformed palladium complexes as well-defined precatalysts.^5^

**Fig. 1:**
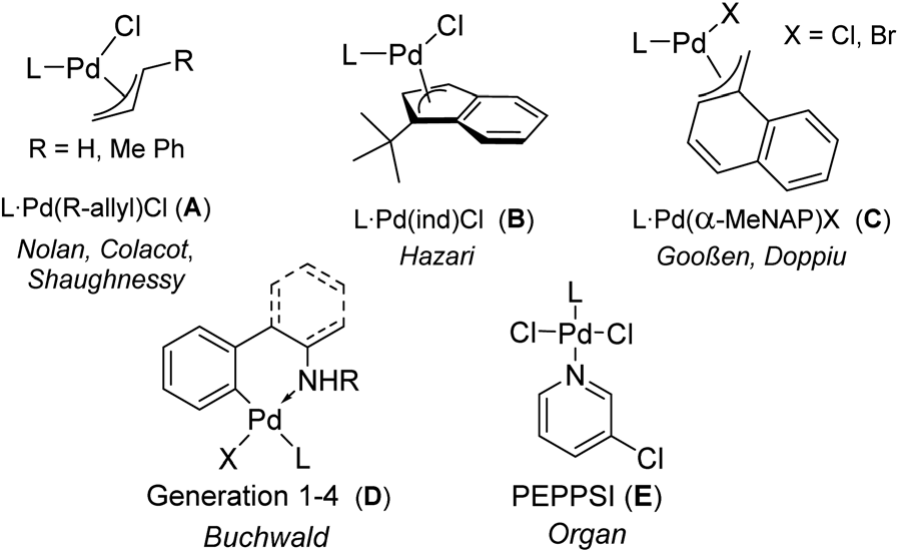
Structures of state-of-the-art bench-stable palladium (ii) precatalysts (L = phosphine or *N*-heterocyclic carbene).

In general, the optimal precatalyst should be air-stable, easy to handle and readily accessible from inexpensive and commercially available starting materials. Furthermore, catalyst activation should be facile and not lead to the formation of toxic or difficult to separate by-products which limit applications such as in the synthesis of pharmaceuticals or organic materials, where highly pure products are mandatory. Since monoligated Pd(0) complexes are usually the active species in coupling reactions,^6^ complexes with palladium in the zero oxidation state and a 1 : 1 metal : ligand ratio are the obvious choices. However, Pd(0) precursors such as Pd_2_dba_3_ often show varying qualities and limited stability particularly at ambient conditions,^7^ so that their phosphine complexes are usually only prepared *in situ*. In contrast, L_2_Pd complexes are usually more stable, but require higher reaction temperatures for the dissociation of the second ligand.^8^ Only a few other Pd(0) precatalysts have been developed to date.^9^ Palladium(i) complexes also readily form the active species, but suffer either from limited stability or are applicable to a limited number of phosphines or carbenes.^10^ Therefore, research endeavors have focused on Pd(ii) complexes which are usually air-stable and thus easy to handle. Here, the design has focused on optimizing the generation of the LPd(0) species, leading to the development of a series of anionic coligands that facilitate the reduction process and minimize catalyst deactivation through formation of inactive metal species. Successfully applied complexes include η^3^-allyl systems such as allyl, crotyl or cinnamyl (A),^11,12^ indenyl (B)^13^ or methylnaphthyl (C)^14^ complexes as well as palladacycles^15^ above all based on the 2-aminobiphenyl structure (D, Fig. 1).^16^ Also oxidative addition complexes of type [LPd(Ar)Cl]_*x*_ (*x* = 1 or 2) have been used as “on-cycle” precatalysts, as was first described by Shaughnessy in 2017.^17^ However, these complexes showed widely varying success as precatalysts and always lead to the formation of another coupling by-product in the amount of pre-catalyst used.^18^

Surprisingly, the simplest Pd(ii) precursor, PdCl_2_, has received less attention in the synthesis of defined precatalysts, although it usually provides stable complexes that do not liberate organic by-products upon activation. Although Kumada reported on the use of PdCl_2_(ddpf) in 1979,^19^ the later developed, more sophisticated monophosphines were rarely applied as their PdCl_2_ complexes.^20^ This may be due to the observation that the halide bridged [LPdX_2_]_2_ dimers often exhibited lower activities^21^ than mononuclear systems of type LPd(X_2_)L′ with an additional “throw-away” ligand as shown for example in case of the PEPPSI catalysts E.^22^

Recently, we reported on the synthesis and application of ylide-substituted phosphines (YPhos).^23^ These ligands feature enhanced donor properties due to the electron-releasing power of the ylide group which led to high activities in a variety of coupling reactions.^24^ In case of Buchwald–Hartwig aminations and Murahashi coupling reactions, an improved performance was observed with indenyl precatalysts of type B compared to *in situ* prepared catalysts from the phosphine and Pd_2_dba_3_, allowing for unprecedented activities.^25^ This improved performance led us to search for other simpler precatalysts, which are accessible from inexpensive precursors, more stable than previous precatalysts and do not produce an additional byproduct. Here, we report that simple PdCl_2_ complexes perfectly fulfil this purpose. The exceptional activity of these simple precatalysts is based on the unusual monomeric structure realized by the YPhos ligands through a unique *k*C,*k*P coordination mode.

## Results and discussion

### YPhos–PdCl_2_ synthesis, structures and properties

**Scheme 1:**
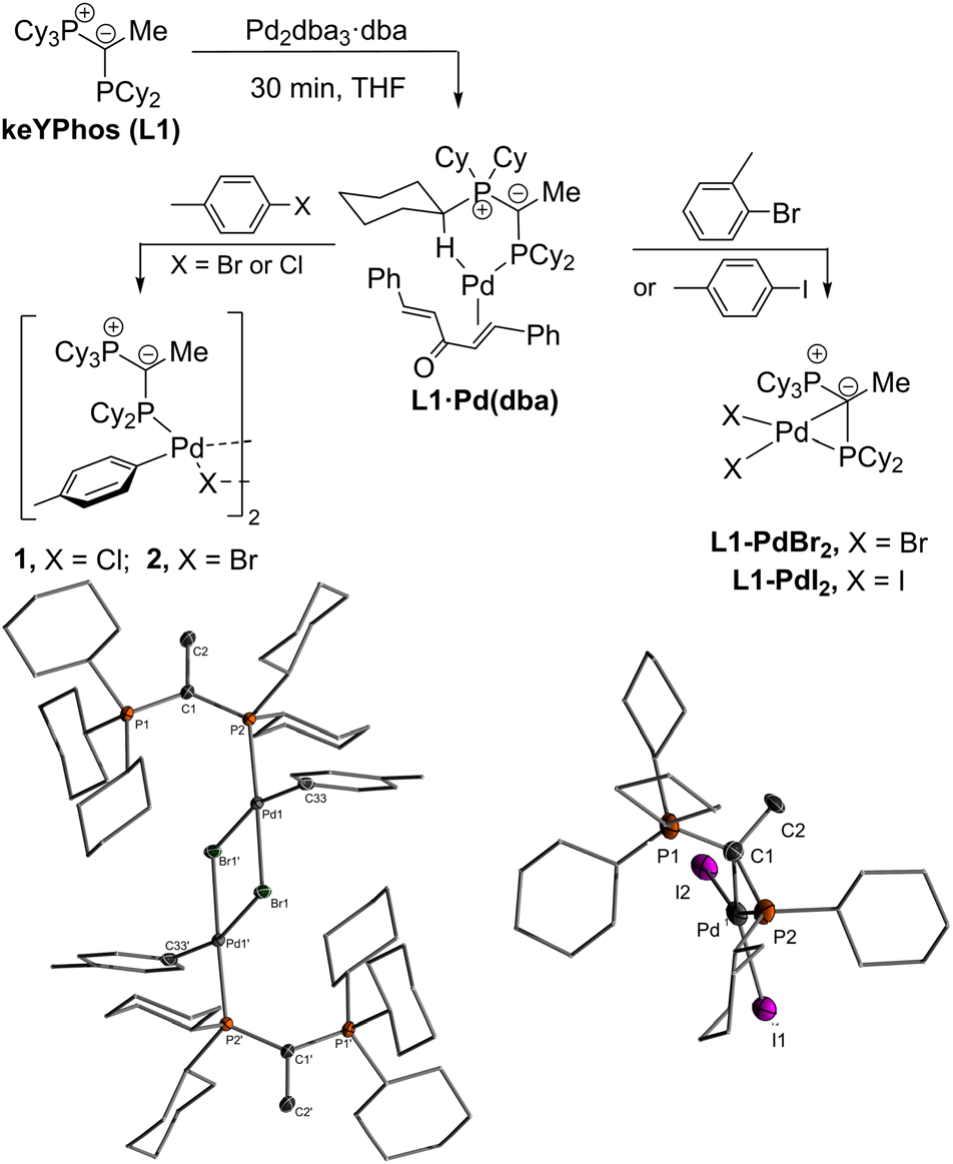
Reactivity of L1·Pd(dba) with different aryl halides.

In search of efficient precatalysts for our YPhos ligands, we initially focused on using oxidative addition complexes which had repeatedly been shown to be suitable entry points into catalytic cycles.^26^ In case of *p*-tolyl chloride and bromide, the dimeric halide-bridged addition complexes were found to be easily accessible such as with the YPhos ligand, keYPhos (L1) and Pd_2_dba_3_ in the presence of an excess of aryl halide (Scheme 1).^27^ Both complexes 1 and 2 feature two characteristic doublets in the ^31^P{^1^H} NMR spectrum with large coupling constants of approx. 50 Hz. In contrast, switching to the sterically more demanding *ortho*-tolyl bromide, which we expected to form a monomeric oxidative addition complex with enhanced reactivity, led to the formation of palladium black along with a further new species featuring two signals with diminishing coupling constants between the two phosphorus centers in the keYPhos ligand. GC analysis of the reaction mixture showed the formation of the homo-coupled biaryl compound. The same observation was made using aryl iodides independent of their steric demand. XRD analysis finally provided unequivocal clarity about the composition of the newly formed compounds as simple Pd(ii) dihalide complexes, L1·PdBr_2_ and L1·PdI_2_. To our surprise, these complexes formed monomeric structures even without an additional coligand such as pyridine (*cf.* the PEPPSI type catalysts). Instead, the palladium center is coordinated by the phosphine as well as the ylidic carbon center (Scheme 1). Despite many YPhos transition metal complexes have been isolated in the past years, such a coordination mode has never been observed before for any metal.^24,28^

**Scheme 2:**
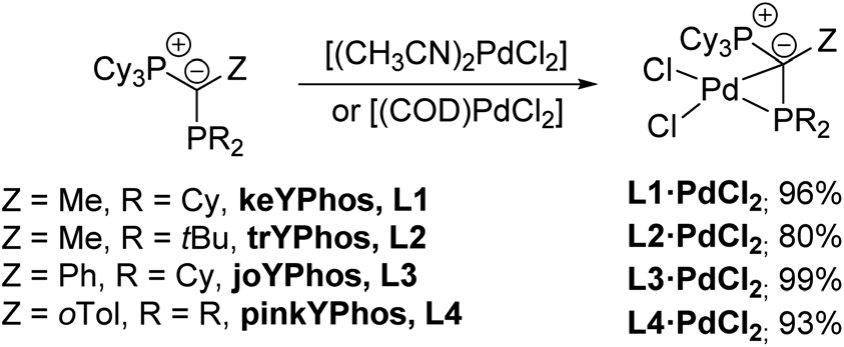
Synthesis of L·PdCl_2_ complexes with different YPhos ligands.

The unique structure of the YPhos Pd(ii) dihalide complexes led us to develop a simpler preparation method starting directly from PdCl_2_. Simple mixing of the ligand with PdCl_2_ revealed to be problematic due to solubility issues. However, the more soluble precursors [(CH_3_CN)_2_PdCl_2_] or [(COD)PdCl_2_] gave L1·PdCl_2_ as a yellow solid by simple filtration from the reaction solution in yields of up to 96% (Scheme 2). Alternatively, L1·PdCl_2_ can also be prepared by *in situ* formation of [(CH_3_CN)_2_PdCl_2_] using a suspension of PdCl_2_ in a 1 : 1 mixture of THF and acetonitrile together with the ligand. The mixture of both solvents was necessary to account for the different solubilities of the ligand and the metal salt. Nonetheless, employment of the preformed acetonitrile complex gave slightly higher yields so that this method was employed to access the PdCl_2_ complexes with the other YPhos ligands joYPhos and pinkYPhos. For the bulkier trYPhos ligand, successful synthesis could only be achieved with [(COD)PdCl_2_]. Overall, all PdCl_2_ complexes could be isolated in excellent yields of greater than 80%.

To our delight, all L·PdCl_2_ complexes feature remarkably high stabilities even towards air and moisture and thus can be handled without any precautions. Storage of L·PdCl_2_ at ambient conditions led to no signs of decomposition even after several months. In solution however, only L3·PdCl_2_ and especially L4·PdCl_2_ with an aromatic backbone were stable toward oxygen and water for a longer time presumably due to the enhanced steric protection of the ylidic carbon center by the aryl substituents. This high stability of L·PdCl_2_ is beneficial for their handling and superior to previously reported YPhos allyl-type palladium precatalysts.^25^

**Fig. 2:**
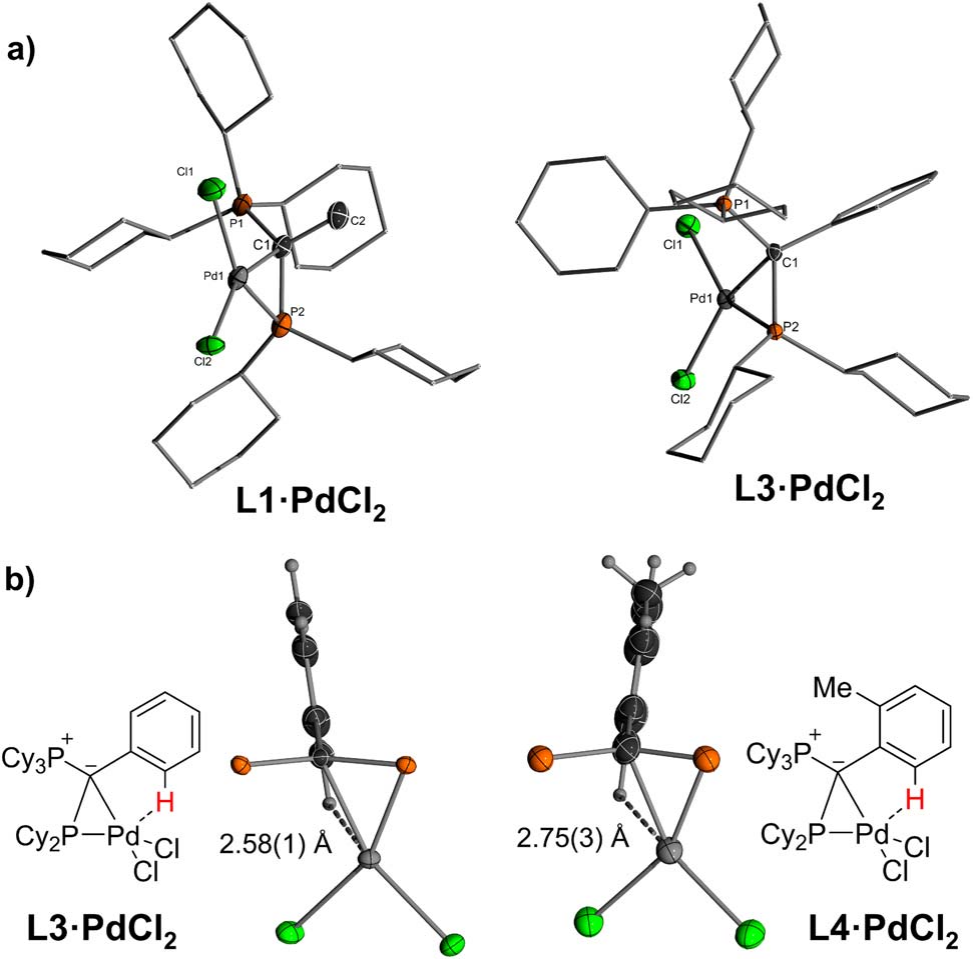
(a) Molecular structures of the PdCl_2_ complexes of keYPhos and joYPhos and (b) drawings of the C–H⋯Pd interaction observed in L3·PdCl_2_ and L4·PdCl_2_. Cy groups have been omitted for clarity.

Single crystals (Fig. 2) of L·PdCl_2_ with keYPhos, joYPhos and pinkYPhos could be obtained by diffusion of pentane into saturated DCM solutions. All complexes feature the same monomeric structure with the unique *κ*C,*κ*P bonding motif of the YPhos ligand as found for the PdBr_2_ and PdI_2_ complexes (Scheme 1). This motif contrasts the usual coordination mode of mono-phosphines towards PdX_2_, which usually leads to halide-bridged dimers or bisphosphine complexes. Monophosphine complexes are in general only formed in the presence of additional donor ligands (*e.g.*E). Yet, some other monophosphine complexes with a *κ*C,*κ*P bonding motif, in which the C-donor only weakly binds to the metal (*e.g.* no carbene), have been reported, amongst phosphines with an additional alkene moiety^29^ and bulky biaryl phosphines, which feature an additional arene metal interaction.^30^ A pallada cyclopropane structure however, has only once been reported by Bertrand and coworkers, yet for an (amino)(phosphino)carbene ligand.^31^ The molecular structures of L·PdCl_2_ feature a planarly coordinated palladium center, whose coordination environment strongly deviates from an ideal square-planar geometry due to the acute C–Pd–P angle of approx. 49° (Table 1). The P–Pd bond lengths (2.145(1) to 2.165(1) Å) are considerably shorter than those found in other YPhos palladium(ii) complexes, such as the L·Pd(allyl)Cl systems or 1 and 2 (2.289(1) to 2.406(1) Å).^25*a*^ The C–Pd bond length is slightly longer than common Pd–C_carbene_ bonds (∼2.0 Å),^22,31^ but in the range of olefin complexes (*e.g.* 2.131(4) Å in L1·Pd(dba)),^24*b*^ thus indicating a weak interaction. Despite the coordination of the ylide moiety to the palladium center, the ylidic center maintains its almost planar geometry. Thus, all L·PdCl_2_ complexes feature a sum of angles around the carbon atom greater than 354°. This corroborates with a weak Pd–C interaction, in line with the relatively long Pd–C distance.

**Table 1:** ^31^P{^1^H} NMR data as well as relevant bond lengths and angles for all L·PdCl_2_ complexes

	L1·PdCl_2_	L2·PdCl_2_	L3·PdCl_2_	L4·PdCl_2_
*δ*_P_ (PCy_3_) [ppm]	37.1	38.7	35.3	38.2
*δ*_P_ (PR_2_) [ppm]	48.6	83.2	53.7	57.4
^2^ *J*_PP_ [Hz]	—	4.7	3.9	5.7
*δ*_C_ (PCP) [ppm]	14.2	20.0	n.d.^a^	n.d.^a^
^1^ *J*_PC_ [Hz]	41.1, 5.1	30.2, 12.7	n.d.^a^	n.d.^a^
P2–Pd [Å]	2.165(1)	—	2.149(1)	2.145(1)
C1–Pd [Å]	2.122(5)	—	2.187(3)	2.186(3)
Pd–Cl1 [Å]	2.412(1)	—	2.436(1)	2.421(1)
Pd–Cl2 [Å]	2.394(1)	—	2.338(1)	2.351(1)
C1–P2 [Å]	1.798(5)	—	1.794(4)	1.798(3)
P1–C1–P2 [°]	124.8(3)	—	124.3(2)	129.4(2)
C1–Pd–P2 [°]	49.6(1)	—	48.9(1)	49.1(1)
C1–P2–Pd [°]	64.0(2)	—	66.7(1)	66.7(1)

a n.d.: The signal could not be detected.

In solution, L·PdCl_2_ feature two singlets or a set of two doublets with very small coupling constants (^2^*J*_PP_ < 6 Hz) in the ^31^P{^1^H} NMR spectrum (Table 1). The signals are substantially low-field shifted compared to those of the free ligands as a result of the coordination of the phosphine to the metal center. Likewise, the ^13^C NMR signals of the ylidic carbon atoms appear at lower field (14.2 ppm in L1·PdCl_2_ and −1.7 ppm in L1) with smaller coupling constants. Interestingly, in the ^1^H NMR spectrum of L3·PdCl_2_ and L4·PdCl_2_, the signal for one of the *ortho* hydrogen atoms of the aryl substituent in the ylide backbone appears at a remarkably lower field (approx. Δ*δ* = 1.7 ppm, Fig. S3 and S4†). This can presumably be explained by a weak Pd–H interaction, which corroborates with short Pd–H distances found in the crystal structure. With 2.58(2) and 2.75(3) Å these distances are shorter or in the range of the sum of the van der Waals radii of palladium and hydrogen (2.73 Å) (Fig. 2b).

To gain further insights into the electronic structures of YPhos–PdCl_2_ complexes, we performed DFT calculations at the PBE0-D3(BJ)/def2-SVP^32^ level of theory (see the ESI† for details) using L1·PdCl_2_ as an outset. The equilibrium geometry is in very good agreement with the structure determined by SC-XRD (Fig. S50†), with the P–Pd bond (2.161 Å) being slightly shorter than the expected bond length for a P–Pd double bond length (2.19 Å), as observed with the experimental structure. In contrast, the C–Pd bond length (2.154 Å) deviates from the experimental measurement by *ca.* 0.03 Å and is clearly longer than expected for a single bond (1.95 Å). Furthermore, the structure exhibits only a small pyramidalization at the carbon atom (Σ∠C = 355°), supporting a weak bonding interaction.

We were particularly interested in the nature of the ylide-palladium chemical bond. Thus, we first analyzed the electronic structure by natural bond orbital (NBO) analysis. Table S25 in the ESI† gathers the most important calculated natural partial charges (*Q*) and Wiberg bond orders (*P*) of the bonding involved in the PdCl_2_ coordination. Natural population analysis (NPA) indicates an electron-rich C1 atom with −0.95*e* and a positively charged P2 atom with +1.22*e*. The complete moiety of PdCl_2_ bears a negative charge of −0.90*e*, mainly located at the chlorine atoms, which are due to the strong donation of the YPhos fragment. The Wiberg bond order values are consistent with a weak single bond character for both, the P–Pd (*P* = 0.54 a.u.) and the C–Pd (*P* = 0.34 a.u.) bond. The NBO analysis leads to a Lewis structure with a lone pair remaining on the C1 atom, supporting a dative interaction between the ylide and the palladium center and thus confirms that the ylide is functioning as a L-type rather than a X-type ligand.

**Fig. 3:**
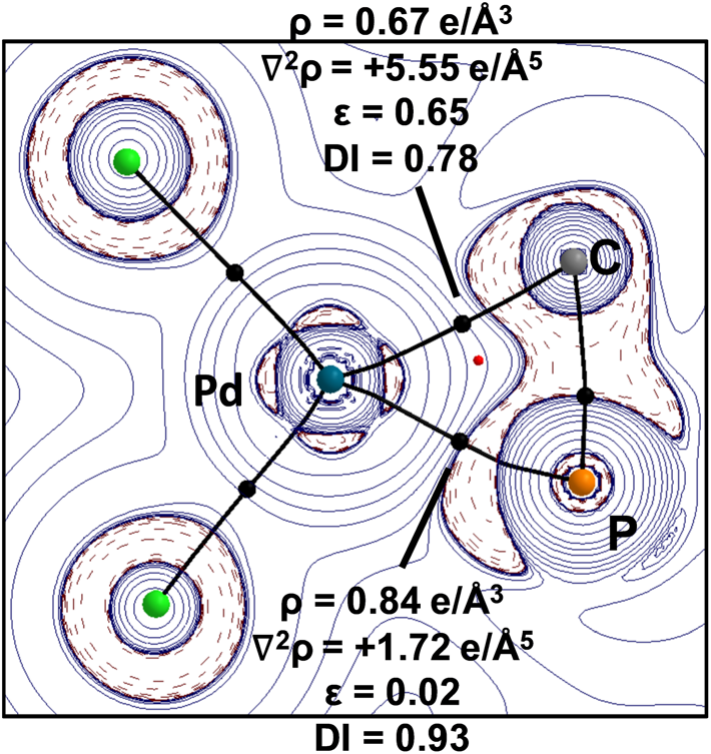
Laplacian distribution of the electron density of L1·PdCl_2_ (PBE0-D3(BJ)/def2-TZVPP//PBE0-D3(BJ)/def2-SVP). Contour line diagrams of the Laplacian distribution ∇^2^*ρ*(*r*) in the Pd–C–P plane. Dashed red lines indicate areas of charge concentration (∇^2^*ρ*(*r*) < 0) while solid blue lines show areas of charge depletion (∇^2^*ρ*(*r*) > 0). The thick solid lines connecting the atomic nuclei are the bond paths and the small dots are the critical points. Bond critical points (in black), Ring critical points (in red).

This is further supported by the analysis of the electron density distribution with atoms in molecules (QTAIM).^33^Fig. 3 depicts the Laplacian distribution ∇^2^*ρ*(*r*) in the C1–Pd–P2 plane. The plot shows an electron accumulation on the phosphorus and carbon atoms localized on the σ-system with an electron density of the bond critical points (BCP) of P–Pd (*ρ*^BCP^ = 0.84 e Å^−3^) and C–Pd (*ρ*^BCP^ = 0.67 e Å^−3^). The corresponding delocalization index (DI) points toward weak single bonds, *i.e.* 0.93 and 0.78 for P–Pd and C–Pd, respectively. It is interesting to note that the electron density at the BCPs and the DI values both suggest that the stronger interaction between the ligand and palladium originates from the phosphine and not from the ylidic carbon center. This observation is well in line with the NBO analysis (see the ESI†) and can be explained by strong donor properties of the YPhos ligand. Notably, the ellipticity of the P–Pd bond fits to a single bond character (*ε* = 0.02), however, the C–Pd bond has a relatively high value, which is a consequence of a small hessian parameter on the three-membered ring plane.

**Fig. 4:**
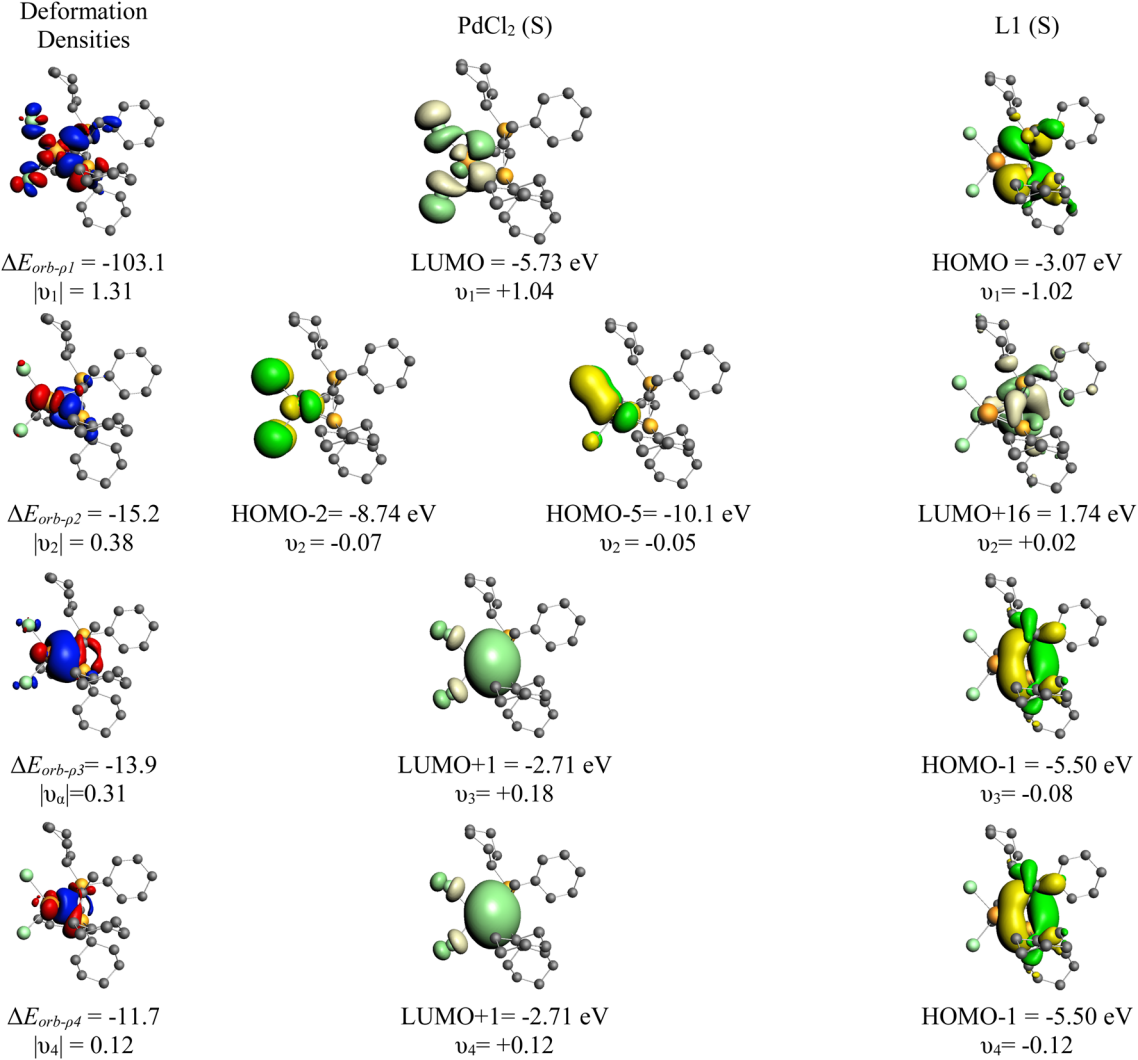
Plot of deformation densities Δ*ρ*_1–3_ (iso value = 0.003) of the pairwise orbital interaction and shape of the most important occupied and vacant orbitals (iso value = 0.03) in YPhos–PdCl_2_ complex with the orbital interaction energies Δ*E*_orb_ (in kcal mol^−1^) and their eigenvalues *ν* (in e). For the deformation densities, the direction of the charge flow is red → blue. The eigenvalues *ν* indicate the amount of donated (negative numbers) and accepted charge (positive numbers). The occupied orbitals are shown in yellow and blue for the different phases, while the unoccupied orbitals are in light yellow and light blue.

More detailed information about the nature of the YPhos–PdCl_2_ coordination was obtained by the Energy Decomposition Analysis (EDA) method.^34^ EDA has proven to be a useful tool to assess the nature of the chemical bond in main group compounds and transition metal compounds.^35,36^ Nonetheless, a recent discussion has been placed about the path function nature of the energy components.^36,37^ Within the EDA scheme, the bond formation (*D*_e_) between two (or more) fragments is dissected in the preparation energy (Δ*E*_prep_) and interaction energy (Δ*E*_int_). The interaction energy can be further divided into different physically meaningful energetic terms, namely, Pauli repulsion (Δ*E*_Pauli_), dispersion (Δ*E*_disp_), electrostatic (Δ*E*_elstat_) and orbital interaction (Δ*E*_orb_, for further details, see the ESI†). Table 2 shows the numerical results of the calculations where PdCl_2_ and the YPhos ligand are both in their neutral and singlet reference state as interacting fragments, although ionic interacting fragments is also possible. The EDA results reveal a strong interaction between the PdCl_2_(S) and YPhos(S) fragment (Δ*E*_int_ = −142.1 kcal mol^−1^). The preparation energy is 39.9 kcal mol^−1^, which is mostly related to the geometrical deformation of the YPhos ligand (29.5 kcal mol^−1^). An examination of the Δ*E*_int_ components suggests a large Coulomb interaction contribution (56.2%) and orbital interaction (40.5%), while the dispersion represents only 3.2% of the total stabilizing interactions. The specific deformation densities as well as the interacting orbitals are shown in Fig. 3 revealing a rather symmetrical bonding between the YPhos ligand and PdCl_2_, with equal contributions from the phosphine and the carbon site.

**Table 2:** E EDA-NOCV at the PBE0-D3(BJ)/TZ2P level of theory^a^

	L1 (S); PdCl_2_ (S)
Δ*E*_int_	−142.1
Δ*E*_Pauli_	282.9
Δ*E*_disp_^b^	−13.6 (3.2%)
Δ*E*_elstat_^b^	−239.0 (56.2%)
Δ*E*_orb total_^b^	−172.3 (40.5%)
Δ*E*_orb HF_	0.3
Δ*E*_orb_	−172.6
Δ*E*_orb-σ_^c^	−103.1 (59.7%)
Δ*E*_orb-π_^c^	−15.2 (8.8%)
Δ*E*_orb-σ_^c^	−13.9 (8.1%)
Δ*E*_orb-pol_^c^	−11.7 (6.8%)
Δ*E*_orb-rest_^c^	−40.4 (23.4%)
Δ*E*_prep PdCl_2__	10.1
Δ*E*_prep L1_	29.5
Δ*E*_prep total_	39.9
*D*_e_	102.2
Fragment electron flow	1.28*e*

a Geometries optimized at the PBE0-D3(BJ)/def2-TZVP level of theory.

b The value in parenthesis gives the percentage contribution to the total attractive interactions Δ*E*_elstat_ + Δ*E*_orb_ + Δ*E*_disp_.

c The values in parenthesis gives the percentage contribution to the total orbital interaction.

### Synthesis and structure of YPhos-based palladate complexes

**Scheme 3:**
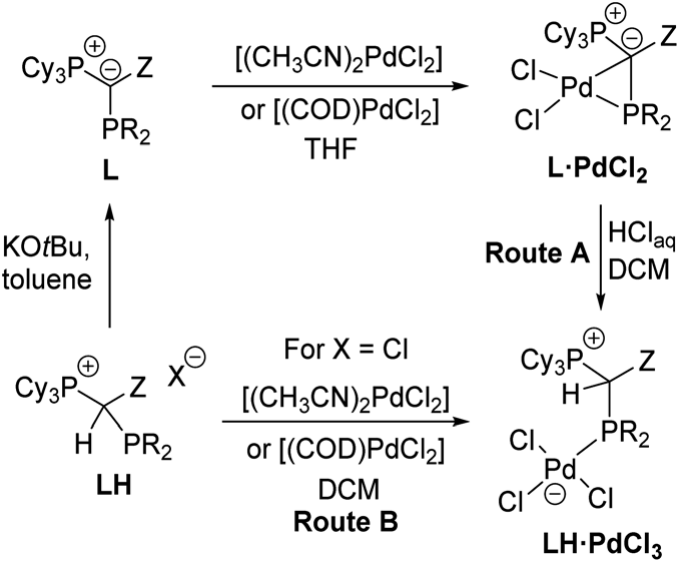
Preparation of the YPhos palladates LH·PdCl_3_.

The unique monomeric structure of the PdCl_2_ complexes led us to explore whether they may also serve as precursors to mononuclear palladate complexes *via* cleavage of the Pd–C bond such as by HCl addition. Indeed, treatment of a solution of the L·PdCl_2_ complexes in DCM with an excess of aqueous HCl gave the zwitterionic products LH·PdCl_3_ as yellow solids in good yields between 62 and 80% (Route A, Scheme 3). Alternatively, the palladate complexes can be obtained in similar purities and yields (69 and 91%) directly from the α-phosphino phosphonium salts L·H and either [(CH_3_CN)_2_PdCl_2_] or [(COD)PdCl_2_] (Route B). However, in this case, the use of the chloride salt of L·H is strictly required to prevent halide scrambling and the formation of mixed bromo/chloro palladates. All LH·PdCl_3_ complexes are extremely robust solids, which do not react with oxygen and water. The stability is also maintained in DCM solutions, even upon addition of water or bubbling of oxygen through a solution of the palladate complexes. Similar to the PdCl_2_ complexes, the palladate complexes show a low solubility in ether or hydrocarbon solvents but are well soluble in chlorinated solvents.

**Fig. 5:**
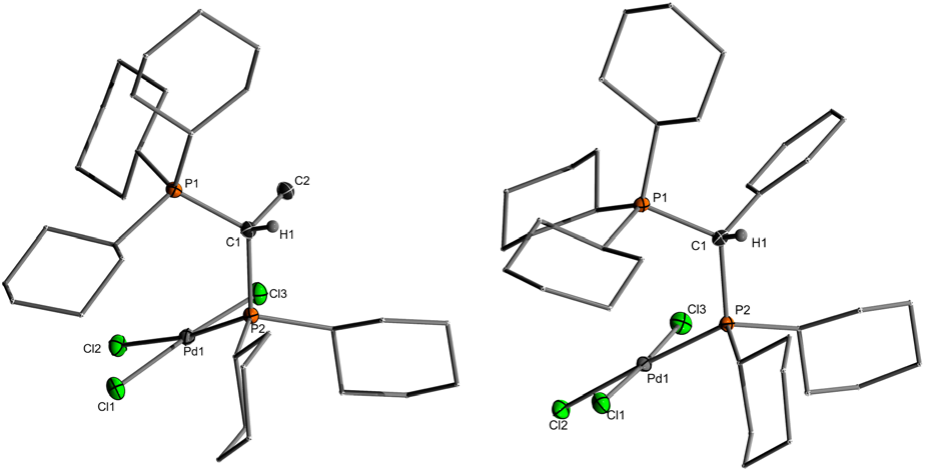
Molecular structures of palladates L1·PdCl_3_ and L3·PdCl_3_.

The palladate complexes were characterized by NMR, elemental and XRD analysis (Table 3). In solution, they are characterized by slightly larger ^2^*J*_PP_ coupling constants (4.7–16.7 Hz) in the ^31^P{^1^H}NMR spectra in comparison to L·PdCl_2_. The phosphine signals are high-field shifted (*e.g.*, from 53.7 ppm in L3·PdCl_2_ to 31.4 ppm in L3H·PdCl_3_), whereas the signals for the phosphonium group appeared at lower field than in L·PdCl_2_ (*e.g.*, 35.3 ppm in L3·PdCl_2_ to 38.5 ppm in L3H·PdCl_3_) (see the ESI† for details). The ^13^C NMR signals of the central carbon center appear slightly more down-field shifted with smaller ^1^*J*_PC_ coupling constants compared to the L·PdCl_2_ complexes due to the protonation of the former carbanionic center and the involved decrease in bond angles. The molecular structures (Fig. 5) confirmed the expected cleavage of the former Pd–C1 contact. This cleavage is accompanied by a significant increase of the C1–P2–Pd1 angle from approx. 65° in the PdCl_2_ complexes to 111.27(6)° to 116.5(1)° in LH·PdCl_3_. Also, the Pd–P2 bond elongates for example from approx. 2.165(1) Å in L1·PdCl_2_ to 2.268(1) Å in the corresponding palladate complex L1H·PdCl_3_. Furthermore, in contrast to L·PdCl_2_ the C1 carbon atom is now clearly pyramidalized with smaller P–C–P angles between 116.2(2)° and 121.2(1)°.

**Table 3:** ^31^P{^1^H} NMR data as well as relevant bond lengths and angles for all LH·PdCl_3_ complexes

	L1H·PdCl_3_	L2H·PdCl_3_	L3H·PdCl_3_	L4H·PdCl_3_
*δ*_P_ (PCy_3_) [ppm]	40.4	43.1	38.5	42.5
*δ*_P_ (PCy_2_) [ppm]	30.5	66.9	31.4	37.0
^2^ *J*_PP_ [Hz]	4.7	10.5	14.5	16.7
*δ*_C_ (PCP) [ppm]	20.7	27.9	34.7	33.0
^1^ *J*_PC_ [Hz]	36.8, —	25.8, 7.5	36.4, 5.3	35.5, 7.9
C1–P2 [Å]	1.891(2)	1.864(6)	1.902(2)	1.925(4)
P2–Pd [Å]	2.2675(5)	2.296(1)	2.2737(6)	2.286(1)
Pd–Cl_longest_ [Å]	2.3853(5)	2.346(1)	2.3750(6)	2.357(1)
Pd–Cl_shortest_ [Å]	2.3150(5)	2.304(2)	2.3128(7)	2.311(1)
P1–C1–P2 [°]	121.2(1)	125.3(3)	118.0(1)	116.2(2)
C1–P2–Pd [°]	111.27(6)	111.9(2)	115.67(7)	116.5(1)

### Applications in catalysis

**Fig. 6:**
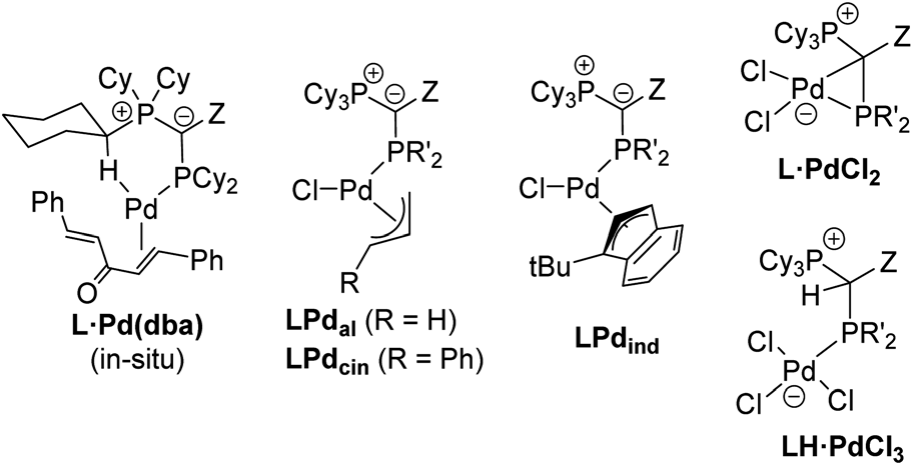
YPhos precatalysts tested in coupling reactions (for YPhos definitions, see Scheme 2). L·Pd(dba) was formed *in situ* by stirring of ligand and Pd_2_dba_3_·dba for 1 h prior to catalysis. Due to its long-term instability L1·Pd_cin_ was also formed *in situ*. The indenyl complex of L2 is not accessible due to steric reasons.

Motivated by the facile synthesis of L·PdCl_2_ and LH·PdCl_3_ and their high stability towards air and moisture, we next evaluated their potential as precatalysts. Their performance was compared with the *in situ* formed palladium(0) complex L·Pd(dba) and established precatalysts, *i.e.* the preformed palladium(ii) precatalysts L·P_al_, L·Pcin, and L·P_ind_ (Fig. 6, see the ESI† for their synthesis).^25*b*^ All complexes were tested in a series of palladium catalyzed C–N and C–C bond formation reactions (Fig. 5).

**Fig. 7:**
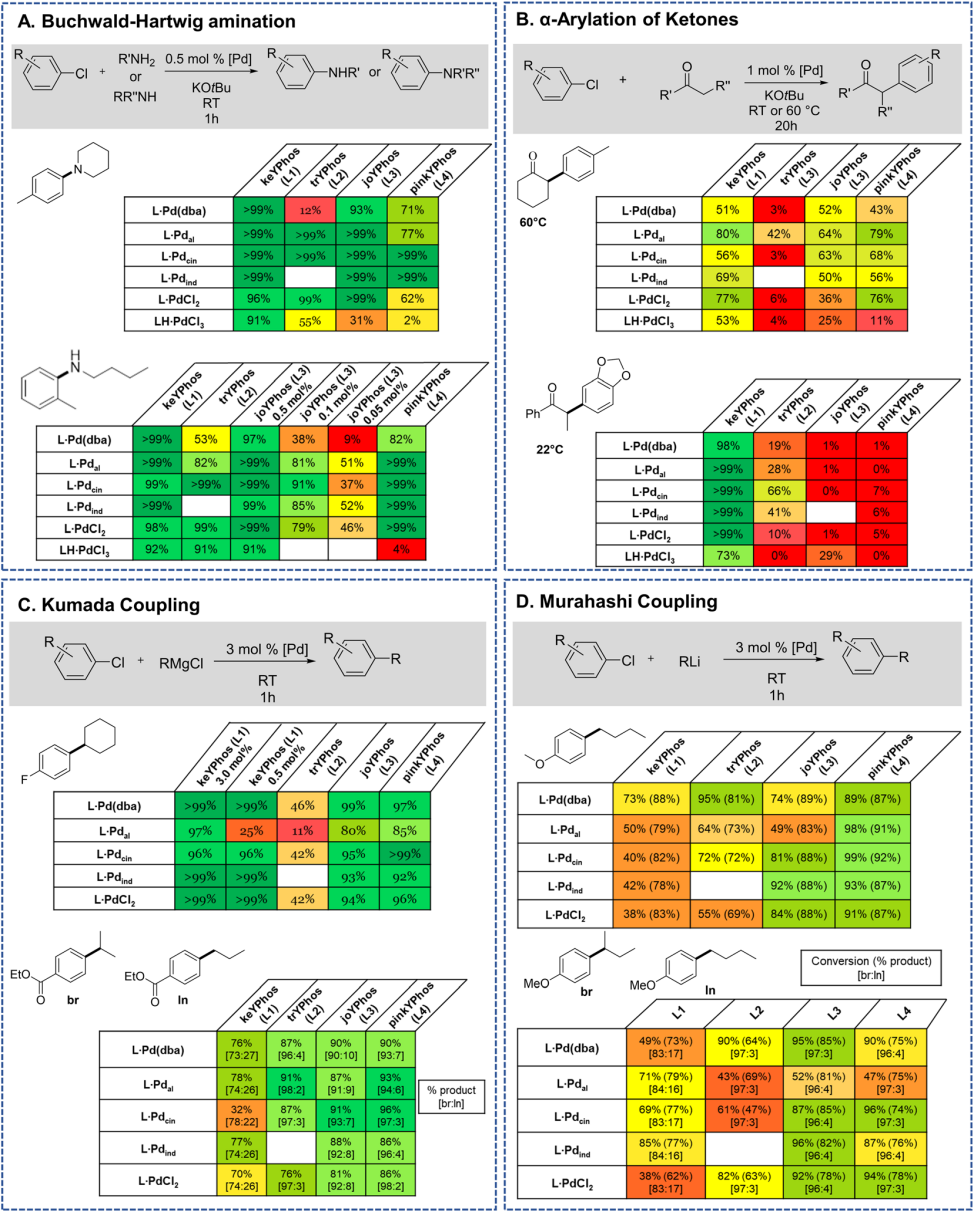
Comparison of the YPhos–PdCl_2_ and palladate complexes with other YPhos precatalysts (depicted in Fig. 4) in their application in (A) Buchwald–Hartwig aminations, (B) α-arylation of ketones, (C) Kumada and (D) Murahashi–Feringa coupling reactions. Detailed information about the reaction conditions are given in the ESI.† Yields are GC yields obtained after the given reaction time.

Previous studies have shown that Buchwald–Hartwig aminations of aryl chlorides are efficiently catalyzed by YPhos complexes with high selectivities under mild conditions.^25*b*^ To evaluate the potential of the L·PdCl_2_ and LH·PdCl_3_ complexes, two amination reactions with aryl chlorides of different steric profile were chosen, one with a secondary amine (4-chlorotoluene + piperidine) and one with a primary amine (2-chlorotoluene + *n*-butylamine). Earlier studies have revealed a higher activity of the η^3^-allyl-type YPhos palladium precatalysts compared to the *in situ* formed L·Pd(dba) complexes.^25*b*^ To our delight, similar activities were reached with the simple PdCl_2_ complexes (Fig. 7a). Full conversion was reached in both reactions with 0.5 mol% using the PdCl_2_ complexes of keYPhos, trYPhos and joYPhos at room temperature. Also, at lower catalyst loadings higher yields than with the Pd(0) precursors L·Pd(dba) and similar yields than obtained with the allyl-systems were reached. Importantly, this performance was reached already at room temperature, thus showing that catalyst activation proceeds facile under the reaction conditions. The performance of L·PdCl_2_ is – despite the similarity of the type of precatalyst – clearly superior to that of the commercially available Pd-PEPPSI-IPent catalyst. Under the same reaction conditions as in Fig. 5A, this catalyst only achieves 36% conversion for the amination of 4-chlorotoluene with piperidine after 1 h (40% after 3 h) at room temperature (see the ESI† for details).

**Fig. 8:**
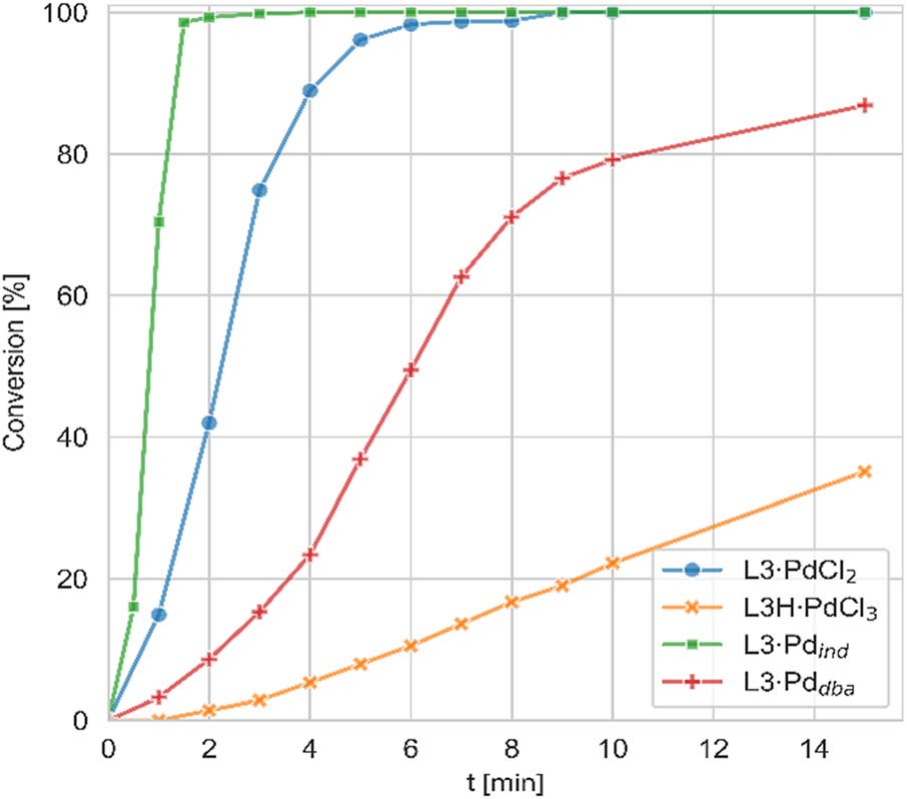
Conversion time plots for the C–N coupling of *p*-toylchloride with piperidine with different precatalysts of joYPhos (L3). Conditions: 0.5 mol% precatalyst, chloride : amine = 1 : 1.1; 1.5 eq. KO*t*Bu, THF, room temperature.

In contrast to the PdCl_2_ complexes, the LH·PdCl_3_ complexes showed a lower activity. We assume that for the formation of the active species the palladate complexes first need to undergo dehydrohalogenation to the corresponding L·PdCl_2_ complex, since the cationic ligand would be too electron-poor to enable the oxidative addition of aryl chlorides at room temperature. However, deprotonation of the phosphonium backbone is presumably slow due to steric crowding, which results in the slow or incomplete catalyst activation. To improve the catalyst activation, different reaction conditions were tested, *i.e.* using different bases as well as chloride abstraction reagents. Yet, no reliable protocol could be established for all ligands. In general, best results were obtained with the smallest ligand, keYPhos, suggesting that steric shielding of the C1 carbon atom by the *Z* substituent indeed complicates deprotonation. This different shielding of the hydrogen atom at the C1 atom by the different ligand structures is illustrated by the van-der-Waals plots (Fig. S3†) of the molecular structures. Whereas H1 is still accessible from the side of the methyl group in L1, it is completely shielded by the *ortho*-tolyl group. Accordingly, L4·PdCl_2_ performed worst due to the more demanding *ortho*-tolyl group, which almost completely prevents catalyst activation with KO*t*Bu as base within 1 h at room temperature. The slower activation of the palladate complexes and their moderate performance in comparison to the PdCl_2_ systems was also confirmed in kinetic studies. Whereas for the amination of *p*-tolylchloride with piperidine at room temperature less than 40% conversion were observed with the joYPhos complexL3H·PdCl_3_, the corresponding PdCl_2_ complex quickly formed the active species to reach full conversion already after 6 min reaction time (Fig. 8).

This fast activation was also seen for the PdBr_2_ system indicating that there is no significant impact of the nature of the halide on the catalytic activity (see Fig. S6†). The activity of L3·PdCl_2_ compared well with the indenyl precatalyst L3·Pd_ind_, which showed a slightly higher activity to reach full conversion already after 2 min. Nonetheless, L3·PdCl_2_ is clearly more active than the catalyst formed from the free ligand and Pd_2_(dba)_3_ which showed a longer induction period and only reached 97% conversion after 60 min. Overall, the PdCl_2_ complexes exhibit similarly high activities in C–N coupling reactions than the more complicated Pd(ii) allyl-type complexes, thus representing a more stable and cost efficient alternative, which furthermore does not produce any further organic by-product.

The effectiveness of the L·PdCl_2_ complexes was also observed in the α-arylation of ketones, in which – according to previous studies^38^ – the keYPhos ligand performed best in comparison to the other YPhos ligands. Again, the corresponding palladate complex gave slightly lower yields. To our delight, also Kumada and Murahashi coupling reactions were easily catalyzed by the PdCl_2_ complexes, demonstrating that organolithium and Grignard reagents quickly produce the active species even at room temperature. Whereas for the Kumada coupling all ligands worked well, the Murahashi coupling was only effectively mediated by the aryl substituted YPhos ligands, joYPhos (L3) and pinkYPhos (L4). This observation also agrees well with the higher stability of these complexes observed in solution. We assume that this increased stability is the reason for the improved performance of these catalysts. In contrast, the more electron-rich P*t*Bu_2_ substituted trYPhos ligand (L2) – which should facilitate the usually rate-limiting oxidative addition and thus exhibit higher activities – gave lower or inconsistent yields not only for the Murahashi but most other coupling reactions. This varying performance thus most likely originates from competing activity and stability trends.

**Fig. 9:**
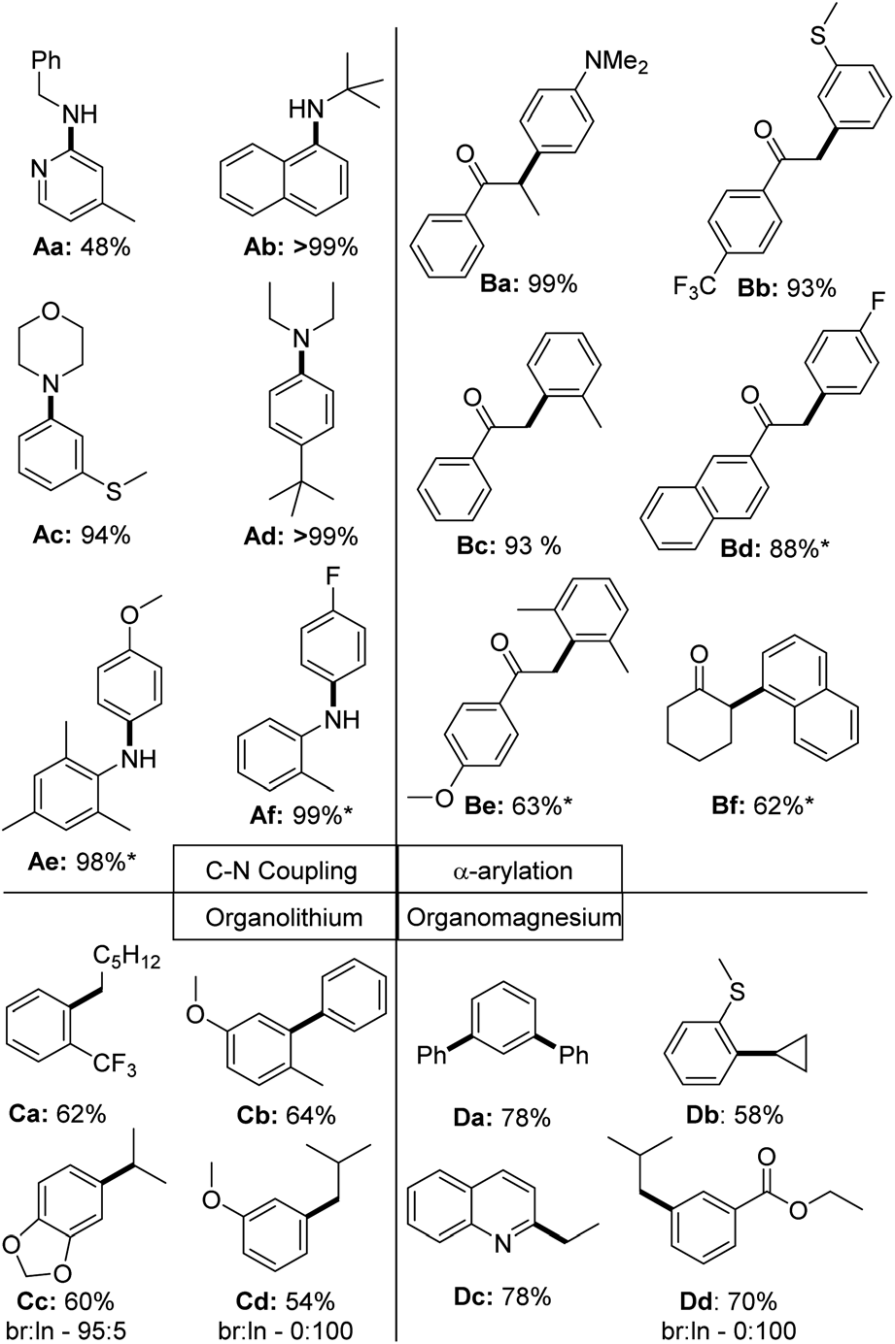
Scope of the application of PdCl_2_ complexes in C–N and C–C coupling reactions of aryl chlorides. * = Reaction was performed at 60 °C; br = branched product; ln = linear product. Used precatalyst: L1·PdCl_2_ for Ab, Ae, Af, Ba – Bf; L2·PdCl_2_ for Aa, Ad; L3·PdCl_2_ for Ac and L4·PdCl_2_ for Ca – Cd, Da – Dd. The same reactions conditions as used in the benchmarking reactions shown in Fig. 5 have been used. Further details are provided in the ESI.†

To finally test the utility of the palladium chloride complexes in catalysis, we attempted the isolation of a series of different coupling products (Fig. 9). In case of the C–N couplings, perfect yields were obtained for a selection of aliphatic and aromatic amines with 0.5 mol% of the PdCl_2_ complex. Sole exception represents the 2-pyridyl benzylamine Aa, which could only be isolated in 48% yield under the reaction conditions (no optimization attempted). All other aryl amines could be isolated in high yields >90%. In case of the α-arylation of ketones the coupling worked well with electron-rich (Ba) and electron-poor (Bd) aryl chlorides. Lower yields were observed with sterically demanding chlorides, such as with the di-*ortho*-methyl substituted (Be) or naphthyl chloride (Bf). In case of the Kumada and Murahashi coupling moderate to good yields were reached without further optimizing the reaction conditions.^39^ Further substrates tested, but not isolated are given in the ESI (Fig. S1†).

Given the efficiency of the YPhos–PdCl_2_ complexes, we were interested in the mode of activation of these precatalysts. Preparation of the catalyst mixture already indicated a fast activation by addition of the base. To test whether treatment with a base already results in the reduction of Pd(ii) to Pd(0), we performed stoichiometric experiments by treating the keYPhos (L1) PdCl_2_ complex in THF with two equiv. KO*t*Bu. Addition of the base instantaneously resulted in the dissolution of the PdCl_2_ complex and the darkening of the reaction mixture. ^31^P NMR spectroscopy indicated the initial formation of a new species, which shows two doublets at 51.2 and 77.5 ppm with a coupling constant of 98 Hz (see the ESI† for details). This intermediate species reacts further to form palladium black along with the palladium(0) bisphosphine complex [(L1)_2_Pd],^27^ which is characterized by two signals at 18.9 and 30.3 ppm and which remains the main product after a longer reaction time. This observation clearly demonstrates that L·PdCl_2_ is readily reduced to Pd(0) species by the metal base. No further substrates, which might give rise to undesired byproducts, are necessary for the activation.

## Conclusions

In conclusion, we reported on the formation of simple palladium dihalide complexes with different YPhos ligands and their application in Pd coupling chemistry. The PdCl_2_ complexes are easily generated from the free ligands and the metal salt precursors and form unusual monomeric structures through coordination of the metal by the phosphine and the ylidic carbon atom. The palladacyclopropane motif easily undergoes Pd–C bond cleavage such as by addition of HCl, thus giving rise to zwitterionic palladate complexes with a cationic phosphine ligand. The PdCl_2_ and palladate complexes of the YPhos ligands exhibit remarkable stabilities in the solid state and in solution even towards air and moisture, thus making them easy-to-handle and applicable precatalysts. Despite their simplicity and stability, the PdCl_2_ precatalysts exhibited remarkably activities in C–N and C–C coupling reactions. According to kinetic studies, catalyst activation proceeds fast under basic conditions at room temperature for the PdCl_2_ complexes, but slower for the palladate systems. Accordingly, the YPhos–PdCl_2_ showed a superior performance and could compete with more complex Pd(ii) precatalysts reported earlier. Hence, these complexes represent easy to synthesize and more stable and cost efficient, but highly active alternatives to other specially designed precatalysts.

## Author contributions

I. R. has synthesized all complexes, performed most of the catalysis reactions and solved most of the crystal structures. L. K. has supported I. R. with the synthesis of the complexes. J. L. has performed the down-scaling reactions. T. S. and A. S. have solved some of the crystal structures. D. M. A. has performed the theoretical calculations. V. H. G. has designed the study and prepared the manuscript together with I. R.

## Conflicts of interest

A patent application has been filed on this work in collaboration with Umicore AG & Co KG.

## Supplementary Material

SC-013-D2SC04523K-s001

SC-013-D2SC04523K-s002
