# Supplementary material for: Formation of exceptional monomeric YPhos–PdCl2 complexes with high activities in coupling reactions
Source: Chem Sci. 2022 Oct 26;13(45):13552–62. doi: 10.1039/d2sc04523k (PMC9683020; doi:10.1039/d2sc04523k)
Supplement: SC-013-D2SC04523K-s001 [file SC-013-D2SC04523K-s001.pdf]

Electronic Supplementary Material (ESI) for Chemical Science.
This journal is © The Royal Society of Chemistry 2022

Formation of Exceptional Monomeric YPhos-$PdCl_2$ Complexes with High Activities in Coupling Reactions

Ilja Rodstein,[a] Leif Kelling,[a] Julian Löffler,[a] Thorsten Scherpf,[a] Abir Sarbajna,[a] Diego M. Andrada,[b] Viktoria H. Gessner[a]*

[a]Faculty of Chemistry and Biochemistry, Chair of Inorganic Chemistry II, Ruhr University Bochum, Universitätsstr. 150, 44801 Bochum (Germany)

[b] General and Inorganic Chemistry Department, University of Saarland, Campus C4.1, 66123 Saarbruecken, Germany.

Index

1. Experimental Procedures 2

1.1 General information 2

1.2 Oxidative Addition Reactions 3

1.3 Synthesis of YPhos Dihalide Complexes 5

1.4 Synthesis of LH·$PdCl_3$ Complexes 9

1.5 Procedures of the Catalytic Reactions 13

1.6 Isolated Cross-Coupling Products 16

2. Comments and Additional Information 24

2.1 Catalyst Activation 24

2.2 Van-der-Waals plots of L1H·$PdCl_3$ and L4H·$PdCl_3$ 25

2.3 H–Pd interaction in L3·$PdCl_2$ and L4·$PdCl_2$ 25

2.4 Reaction Kinetics 26

3. NMR Spectra 28

3.1 NMR Spectra of the Ligands and Complexes 28

3.2 NMR Spectra of the Isolated Products from Catalysis 45

4. Crystal Structure Determination 59

4.1 General Information 59

4.2 Details on the crystal structure analyses 64

5. Computational Studies 94

5.1 Computational details 94

5.2 xyz coordinations (in Å) and Energies (in Hartree) 104

6. References 107

1. Experimental Procedures

1.1 General information

Syntheses and chemicals.

All experiments were carried out under a dry, oxygen-free argon atmosphere using standard *Schlenk* techniques. Argon atmosphere was generated by gas bottles, which were filled up by *Air Liquide*.

Involved solvents were dried using a *MBraun SPS-800* device (tetrahydrofuran, toluene, dichloromethane, pentane, hexane, diethyl ether, acetonitrile) or dried in accordance with standard procedures. The solvents were stored in flasks with a *J. Young* valve under an argon atmosphere over molecular sieve (3Å or 4 Å). The amount of water was checked regularly *via* a *Karl Fischer* titration. Reactions on air were performed in solvents at analytical grade.

All reagents were purchased from Sigma-Aldrich, ABCR, Rockwood Lithium, Acros Organics, TCI Chemicals and used without further purification. Umicore donated all palladium complexes used in this work. **1** was synthesized according to a previous report.[1] The YPhos ligands were synthesized according to previous reports (keYPhos[2], trYPhos[3], joYPhos[4], pinkYPhos[5]) and named as follows:

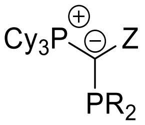

Z = Me, R = Cy, **keYPhos, L1**
Z = Me, R = *t*Bu, **trYPhos, L2**
Z = Ph, R = Cy, **joYPhos, L3**
Z = *o*Tol, R = Cy, **pinkYPhos, L4**

Compound characterizations

^{1}H, ^{13}C{^{1}H} and ^{31}P{^{1}H} NMR spectra were recorded on Avance-400 spectrometer at 25 °C if not mentioned otherwise. ^{19}F{^{1}H} NMR spectra were measured on DPX-250 or Spinsolve 80 spectrometer. All values of the chemical shift are in ppm regarding the δ-scale. All spin-spin coupling constants (*J*) are printed in Hertz (Hz). To display multiplicities and signal forms correctly the following abbreviations were used: s = singlet, d = doublet, m = multiplet, dd = doublet of doublet, ddd = doublet of doublet of doublet, br = broad signal, vbr = very broad signal. Signal assignments were supported by HSQC (^{1}H/ ^{13}C) and HMBC (^{1}H/ ^{13}C, ^{1}H/ ^{31}P) correlation experiments and literature studies on similar compounds. The spectra were evaluated with the program *MestreNova 14.3.0* by *Mestrelab Research*.

Molecular structures were measured *via* single crystal X-ray diffraction on an XtaLAB Synergy *(*HyPix detector*)* at 100 K. Measurement was adjusted with *CrysAlisPro* and refined with *SHELXL 2018*.

GC/MS analyses were carried out with an Agilent 8890 GC and 5977B MSD system using an HP-5 capillary column (Phenyl methyl siloxane, 30 m × 320 × 0.25, 100/2.3-

30-300/3, 2 min at 60 °C, heating rate 30 °C/min, 3 min at 300 °C). Yields were determined by GC-FID using *n*-tetradecane as the internal standard.

HRMS analyses were carried out with an Agilent 8890 GC and Q-TOF system and measured by Dr. Michael Wüstenfeld.

Elemental analyses were performed by Manuela Winter, Dr. Harish Parala and Maurice Paaßen and Fabian Töpfer on an Elementar vario MICRO-cube elemental analyzer.

IR-Spectra were recorded on a Thermo Nicolet iS5 FT-IR spectrometer in the transmission mode with a Specac "Omni-cell" with an ATR module at 22 °C.

Column chromatography was performed on a Reveleris X2 (BÜCHI) flash chromatography system using Reveleris packed columns.

Melting points were collected on a Stuart SMP 30 with a heat-up speed of 2 °C per minute.

All cyclohexyl groups were labeled according to the following scheme:

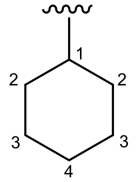

All *ortho*-tolyl groups were labeled according to the following scheme:

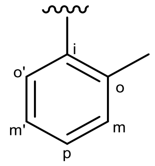

1.2 Oxidative Addition Reactions

1.2.1 Synthesis of **2**

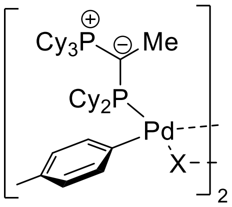

keYPhos (15.0 mg, 0.030 mmol, 1 eq.) and Pd_2dba_3·dba (22.3 mg, 0.033 mmol, 1.1 eq.) were suspended in a *J. Young* NMR tube in THF-d_8. After 30 minutes, a large excess of *para*-bromo toluene (>20 eq.) was added, and the mixture was shaken for 4 hours. The solvent was slowly evaporated overnight in the glovebox to yield yellow crystals, suitable for single X-ray diffraction to obtain a molecular structure in the solid-state.

$^{31}P\{^1H\}$ NMR (162 MHz, THF-d_8) δ = 16.4 (d, $^2J_{PP}$ = 54.2 Hz, *P*Cy_2), 31.4 (d, $^2J_{PP}$ = 54.2 Hz, *P*Cy_3) ppm.

The isolation of complex **2** in pure form was not successful. Stirring of the complex in solution for led to the slow formation of **L1·PdBr₂**. After 2 days in THF solution, **L1·PdBr₂** was isolated in pure form in 67% yield.

1.2.2 Synthesis of **L1·PdBr$_2$**

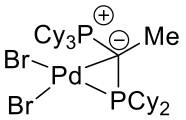

keYPhos (L1) (300 mg, 0.59 mmol, 1 eq.) and Pd_2dba_3·dba (405 mg, 0.059 mmol, 1 eq.) were dissolved in 5 ml of THF. The solution was stirred for one hour. 2-bromotoluene (0.358 ml, 508 mg, 3.0 mmol, 5 eq.) was added and the mixture was stirred overnight. The formed dark yellow solid was filtered off and washed with three portions of 10 ml of THF. The solid was dissolved in 10 ml of dichloromethane and the solution was filtered through a filter canula to remove palladium impurities. The solvent was removed in vacuo and the crude product solidified by addition of 20 ml of diethyl ether. The diethyl ether was removed with syringe and the solid was dried *in vacuo* for three hours. 404 mg (0.52 mmol, 88%) of a light yellow solid were isolated.

^{1}H NMR (400 MHz, CD_2Cl_2) δ = 1.18 – 1.53 (m, 15H, $CH_{2,\ PCy2,\ H3\ +\ H4\ +\ PCy3,\ H3\ +\ H4}$), 1.48 – 1.99 (m, 29H, $CH_{PCy2,\ H1}$+ $CH_{2,\ PCy2,\ H2\ +\ H3\ +\ H4\ +\ PCy3,\ H2\ +\ H3\ +\ H4}$ + CH_3), 2.02 – 2.11 (m, 1H, $CH_{2,\ PCy2,\ H2}$), 2.26 – 2.35 (m, 4H, $CH_{2,\ PCy2,\ H2\ +\ PCy3,\ H2}$), 2.35 – 2.44 (m, 1H, $CH_{2,\ PCy2,\ H2}$), 2.48 – 2.60 (m, 4H, $CH_{2,\ PCy3,\ H2}$ + $CH_{PCy2,\ H1}$), 2.60 – 2.85 (m, 4H, $CH_{2,\ PCy2,\ H2}$ + $CH_{PCy3,\ H1}$) ppm.

^{13}C{^{1}H} NMR (101 MHz, CD_2Cl_2) δ = 17.2 (dd, $^1J_{CP}$ = 41.7 Hz, $^1J_{CP}$ = 6.4 Hz, P–C^-–P), 21.4 (CH_3), 25.7 (d, $^4J_{CP}$ = 2.0 Hz, $CH_{2,\ PCy2,\ C4}$), 25.8 (d, $^4J_{CP}$ = 1.7 Hz, $CH_{2,\ PCy3,\ C4}$), 26.0 (d, $^4J_{CP}$ = 1.9 Hz, $CH_{2,\ PCy2,\ C4}$), 27.1 – 27.6 (m, $CH_{2,\ PCy3,\ C3}$), 27.6 – 27.9 (m, $CH_{2,\ PCy2,\ C3}$), 28.2 (d, $^3J_{CP}$ = 16.8 Hz, $CH_{2,\ PCy2,\ C3}$), 28.6 (d, $^2J_{CP}$ = 2.1 Hz, $CH_{2,\ PCy3,\ C2}$), 29.2 – 30.1 (m, $CH_{2,\ PCy2,\ C2\ +\ PCy3,\ C2}$), 31.3 (d, $^2J_{CP}$ = 7.9 Hz, $CH_{2,\ PCy2,\ C2}$), 31.9 (d, $^2J_{CP}$ = 4.6 Hz, $CH_{2,\ PCy2,\ C2}$), 32.2 (d, $^2J_{CP}$ = 4.0 Hz, $CH_{2,\ PCy2,\ C2}$), 36.7 (d, $^1J_{CP}$ = 38.8 Hz, $CH_{PCy3,\ C1}$), 37.8 (d, $^1J_{CP}$ = 20.7 Hz, $CH_{PCy2,\ C1}$), 39.9 (d, $^1J_{CP}$ = 14.9 Hz, $CH_{2,\ PCy2,\ C1}$) ppm.

^{31}P{^{1}H} NMR (162 MHz, CD_2Cl_2) δ = 36.5 (*P*Cy_3), 51.6 (*P*Cy_2) ppm.

CHNS Calculated: C: 49.85, H: 7.58. Measured: C: 49.78, H: 7.49.

IR (ATR) ṽ = 2925 (vs), 2850 (s), 1638 (w), 1444 (s), 1379 (w), 1347 (w), 1295 (w), 1268 (w), 1219 (w), 1175 (w), 1115 (w), 1072 (w), 1026 (w), 998 (s), 916 (w), 888 (s), 888 (s), 850 (vs), 750 (w), 727 (m), 573 (m), 529 (s), 513 (s) cm^{-1}.
m.p. 188.8 °C (decomposition).

1.2.3 Synthesis of **L1·PdI$_2$**

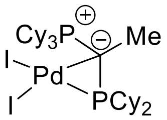

keYPhos (L1) (300 mg, 0.59 mmol, 1 eq.) and Pd_2dba_3·dba (405 mg, 0.059 mmol, 1 eq.) were dissolved in 5 ml of THF. The solution was stirred for one hour. 4-iodotoluene (0.386 ml, 648 mg, 3.0 mmol, 5 eq.) was added and the mixture was stirred overnight. The formed dark yellow solid was filtered off and washed with three portions of 10 ml of THF. The solid was dissolved in 10 ml of dichloromethane and the solution was filtered through a filter canula to remove palladium impurities. The solvent was removed in vacuo and the crude product

solidified by addition of 20 ml of diethyl ether. The diethyl ether was removed with syringe and the solid was dried *in vacuo* for three hours. 443 mg (0.51 mmol, 86%) of a light yellow solid were isolated.

^{1}H NMR (400 MHz, CD_2Cl_2) δ = 1.12 – 1.55 (m, 15H, $CH_{2,\ PCy2,\ H3\,+\,H4\,+\,PCy3,\ H3\,+\,H4}$), 1.56 – 2.04 (m, 30H, $CH_{PCy2,\ H1}$ + $CH_{2,\ PCy2,\ H2\,+\,H3\,+\,H4\,+\,PCy3,\ H2\,+\,H3\,+\,H4}$ + CH_3), 2.04 – 2.11 (m, 1H, $CH_{2,\ PCy2,\ H2}$), 2.21 – 2.40 (m, 4H, $CH_{2,\ PCy2,\ H2\,+\,PCy3,\ H2}$), 2.46 – 2.64 (m, 4H, $CH_{PCy2,\ H1}$ + $CH_{2,\ PCy3,\ H2}$), 2.63 – 2.91 (m, 4H, $CH_{PCy3,\ H1}$+ $CH_{2,\ PCy2,\ H2}$) ppm.

^{13}C{^{1}H} NMR (101 MHz, CD_2Cl_2) δ = 19.9 (dd, $^1J_{CP}$ = 41.8 Hz, $^1J_{CP}$ = 9.2 Hz, P–C^-–P), 22.4 (CH_3), 25.7 (d, $^4J_{CP}$ = 2.0 Hz, $CH_{2,\ PCy2,\ C4}$), 25.9 (d, $^4J_{CP}$ = 1.7 Hz, $CH_{2,\ PCy3,\ C4}$), 26.2 (d, $^4J_{CP}$ = 2.0 Hz, $CH_{2,\ PCy2,\ C4}$), 27.5 (d, $^3J_{CP}$ = 2.6 Hz, $CH_{2,\ PCy3,\ C3}$), 27.6 (d, $^3J_{CP}$ = 2.4 Hz, $CH_{2,\ PCy3,\ C3}$), 27.7 ($CH_{2,\ PCy2,\ C3}$), 27.8 (d, $^3J_{CP}$ = 2.5 Hz, $CH_{2,\ PCy2,\ C3}$), 28.0 (d, $^3J_{CP}$ = 9.0 Hz, $CH_{2,\ PCy2,\ C3}$), 28.3 (d, $^3J_{CP}$ = 16.7 Hz, $CH_{2,\ PCy2,\ C3}$), 29.1 (d, $^2J_{CP}$ = 3.7 Hz, $CH_{2,\ PCy3,\ C2}$), 29.7 ($CH_{2,\ PCy2,\ C2}$), 30.0 (d, $^2J_{CP}$ = 4.4 Hz, $CH_{2,\ PCy3,\ C2}$), 31.7 (d, $^2J_{CP}$ = 8.3 Hz, $CH_{2,\ PCy2,\ C2}$), 32.0 (d, $^2J_{CP}$ = 5.5 Hz, $CH_{2,\ PCy2,\ C2}$), 33.1 (d, $^2J_{CP}$ = 4.1 Hz, $CH_{2,\ PCy2,\ C2}$), 37.7 (d, $^1J_{CP}$ = 38.7 Hz, $CH_{PCy3,\ C1}$), 38.6 (d, $^1J_{CP}$ = 20.4 Hz, $CH_{PCy2,\ C2}$), 40.1 (d, $^1J_{CP}$ = 13.5 Hz, $CH_{PCy2,\ C1}$) ppm.

^{31}P{^{1}H} NMR (162 MHz, CD_2Cl_2) δ = 35.1 (PCy_3), 56.5 (PCy_2) ppm.

CHNS Calculated: C: 44.43, H: 6.76. Measured: C: 44.23, H: 6.65.

IR (ATR) ṽ = 2921 (s), 2849 (s), 1444 (s), 1266 (w), 1175 (w), 1113 (m), 997 (m), 887 (m), 849 (vs), 750 (w), 729 (m), 577 (m), 527 (s), 509 (s) cm^{-1}.
m.p. 192.7 °C (decomposition).

1.3 Synthesis of YPhos Dihalide Complexes

1.3.1 Synthesis of L1·$PdCl_2$

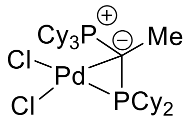

keYPhos (L1) (1.00 g, 1.98 mmol, 1.05 eq.) and [$(CH_3CN)_2PdCl_2$] (0.49 g, 1.89 mmol, 1.00 eq.) were suspended in THF (30 ml). The red suspension was stirred for two days at room temperature. The precipitated solid was filtered through a *Schlenk* frit and washed three times with THF (10 ml), and dried *in vacuo.* The product was obtained as a yellow solid (1.24 g, 1.81 mmol, 96%).

Alternative synthesis pathway starting from $PdCl_2$

keYPhos (0.50 g, 1.00 mmol, 1.0 eq.) and $PdCl_2$ (0.18 g, 1.00 mmol, 1.0 eq.) were suspended in a mixture of 15 ml dry THF and 15 ml dry acetonitrile. The mixture was stirred overnight at room temperature. The solution was filtered through a filter canula, and 20 ml of dry pentane was added to the mixture to form a green-black suspension. The solid was filtered off and washed with two portions of 10 ml of dry THF. The product, which contained significant amounts of palladium black, was obtained as a dark green solid (0.35 g, 0.51 mmol, 51%).

^{1}H NMR (400 MHz, CD_2Cl_2) δ = 1.10 – 1.54 (m, 15H, C*H*$_2$, $_{PCy3, H4 + PCy2, H3 + H4}$), 1.55 – 2.18 (m, 30H, C*H*, $_{PCy2, H1}$ + C*H*$_2$, $_{PCy3, H2 + H3 + H4, PCy2, H2 + H3 + H4}$ + C*H*$_3$), 2.31 (d, $^{3}J_{HH}$ = 12.0 Hz, 5H, C*H*$_2$, $_{PCy2, H2 + PCy3, H3,}$), 2.47 – 2.83 (m, 8H, C*H*$_2$, $_{PCy3, H2 + PCy2, H2}$ + C*H*, $_{PCy3, H1 + PCy2, H1}$) ppm.

^{13}C{^{1}H} NMR (101 MHz, CD_2Cl_2) δ = 14.2 (dd, $^{1}J_{CP}$ = 41.1 Hz, $^{1}J_{CP}$ = 5.1 Hz, P-*C*$^{-}$-P), 21.2 (*C*H$_3$), 26.0 (d, $^{4}J_{CP}$ = 1.9 Hz, *C*H$_2$, $_{PCy2, C4}$), 26.1 (d, $^{4}J_{CP}$ = 1.7 Hz, *C*H$_2$, $_{PCy3, C4}$), 26.3 (d, $^{4}J_{CP}$ = 2.0 Hz, *C*H$_2$, $_{PCy2, C4}$), 27.3 – 28.0 (m, C*H*$_2$, $_{PCy2, C3}$), 28.3 (d, $^{3}J_{CP}$ = 17.1 Hz, *C*H$_2$, $_{PCy2, C3}$), 28.5 (d, $^{2}J_{CP}$ = 3.6 Hz, *C*H$_2$, $_{PCy3, C3}$), 29.7 (d, $^{2}J_{CP}$ = 4.2 Hz, *C*H$_2$, $_{PCy3, C2}$), 29.7 (d, $^{2}J_{CP}$ = 2.7 Hz, *C*H$_2$ $_{PCy2, C2}$), 31.2 (d, $^{3}J_{CP}$ = 7.9 Hz, *C*H$_2$, $_{PCy2, C2}$), 31.8 (d, $^{2}J_{CP}$ = 4.4 Hz, *C*H$_2$, $_{PCy2, C2}$), 32.1 (d, $^{2}J_{CP}$ = 3.9 Hz, *C*H$_2$, $_{PCy2, C2}$), 36.3 (d, $^{1}J_{CP}$ = 39.2 Hz, *C*H, $_{PCy3, C1}$), 37.3 (d, $^{1}J_{CP}$ = 20.3 Hz, *C*H, $_{PCy2, C1}$), 39.9 (d, $^{1}J_{CP}$ = 15.0 Hz, *C*H, $_{PCy2, C1}$) ppm.

^{31}P{^{1}H} NMR (162 MHz, CD_2Cl_2) δ = 37.1 (*P*Cy$_3$), 48.6 (*P*Cy$_2$) ppm.

CHNS Calculated: C: 56.46, H: 8.53. Measured: C: 56.67, H: 8.72.

IR (ATR) ṽ = 2929 (vs), 2847 (s), 1444 (s), 885 (m), 849 (vs), 838 (vs), 569 (w), 465 (m) cm^{-1}.

m.p. 217.3 °C (decomposition).

1.3.2 Synthesis of **L2·PdCl$_2$**

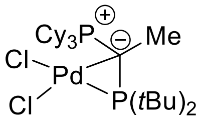

trYPhos (L2) (2.00 g, 4.42 mmol, 1.0 eq.) and [(COD)PdCl$_2$] (1.26 g, 4.42 mmol, 1.0 eq.) were suspended in THF (25 ml), whereby the reaction mixture became dark yellow. After stirring overnight, the precipitate was allowed to settle before removing the solution with a filter cannula. The remaining solid was washed twice with THF (25 ml) and dried *in vacuo*. The product was obtained as a yellow solid (2.24 g, 3.56 mmol, 80%).

^{1}H NMR (400 MHz, CD_2Cl_2) δ = 1.22 – 1.45 (m, 9H, C*H*$_2$, $_{Cy, H3 + H4}$), 1.61 (d, $^{3}J_{HP}$ = 16.7 Hz, 9H, C*H*$_3$, $_{tBu}$), 1.74 (d, $^{3}J_{HP}$ = 15.7 Hz, 9H, C*H*$_3$, $_{tBu}$), 1.66 – 1.91 (m, 6H, C*H*$_2$, $_{Cy, H2 + H4}$), 1.90 – 2.01 (m, 6H, C*H*2, $_{Cy, H3}$), 2.06 (dd, $^{3}J_{HP}$ = 15.0 Hz, $^{3}J_{HP}$ = 12.8 Hz, 3H, C*H*$_3$, $_{Me}$), 2.31 – 2.54 (m, 6H, C*H*$_2$, $_{Cy, H1 + H2}$), 2.87 (s, 3H, C*H*$_2$, $_{Cy, H2}$).

^{13}C{^{1}H} NMR (101 MHz, CD_2Cl_2): δ = 20.0 (dd, $^{1}J_{CP}$ = 30.2 Hz, $^{1}J_{CP}$ = 12.7 Hz, P–*C*$^{-}$–P), 24.9 (s, *C*H$_3$, $_{Me}$), 25.6 (s, *C*H$_2$, $_{PCy3, C4}$), 27.5 (d, $^{2}J_{CP}$ = 11.6 Hz, *C*H$_2$, $_{PCy3, C2}$), 27.6 (d, $^{2}J_{CP}$ = 11.3 Hz, *C*H$_2$, $_{PCy3, C2}$), 29.2 (s, *C*H$_2$, $_{PCy3, C3}$), 30.3 (d, $^{3}J_{CP}$ = 3.4 Hz, *C*H$_2$, $_{PCy3, C3}$), 31.4 (d, $^{2}J_{CP}$ = 4.2 Hz, *C*H$_3$, $_{tBu}$), 33.2 (d, $^{2}J_{CP}$ = 2.4 Hz, *C*H$_3$, $_{tBu}$), 39.1 – 40.2 (m, *C*H, $_{PCy3, C1}$), 40.1 (d, $^{1}J_{CP}$ = 2.9 Hz, *C*, $_{tBu}$), 41.3 (d, $^{1}J_{CP}$ = 13.1 Hz, *C*, $_{tBu}$) ppm.

^{31}P{^{1}H} NMR (162 MHz, CD_2Cl_2): δ = 38.7 (d, $^{2}J_{PP}$ = 4.7 Hz, *P*Cy2), 83.2 (d, $^{2}J_{PP}$ = 4.7 Hz, *P*Cy3), ppm.

CHNS Calculated: C: 53.33, H: 8.57. Measured: C: 53.24, H: 8.73.

IR (ATR) ṽ = 2933 (s), 2850 (m), 1489 (w), 1447 (m), 1396 (m), 1370 (m), 1325 (w), 1296 (w), 1172 (s), 1124 (w), 1004 (m), 918 (w), 893 (m), 849 (m), 814 (vs), 802 (vs), 747 (w), 619 (m), 596 (w), 541 (m), 508 (m) cm^{-1}.

m.p. 161.0 °C (decomposition).

1.3.3 Synthesis of **L3·PdCl$_2$**

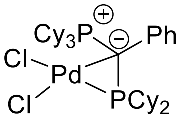

joYPhos (L3) (2.00 g, 3.53 mmol, 1.0 eq.) and [(COD)PdCl$_2$] (1.01 g, 3.53 mmol, 1.0 eq.) were suspended in THF (30 ml). The orange suspension was stirred overnight. The crude product was filtered through a *Schlenk* frit and washed with two additional portions of THF (15 ml). The product was dried *in vacuo* and obtained as a light-yellow powder (1.24 g, 3.52 mmol, 99%).

^{1}H NMR (400 MHz, CD$_2$Cl$_2$) δ = 0.60 – 2.88 (m, 55H, C*H*, $_{PCy3 + PCy2}$ + C*H*$_2$, $_{PCy3 + PCy2}$), 7.12 (d, $^3J_{HH}$ = 7.9 Hz, 1H, C*H*, $_{ortho}$), 7.22 (t, $^3J_{HH}$ = 7.5 Hz, 1H, C*H*, $_{meta}$), 7.32 (t, $^3J_{HH}$ = 7.4 Hz, 1H, C*H*, $_{para}$), 7.40 (t, $^3J_{HH}$ = 7.7 Hz, 1H, C*H*, $_{meta}$), 8.91 (d, $^3J_{HH}$ = 7.7 Hz, 1H, C*H*, $_{ortho}$) ppm.

^{13}C{^{1}H} NMR (101 MHz, CD$_2$Cl$_2$): δ = 26.1 (*C*H$_2$, $_{PCy2, C4 + PCy3, C4}$), 27.3 (d, $^3J_{CP}$ = 11.6 Hz, *C*H$_2$, $_{PCy3, C3}$), 27.5 (d, $^3J_{CP}$ = 12.1 Hz, *C*H$_2$, $_{PCy2, C3}$), 27.87 (*C*H$_2$, $_{PCy2, C3}$), 27.90 (d, $^3J_{CP}$ = 23.6 Hz, *C*H$_2$, $_{PCy2, C3}$), 28.4 (d, $^2J_{CP}$ = 16.5 Hz, *C*H$_2$, $_{PCy2, C3}$), 30.7 (*C*H$_2$, $_{PCy2, C2}$), 32.0 (d, $^2J_{CP}$ = 8.0 Hz, *C*H$_2$, $_{PCy2, C2}$), 32.2 – 32.0 (m, *C*H$_2$, $_{PCy2, C2}$), 39.7 (d, $^1J_{CP}$ = 21.8 Hz, *C*H, $_{PCy2, C1}$), 40.8 (d, $^1J_{CP}$ = 13.0 Hz, *C*H, $_{PCy2, C1}$), 127.5 (*C*H, $_{Ph,\ meta}$), 128.8 (*C*H, $_{Ph,\ para}$), 130.1 (s, *C*H, $_{Ph,\ meta}$), 132.2 (*C*H, $_{Ph,\ ortho}$), 133.8 (*C*H, $_{Ph,\ ipso}$), 141.1 (t, $^3J_{CP}$ = 5.2 Hz, *C*H, $_{Ph,\ ortho}$).
^{31}P{^{1}H} NMR (162 MHz, CD$_2$Cl$_2$): δ = 35.3 (d, $^2J_{PP}$ = 4.0 Hz, *P*Cy$_3$), 53.7 (d, $^2J_{PP}$ = 4.0 Hz, *P*Cy$_2$) ppm.
CHNS Calculated: C: 59.72, H: 8.13. Measured: C: 60.08, H: 8.10.
IR (ATR) ṽ = 2918 (vs), 2851 (s), 1446 (s), 1174 (m), 1000 (s), 950 (m), 918 (m), 892 (w), 857 8m), 851 (m), 707 (s), 538 (s), 529 (s), 512 (m) cm^{-1}.
m.p. 224.4 °C (decomposition).

1.3.4 Synthesis of **L3·PdBr$_2$**

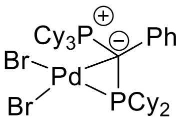

joYPhos (L3) (500 mg, 0.88 mmol, 1.1 eq.) and (COD)PdBr$_2$ (300 mg, 0.80 mmol, 1.0 eq.) was suspended in THF (25 ml). The orange suspension was stirred overnight. A yellow solid dropped out from the solution and was filtered through a *Schlenk* frit. The solid was washed two times with 10 ml of THF and dried *in vacuo*. The product was obtained as yellow powder (550 mg, 0.66 mmol, 82%).

^{1}H NMR (400 MHz, CD$_2$Cl$_2$) δ = 0.56 – 3.16 (m, 55H, C*H*, $_{PCy_3 + PCy_2}$ + C*H*$_2$, $_{PCy_3 + PCy_2}$), 7.15 (d, $^3J_{HH}$ = 7.9 Hz, 1H, C*H*, $_{ortho}$), 7.23 (t, $^3J_{HH}$ = 7.5 Hz, 1H, C*H*, $_{meta}$), 7.33 (t, $^3J_{HH}$ = 7.4 Hz, 1H, C*H*, $_{para}$), 7.40 (t, $^3J_{HH}$ = 7.7 Hz, 1H, C*H*, $_{meta}$), 8.96 (d, $^3J_{HH}$ = 7.7 Hz, 1H, C*H*, $_{ortho}$) ppm.
^{13}C{^{1}H} NMR (101 MHz, CD$_2$Cl$_2$): δ = 25.8 – 26.1 (m, *C*H$_2$, $_{PCy_2, C4 + PCy_3, C4}$), 27.1 – 27.4 (m, *C*H$_2$, $_{PCy_3, C3}$), 27.6 (d, $^3J_{CP}$ = 12.3 Hz, *C*H$_2$, $_{PCy_2, C3}$), 27.9 (d, $^3J_{CP}$ = 4.9 Hz, *C*H$_2$, $_{PCy_2, C3}$), 28.0 (*C*H$_2$, $_{PCy_2, C3}$), 28.3 (d, $^2J_{CP}$ = 16.4 Hz, *C*H$_{2,PCy_2,C3}$), 30.9 (*C*H$_2$, $_{PCy_2, C2}$), 32.08

(d, $^2J_{CP}$ = 13.6 Hz, CH$_{2,PCy_2,C2}$), 32.09 (CH$_{2,PCy_2,C2}$), 32.4 (d, $^2J_{CP}$ = 2.9 Hz, CH$_{2,\ PCy_2,\ C2}$), 40.0 (d, $^1J_{CP}$ = 21.7 Hz, CH$_{\ PCy_2,\ C1}$), 41.3 (d, $^1J_{CP}$ = 13.0 Hz, CH$_{PCy_2,C1}$), 127.6 (t, $^4J_{CP}$ = 2.1 Hz, CH$_{\ Ph,\ meta}$), 128.9 (t, $^5J_{CP}$ = 2.5 Hz, CH$_{\ Ph,\ para}$), 130.2 (CH$_{\ Ph,\ meta}$), 132.5 (t, $^3J_{CP}$ = 3.8 Hz, CH$_{Ph,ortho}$), 133.5 (CH$_{,Ph,ipso}$), 141.7 (t, $^3J_{CP}$ = 5.1 Hz, CH$_{,Ph,\ ortho}$).
^{31}P{^{1}H} NMR (162 MHz, CD_2Cl_2): δ = 35.2 (d, $^2J_{PP}$ = 3.9 Hz, PCy$_3$), 59.2 (d, $^2J_{PP}$ = 3.9 Hz, PCy$_2$) ppm.
CHNS Calculated: C: 53.35, H: 7.26. Measured: C: 53.32, H: 7.32.
IR (ATR) ṽ = 2930 (s), 2845 (m), 1440 (m), 1062 (w), 1005 (m), 971 (m), 918 (w), 884 (w), 854 (w), 725 (w), 704 (s), 540 (w), 524 (s), 514 (vs) cm^{-1}.
m.p. 231.2 °C

1.3.5 Synthesis of L4·PdCl2

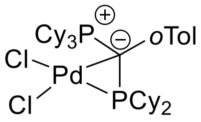

pinkYPhos (L4) (1.00 g, 1.72 mmol, 1.05 eq.) and [(COD)$PdCl_2$] (0.47 g, 1.64 mmol, 1.0 eq.) were dissolved in THF (20 ml) to obtain an orange solution which was stirred overnight. The resulting yellow suspension was separated with a *Schlenk* frit and washed two times with THF (20 ml). The solid was dried *in vacuo*. The product was obtained as a yellow powder (1.15 g, 1.52 mmol, 93%).

^{1}H NMR (400 MHz, CD_2Cl_2) δ = 0.46 – 2.41 (m, 50H, CH + CH_2, $_{PCy3\ +\ PCy2}$), 2.48 (s, 3H, CH_3), 2.55 – 3.87 (m, 5H, CH + CH_2, $_{PCy3\ +\ PCy2}$ + C$H_{3,\ isomer}$), 7.00 – 7.14 (m, 1H), 7.14 – 7.32 (m, 2H), 8.62 – 9.27 (m, 1H, CH, $_{Ar,\ ortho}$) ppm.
^{31}P{^{1}H} NMR (162 MHz, CD_2Cl_2): δ = 38.2 (d, $^2J_{PP}$ = 5.7 Hz, PCy$_2$), 57.4 (PCy$_3$) (isomer: 40.0 (PCy$_2$), 51.8 (PCy$_3$)).
No clear allocation of the ^{13}C{^{1}H} NMR data to the molecular structure can be stated.
CHNS Calculated: C: 60.03, H: 8.20. Measured: C: 60.04, H: 8.27.
IR (ATR) ṽ = 2932 (s), 2917 (vs), 2853 (m), 1445 (m), 1174 (w), 1005 (w), 964 (w), 933 (w), 893 (w), 865 (w), 854 (w), 773 (w), 756 (w), 732 (w), 550 (w), 530 8w), 516 (w) cm^{-1}.
m.p. 194.8 °C (decomposition)

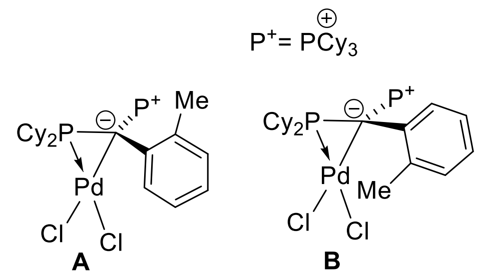

L4·PdCl2 forms two stable rotamers in solution which differ in the orientation of the tolyl group relative to the P–C–P plane: in the major conformer **A** characterized by the signals at 38.2 and 57.4 ppm in the ^{31}P NMR spectrum, the methyl group points away from the $PdCl_2$ fragment, resulting in a short Pd-H distance and a down field shift of the corresponding proton signal in the ^{1}H NMR spectrum (Δ_δ = 1.7 ppm). In the minor conformer (δ = 40.0 and 51.8 ppm), the methyl group points towards the $PdCl_2$ moiety, thus giving rise to a down-field shift of the signal of the methyl group (Δ_δ = 0.8 ppm).

VT-NMR studies in the temperature range between –30 and 60 °C did not lead to the coalescence of the signals, accounting for an appreciable barrier between both rotamers.

1.4 Synthesis of LH·PdCl$_3$ Complexes

1.4.1 Synthesis of **L1H·PdCl$_3$**

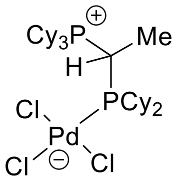

L1·PdCl$_2$ (1.00 g, 1.46 mmol, 1.0 eq.) was suspended in DCM (10 ml), and a large excess of HCl (about 0.5 ml, 37% in water) was added to the solution. The dark yellow solution was stirred overnight. The solvent was removed *in vacuo,* and the residue was washed with THF (2 x 15 ml). The product was dried and isolated as an orange powder (0.81 g, 1.13 mmol, 77%).

^{1}H NMR (400 MHz, CD_2Cl_2) δ = 1.07 – 1.16 (m, 1H, C*H*$_2$, $_{PCy2,\ H3}$), 1.21 – 1.26 (m, 1H, C*H*$_2$, $_{PCy2,\ H3}$), 1.27 – 1.42 (m, 8H, C*H*$_2$, $_{PCy3,\ H3\ +\ H4\ +\ PCy2,\ H4}$), 1.46 – 2.00 (m, 35H, C*H*$_2$, $_{PCy3,\ H2\ +\ H3\ +\ H4\ +\ PCy2,\ H2\ +H3\ +H4}$ + C*H*$_3$), 2.16 – 2.24 (m, 3H, C*H*$_2$, $_{PCy3,\ H2}$), 2.26 – 2.34 (m, 3H, C*H*$_2$, $_{PCy3,\ H2}$), 2.33 – 2.55 (m, 1H, C*H*, $_{PCy2,\ H1}$), 2.60 – 2.82 (m, 1H, C*H*$_2$, $_{PCy2,\ H2}$), 2.86 – 3.02 (m, 1H, C*H*, $_{PCy2,\ H1}$), 3.02 – 3.11 (m, 1H, C*H*$_2$, $_{PCy2,\ H2}$), 3.10 – 3.21 (m, 1H, P–C*H*–P), 3.54 – 4.05 (m, 3H, C*H*, $_{PCy3,\ H1}$) ppm.
^{13}C{^{1}H} NMR (101 MHz, CD_2Cl_2): δ = 11.6 (*C*H$_3$), 20.7 (d, $^1J_{CP}$ = 36.8 Hz, P-*C*H-P), 26.2 (d, $^4J_{CP}$ = 1.7 Hz, *C*H$_2$, $_{PCy3,\ C4}$), 26.9 (*C*H$_2$, $_{PCy2,\ C4}$), 27.1 (d, $^3J_{CP}$ = 11.4 Hz, *C*H$_2$, $_{PCy3,\ C3}$), 27.6 (d, $^3J_{CP}$ = 11.9 Hz, *C*H$_2$, $_{PCy3,\ C3}$), 27.8 (d, $^3J_{CP}$ = 8.0 Hz, *C*H$_2$, $_{PCy2,\ C3}$), 28.0 (d, $^3J_{CP}$ = 15.3 Hz, *C*H$_2$, $_{PCy2,\ C3}$), 28.3 (d, $^3J_{CP}$ = 8.3 Hz, *C*H$_2$, $_{PCy2,\ C3}$), 28.9 (d, $^3J_{CP}$ = 15.7 Hz, *C*H$_2$, $_{PCy2,\ C3}$), 29.7 (*C*H$_2$, $_{PCy3,\ C2}$), 30.3 (*C*H$_2$, $_{PCy3,\ C2}$), 30.5 (d, $^2J_{CP}$ = 8.3 Hz, *C*H$_2$, $_{PCy2,\ C2}$), 31.8 (d, $^2J_{CP}$ = 8.0 Hz, *C*H$_2$, $_{PCy2,\ C2}$), 32.3 (d, $^2J_{CP}$ = 2.7 Hz, *C*H$_2$, $_{PCy2,\ C2}$), 33.7 (d, $^1J_{CP}$ = 33.7 Hz, *C*H, $_{PCy3,\ C1}$), 34.0 (d, $^2J_{CP}$ = 5.5 Hz, *C*H$_2$, $_{PCy2,\ C2}$), 37.2 (d, $^1J_{CP}$ = 19.2 Hz, *C*H, $_{PCy2,\ C1}$), 38.6 (dd, $^1J_{CP}$ = 20.0 Hz, $^3J_{CP}$ = 3.7 Hz, *C*H, $_{PCy2,\ C1}$) ppm.
CHNS Calculated: C: 53.49, H: 8.28. Measured: C: 53.24, H: 8.29.
^{31}P{^{1}H} NMR (162 MHz, CD_2Cl_2): δ = 30.5 (*P*Cy$_2$), 40.4 (d, $^2J_{PP}$ = 4.7 Hz, *P*Cy$_3$) ppm.
IR (ATR) ṽ = 2932 (vs), 2854 (s), 1446 (s), 1296 (w), 1267 (w), 1177 (m), 1003 (s), 921 (w), 896 (m), 850 (s), 758 (m), 729 (vs), 698 (s), 638 (m), 620 (s), 553 (w), 539 (w), 522 (m) cm^{-1}.
m.p. 241.6 °C (decomposition).

1.4.2 Synthesis of **L2H·PdCl3**

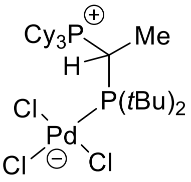

L2·PdCl2 (1.00 g, 1.59 mmol, 1.0 eq.) was suspended in 10 ml of DCM, and a large excess of HCl (about 0.5 ml, 37% in water) was added to the solution. The brown solution was stirred overnight, and the solvent was removed at reduced pressure. The residue was washed two times with 15 ml of THF, and the solid was dried *in vacuo*. The product was obtained as a dark yellow solid (0.79 g, 1.19 mmol, 75%).

^{1}H NMR (400 MHz, CD_2Cl_2) δ = 1.09 – 1.47 (m, 3H, C*H*2, Cy, H4), 1.56 – 1.85 (m, 27H, C*H*2, Cy, H2 + H3 + H4 + C*H*3, *t*Bu + Me), 1.89 (d, 9H, $^{3}J_{HP}$ = 14.6 Hz, C*H*3, *t*Bu), 1.91 – 2.14 (m, 6H, C*H*2, Cy, H3), 2.22 – 2.41 (m, 3H, C*H*2, Cy, H2), 2.46 – 3.02 (m, 3H, C*H*2, Cy, H2), 2.99 – 4.07 (vbr, 3H, C*H*, Cy, H1), 4.94 (ddq, 1H, $^{2}J_{HP}$ = 23.0 Hz, $^{2}J_{HP}$ = 15.3, $^{3}J_{HH}$ = 7.6 Hz, P–C*H*–P) ppm.
^{13}C{^{1}H} NMR (101 MHz, CD_2Cl_2): δ = 16.8 (dd, $^{2}J_{CP}$ = 6.2 Hz, $^{2}J_{CP}$ = 2.7 Hz, *C*H3, Me), 26.0 (d, $^{4}J_{CP}$ = 1.8 Hz, *C*H2, Cy, C4), 27.5 (d, $^{3}J_{CP}$ = 11.4 Hz, *C*H2, Cy, C3), 27.7 (d, $^{3}J_{CP}$ = 11.8 Hz, *C*H2, Cy, C3), 27.9 (dd, $^{1}J_{CP}$ = 25.8 Hz, $^{1}J_{CP}$ = 7.5 Hz, P-*C*H-P), 29.4 – 30.3 (m, *C*H2, Cy, C2), 31.0 – 32.3 (m, *C*H3, *t*Bu), 33.4 (d, $^{2}J_{CP}$ = 3.9 Hz, *C*H3, *t*Bu), 35.8 (d, $^{1}J_{CP}$ = 33.0 Hz, *C*H, Cy, C1), 41.6 (d, $^{1}J_{CP}$ = 7.8 Hz, *C*, *t*Bu), 44.5 (dd, $^{1}J_{CP}$ = 9.8 Hz, $^{3}J_{CP}$ = 5.7 Hz, *C*, *t*Bu) ppm.
^{31}P{^{1}H} NMR (162 MHz, CD_2Cl_2): δ = 43.1 (d, $^{2}J_{PP}$ = 10.5 Hz, *P*Cy3), 66.9 (d, $^{2}J_{PP}$ = 10.5 Hz, *Pt*Bu2) ppm.
CHNS Calculated: C: 50.46, H: 8.32. Measured: C: 50.04, H: 7.97.
IR (ATR) ṽ = 2980 (s), 2971 (s), 2924 (vs), 2853 (m), 1446 (s), 1404 (w), 1392 (m), 1367 (w), 1232 (w), 1171 (m), 1021 (w), 1001 (w), 934 (w), 888 (w), 850 (w), 742 (s), 702 (w), 610 (m), 536 (s), 509 (w) cm^{-1}.
m.p. 204.5 °C (decomposition).

1.4.3 Synthesis of **L3H·PdCl3**

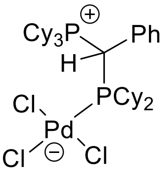

Synthesis from L3·PdCl2

L3·PdCl2 (0.20 g, 0.27 mmol, 1.0 eq.) was suspended in DCM (10 ml) and concentrated HCl_{aq} (0.10 ml, 37% in water, 1.4 mmol, 5.0 eq.) was added. The mixture was stirred overnight, and an orange solution was formed. The solvent was removed *in vacuo,* and THF (20 ml) was added. The mixture was stirred for one hour until a yellow solid precipitated from the solution. The yellow powder was filtered through a frit and washed with two additional portions of THF (10 ml), and dried *in vacuo*. The product was obtained as a dark yellow powder (130 mg, 0.17 mmol, 62%).

Synthesis from L3H

L3H (1.0 g, 1.66 mmol, 1.0 eq.) and $(CH_3CN)_2PdCl_2$ (0.38 g, 1.66 mmol, 1.0 eq.) was dissolved in 10 ml of DCM and stirred overnight. The solvent was removed *in vacuo,*

and the solid was washed two times with 10 ml of THF. The solid was obtained as a dark yellow powder (0.89 g, 1.14 mmol, 69%).

^{1}H NMR (400 MHz, CD_2Cl_2) δ = –0.12 – 0.24 (m, 1H, C*H*$_2$, $_{\text{PCy2, H3}}$), 0.96 – 2.01 (m, 45H, C*H*$_2$, $_{\text{PCy3 + PCy2}}$), 2.03 – 2.32 (m, 7H, C*H*$_2$, $_{\text{PCy3, H2 + PCy2, H2}}$), 2.33 – 2.52 (m, 1H, C*H*, $_{\text{PCy2, H1}}$), 2.63 – 2.75 (m, 1H, C*H*, $_{\text{PCy2, H1}}$), 2.75 – 2.84 (m, 1H, C*H*$_2$, $_{\text{PCy2, H2}}$), 3.06 – 3.17 (m, 1H, C*H*$_2$, $_{\text{PCy2, H2}}$), 3.21 – 4.67 (m, 2H, C*H*, $_{\text{PCy3, H1}}$), 4.39 (dd, 1H, $^1J_{CP}$ = 15.6 Hz, $^3J_{CP}$ = 12.3 Hz, P–C*H*–P), 7.22 (d, 1H, $^4J_{CP}$ = 7.7 Hz, C*H*, $_{\text{ortho}}$), 7.37 (t, 1H, $^5J_{CP}$ = 7.6 Hz, C*H*, $_{\text{meta}}$), 7.45 (t, 1H, $^6J_{CP}$ = 7.5 Hz, C*H*, $_{\text{para}}$), 7.52 (t, 1H, $^5J_{CP}$ = 7.6 Hz, C*H*, $_{\text{meta}}$), 8.55 (d, 1H, $^4J_{CP}$ = 7.9 Hz, C*H*, $_{\text{ortho}}$) ppm.

^{13}C{^{1}H} NMR (101 MHz, CD_2Cl_2): δ = 26.3 (d, *J*=1.8 Hz, *C*H$_2$, $_{\text{PCy3, C4}}$), 26.6 – 27.1 (m, *C*H$_2$, $_{\text{PCy2, C4 + PCy3, C3}}$), 27.3 (d, $^3J_{CP}$ = 11.8 Hz, *C*H$_2$, $_{\text{PCy3, C3}}$), 28.1 (d, $^3J_{CP}$ = 15.6 Hz, *C*H$_2$, $_{\text{PCy2, C3}}$), 28.26 (d, $^3J_{CP}$ = 2.3 Hz, *C*H$_2$, $_{\text{PCy2, C3}}$), 28.34 (d, $^3J_{CP}$ = 1.9 Hz, *C*H$_2$, $_{\text{PCy2, C3}}$), 28.9 (d, $^3J_{CP}$ = 16.2 Hz, *C*H$_2$, $_{\text{PCy2, C3}}$), 30.5 (d, $^2J_{CP}$ = 8.7 Hz, *C*H$_2$, $_{\text{PCy2, C2}}$), 30.8 (d, $^2J_{CP}$ = 4.9 Hz, *C*H$_2$, $_{\text{PCy3, C2}}$), 31.4 (*C*H$_2$, $_{\text{PCy3, C2}}$), 32.0 (d, $^2J_{CP}$ = 8.7 Hz, *C*H$_2$, $_{\text{PCy2, C2}}$), 32.8 (d, $^2J_{CP}$ = 3.8 Hz, *C*H$_2$, $_{\text{PCy2, C2}}$), 34.3 (d, $^1J_{CP}$ = 6.2 Hz, *C*H$_2$, $_{\text{PCy2, C2}}$), 34.4 – 35.1 (m, *C*H, $_{\text{PCy3, C1}}$ + P–*C*H–P), 38.4 (d, $^1J_{CP}$ = 16.5 Hz, *C*H, $_{\text{PCy2, C1}}$), 41.1 (dd, $^1J_{CP}$ = 17.7 Hz, $^3J_{CP}$ = 2.3 Hz, *C*H, $_{\text{PCy2, C1}}$), 127.4 (dd, $^2J_{CP}$ = 5.5 Hz, $^4J_{CP}$ = 3.8 Hz, *C*H, $_{\text{Ph, }\textit{ipso}}$), 129.1 (*C*H, $_{\text{Ph, }\textit{meta}}$), 129.5 (d, $^4J_{CP}$ = 2.5 Hz, *C*H, $_{\text{Ph, }\textit{meta}}$), 130.2 (t, $^5J_{CP}$ = 1.9 Hz, *C*H, $_{\text{Ph, }\textit{para}}$), 132.3 (dd, $^3J_{CP}$ = 6.7 Hz, $^5J_{CP}$ = 4.1 Hz, *C*H, $_{\text{Ph, }\textit{ortho}}$), 134.0 (d, $^3J_{CP}$ = 2.7 Hz, *C*H, $_{\text{Ph, }\textit{ortho}}$) ppm.

^{31}P{^{1}H} NMR (162 MHz, CD_2Cl_2): δ = 31.4 (d, $^2J_{PP}$ = 14.5 Hz, *P*Cy$_2$), 38.5 (d, $^2J_{PP}$ = 14.5 Hz, *P*Cy$_3$) ppm.

CHNS Calculated: C: 56.93, H: 7.88. Measured: C: 57.27, H: 7.88.

IR (ATR) ṽ = 2924 (vs), 2851 (vs), 1493 (w), 1452 (s), 1323 (w), 1291 (m), 1274 (m), 1234 (w), 1175 (m), 1130 (w), 1080 (w), 1041 (w), 1003 (vs), 949 (w), 920 (w), 894 (m), 849 (s), 821 (w), 803 (w), 737 (w), 724 (vs), 712 (vs), 696 (m), 670 (m), 615 (w), 597 (m), 525 (s), 514 (vs) cm^{-1}.

m.p. 222.8 °C (decomposition).

1.4.4 Synthesis of **L4H·PdCl$_3$**

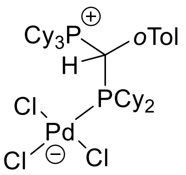

Synthesis from L4·PdCl$_2$

L4·PdCl$_2$ (1.0 g, 1.34 mmol, 1.0 eq.) was dissolved in 20 ml of DCM, and 0.5 ml (37% in water, excess) of HCl was added to the mixture. The dark red solution was stirred overnight. The solvent was removed at reduced pressure, and the residue was solidified in 10 ml of diethyl ether. The solid was filtered and washed with two portions of THF (10 ml). The product was obtained as an orange solid (0.84 g, 1.08 mmol, 80%).

Synthesis from L4H

L4H (1.0 g, 1.66 mmol, 1.0 eq.) and (COD)$PdCl_2$ (0.47 g, 1.66 mmol, 1.0 eq.) were dissolved in 10 ml of DCM. The red solution was stirred overnight, and the solvent was removed *in vacuo*. The residue was suspended in 10 ml of THF, and the solid was filtrated and washed with additional 10 ml of THF. The solid was dried *in vacuo,* and the product was obtained as orange solid (1.2 g, 1.51 mmol, 91%).

^{1}H NMR (400 MHz, CD_2Cl_2) δ = -0.09 – 0.11 (m, 1H, CH_2, $_{PCy2, H3}$), 0.77 – 0.91 (m, 1H, CH_2, $_{PCy2, H2}$), 0.91 – 1.81 (m, 39H, CH_2, $_{PCy3, H2 + H3 + H4 + PCy2, H2 +H3 +H4}$), 1.83 – 2.00 (m, 1H, C$H_2$, $_{PCy2, H2}$), 2.02 – 2.16 (m, 3H, CH_2, $_{PCy3, H2}$), 2.16 – 2.27 (m, 3H, CH_2, $_{PCy3, H2}$), 2.27 – 2.33 (m, 1H, C*H*, $_{PCy2, H1}$), 2.35 (s, 3H, CH_3), 2.64 – 2.81 (m, 2H, C*H*, $_{PCy2, H1}$ + CH_2, $_{PCy2, H2}$), 2.93 – 3.11 (m, 1H, CH_2, $_{PCy2, H2}$), 3.35 – 4.18 (vbr, 3H, C*H*, $_{PCy3, H1}$), 4.50 (dd, 1H, $^2J_{CP}$ = 15.3 Hz, $^2J_{CP}$ = 12.3 Hz, P–C*H*–P), 7.06 – 7.15 (m, 1H, C*H*, $_{arom, meta}$), 7.19 – 7.38 (m, 2H, C*H*, $_{arom, meta' + para}$), 8.12 – 8.26 (m, 1H, C*H*, $_{arom,ortho'}$) ppm.
^{13}C{^{1}H} NMR (101 MHz, CD_2Cl_2): δ = 21.6 (*C*H_3), 26.2 (d, $^4J_{CP}$ = 1.8 Hz, *C*H_2, $_{PCy3, C4}$), 26.5 (d, $^4J_{CP}$ = 1.8 Hz, *C*H_2, $_{PCy2, C4}$), 26.7 (d, $^4J_{CP}$ = 1.8 Hz, *C*H_2, $_{PCy2, C4}$), 27.0 (d, $^3J_{CP}$ = 11.5 Hz, *C*H_2, $_{PCy3, C3}$), 27.5 (d, $^3J_{CP}$ = 11.7 Hz, *C*H_2, $_{PCy3, C3}$), 28.2 (d, $^3J_{CP}$ = 7.2 Hz, *C*H_2, $_{PCy2, C3}$), 28.3 (d, $^3J_{CP}$ = 14.8 Hz, *C*H_2, $_{PCy2, C3}$), 28.4 (d, $^3J_{CP}$ = 8.0 Hz, *C*H_2, $_{PCy2, C3}$), 28.9 (d, $^3J_{CP}$ = 16.0 Hz, *C*H_2, $_{PCy2, C3}$), 30.7 (d, $^2J_{CP}$ = 4.7 Hz, *C*H_2, $_{PCy3, C2}$), 30.9 – 31.2 (m, *C*H_2, $_{PCy3, C2 + PCy2, C2}$), 31.3 (d, $^2J_{CP}$ = 9.1 Hz, *C*H_2, $_{PCy2, C2}$), 33.0 (dd, $^2J_{CP}$ = 35.5 Hz, $^2J_{CP}$ = 7.9 Hz, P–CH–P), 33.2 (d, $^2J_{CP}$ = 4.6 Hz, *C*H_2, $_{PCy2, C2}$), 34.6 (d, $^2J_{C P}$ = 5.9 Hz, *C*H_2, $_{PCy2, C2}$), 35.9 (d, $^1J_{CP}$ = 31.4 Hz, *C*H, $_{PCy3, C1}$), 39.4 (d, $^1J_{CP}$ = 16.3 Hz, *C*H, $_{PCy2, C1}$), 41.1 (dd, $^1J_{CP}$ = 16.5, $^3J_{CP}$ = 2.0 Hz, *C*H, $_{PCy2, C1}$), 126.6 – 126.9 (m, *C*, $_{arom, ortho}$), 126.9 (*C*H, $_{arom, meta'}$), 130.1 (d, $^5J_{CP}$ = 2.4 Hz, *C*H, $_{arom, para}$), 132.6 (d, $^4J_{CP}$ = 1.7 Hz, *C*H, $_{arom, meta}$), 134.5 – 136.0 (m, *C*H, $_{arom, ortho'}$), 136.6 (dd, $^2J_{CP}$ = 6.4 Hz, $^2J_{CP}$ = 3.6 Hz, *C*, $_{arom, ipso}$) ppm.
^{31}P{^{1}H} NMR (162 MHz, CD_2Cl_2): δ = 36.1 – 38.0 (br, *P*Cy_2), 42.5 (d, $^2J_{PP}$ = 16.7 Hz, *P*Cy_3) ppm.
CHNS Calculated: C: 57.44, H: 7.99. Measured: C: 57.80, H: 7.59.
IR (ATR) ṽ = 2980 (w), 2928 (vs), 2853 (s), 1445 (s), 1287 (w), 1268 (w), 1170 (w), 1128 (w), 1062 (w), 1003 (m), 893 (w), 848 (s), 789 (m), 749 (w), 730 (vs), 697 (w), 670 (w), 538 (w), 523 (w) cm^{-1}.
m.p. 192.8 °C (decomposition).

1.5 Procedures of the Catalytic Reactions

1.5.1 Procedure of the Buchwald-Hartwig amination

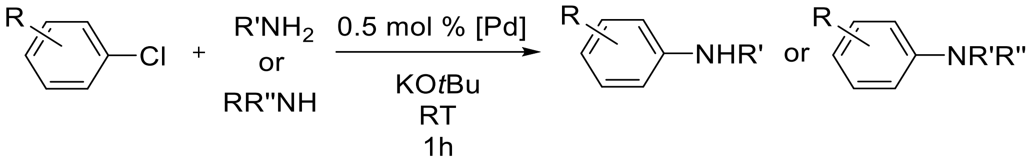

In the glovebox, the precatalyst (0.005 mmol, 0.005 eq.) [For L·Pd(dba): ligand (0.005 mmol, 0.005 eq.) and Pd_2dba_3·dba (0.005 mmol, 0.005 eq.)] and potassium *tert*-butoxide (1.5 mmol, 1.5 eq.) were loaded in a 6 ml vial, and the vial was closed with a septum cap. Outside of the glovebox, a second vial, which was purged with argon, was loaded with 1.0 mmol (1.0 eq.) of a haloaryl compound, 1.1 mmol (1.1 eq.) of a primary or secondary amine, and the GC-standard tetradecane (1.0 mmol, 1.0 eq.) and dry THF was filled up to 3 ml. Dry THF (1 ml) was added to the first vial containing the catalyst. After 5 minutes [For L·Pd(dba) after 30 minutes], the catalyst solution was added to the reagent solution. The reaction was stirred for one hour at room temperature if not stated otherwise.

After one hour, the solution was quenched with 2 ml of a saturated NaCl solution, and one aliquot of the organic phase was washed through a silica-filled pipette with ethyl acetate. A GC-FID spectrum of the resulting solution was measured, and the conversion was determined by integration and calculation with the response factor.

For isolation, the aqueous phase was extracted three times with 10 ml of ethyl acetate. The organic phases were combined, and the solvent was removed *in vacuo*. The crude product was purified *via* column chromatography (4 g silica-packed weld column, 0-30% EtOAc in hexane).

Comparison to Pd-PEPPSI-IPent

To compare the reactivity of our **L·PdCl2** complexes with similar complexes reported in literature, we performed the coupling of 4-chlorotoluene with piperidine under the same reaction conditions as described above as well as at 60 °C with 0.5 mol% of the commercially available Pd-PEPPSI-IPent complex. The following results were obtained.

Temperature	Yield (after 1h)	Yield (after 3h)
RT	36	40
60 °C	51	64

1.5.2 Procedure of the Kinetic Studies of the Buchwald-Hartwig Amination

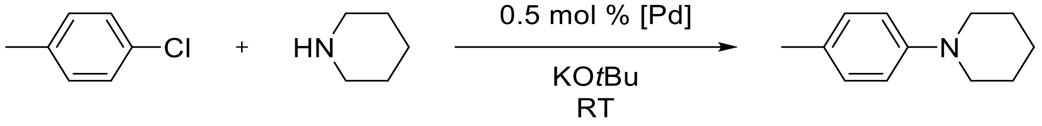

In the glovebox, the precatalyst (0.025 mmol, 0.005 eq.) [For L·Pd(dba): ligand (0.025 mmol, 0.005 eq.) and Pd_2dba_3·dba (0.025 mmol, 0.005 eq.)] and potassium *tert*-butoxide (7.5 mmol, 1.5 eq.) were loaded into a 6 ml vial with a septum cap, and the vial was closed with a septum cap. Outside of the glovebox, a *Schlenk* flask, which was purged with argon, was loaded with 0.59 ml (0.63 g, 5.0 mmol, 1.0 eq.) of 4-chlorotoluene, 0,54 ml (0.47 g, 5.5 mmol, 1.1 eq.) of piperidine, and the GC-standard tetradecane (1,30 ml, 1.0 g, 5.0 mmol, 1.0 eq.) and dry THF was filled up to 15 ml. Dry THF (5 ml) was added to the first vial containing the catalyst. After 5 minutes [For L·Pd(dba) after 30 minutes], the catalyst solution was added to the reagent solution. The reaction was stirred at room temperature. Small aliquots of the reaction solution were quenched after the appropriate amount of time with aqueous saturated NaCl solution and the organic phase was filtered through a silica-filled pipette with ethylacetate. A GC-FID spectrum of the resulting solution was measured, and the conversion was determined by integration and calculation with the response factor. Detailed results are given in Figure S6 and Figure S7.

1.5.3 Procedure of the α-Arylation of Ketones

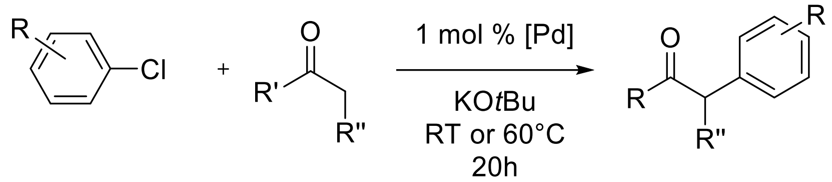

Two 6 ml vials were put in the glovebox, and the first one was filled with the precatalyst (0.01 mmol, 0.01 eq.) [For L·Pd(dba): ligand (0.01 mmol, 0.01 eq.) and Pd_2dba_3·dba (0.01 mmol, 0.01 eq.)] and the second one was filled with potassium *tert*-butoxide (1.5 mmol, 1.5 eq.) and both vials were sealed with a septum cap and removed from the glovebox. The base was dissolved in 4 ml of dry THF, and the corresponding ketone (1.1 mmol, 1.1 eq.) was added. The solution was stirred for 30 minutes at room temperature, and the haloarene (1.0 mmol, 1.0 eq.) and the GC standard tetradecane (1.0 mmol, 1.0 eq.) were added to the solution. The catalyst was suspended in 1 ml of THF and stirred for 5 minutes [For L·Pd(dba) for 30 minutes]. The reagent solution was added to the catalyst solution, and the mixture was stirred overnight at room temperature or 60 °C.

The solution was quenched with 2 ml of a saturated NaCl solution, and one aliquot of the organic phase was washed through a silica-filled pipette with ethyl acetate. A GC-

FID spectrum was measured of the resulting solution, and the conversion was determined by integration and calculation with the response factor.

For isolation, the aqueous phase was extracted three times with 10 ml of ethyl acetate. The organic phases were combined, and the solvent was removed *in vacuo*. The crude product was purified *via* column chromatography (4 g silica-packed weld column, 0-30% EtOAc in hexane).

1.5.4 Procedure of the Kumada Coupling

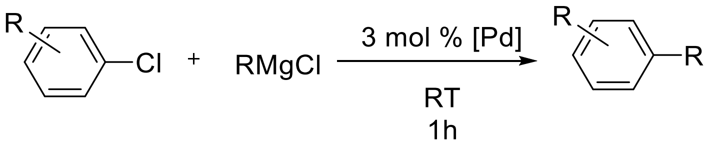

A 6 ml vial was filled in the glovebox with the precatalyst (0.03 mmol, 0.03 eq.) [For L·Pd(dba): ligand (0.03 mmol, 0.03 eq.) and Pd_2dba_3·dba (0.03 mmol, 0.03 eq.)] and closed with a septum cap. The vial was removed from the glovebox, and the haloarene (1.0 mmol, 1.0 eq.) and tetradecane (1.0 mmol, 1.0 eq.) were added into the vial and diluted in 1 ml of dry toluene. [For L·Pd(dba): The mixture was stirred for one hour.] The Grignard species (1.2 mmol) was filled to 3.3 ml with dry toluene (0.36 M) and added dropwise within one hour with a syringe pump to the reaction solution.

The black suspension was stirred for 10 more minutes and quenched with 1 ml of a saturated NaCl solution, and one aliquot of the organic phase was filtrated through a silica-filled pipette with ethyl acetate. A GC-FID spectrum was measured of the resulting solution, and the conversion was determined by integration and calculation with the response factor.

For isolation, the aqueous phase was extracted three times with 10 ml of ethyl acetate. The organic phases were combined, and the solvent was removed *in vacuo*. The crude product was washed through a silica-packed column with hexane. The solvent was removed under reduced pressure. Further purification steps are described in each procedure.

1.5.5 Procedure of the Murahashi Coupling

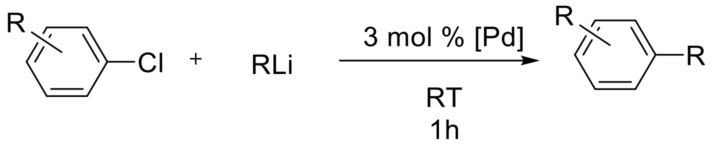

A 6 ml vial was filled in the glovebox with the precatalyst (0.03 mmol, 0.03 eq.) [For L·Pd(dba): ligand (0.03 mmol, 0.03 eq.) and Pd_2dba_3·dba (0.03 mmol, 0.03 eq.)] and closed with a septum cap. The vial was removed from the glovebox, and the haloarene (1.0 mmol, 1.0 eq.) and tetradecane (1.0 mmol, 1.0 eq.) were added into the vial and diluted in 1 ml of dry toluene. [For L·Pd(dba): The mixture was stirred for one hour.] The alkyllithium species (1.2 mmol) was filled to 3.3 ml with dry toluene (0.36 M) and added dropwise within one hour with a syringe pump to the reaction solution.

The black suspension was stirred for 10 more minutes and quenched with 1 ml of a saturated NaCl solution, and one aliquot of the organic phase was filtrated through a silica-filled pipette with ethyl acetate. A GC-FID spectrum was measured of the resulting solution, and the conversion was determined by integration and calculation with the response factor.

For isolation, the aqueous phase was extracted three times with 10 ml of ethyl acetate. The organic phases were combined, and the solvent was removed *in vacuo*. The crude product was washed through a silica-packed column with hexane. The solvent was removed under reduced pressure. Further purification steps are described in each procedure.

1.6 Isolated Cross-Coupling Products

1.6.1 Isolation of **Aa**

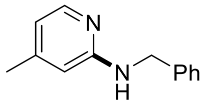

96 mg (48%) of a colorless solid was isolated.

^{1}H NMR ($CDCl_3$, 400 MHz): δ = 2.07 (s, 3H, CH_3), 4.36 (d, $^3J_{HH}$ = 5.8 Hz, 2H, -CH_2-), 4.97 – 5.17 (br, 1H, N*H*), 6.07 (d, $^4J_{HH}$ = 1.5, 1H, CH_{Pyr}), 6.30 (dd, $^3J_{HH}$ = 5.2 Hz, $^4J_{HH}$ = 1.5 Hz, 1H, CH_{Pyr}), 7.08 – 7.17 (m, 1H, CH_{Ph}), 7.17 – 7.28 (m, 4H, CH_{Ph}), 7.81 (d, $^3J_{HH}$ = 5.2 Hz, 1H, CH_{Pyr}) ppm.

MS (EI): m/z (%) = 198.1 (100 [M+]), 182.1, 170.1, 168.1, 154.1.

Spectral data obtained for the compound are in good agreement with the reported data.[6]

1.6.2 Isolation of **Ab**

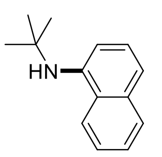

200 mg (100%) of a dark orange oil was isolated.

^{1}H NMR ($CDCl_3$, 400 MHz): δ = 1.52 (s, 9H, CH_3), 4.14 – 4.40 (br, 1H, N*H*), 7.00 (d, $^3J_{HH}$ = 7.5 Hz, 1H, C$H_{,Ar}$), 7.31 (d, $^3J_{HH}$ = 8.1 Hz, 1H, C$H_{,Ar}$), 7.39 (t, $^3J_{HH}$ = 7.9 Hz, 1H, C$H_{,Ar}$), 7.44 – 7.55 (m, 2H, C$H_{,Ar}$), 7.66 – 8.00 (m, 2H, C$H_{,Ar}$) ppm.

MS (EI): m/z (%) = 199.1 (100 [M+]), 184.1, 168.1, 154.1, 143.1.

Spectral data obtained for the compound are in good agreement with the reported data.[7]

1.6.3 Isolation of **Ac**

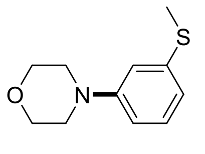

196 mg (94%) of a light-yellow oil was isolated.

^{1}H NMR ($CDCl_3$, 400 MHz): δ = 2.48 (s, 3H, CH_3), 3.12 – 3.16 (m, 4H, -CH_2-), 3.82 – 3.87 (m, 4H, -CH_2-), 6.70 (dd, $^3J_{HH}$ = 8.2 Hz, $^4J_{HH}$ = 2.5 Hz, 1H, C$H_{,Ar}$), 6.78 (d, $^3J_{HH}$ = 8.2 Hz, $^4J_{HH}$ = 1.8 Hz, 1H, C$H_{,Ar}$), 6.80 – 6.84 (m, 1H, C$H_{,Ar}$), 7.17 – 7.23 (m, 1H, C$H_{,Ar}$) ppm.

MS (EI): m/z (%) = 209.1 (100 [M+]), 207.0, 194.1, 178.0, 164.1, 151.0.

Spectral data obtained for the compound are in good agreement with the reported data.[8]

1.6.4 Isolation of **Ad**

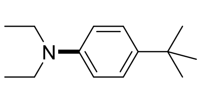

206 mg (100%) of a light-yellow solid was isolated.

^{1}H NMR ($CDCl_3$, 400 MHz): δ = 1.15 (t, $^3J_{HH}$ = 7.0 Hz, 6H, C$H_{3,Et}$), 1.29 (s, 9H, C$H_{3,tBu}$), 3.33 (q, $^3J_{HH}$ = 7.0 Hz, 4H, C$H_{2,Et}$), 6.65 (d, $^3J_{HH}$ = 8.8 Hz, 2H), 7.33 (d, $^3J_{HH}$ = 8.8 Hz, 2H) ppm.

MS (EI): m/z (%) = 205.2 (100 [M+]), 190.2, 188.1.

Spectral data obtained for the compound are in good agreement with the reported data.[9]

1.6.5 Isolation of **Ae**

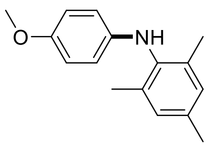

The reaction was performed at 60 °C. 236 mg (98%) of a light red solid was isolated.

^{1}H NMR ($CDCl_3$, 400 MHz): δ = 2.17 (s, 6H, C$H_{3,Mes,ortho}$), 2.31 (s, 3H, C$H_{3,Mes,para}$), 3.75 (s, 3H, C$H_{3,OMe}$), 4.90 – 4.98 (br, 1H, N*H*), 6.48 (d, $^3J_{HH}$ = 8.9 Hz, 2H, C$H_{,Ar}$), 6.75 (d, $^3J_{HH}$ = 8.9 Hz, 2H, C$H_{,Ar}$), 6.94 (s, 2H, C$H_{,Mes}$) ppm.

MS (EI): m/z (%) = 241.1 (100 [M+]), 226.1, 208.1.

Spectral data obtained for the compound are in good agreement with the reported data.[10]

1.6.6 Isolation of **Af**

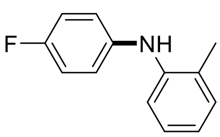

The reaction was performed at 60 °C. 199 mg (99%) of a light-yellow oil was isolated.

^{1}H NMR ($CDCl_3$, 400 MHz): δ = 2.17 (s, 3H, C*H*$_3$), 5.13 – 5.22 (br, 1H, N*H*), 6.70 – 6.94 (m, 5H, C*H*$_{, Ar}$), 6.95 – 7.08 (m, 2H, C*H*$_{, Ar}$), 7.08 – 7.20 (m, 1H, C*H*$_{, Ar}$) ppm.
^{19}F {^{1}H} NMR ($CDCl_3$, 235 MHz): δ = –122.6 ppm.
MS (EI): m/z (%) = 201.1 (100 [M+]), 185.0, 180.1.
Spectral data obtained for the compound are in good agreement with the reported data.[11]

1.6.7 Isolation of **Ba**

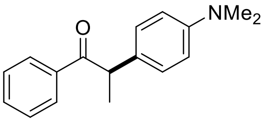

253 mg (99%) of a colorless solid was isolated.

^{1}H NMR ($CDCl_3$, 400 MHz): δ = 1.52 (d, $^3J_{HH}$ = 6.8 Hz, 3H, C*H*$_3$), 2.90 (s, 6H, NMe_2), 4.62 (q, $^3J_{HH}$ = 6.8 Hz, 1H, C*H*), 6.67 – 6.69 (m, 2H, C*H*$_{arom}$), 7.14 – 7.19 (m, 2H, C*H*$_{arom}$), 7.35 – 7.40 (m, 2H, C*H*$_{arom}$), 7.43 – 7.48 (m, 1H, C*H*$_{arom}$), 7.96 – 8.01 (m, 2H, C*H*$_{arom}$) ppm.
MS (EI): m/z (%) = 253.1 (100 [M+]), 148.1, 132.1.
Spectral data obtained for the compound are in good agreement with the reported data.[12]

1.6.8 Isolation of **Bb**

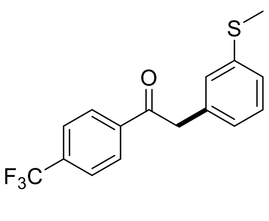

288 mg (93%) of a yellow solid was isolated.

^{1}H NMR ($CDCl_3$, 400 MHz): δ = 2.43 (s, 3H, C*H*$_3$), 4.24 (s, 2H, C*H*$_2$), 6.97 – 7.01 (m, 1H, C*H*$_{arom}$), 7.10 – 7.14 (m, 2H, C*H*$_{arom}$), 7.20 – 7.25 (m, 1H, C*H*$_{arom}$), 7.66 – 7.71i (m, 2H, C*H*$_{arom}$), 8.03 – 8.09 (m, 2H, C*H*$_{arom}$) ppm.
^{13}C{^{1}H} NMR ($CDCl_3$, 101 MHz) δ = 15.7 (S*C*H$_3$), 45.7 (-*C*H$_2$-), 123.6 ($^1J_{CF}$ = 273 Hz, *C*F$_3$), 125.2 (*C*H$_{ArSMe}$), 125.8 (q, $^3J_{CF}$ = 3.8 Hz, *C*H$_{ArCF3}$), 126.2 (*C*H$_{ArSMe}$), 127.4 (*C*H$_{ArSMe}$), 129.0 (*C*H$_{ArCF3}$), 129.3 (*C*H$_{ArSMe}$), 134.53 (q, $^2J_{CF}$ = 32.7 Hz, *C*$_{ArCF3}$), 134.55 (*C*$_{arom}$) 139.2 (*C*$_{arom}$), 139.3 (*C*$_{arom}$), 196.4 (*C*=O) ppm.
^{19}F{^{1}H} NMR ($CDCl_3$, 235 MHz) δ = –63.1 ppm.
HRMS (m/z): [M-H]+ calcd for $C_{16}H_{13}F_3OS$, 310.0639; found, 310.0645.

IR (ATR) ṽ = 2932 (w), 2898 (w), 1684 (s), 1593 (w), 1573 (w), 1511 (w), 1478 (w), 1442 (w), 1409 (m), 1320 (s), 1209 (w), 1161 (m), 1124 (s), 1110 (vs), 1085 (w), 1064

(vs), 1016 (w), 1001 (m), 991 (m), 906 (w), 892 (w), 873 (w), 851 (w), 836 (m), 783 (w), 758 (m), 738 (w), 723 (w), 702 (m), 688 (m), 604 (m), 574 (w), 557 (w) cm^{-1}.
m.p. 64.7 – 67.3°C

1.6.9 Isolation of **Bc**

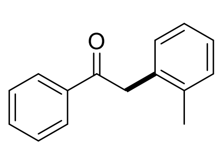

195 mg (93%) of a light red solid was isolated.
^{1}H NMR ($CDCl_3$, 400 MHz): δ = 2.23 (s, 3H, C*H*3), 4.27 (s, 2H, C*H*2), 7.07 – 7.20 (m, 4H, C*H*arom), 7.38 – 7.48 (m, 2H, C*H*arom), 7.50 – 7.56 (m, 1H, C*H*arom), 7.97 – 8.02 (m, 2H, C*H*arom) ppm.
MS (EI): m/z (%) = 210.2 (100 [M+]), 192.1, 179.1, 165.0, 105.0.
Spectral data obtained for the compound are in good agreement with the reported data.[13]

1.6.10 Isolation of **Bd**

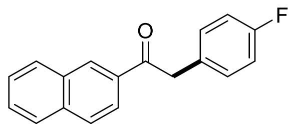

The reaction was performed at 60 °C. 232 mg (88%) of a colorless solid was isolated.
^{1}H NMR ($CDCl_3$, 400 MHz): δ = 4.39 (s, 2H, C*H*2), 6.98 – 7.06 (m, 2H, C*H*arom), 7.23 – 7.29 (m, 2H, C*H*arom), 7.52 – 7.63 (m, 2H, C*H*arom), 7.84 – 7.91 (m, 2H, C*H*arom), 7.93 – 7.98 (m, 1H, C*H*arom), 8.00 – 8.06 (m, 1H, C*H*arom), 8.51 – 8.54 (m, 1H, C*H*arom) ppm.
^{13}C{^{1}H} NMR ($CDCl_3$, 101 MHz) δ = 44.7 (*C*H2), 115.6 (*C*Harom, Napht), 115.8 (*C*Harom, Napht), 124.3 (*C*Harom, Napht), 127.0 (*C*Harom, Napht), 127.9 (*C*Harom, Napht), 128.8 (d, $^3J_{CF}$ = 3.9 Hz, *C*Harom, ArF), 129.7 (*C*Harom, Napht), 130.40 (d, $^4J_{CF}$ = 3.5 Hz, *C*arom, ArF), 130.43 (*C*Harom, Napht), 131.2 (d, $^2J_{CF}$ = 8.0 Hz, *C*Harom, ArF), 132.6 (*C*arom, Napht), 133.9 (*C*arom, Napht), 135.8 (*C*arom, Napht), 162.0 (d, $^1J_{CF}$ = 245 Hz, *C*-F) , 197.5 (*C*=O) ppm.
^{19}F{^{1}H} NMR ($CDCl_3$, 235 MHz) δ = –28.2 ppm.
HRMS (m/z): [M-H]+ calcd for $C_{18}H_{13}FO$, 264.0950; found, 264.0959.
IR (ATR) ṽ = 1685 (w), 1676 (s), 1611 (w), 1598 (w), 1511 (vs), 1466 (w), 1356 (w), 1326 (w), 1224 (vs), 1183 (w), 1169 (w), 1157 (w), 1121 (w), 1092 (w), 1028 (w), 1015 (w), 993 (w), 940 (w), 864 (s), 820 (vs), 789 (vs), 745 (vs), 698 (w), 648 (w), 524 (vs), 513 (m), 502 (w) cm^{-1}.
m.p. 115.9 – 116.6°C

1.6.11 Isolation of **Be**

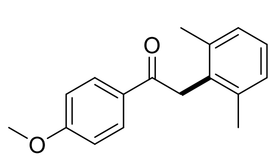

The reaction was performed at 60 °C. 160 mg (63%) of a colorless solid was isolated.
^{1}H NMR ($CDCl_3$, 400 MHz): δ = 2.25 (s, 6H, CH_3), 3.90 (s, 3H, OC*H*3), 4.35 (s, 2H, C*H*2), 6.97 – 7.02 (m, 2H, C*H*arom), 7.06 – 7.15 (m, 3H, C*H*arom), 8.07 – 8.12 (m, 2H, C*H*arom) ppm.
MS (EI): m/z (%) = 254.1 (100 [M+]), 135.0.

Spectral data obtained for the compound are in good agreement with the reported data.[14]

1.6.12 Isolation of **Bf**

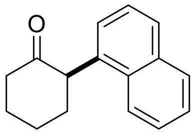

The reaction was performed at 60 °C. 140 mg (62%) of a colorless solid was isolated.

^{1}H NMR ($CDCl_3$, 400 MHz): δ = 1.84 – 2.02 (m, 2H, CH_2), 2.04 – 2.20 (m, 1H, CH_2), 2.20 – 2.36 (m, 2H, CH_2), 2.40 – 2.50 (m, 1H, CH_2), 2.63 – 2.78 (m, 2H, CH_2), 4.37 (dd, $^3J_{HH}$ = 12.6 Hz, $^3J_{HH}$ = 5.3 Hz, 1H, C*H*), 7.31 – 7.41 (m, 1H, CH_{arom}), 7.42 – 7.55 (m, 3H, CH_{arom}), 7.62 – 7.76 (m, 1H, CH_{arom}), 7.76 – 7.83 (m, 1H, CH_{arom}), 7.83 – 7.92 (m, 1H, CH_{arom}) ppm.
MS (EI): m/z (%) = 224.1 (100 [M+]), 180.1, 167.1, 153.1, 141.1.
Spectral data obtained for the compound are in good agreement with the reported data.[15]

1.6.13 Isolation of **Ca**

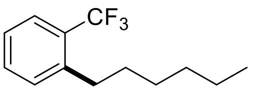

The reaction was performed with *n*-hexyllithium (in hexane). No further purification steps were performed. The product was isolated as a colorless oil (143 mg, 0.62 mmol, 62%).

^{1}H NMR ($CDCl_3$, 400 MHz): δ = 0.82 – 0.91 (m, 3H, $CH_{3, Hex}$), 1.26 – 1.33 (m, 4H, $CH_{2, Hex}$), 1.34 – 1.43 (m, 2H, $CH_{2, Hex}$), 1.43 – 1.71 (m, 2H, $CH_{2, Hex}$), 2.62 – 2.89 (m, 2H, $CH_{2, Hex}$), 7.22 (t, $^3J_{HH}$ = 7.8 Hz, 1H, CH_{arom}), 7.29 (d, $^3J_{HH}$ = 7.6 Hz, 1H, CH_{arom}), 7.41 (t, $^3J_{HH}$ = 7.6 Hz, 1H, CH_{arom}), 7.57 (dd, $^3J_{HH}$ = 7.8 Hz, $^4J_{HH}$ = 1.4 Hz, 1H, CH_{arom}) ppm.
^{19}F{^{1}H} NMR ($CDCl_3$, 235 MHz) δ = 57.7 ppm.
MS (EI): m/z (%) = 230.1 (100 [M+]), 172.0, 160.0, 133.0.
Spectral data obtained for the compound are in good agreement with the reported data.[16]

1.6.14 Isolation of **Cb**

The reaction was performed with phenyllithium (in di-*n*-butylether). The crude product was purified *via* column chromatography (0-10% EtOAc in hexane). The product was isolated as colorless solid (127 mg, 0.64 mmol, 64%).

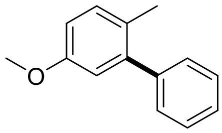

^{1}H NMR ($CDCl_3$, 400 MHz): δ = 2.20 (s, 3H, CH_3), 3.81 (s, 3H, OCH_3), 6.78 – 6.82 (m, 1H, CH_{Ar}), 6.82 – 6.86 (m, 1H, CH_{arom}), 7.18 (d, $^3J_{HH}$ = 8.3 Hz, 1H, CH_{arom}), 7.29 – 7.36 (m, 3H, CH_{arom}), 7.38 – 7.46 (m, 2H, CH_{arom}) ppm.
^{13}C {^{1}H} NMR ($CDCl_3$, 101 MHz): δ = 19.6 (CH_3), 55.5 (OCH_3), 113.1 (CH_{arom}), 115.3 (CH_{arom}), 127.0 (CH_{arom}), 127.5 (C_{arom}), 128.2 (CH_{arom}), 129.2 (CH_{arom}), 131.3 (CH_{arom}), 142.1 (C_{arom}), 143.0 (C_{arom}), 157.7 (C_{arom}) ppm.

IR (ATR) ṽ = 2981 (m), 2831 (w), 1606 (m), 1570 (w), 1505 (m), 1484 (vs), 1463 (m), 1442 (m), 1402 (w), 1381 (w), 1319 (w), 1295 (s), 1264 (m), 1220 (s), 1210 (s), 1175 (s), 1140 (w), 1125 (w), 1073 (w), 1040 (vs), 1020 (m), 991 (w), 956 (w), 877 (w), 856 (w), 805 (w), 774 (m), 743 (m), 702 (vs), 628 (w), 611 (w), 548 (w), 506 (w) cm^{-1}.
HRMS (m/z): [M-H]+ calcd for $C_{14}H_{14}O$, 198.1045; found, 198.1048.

1.6.15 Isolation of **Cc**

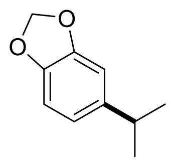

The reaction was perfomed with *iso*-propyllithium (in pentane). The product was purified by *Kugelrohr* distillation (120°C, 30 mbar). The product was obtained as colorless oil (98 mg, 0.60 mmol, 60%; br:ln = 95:5).

^{1}H NMR ($CDCl_3$, 400 MHz): δ = 1.21 (d, $^3J_{HH}$ = 6.9 Hz, 6H, CH_3), 2.84 (hept, $^3J_{HH}$ = 6.9 Hz, 1H, C*H*), 5.91 (s, 2H, CH_2), 6.54 – 6.71 (m, 1H, CH_{arom}), 6.70 – 6.90 (m, 2H, CH_{arom}) ppm.
MS (EI): m/z (%) = 164.1 (100 [M+]), 149.0, 119.0, 91.0.
Spectral data obtained for the compound are in good agreement with the reported data.[17]

1.6.16 Isolation of **Cd**

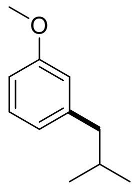

The reaction was performed with *tert*-butyllithium (in pentane). The crude product was purified *via* column chromatography (0-10% EtOAc in hexane). The product was obtained as colorless oil (88 mg 0.54 mmol, 54%; br:ln = 0:100).

^{1}H NMR ($CDCl_3$, 400 MHz): δ = 0.91 (d, $^3J_{HH}$ = 6.7 Hz, 6H, CH_3), 1.71 – 2.02 (m, 1H, C*H*), 2.45 (d, $^3J_{HH}$ = 7.2 Hz, 2H, CH_2), 3.80 (s, 3H, OCH_3), 6.60 – 6.71 (m, 1H, CH_{arom}), 6.71 – 6.86 (m, 2H, CH_{arom}), 7.01 – 7.23 (m, 1H, CH_{arom}) ppm.
MS (EI): m/z (%) = 164.1 (100 [M+]), 149.1, 122.1, 121.1, 107.1, 91.1.
Spectral data obtained for the compound are in good agreement with the reported data.[16]

1.6.17 Isolation of **Da**

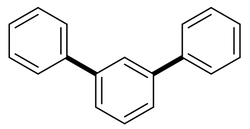

The reaction was performed with 3 equiv. of phenylmagnesium chloride (in THF). The crude product was purified by sublimation ($5\cdot10^{-2}$ mbar, 100 °C). 180 mg (0.78 mmol, 78 %) of a colorless solid was isolated.

^{1}H NMR ($CDCl_3$, 400 MHz): δ = 7.18 – 7.28 (m, 2H, CH_{arom}), 7.29 – 7.6 (m, 4H, CH_{arom}), 7.36 – 7.41 (m, 1H, CH_{arom}), 7.42 – 7.49 (m, 2H, CH_{arom}), 7.49 – 7.60 (m, 4H, CH_{arom}), 7.65 – 7.82 (m, 1H, CH_{arom}) ppm.
MS (EI): m/z (%) = 230.1 (100 [M+]), 215.1, 202.1, 152.1, 115.0.

Spectral data obtained for the compound are in good agreement with the reported data.[18]

1.6.18 Isolation of **Db**

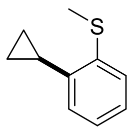

The reaction was performed with cyclopropylmagnesium bromide (in methyl-THF). The product was purified by *Kugelrohr* distillation (90°C, 20 mbar) and obtained as colorless oil.

^{1}H NMR ($CDCl_3$, 400 MHz): δ = 0.51 – 0.79 (m, 2H, $CH_{2,\ cyclopentyl}$), 0.87 – 1.09 (m, 2H, $CH_{2,\ cyclopentyl}$), 2.05 (tt, $^3J_{HH}$ = 8.4 Hz, $^3J_{HH}$ = 5.4 Hz, 1H, $CH_{,\ cyclopentyl}$), 2.48 (s, 3H, SCH_3), 6.95 – 7.02 (m, 1H, CH_{arom}), 7.04 – 7.14 (m, 1H, CH_{arom}), 7.16 – 7.25 (m, 2H, CH_{arom}) ppm.
^{13}C {^{1}H} NMR ($CDCl_3$, 101 MHz): δ = 7.5 ($CH_{2,\ cyclopentyl}$), 13.8 ($CH_{,\ cyclopentyl}$ or SCH_3), 15.5 ($CH_{,\ cyclopentyl}$ or SCH_3), 124.7 (CH_{arom}), 124.8 (CH_{arom}), 125.7 (CH_{arom}), 126.4 (CH_{arom}), 139.3 (C_{arom}), 140.6 (C_{arom}) ppm.
IR (ATR) ṽ = 3081 (w), 3057 (w), 3002 (w), 2918 (w), 1588 (w), 1472 (m), 1454 (w), 1436 (s), 1267 (w), 1123 (w), 1051 (m), 1032 (w), 967 (w), 888 (w), 821 (w), 746 (vs), 671 (w) cm^{-1}.
HRMS (m/z): $[M-H]^+$ calcd for $C_{10}H_{12}S$, 164.0666; found, 164.0664.

1.6.19 Isolation of **Dc**

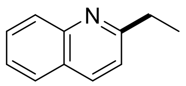

The reaction was performed with ethylmagnesium bromide (in diethylether). The crude product was purified *via* column chromatography (0-30% EtOAc in hexane). A yellow oil (122 mg, 0.78 mmol, 78%) was obtained.

^{1}H NMR ($CDCl_3$, 400 MHz): δ = 1.39 (t, $^3J_{HH}$ = 7.6 Hz, 3H, CH_3), 3.00 (q, $^3J_{HH}$ = 7.6 Hz, 2H, CH_2), 7.29 (d, $^3J_{HH}$ = 8.4 Hz, 1H, CH_{arom}), 7.35 – 7.51 (m, 1H, CH_{arom}), 7.54 – 7.72 (m, 1H, CH_{arom}), 7.72 – 7.88 (m, 1H, CH_{arom}), 8.04 (d, $^3J_{HH}$ = 8.4 Hz, 2H, CH_{arom}) ppm.
MS (EI): m/z (%) = 157.1 (100 [M+]), 156.1, 130.1, 129.1, 128.1, 102.1, 77.1.
Spectral data obtained for the compound are in good agreement with the reported data.[19]

1.6.20 Isolation of **Dd**

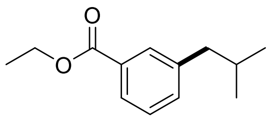

The reaction was performed with *tert*-butylmagnesium chloride (in THF). The crude product was purified by *Kugelrohr* distillation (130°C, 10 mbar, br:ln = 0:100). The product was obtained as colorless oil (144 mg, 0.70 mmol, 70%).

^{1}H NMR ($CDCl_3$, 400 MHz): δ = 0.91 (d, $^3J_{HH}$ = 6.6 Hz, 6H, $CH_{3,\ iPr}$), 1.40 (t, $^3J_{HH}$ = 7.1 Hz, 3H, $CH_{3,\ OEt}$), 1.82 – 2.04 (m, 1H, $CH_{,\ iPr}$), 2.53 (d, $^3J_{HH}$ = 7.2 Hz, 2H, CH_2), 4.37 (q, $^3J_{HH}$ = 7.1 Hz, 2H, $CH_{2,\ OEt}$), 7.29 – 7.38 (m, 2H, CH_{arom}), 7.81 – 7.84 (m, 1H, CH_{arom}), 7.85 – 7.92 (m, 1H, CH_{arom}) ppm.

MS (EI): m/z (%) = 206.1 (100 [M+]), 164.1, 163.1, 136.0, 135.0, 119.0, 91.0.
Spectral data obtained for the compound are in good agreement with the reported data.[20]

Further compounds synthesized but not isolated.

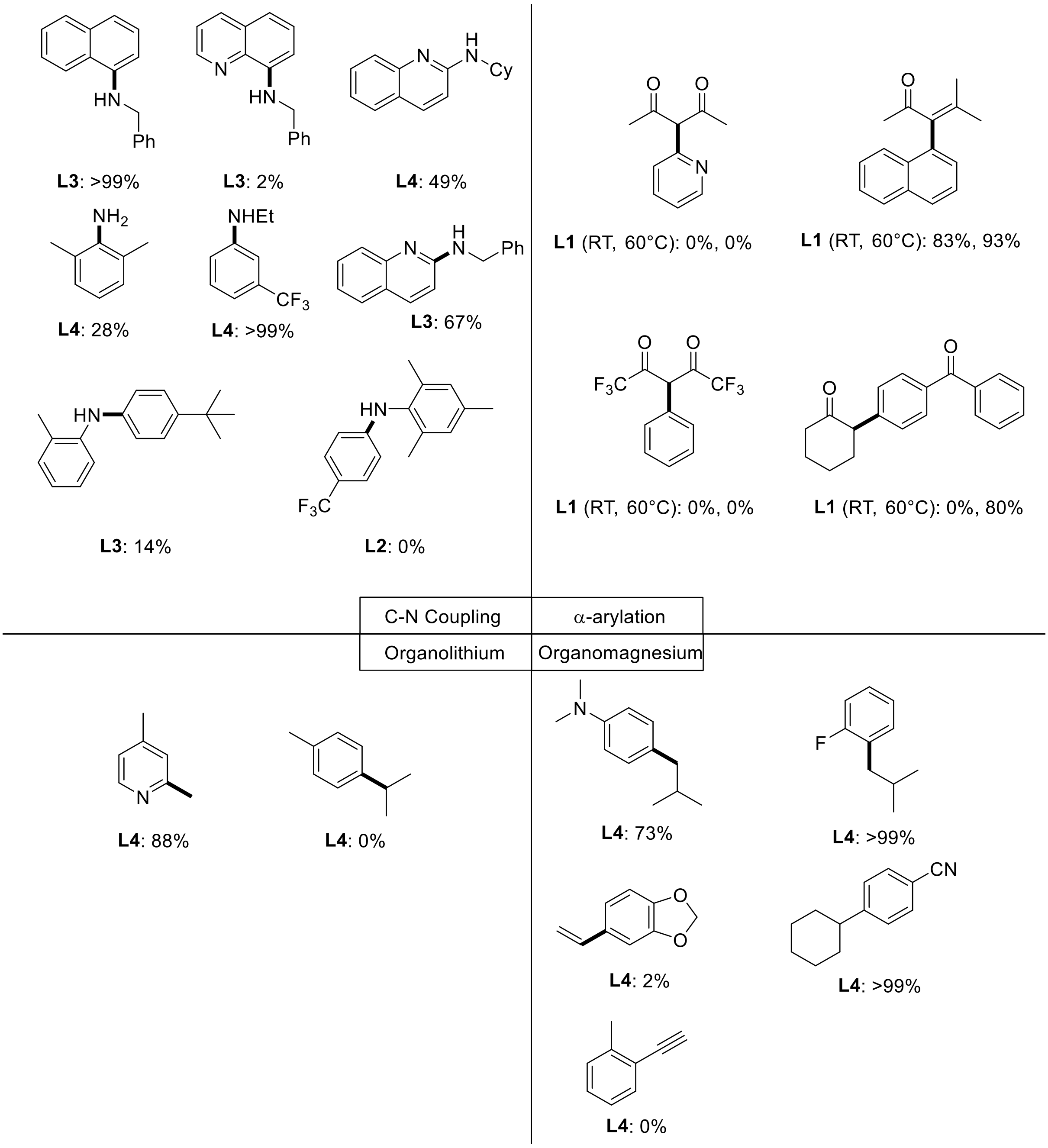

Figure S1. Compounds synthesized according to the catalytic procedures given above (similar to Figure 7), but haven't been isolated due to low conversion or difficulties in the purification.

2. Comments and Additional Information

2.1 Catalyst Activation

To obtain insights into the activation of **L·PdCl₂**, the corresponding complex with keYPhos (**L1**) was reacted in THF with two equiv. KOtBu. Addition of the base instantaneously resulted in the dissolution of the $PdCl_2$ complex and the darkening of the reaction mixture. $^{31}P\{^{1}H\}$ NMR spectroscopy indicates the initial formation of a new species, which shows two doublets at 51.2 and 77.5 ppm with a coupling constant of 98 Hz (**Figure S2**). This species reacts further to form palladium black along with the palladium(0) bisphosphine complex $(\mathbf{L1})_2$·Pd (**Figure S2**),[1] which is characterized by two signals at 18.9 and 30.3 ppm and remains the main product after a longer reaction time. This observation clearly indicates that L·$PdCl_2$ is readily reduced to Pd(0) species by the metal base. No further substrates, which might give rise to undesired byproducts, are necessary for the activation. It is interesting to note that GC analysis of the reaction solution showed no identifiable organic by-product. We assume that initially *tert*-butoxide for chloride exchange occurs at palladium. The thus formed complex is highly electron rich and may form a cationic $[LPdOtBu]^+$ species in solution which readily abstracts a proton e.g. from the THF solvent to form the palladium(0) species LPd after reductive elimination of *tert*-butanol. Unfortunately, we were not able to detect *tert*-butanol or any other organic compound (e.g. isobutanal) in the reaction mixture after filtration via silica. Also, no phosphine oxide formation was observed. The oxide of the keYPhos ligand shows signals at 36 and 1 ppm in the ^{31}P NMR spectrum. No signals in this region were observed when treating LPdCl2 with KOtBu.

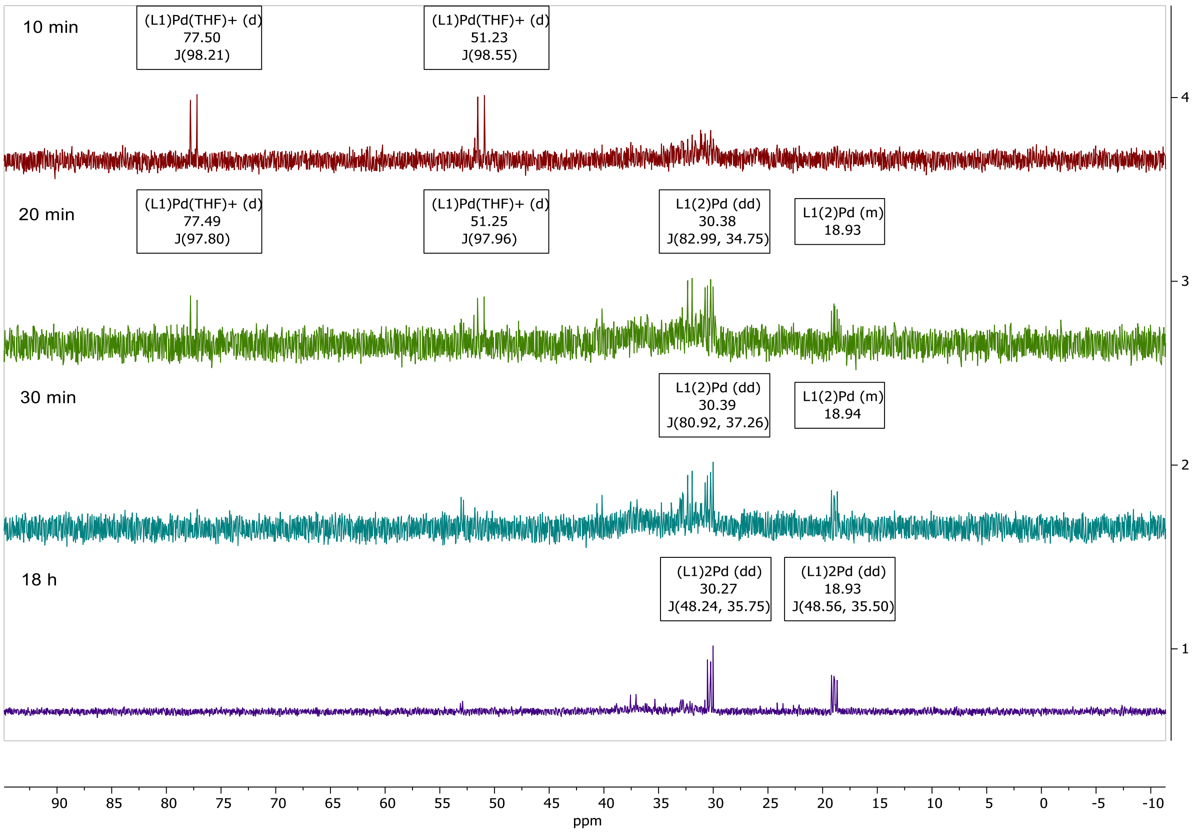

Figure S2. $^{31}P\{^{1}H\}$ NMR spectra at different reaction times of the reaction of **L1·PdCl₂** with two eq. of KO*t*Bu.

2.2 Van-der-Waals plots of L1H·$PdCl_3$ and L4H·$PdCl_3$

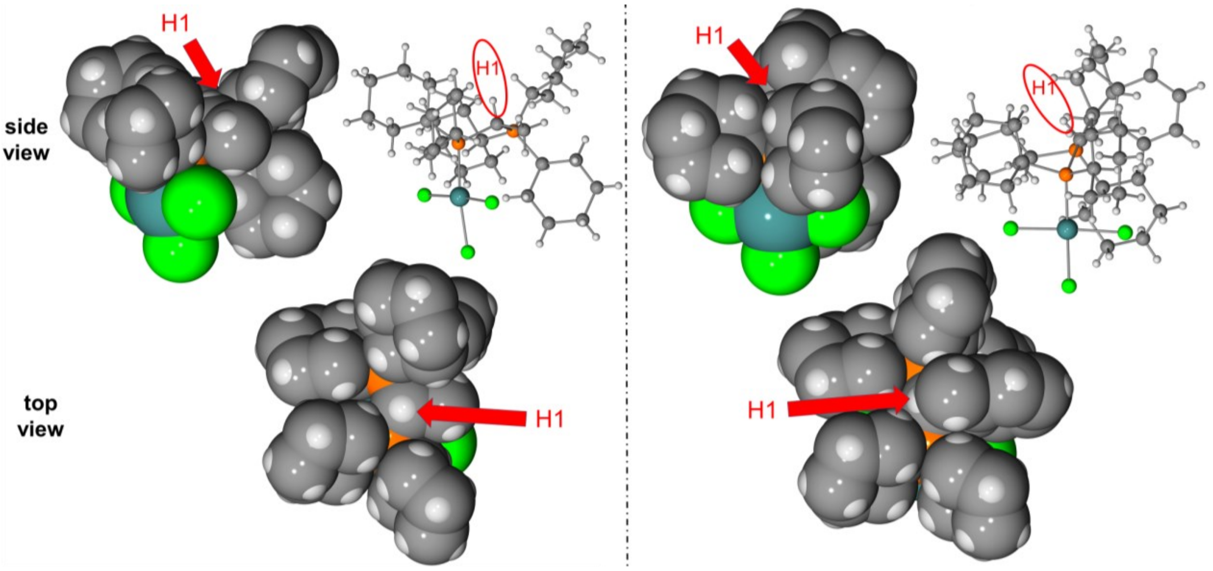

Figure S3. Comparison of the van-der-Waals plots of **L1H·$PdCl_3$** (left) and **L4H·$PdCl_3$** (right). The plots show the different shielding of the central hydrogen atom H1 by the cyclohexyl groups as well es the Z substituents. In case of **L1**, the smaller methyl group leaves one side open and thus H1 accessible to bases for deprotonation. In contrast, H1 is highly shielded also by the ortho-tolyl group, making it difficult to be accessed by bases.

2.3 H–Pd interaction in L3·$PdCl_2$ and L4·$PdCl_2$

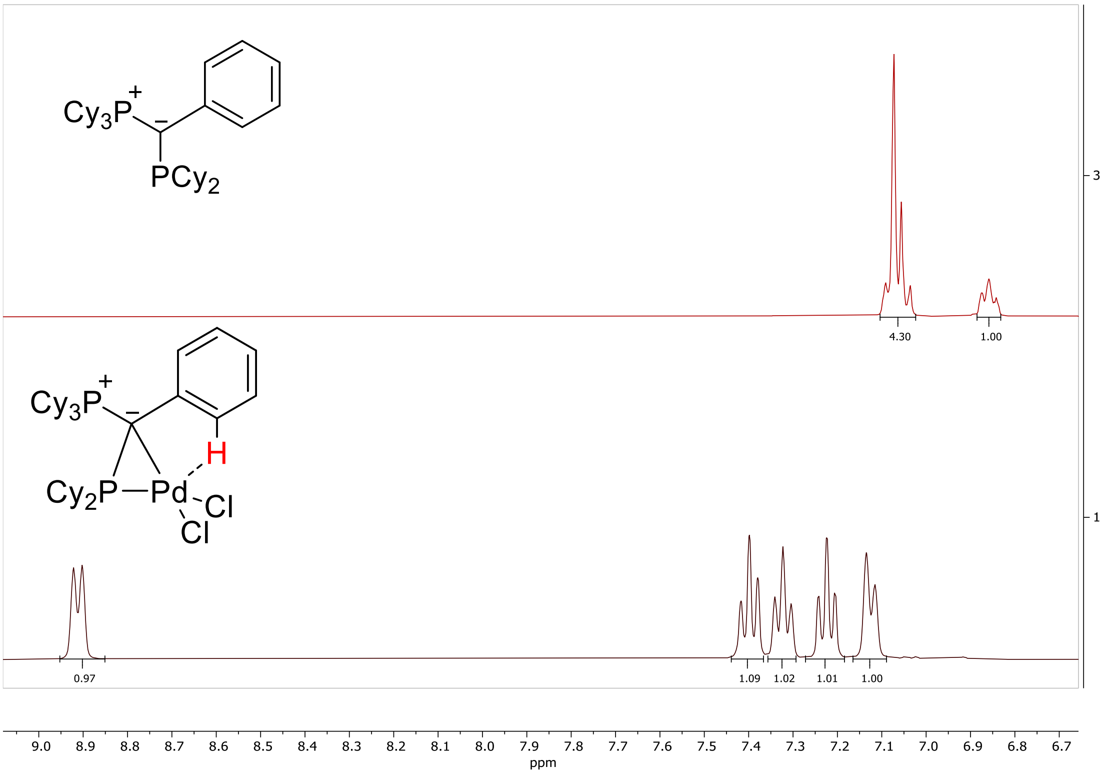

Figure S4. Comparison of the chemical ^{1}H NMR shifts in aromatic area of **L3** and **L3·$PdCl_2$** in CD_2Cl_2.

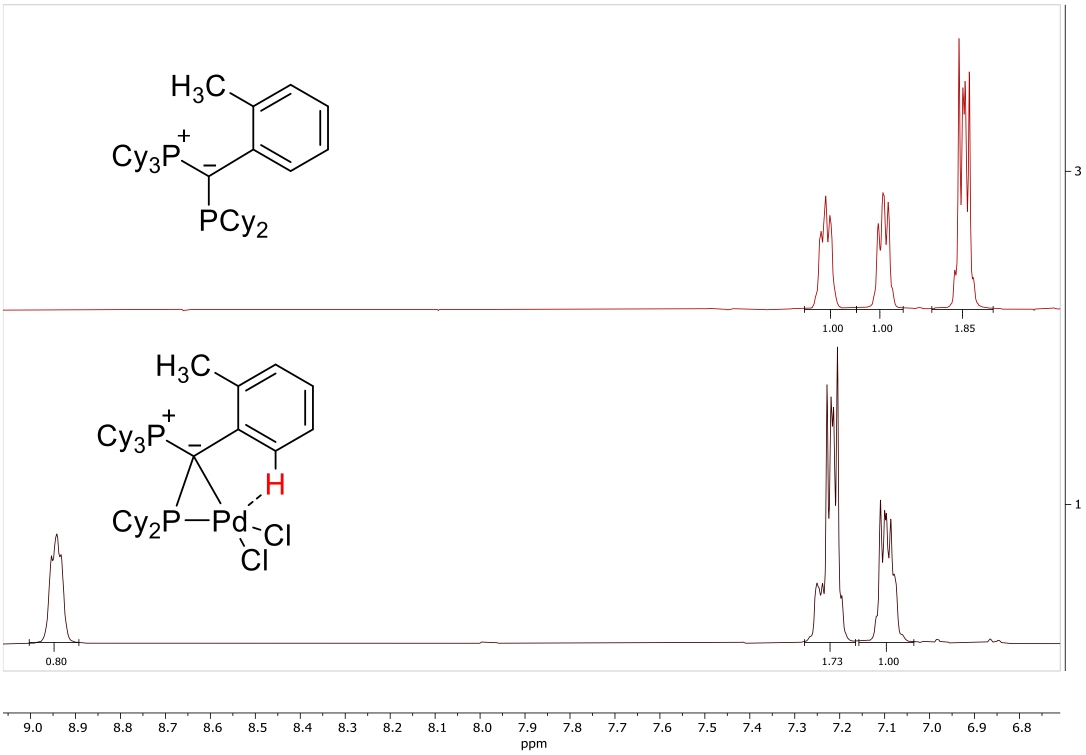

Figure S5. Comparison of the chemical ^{1}H NMR shifts in aromatic area of **L4** and **L4·PdCl$_2$**

2.4 Reaction Kinetics

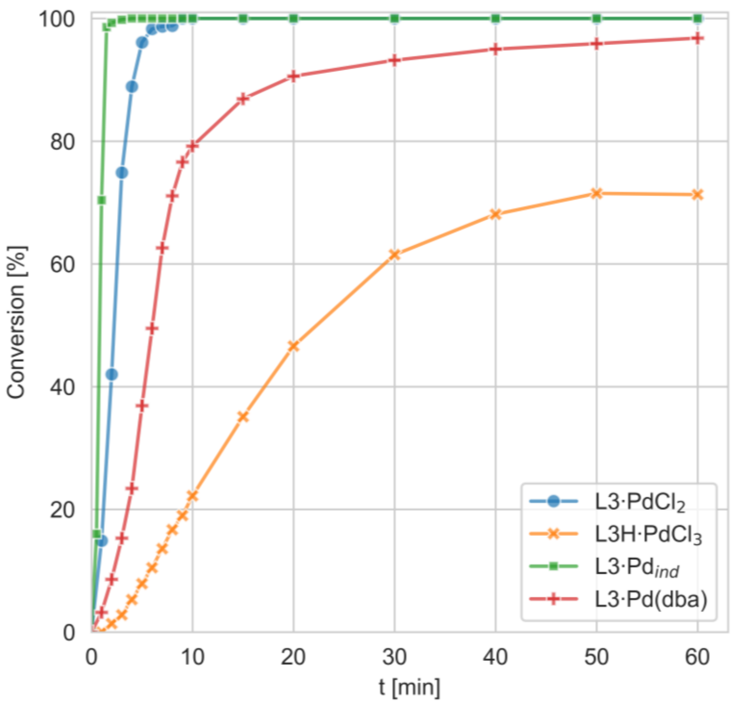

Figure S6. Conversion time plots for the C-N coupling of *p*-tolyl chloride with piperidine with different precatalysts of **joYPhos** (**L3**). Conditions: 0.5 mol% precatalyst, chloride:amine = 1:1.1; 1.5 eq. KOtBu, THF, room temperature.

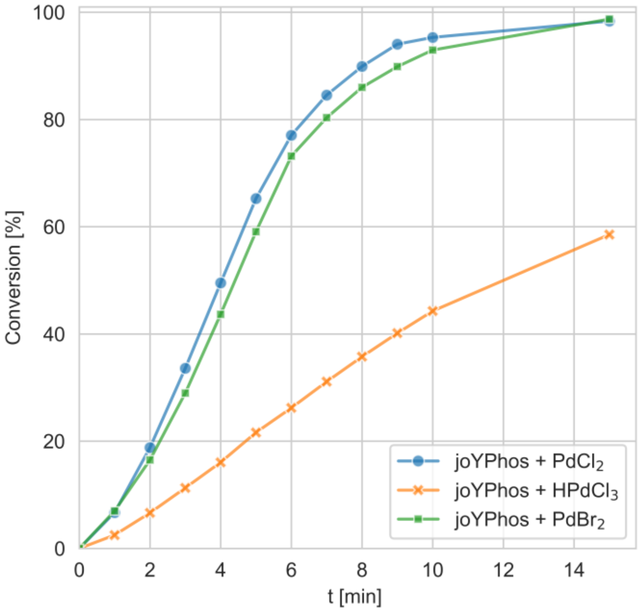

Figure S7. Conversion time plots for the C-N coupling of *p*-tolyl chloride with piperidine with different precatalysts of **joYPhos** (**L3**). Conditions: 0.5 mol% precatalyst, chloride:amine = 1:1.1; 1.5 eq. KOtBu, THF, room temperature. Note: This experiment was conducted on different ambient conditions than the experiment in **Figure S6**, therefore the results were different.

3. NMR Spectra

3.1 NMR Spectra of the Ligands and Complexes

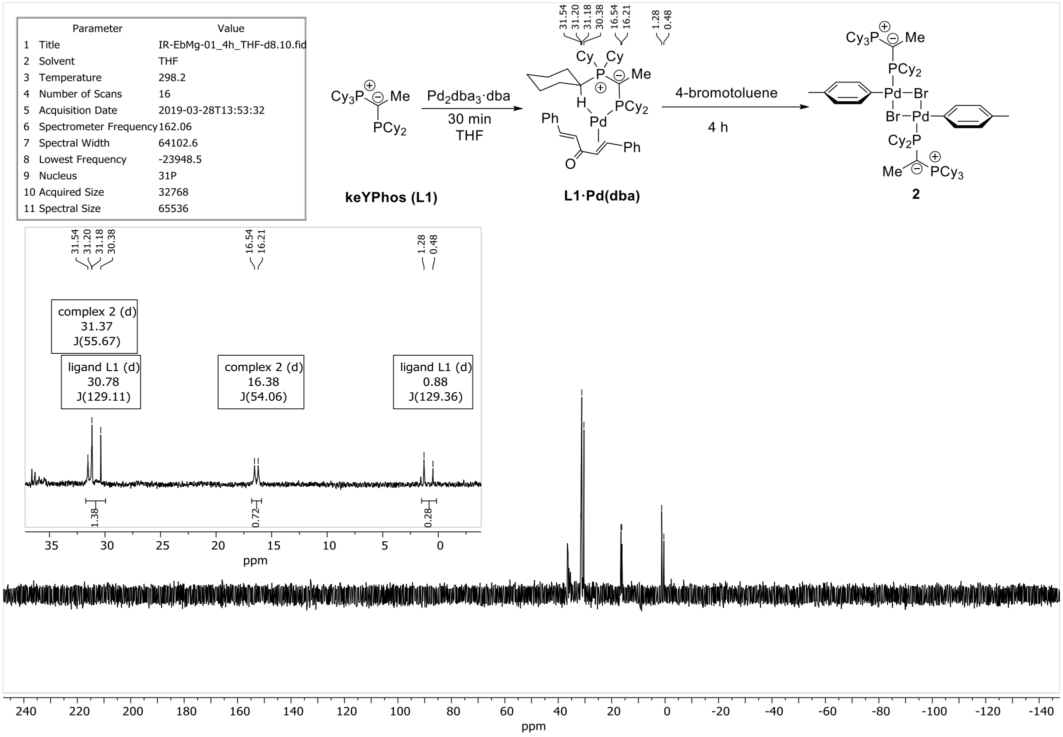

Figure S8. $^{31}P\{^{1}H\}$ NMR spectrum of reaction solution of **L1·Pd(dba)** with an excess of 4-bromotoluene to form complex **2**.

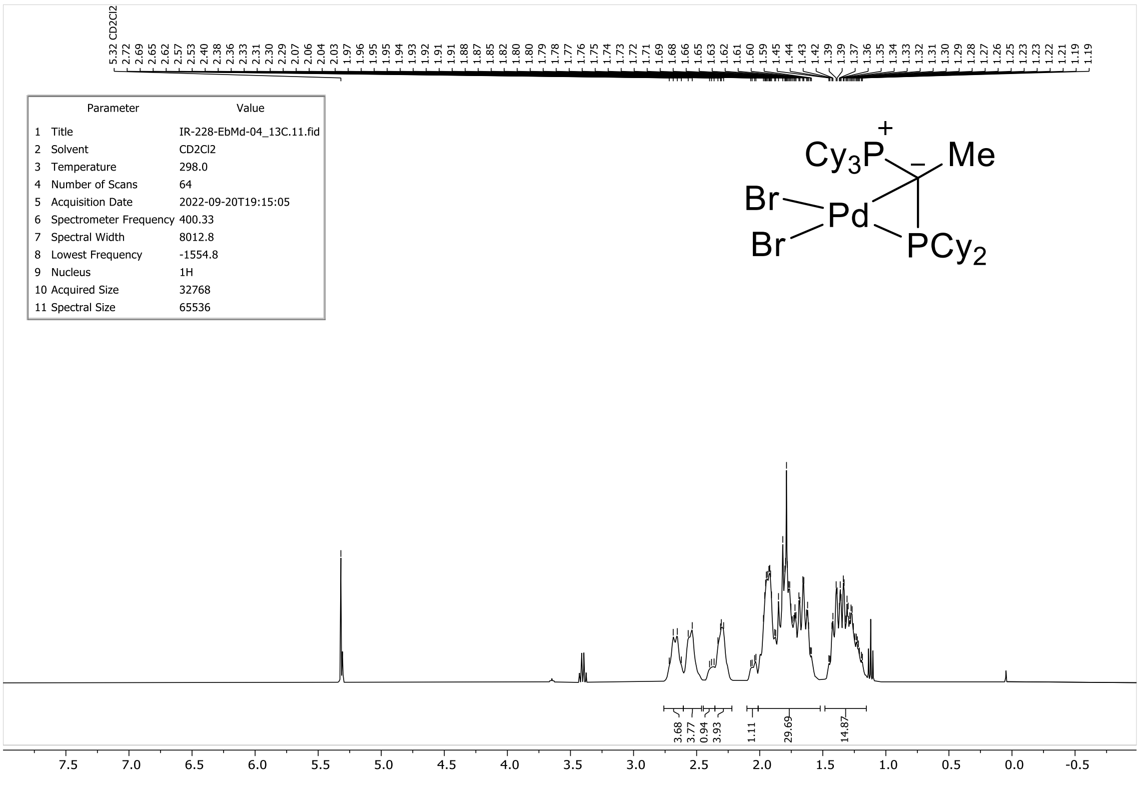

Figure S9a. ^{1}H NMR spectrum of **L1·PdBr$_2$** (+residual diethylether).

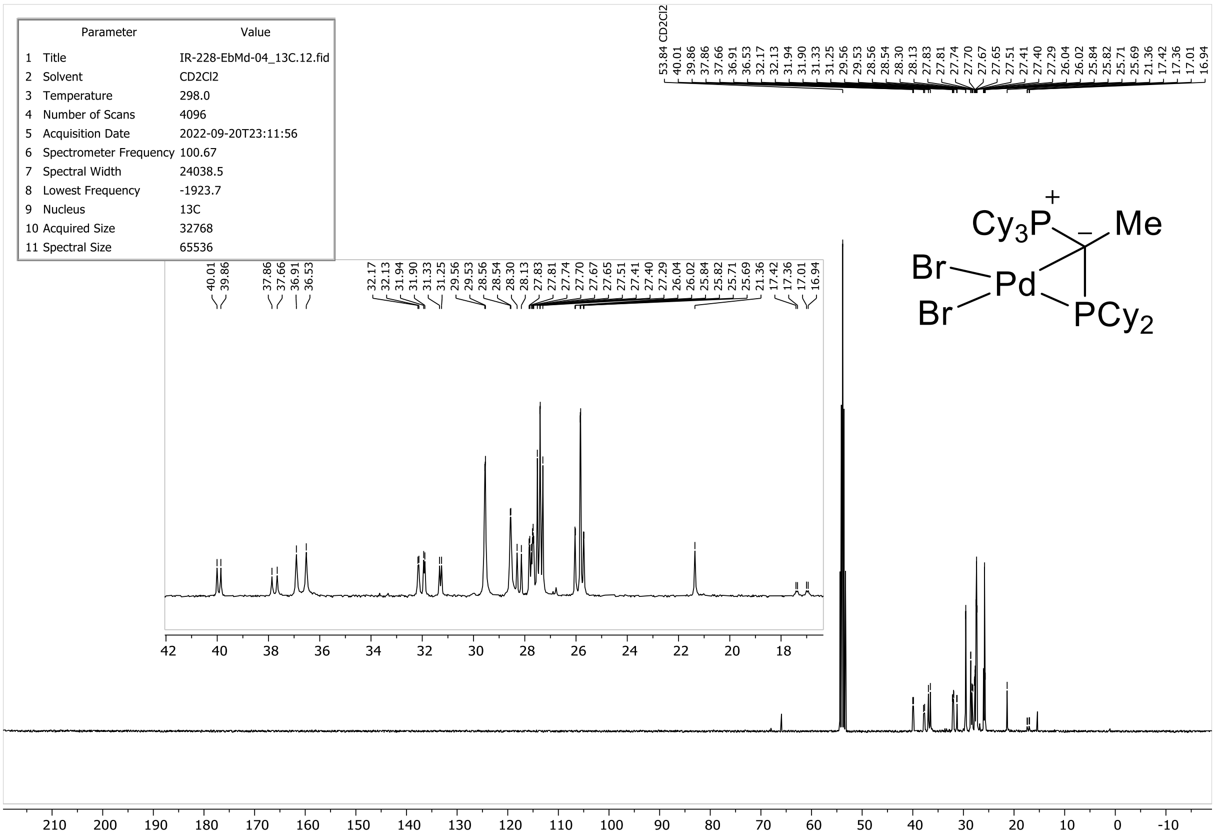

Figure S9b. $^{13}C\{^1H\}$ NMR spectrum of **L1·PdBr$_2$**.

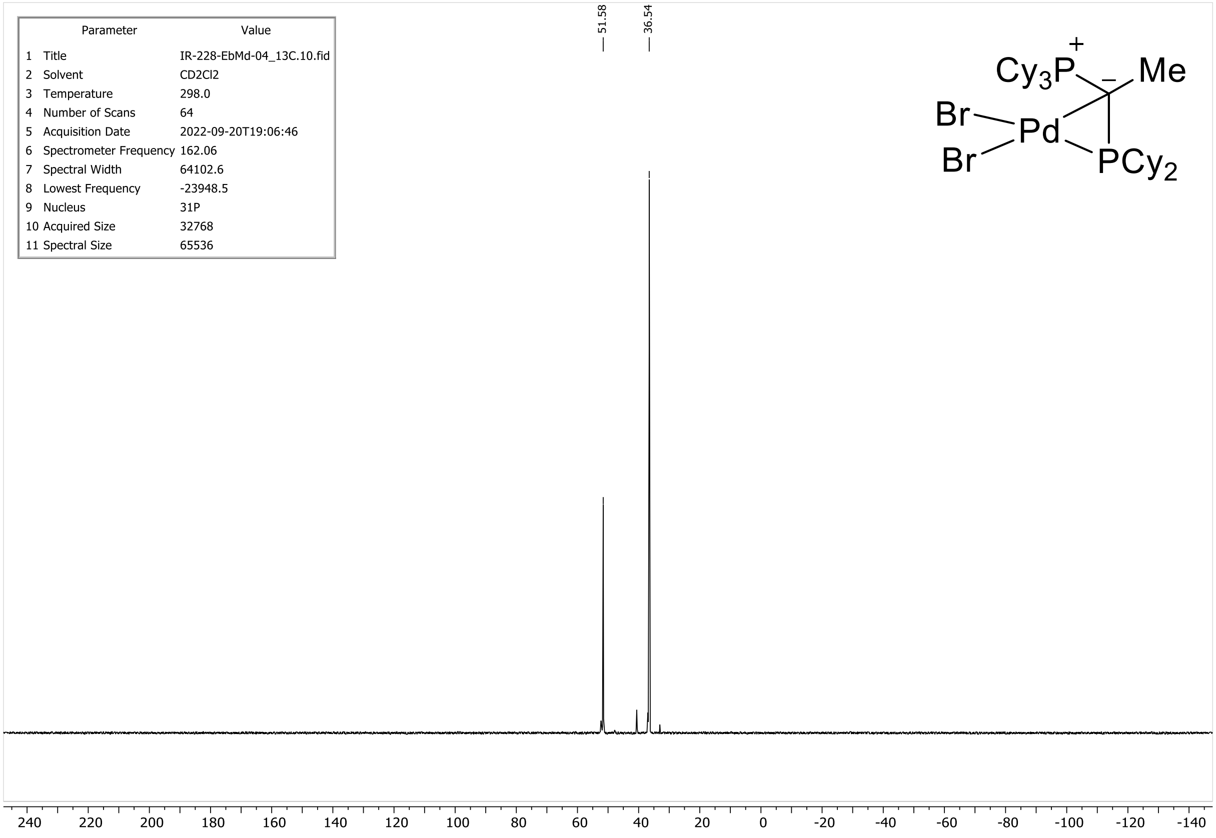

Figure S9c. $^{31}P\{^1H\}$ NMR spectrum of **L1·PdBr$_2$**.

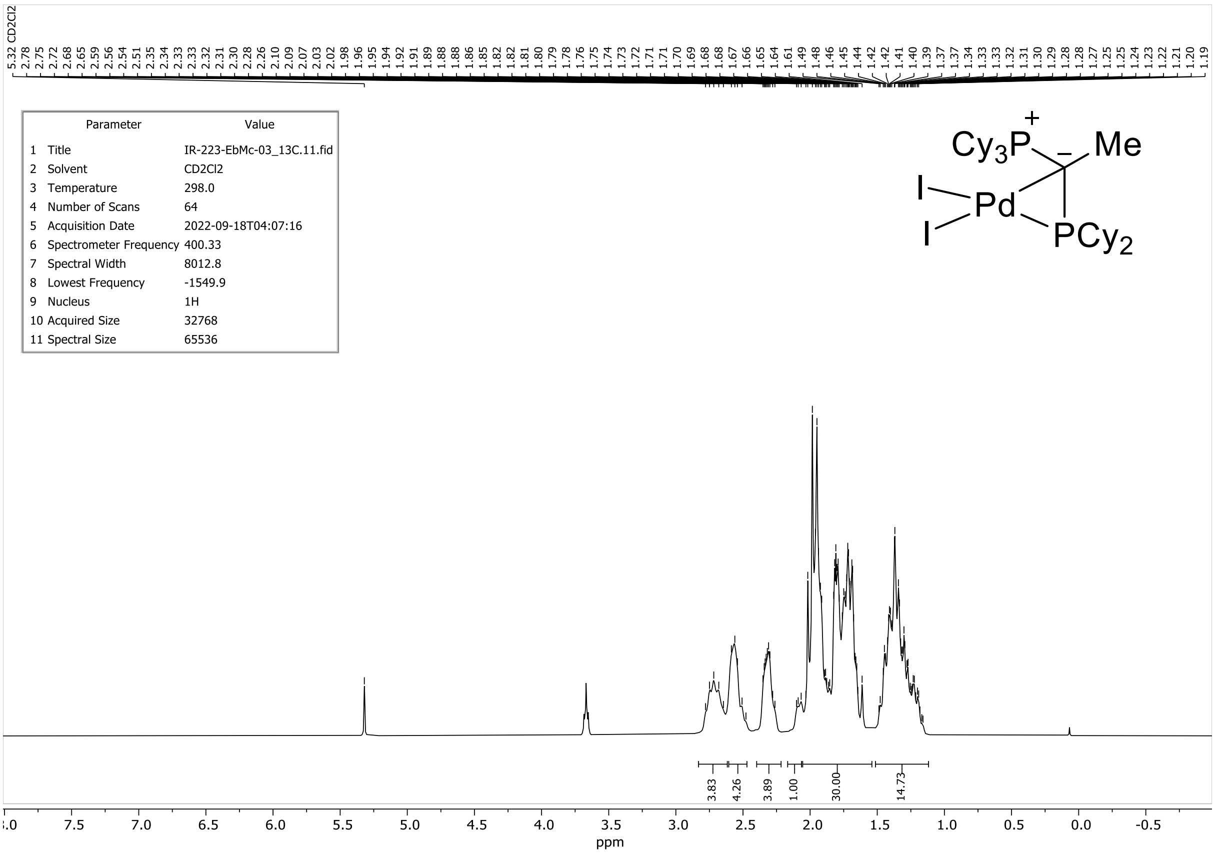

Figure S10a. ^{1}H NMR spectrum of **L1·PdI$_2$** (+residual THF).

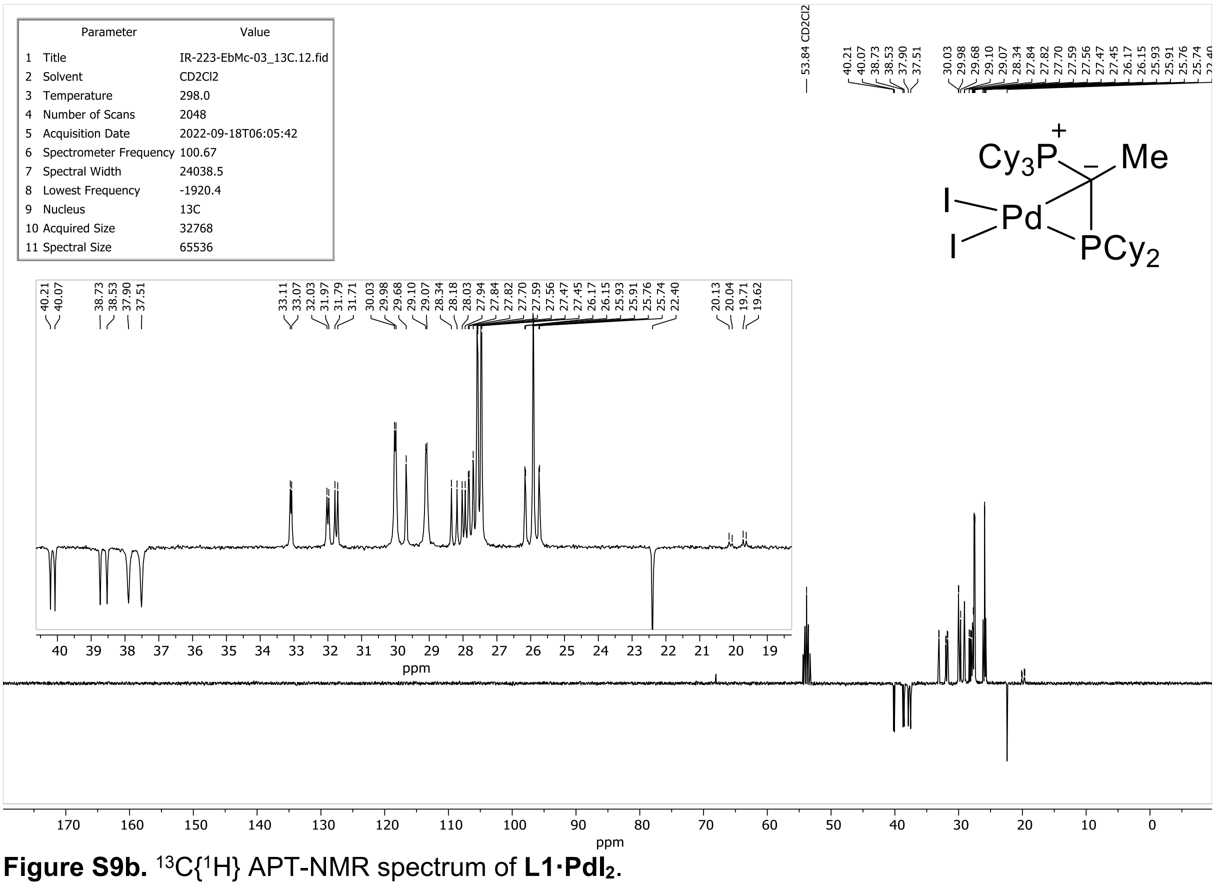

Figure S9b. $^{13}C\{^{1}H\}$ APT-NMR spectrum of **L1·PdI$_2$**.

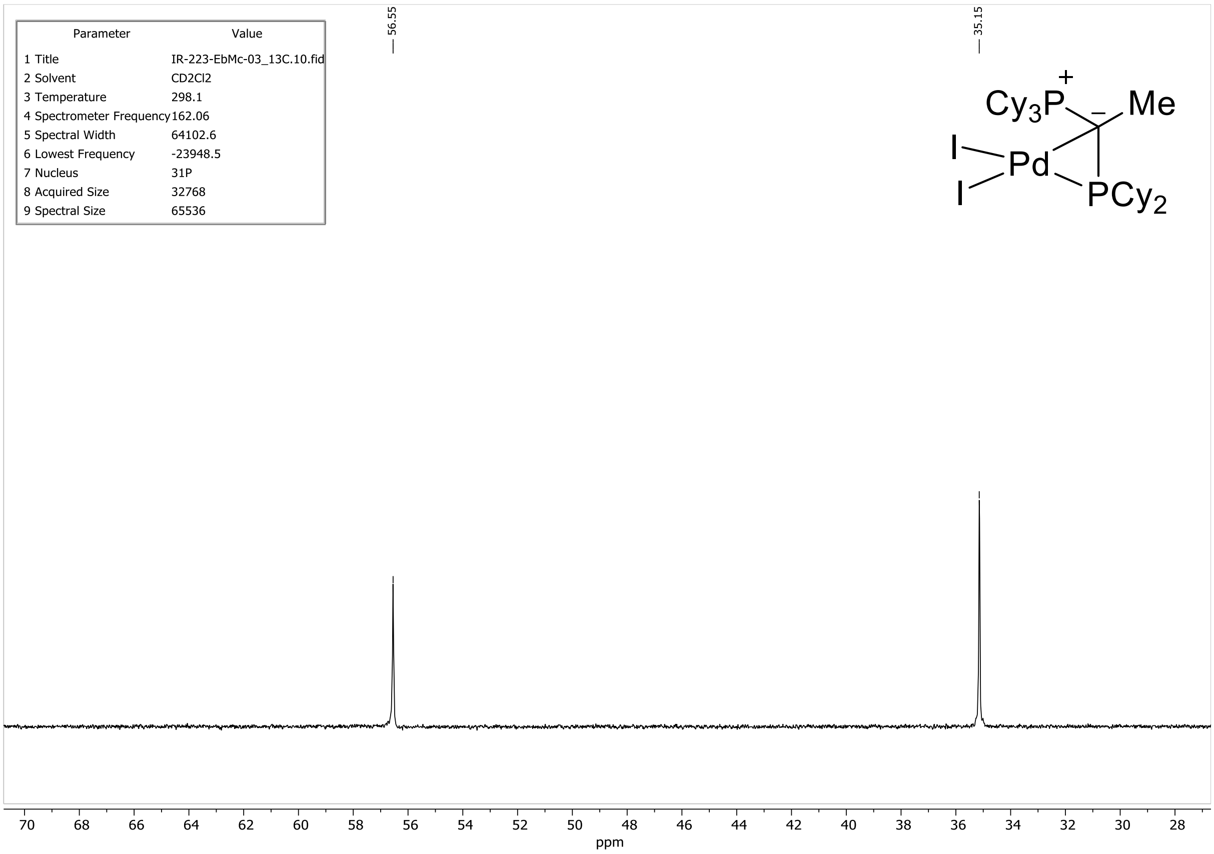

Figure S9c. $^{31}P\{^1H\}$ NMR spectrum of **L1·PdI$_2$**.

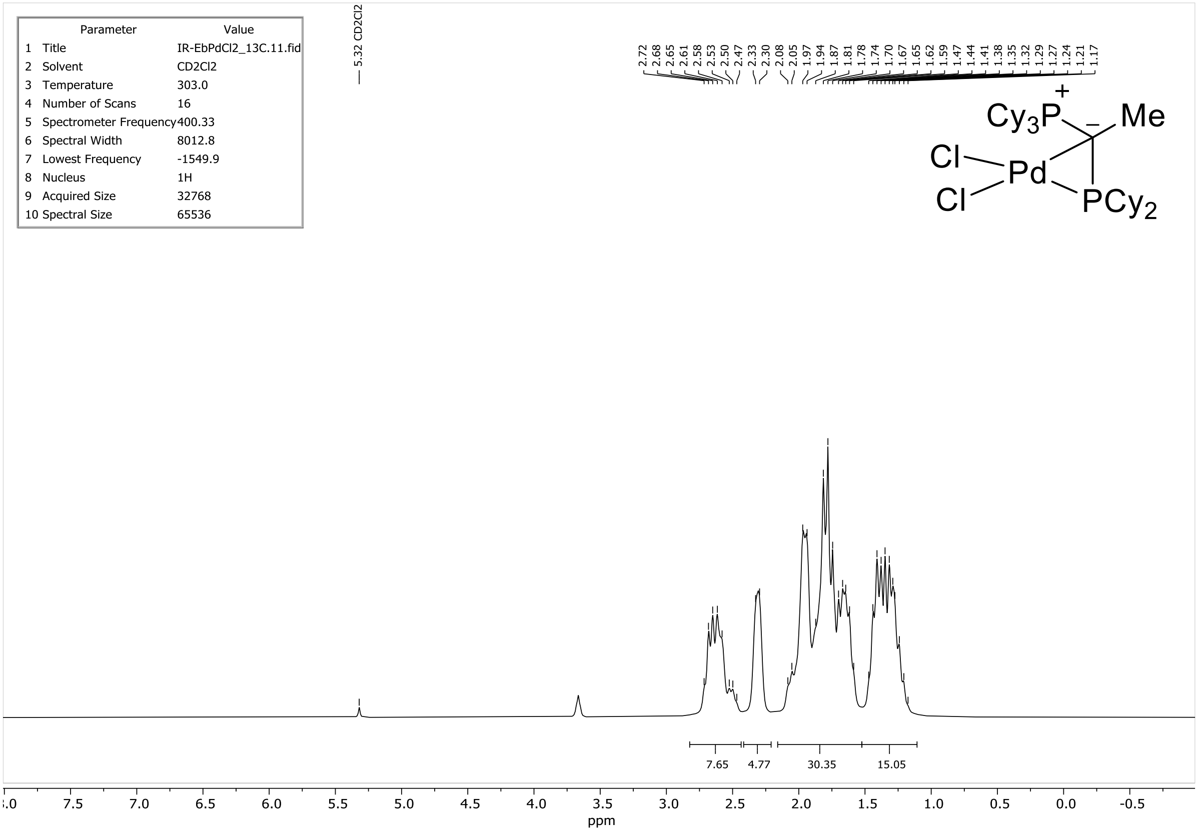

Figure S11a. ^{1}H NMR spectrum of **L1·PdCl₂** (+residual THF).

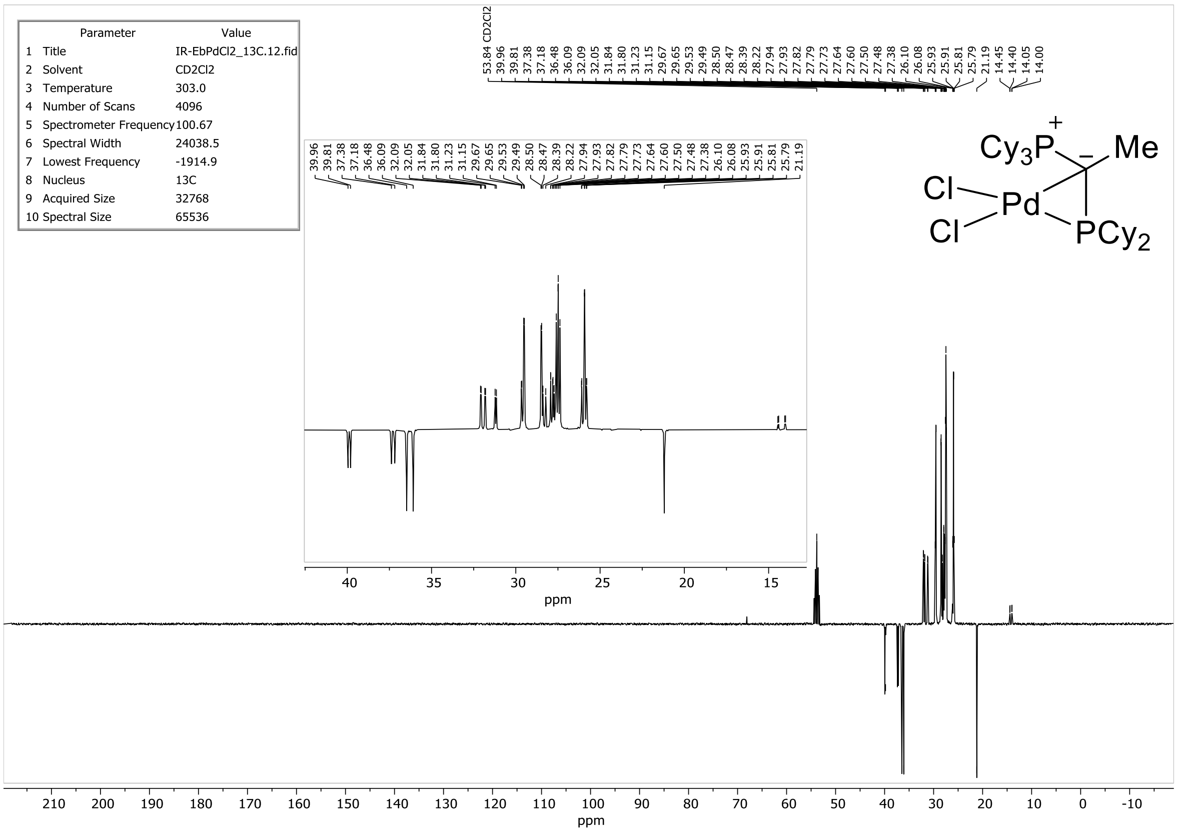

Figure S11b. $^{13}C\{^{1}H\}$ APT-NMR spectrum of **L1·PdCl₂**.

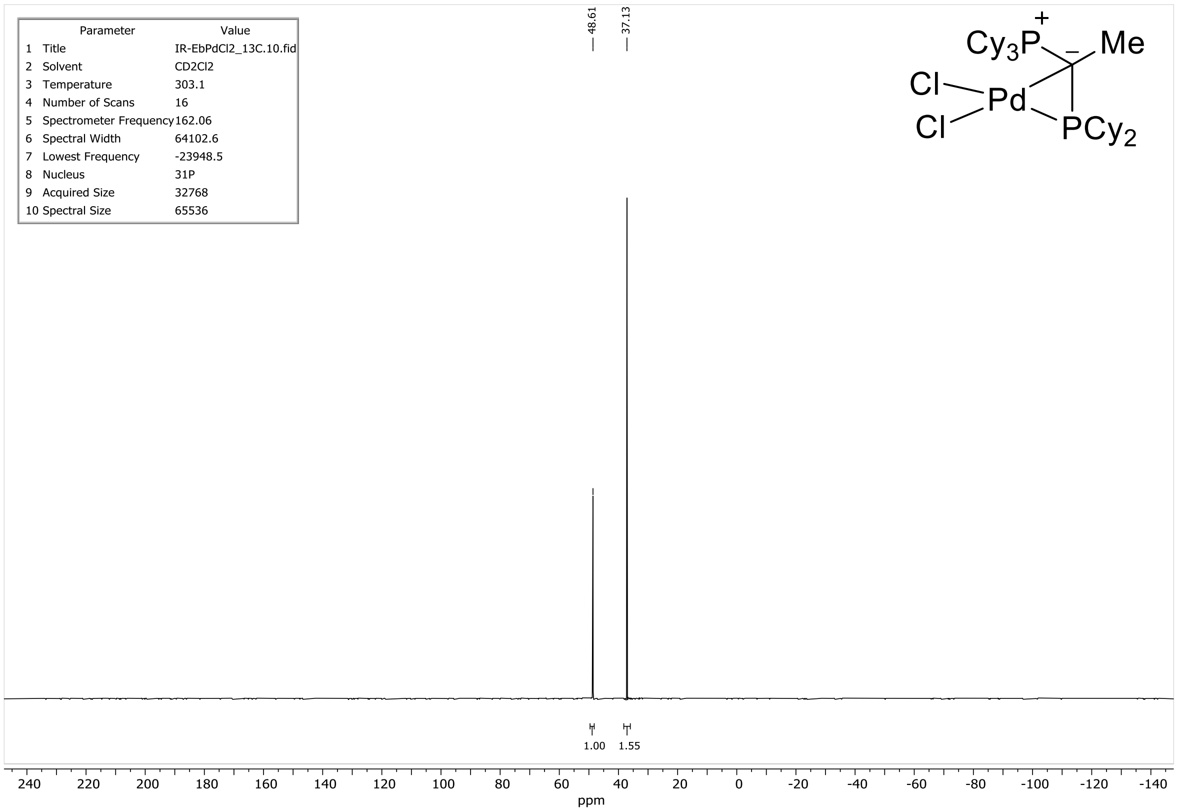

Figure S11c. $^{31}P\{^{1}H\}$ NMR spectrum of **L1·PdCl₂**.

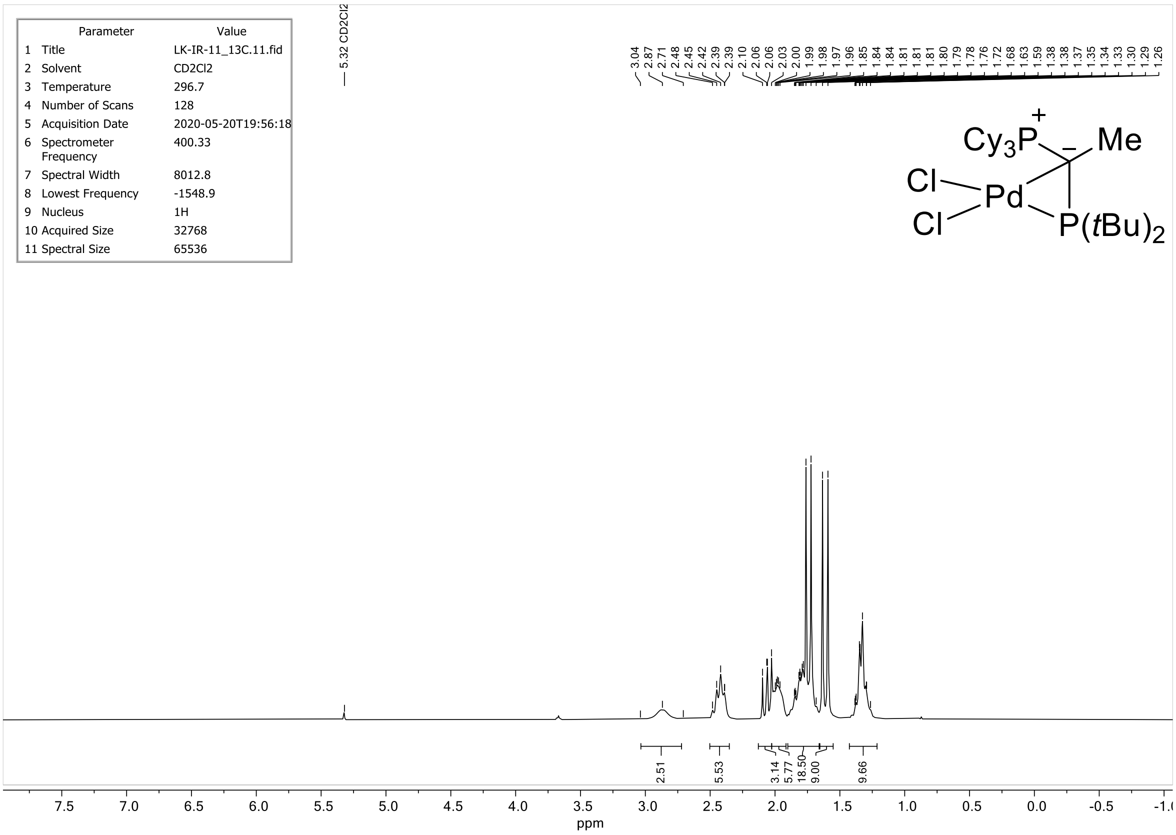

Figure S12a. ^{1}H NMR spectrum of **L2·PdCl₂**.

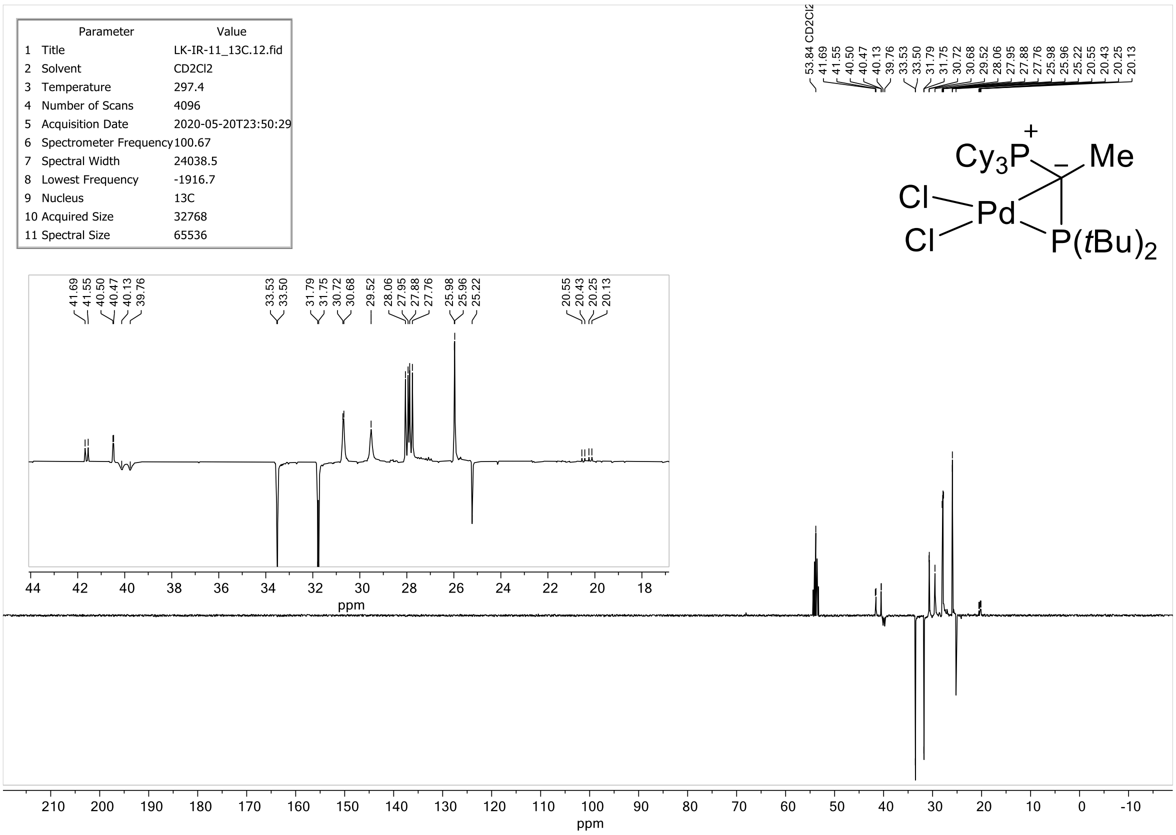

Figure S12b. $^{13}C\{^{1}H\}$ APT-NMR spectrum of **L2·PdCl₂**.

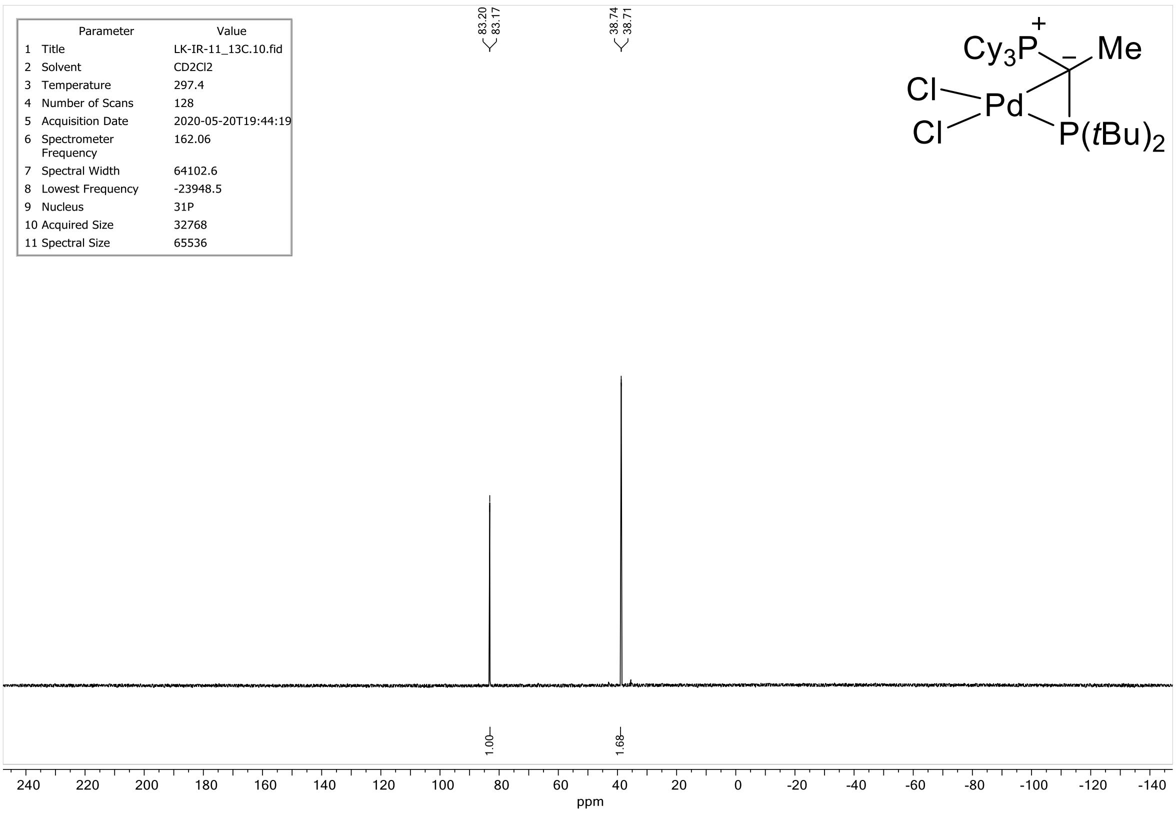

Figure S12c. ^{31}P{^{1}H} NMR spectrum of **L2·PdCl$_2$**.

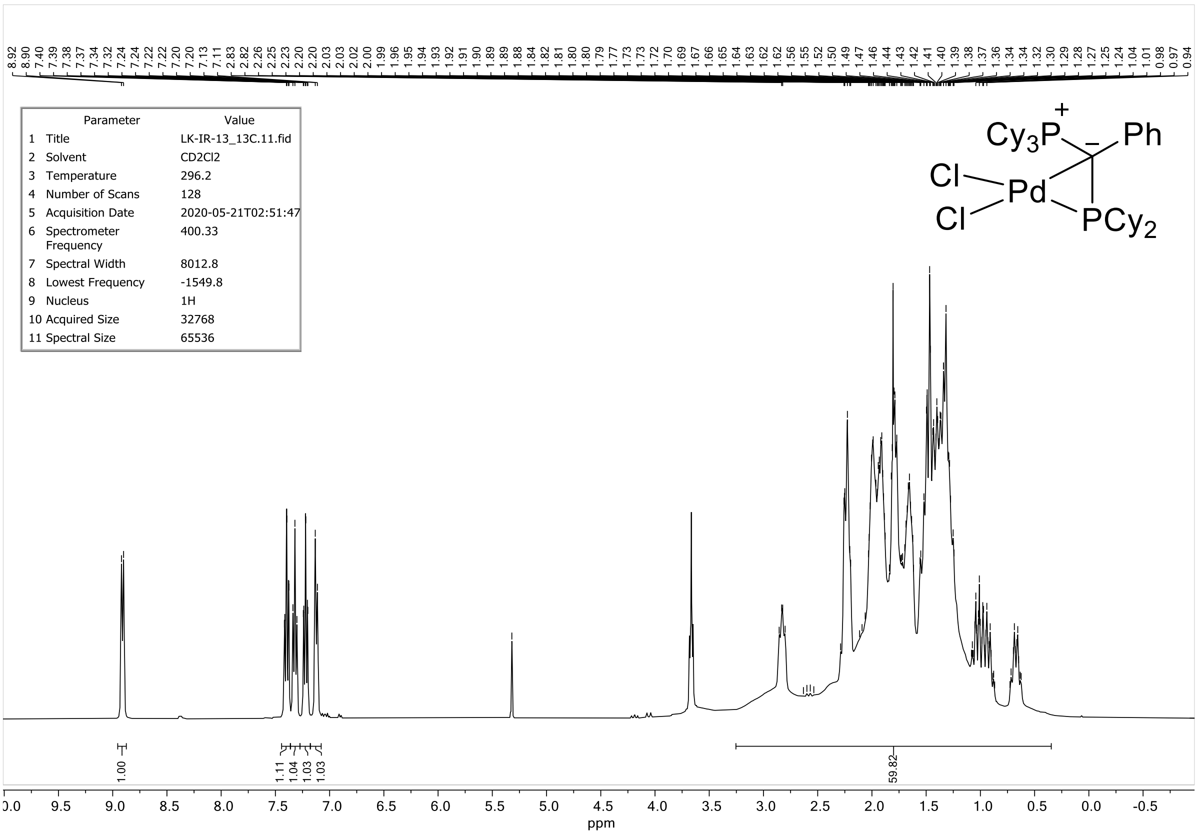

Figure S13a. ^{1}H NMR spectrum of **L3·PdCl$_2$** (+ residual THF).

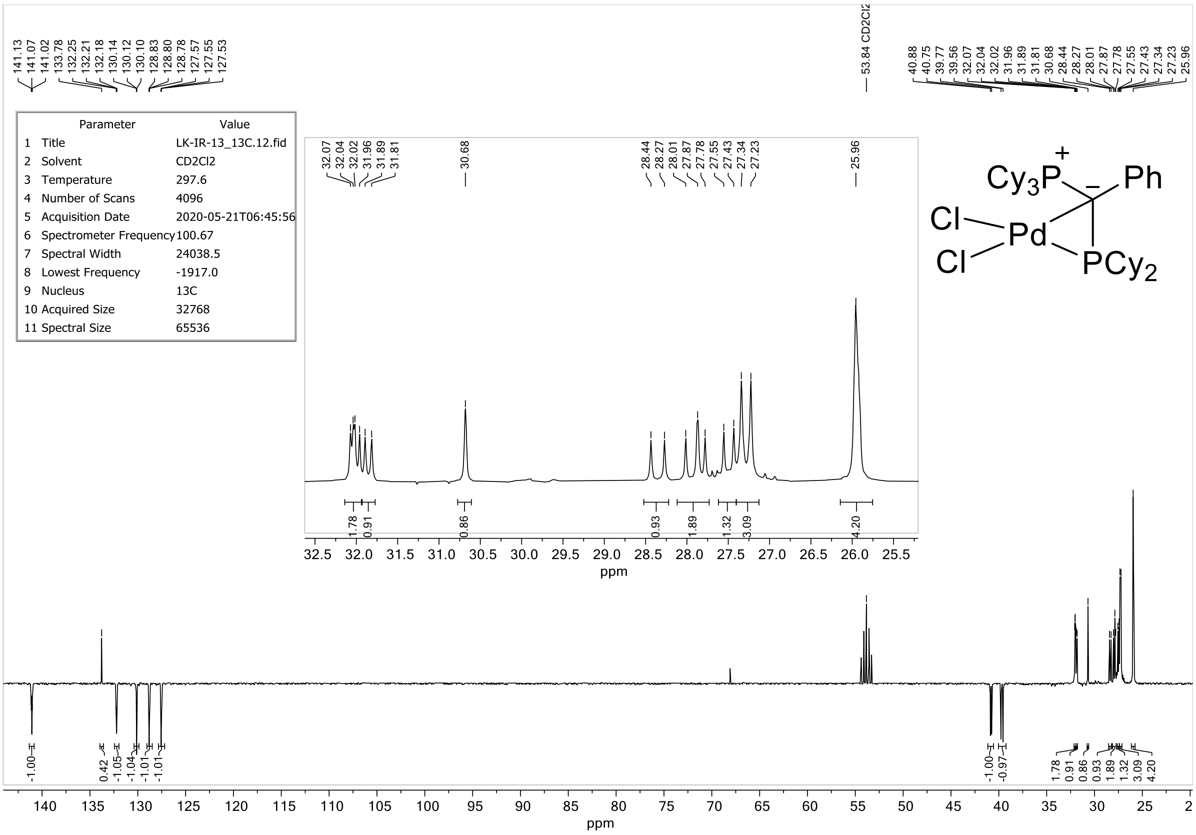

Figure S13b. $^{13}C\{^1H\}$ APT-NMR spectrum of **L3·PdCl₂**.

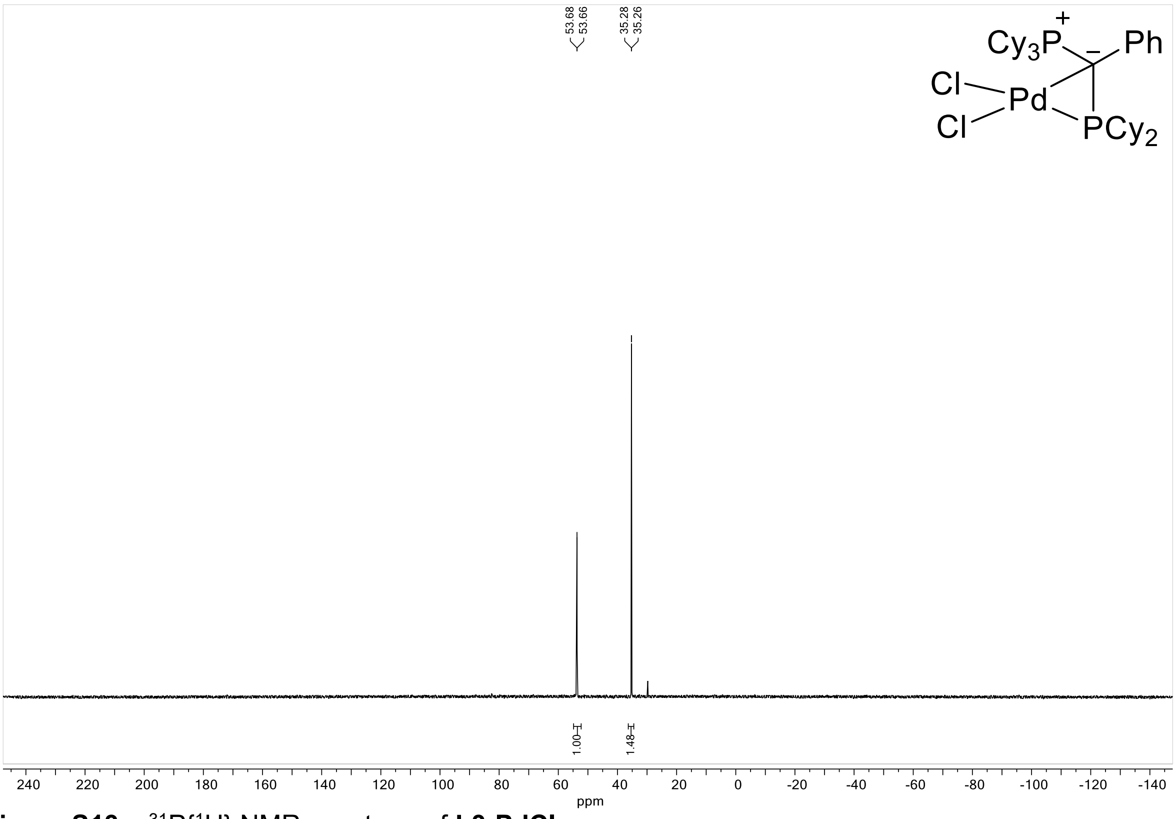

Figure S13c. $^{31}P\{^1H\}$ NMR spectrum of **L3·PdCl₂**.

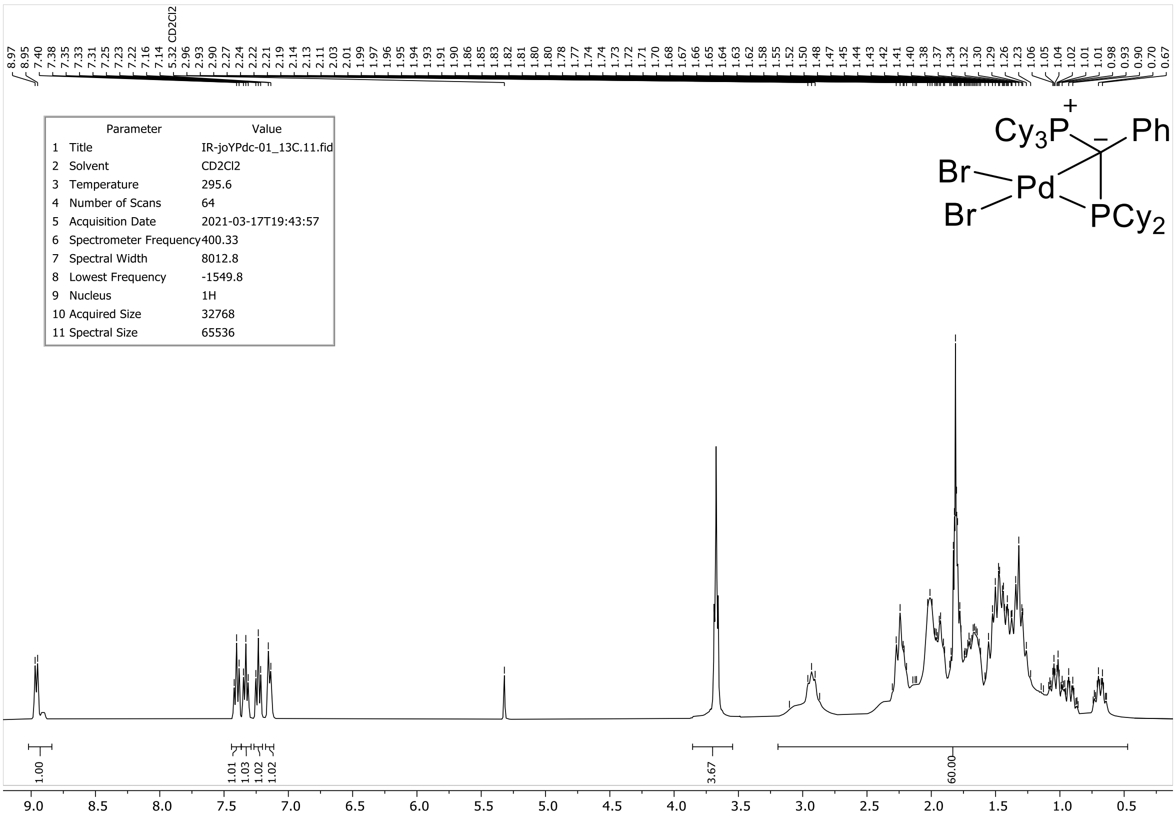

Figure S14a. ^{1}H NMR spectrum of **L3·PdBr₂** (+residual THF).

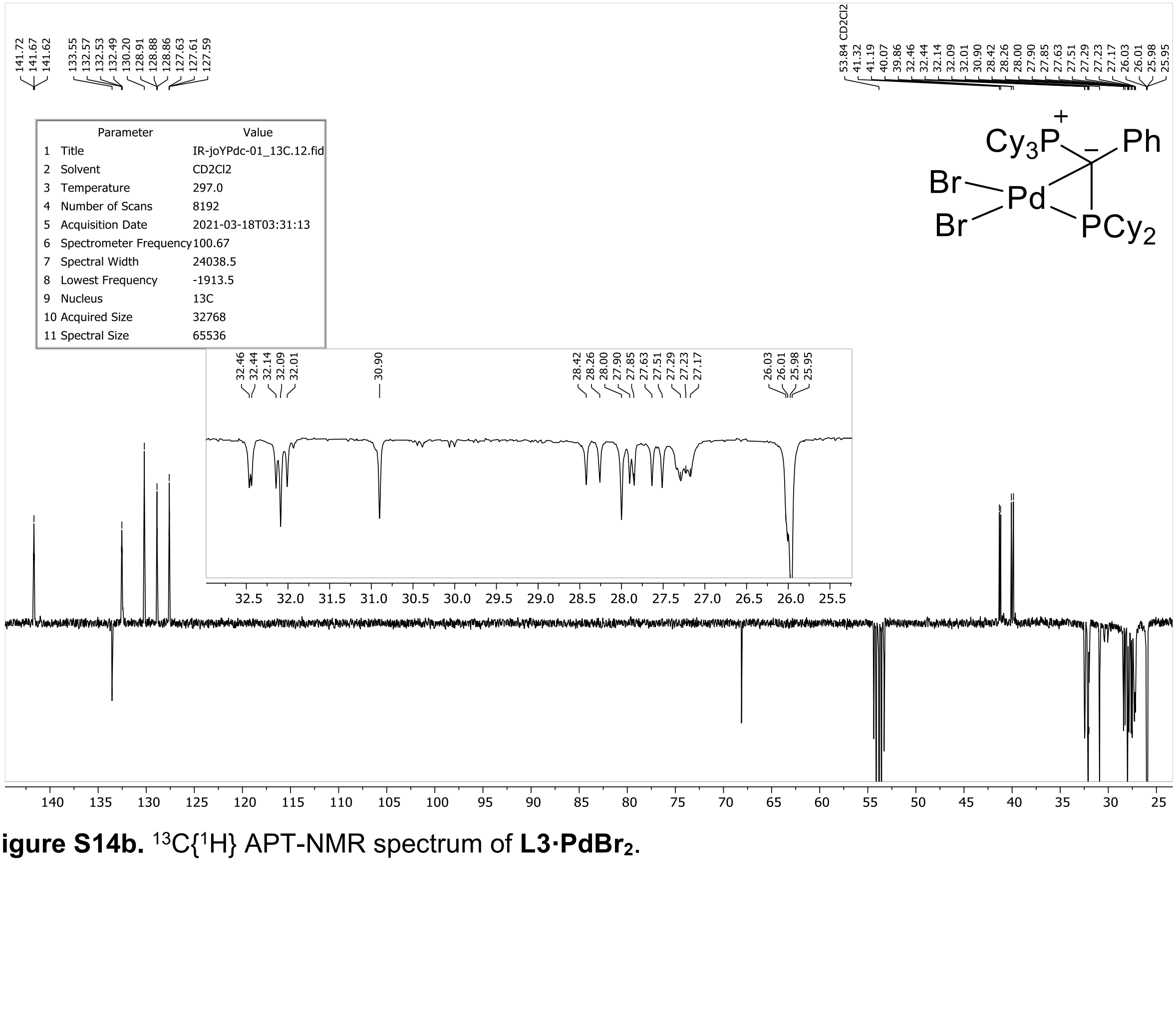

Figure S14b. $^{13}C\{^{1}H\}$ APT-NMR spectrum of **L3·PdBr₂**.

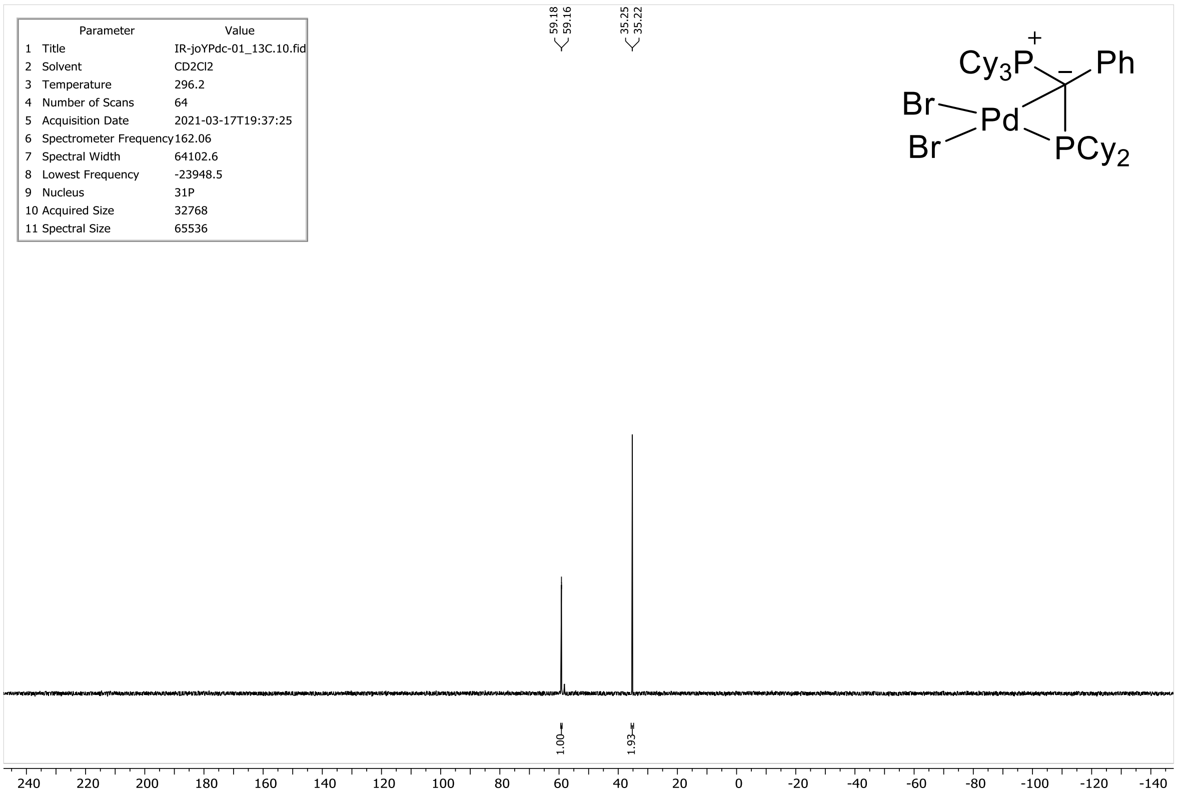

Figure S14c. $^{31}P\{^{1}H\}$ NMR spectrum of **L3·PdBr₂**.

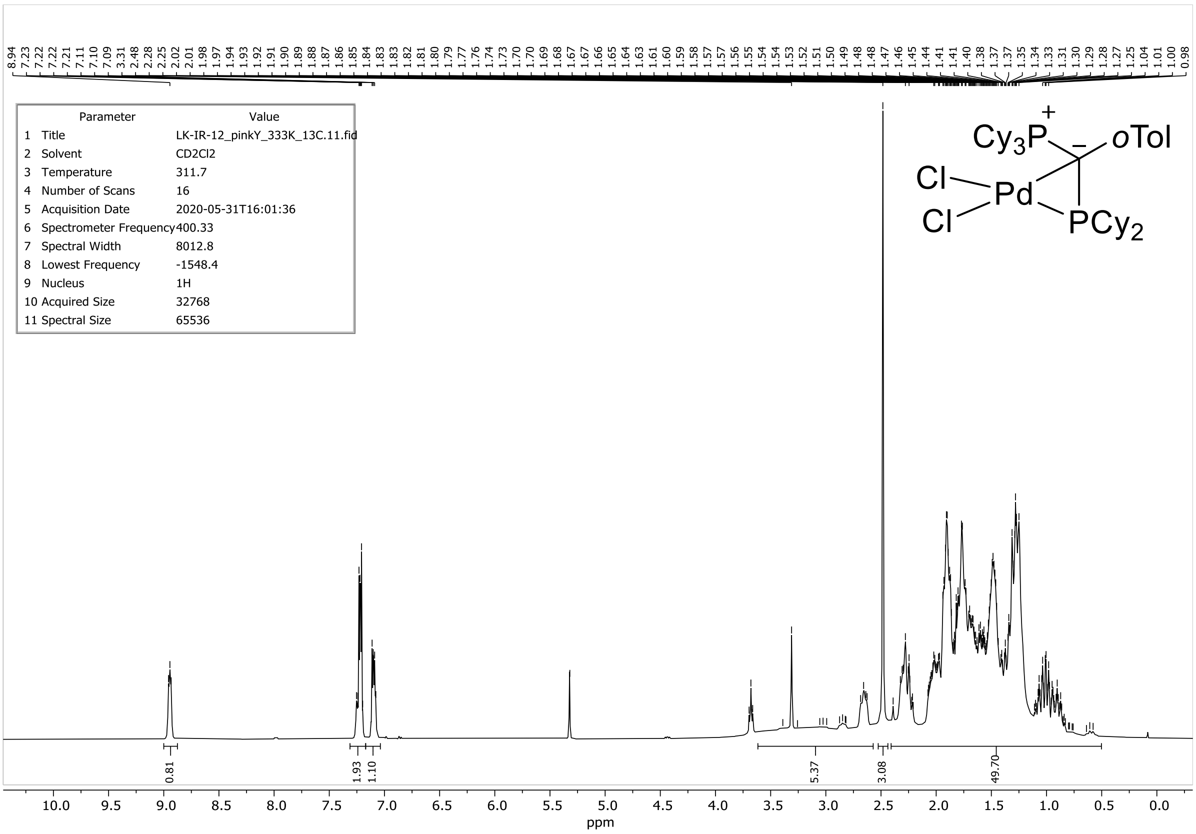

Figure S15a. ^{1}H NMR spectrum of **L4·PdCl₂** (+residual THF).

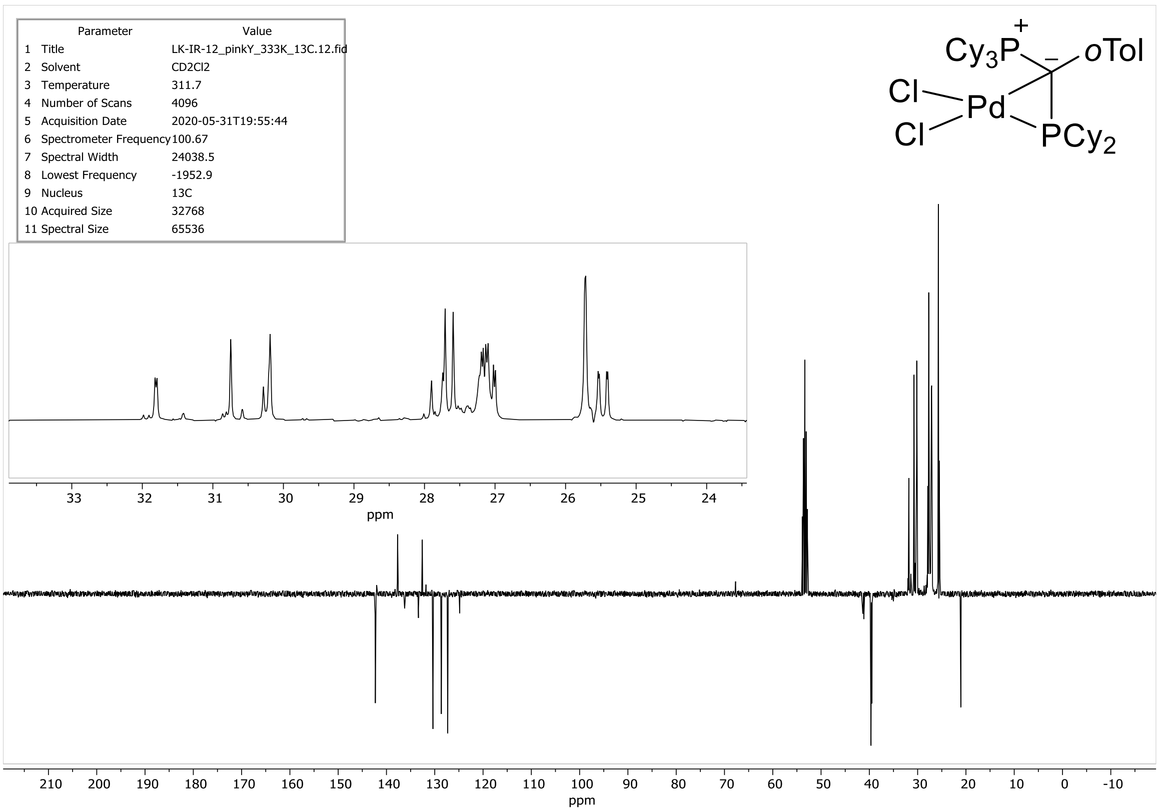

Figure S15b. $^{13}C\{^{1}H\}$ APT-NMR spectrum of **L4·PdCl₂** (isomeric mixture).

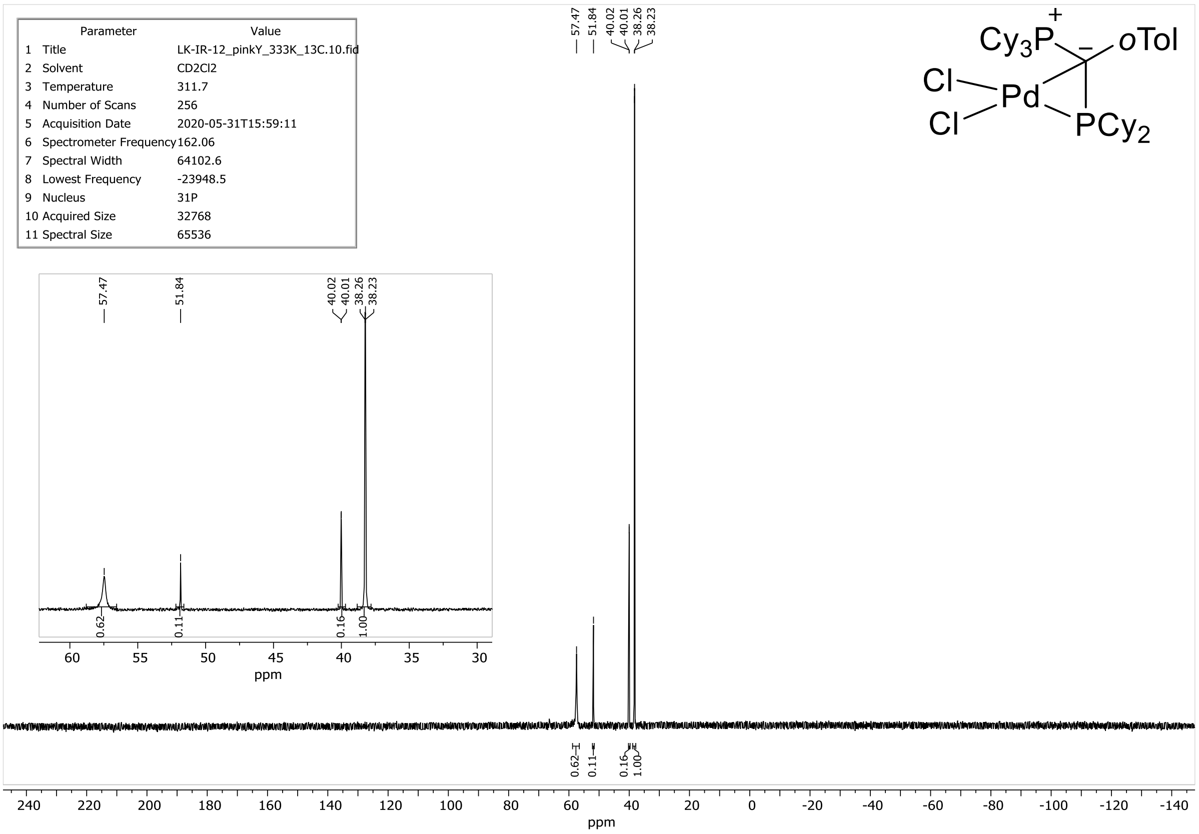

Figure S15c. $^{31}P\{^{1}H\}$ NMR spectrum of **L4·PdCl₂** (isomeric mixture).

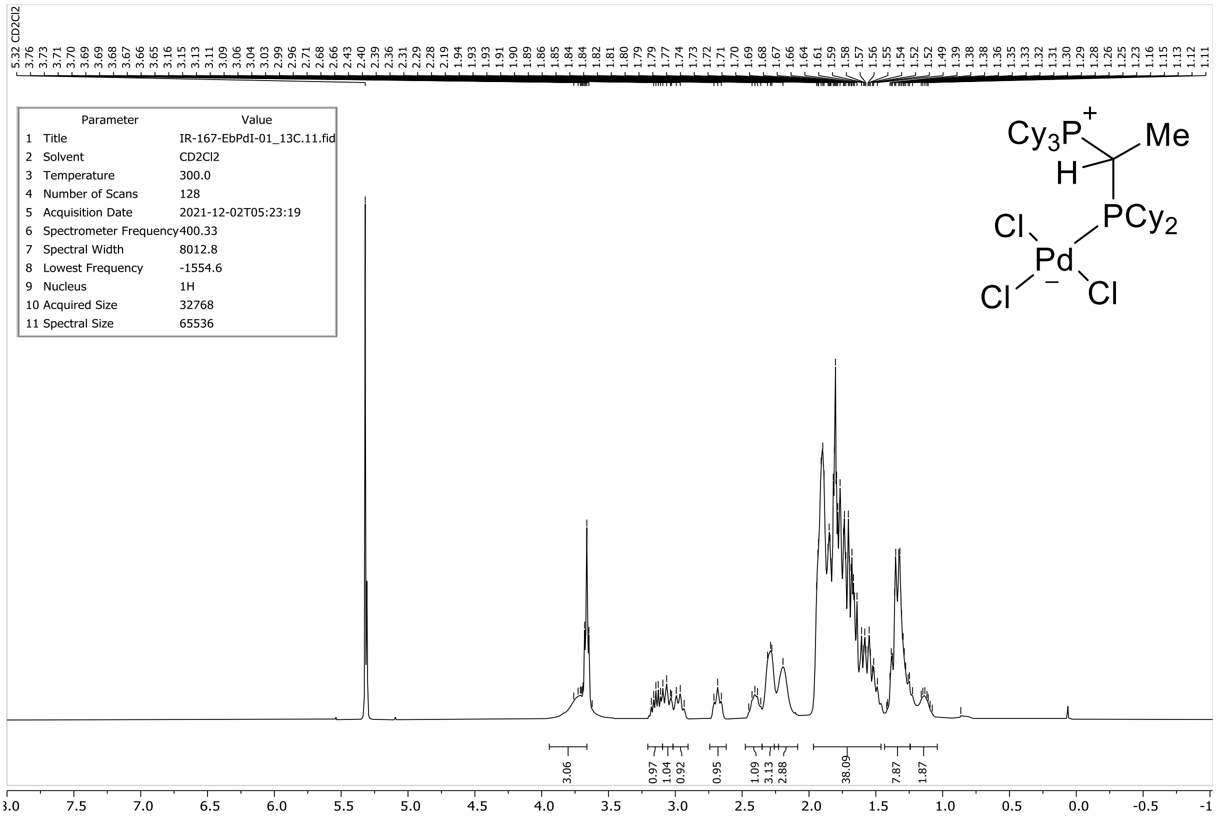

Figure S16a. ^{1}H NMR spectrum of **L1H·PdCl$_3$**.

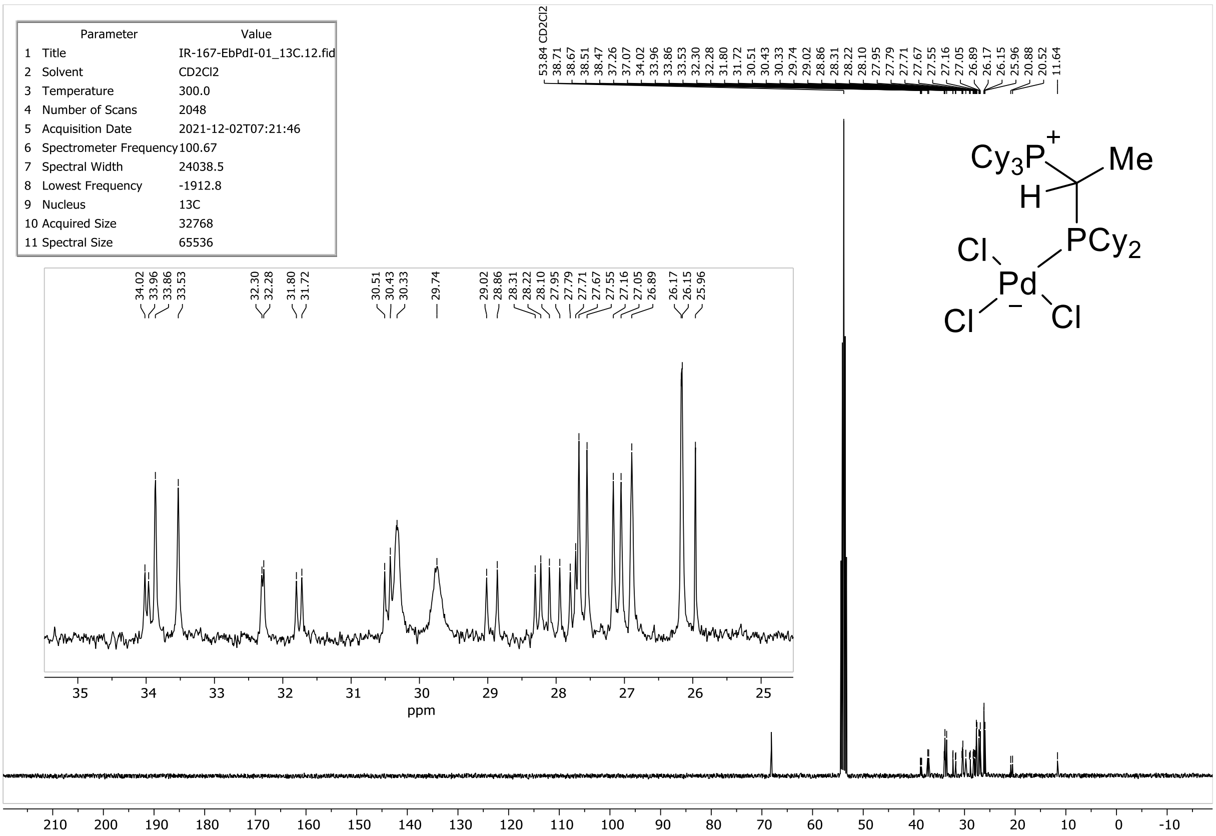

Figure S16b. $^{13}C\{^{1}H\}$ NMR spectrum of **L1H·PdCl$_3$**.

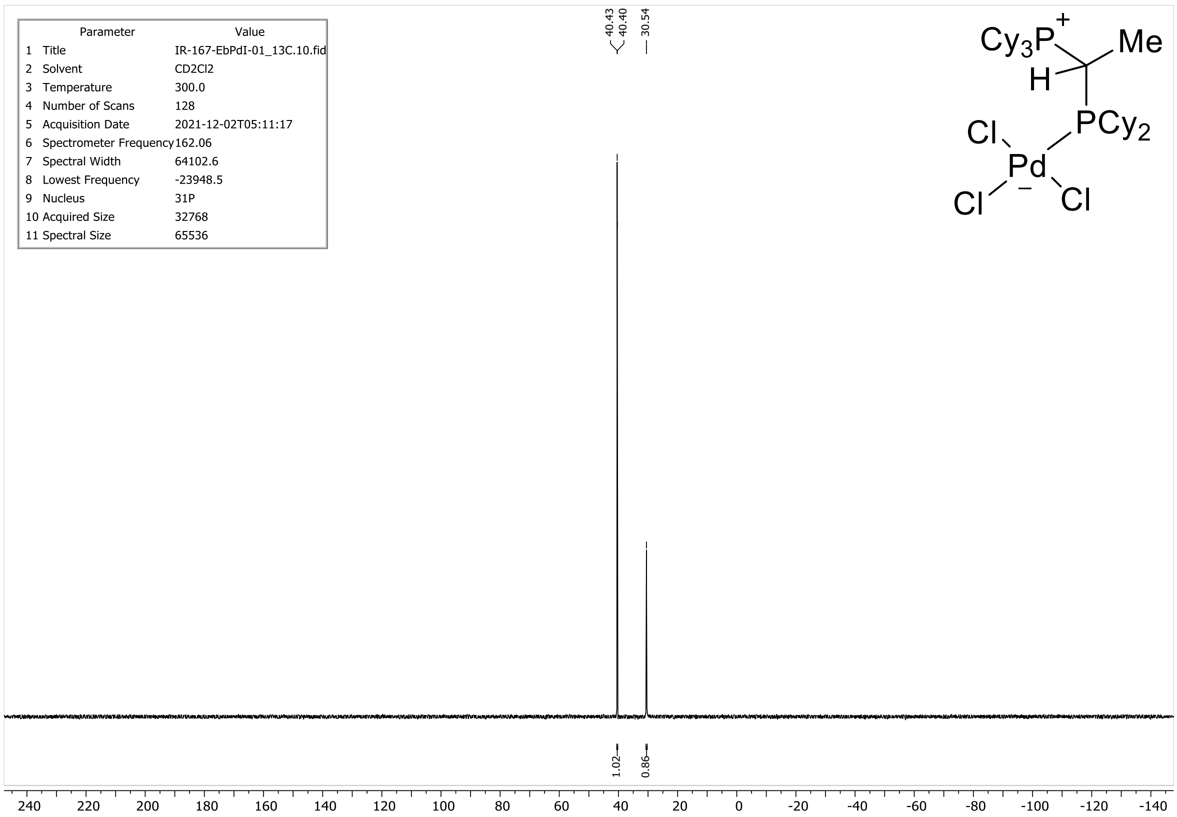

Figure S16c. $^{31}P\{^{1}H\}$ NMR spectrum of **L1H·PdCl$_3$**.

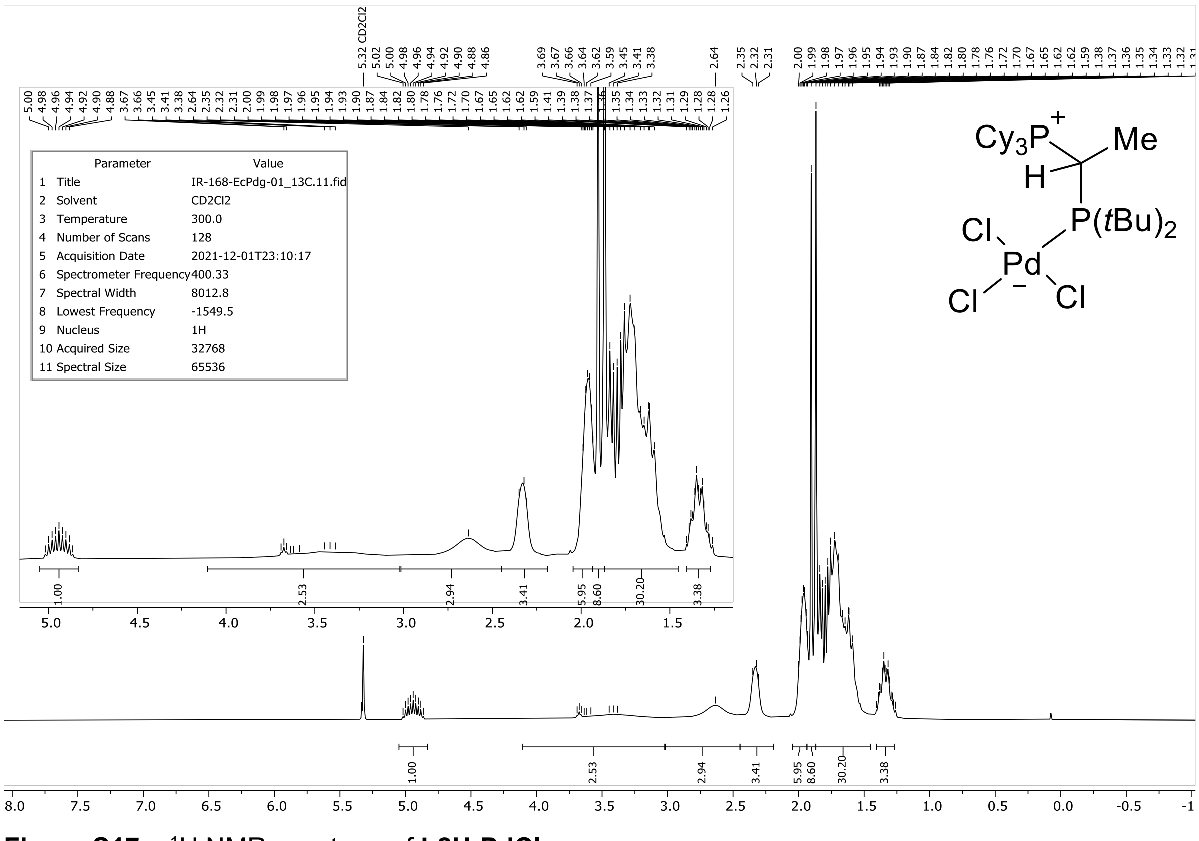

Figure S17a. ^{1}H NMR spectrum of **L2H·PdCl$_3$**.

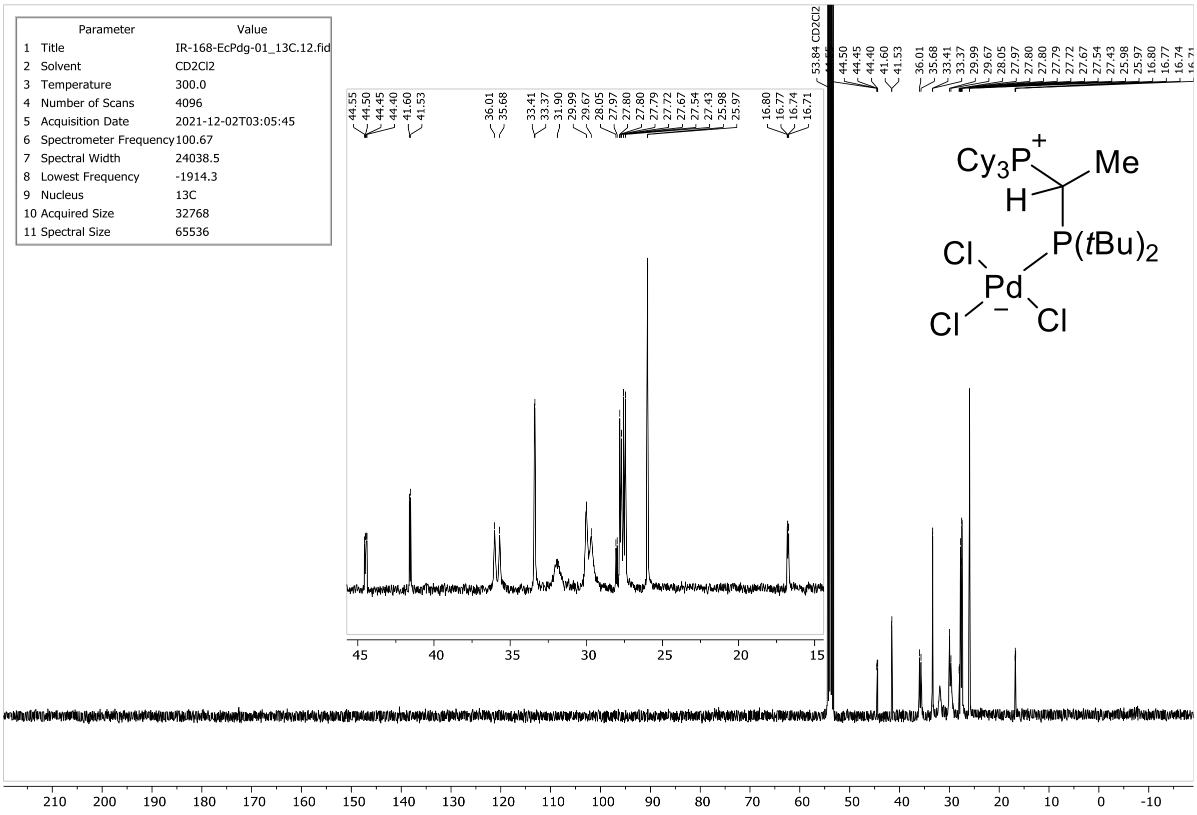

Figure S17b. $^{13}C\{^{1}H\}$ NMR spectrum of **L2H·PdCl$_3$**.

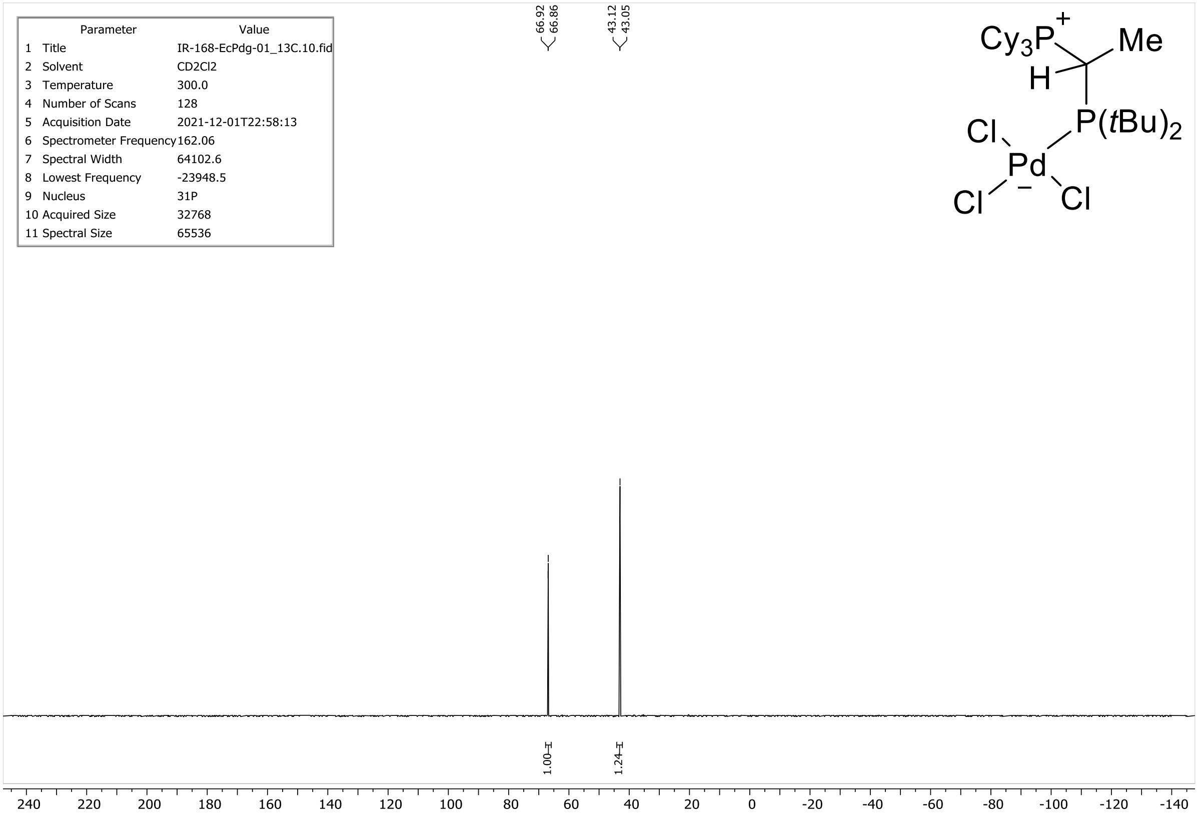

Figure S17c. $^{31}P\{^{1}H\}$ NMR spectrum of **L2H·PdCl$_3$**.

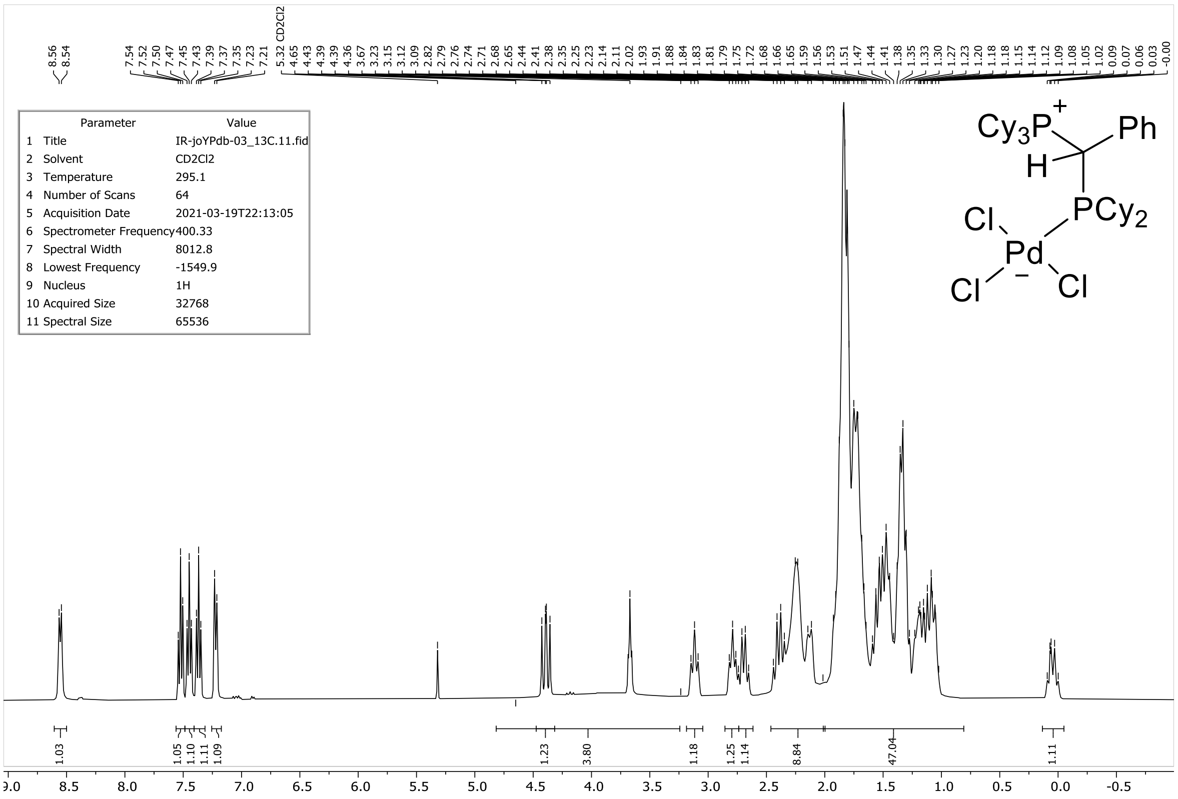

Figure S18a. ^{1}H NMR spectrum of **L3H·PdCl$_3$**.

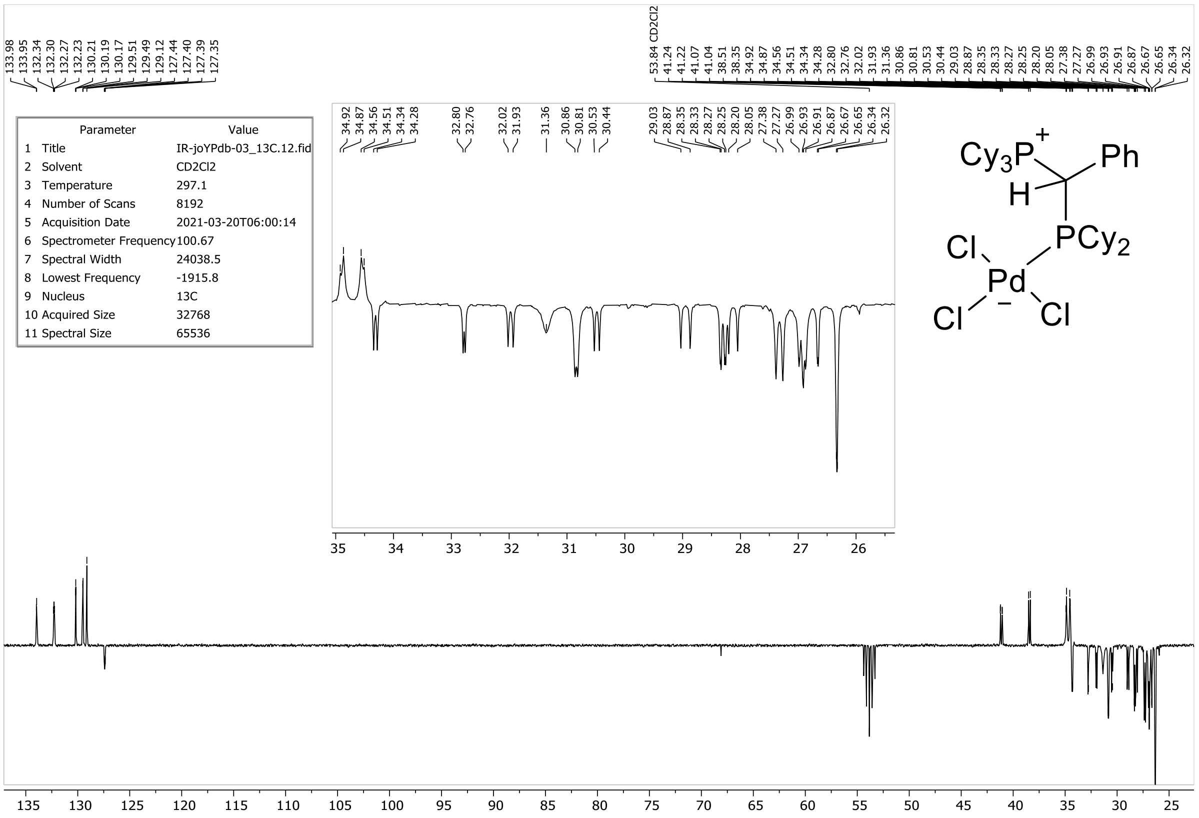

Figure S18b. $^{13}C\{^{1}H\}$ APT-NMR spectrum of **L3H·PdCl$_3$**.

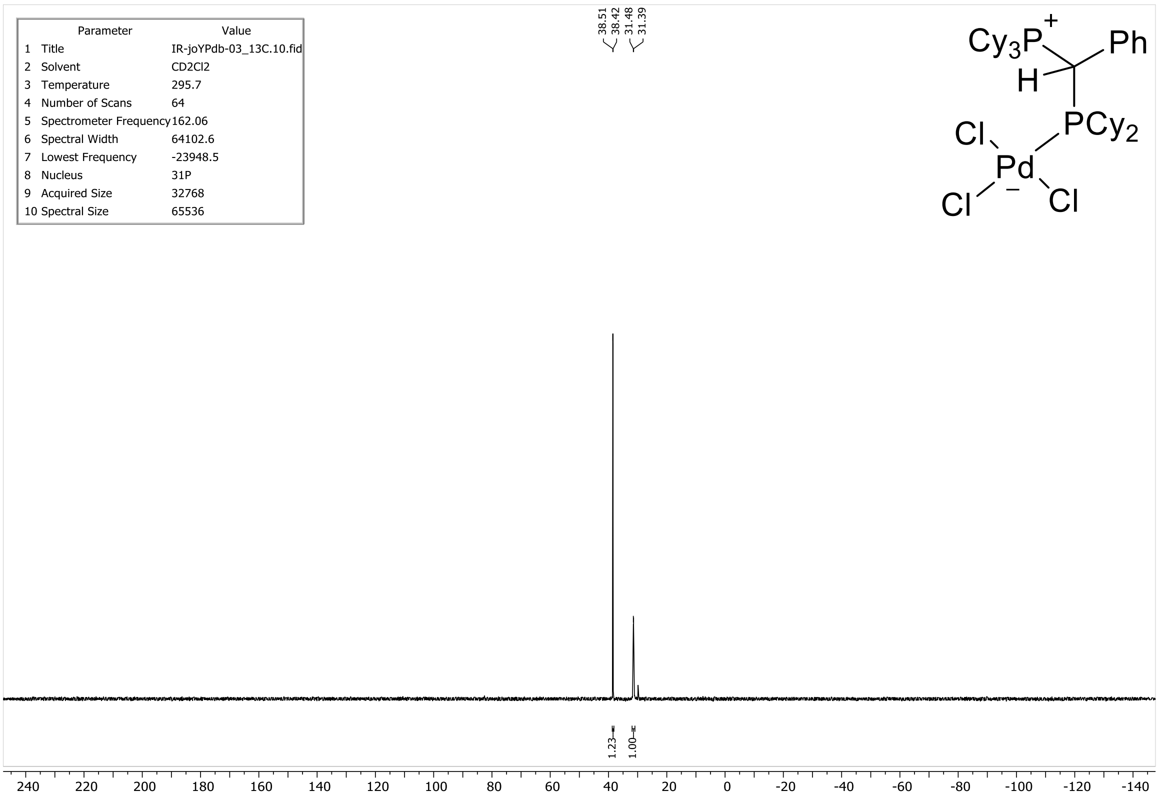

Figure S18c. $^{31}P\{^{1}H\}$ NMR spectrum of **L3H·PdCl₃**.

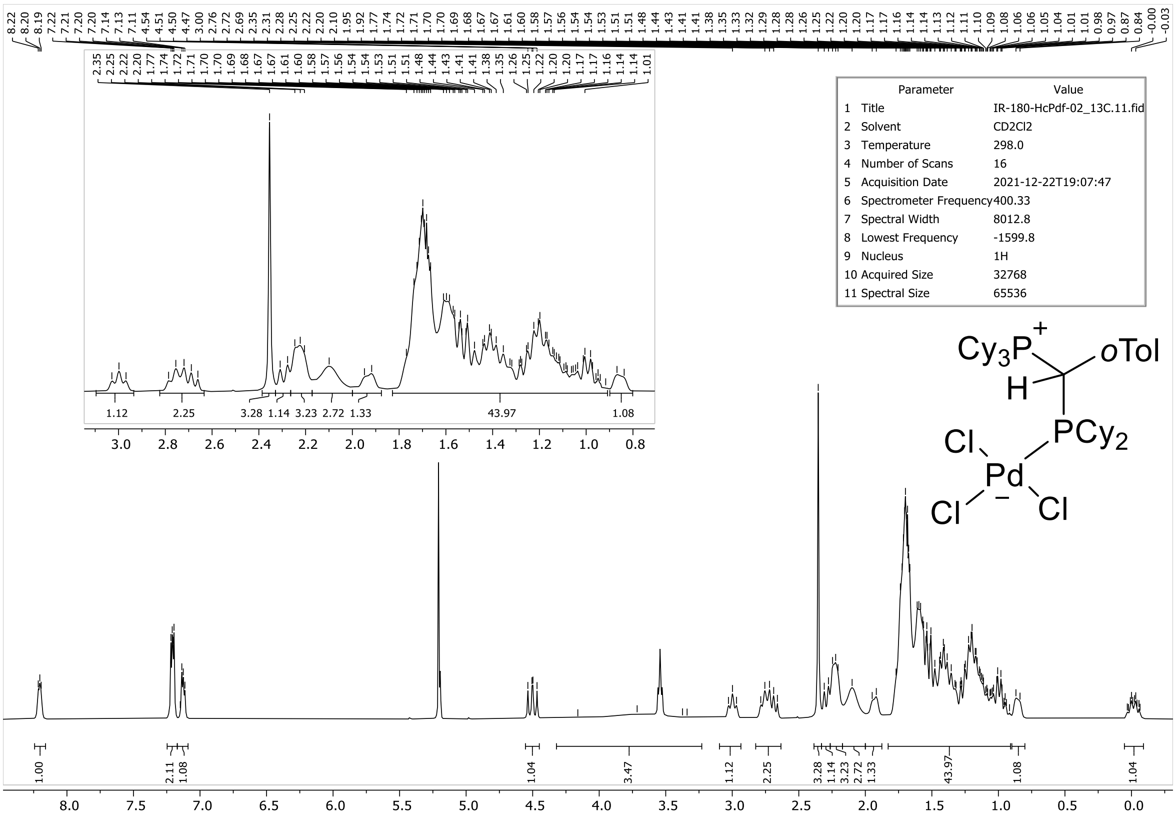

Figure S19a. ^{1}H NMR spectrum of **L4H·PdCl₃**.

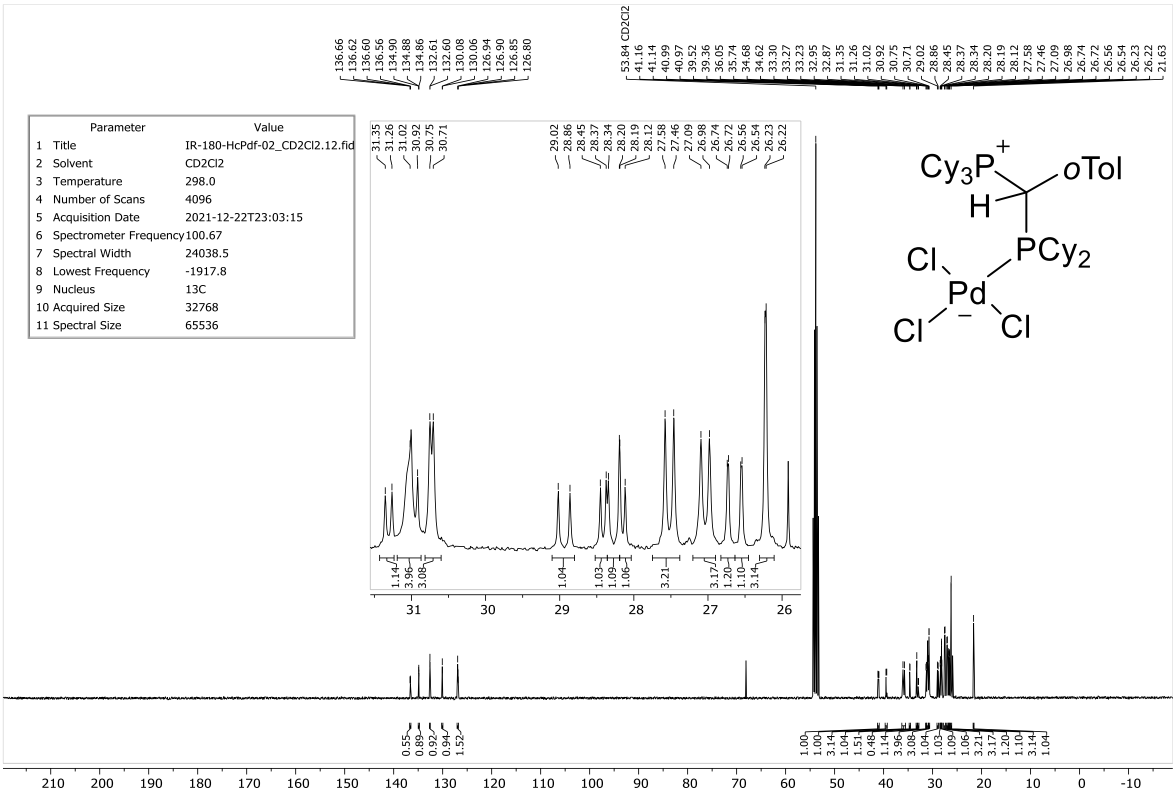

Figure S19b. $^{13}C\{^1H\}$ NMR spectrum of **L4H·PdCl₃**.

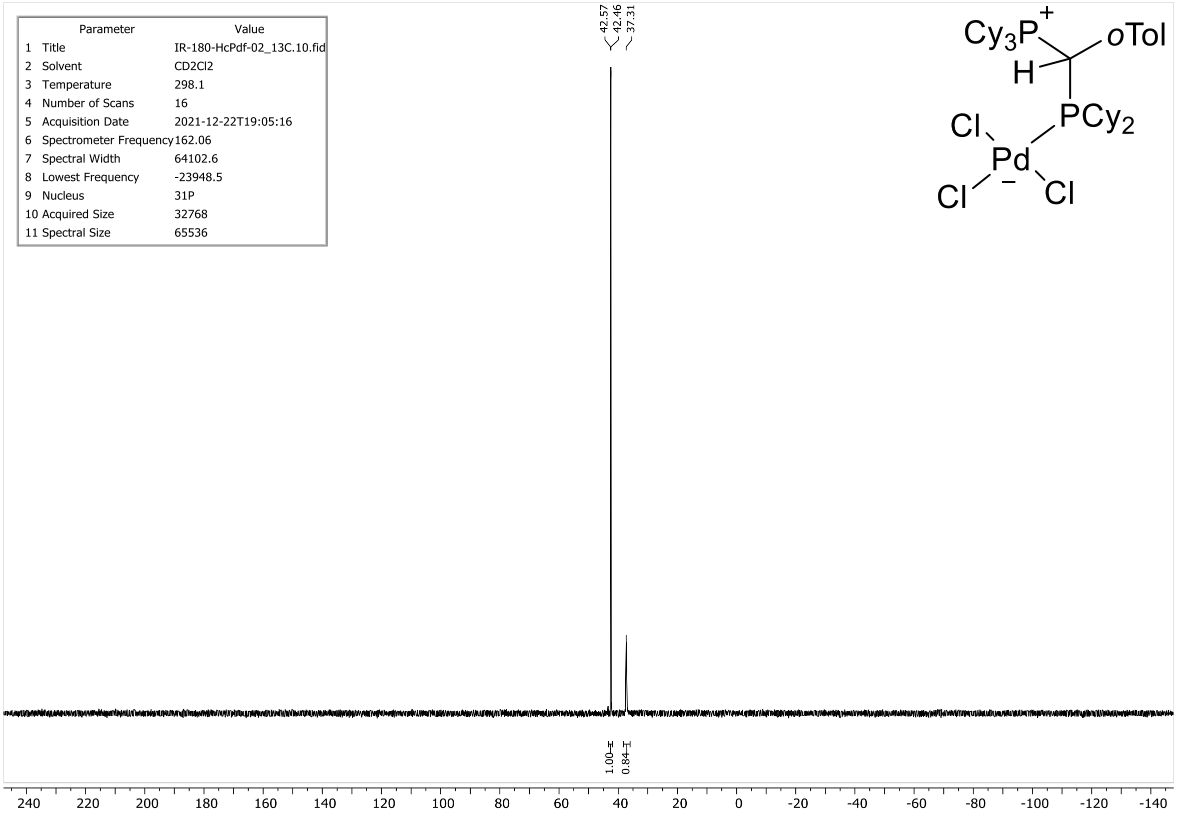

Figure S19c. $^{31}P\{^1H\}$ NMR spectrum of **L4H·PdCl$_3$**.

3.2 NMR Spectra of the Isolated Products from Catalysis

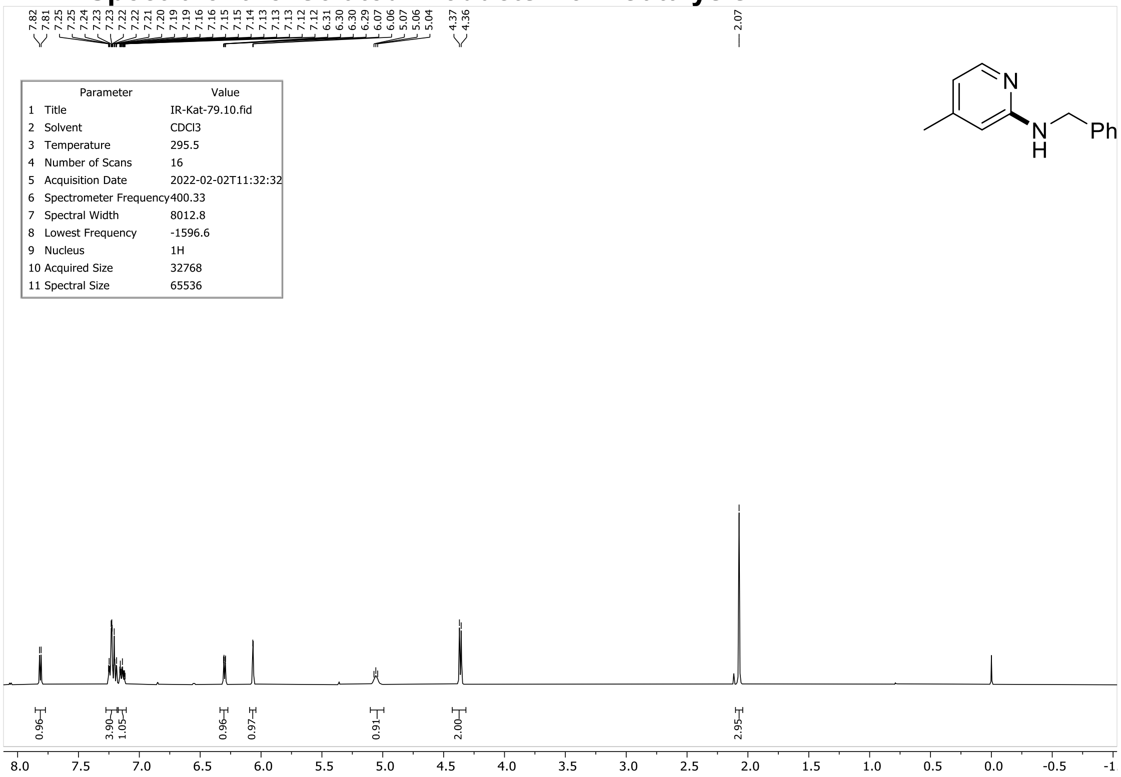

Figure S20. ^{1}H NMR spectrum of **Aa**.

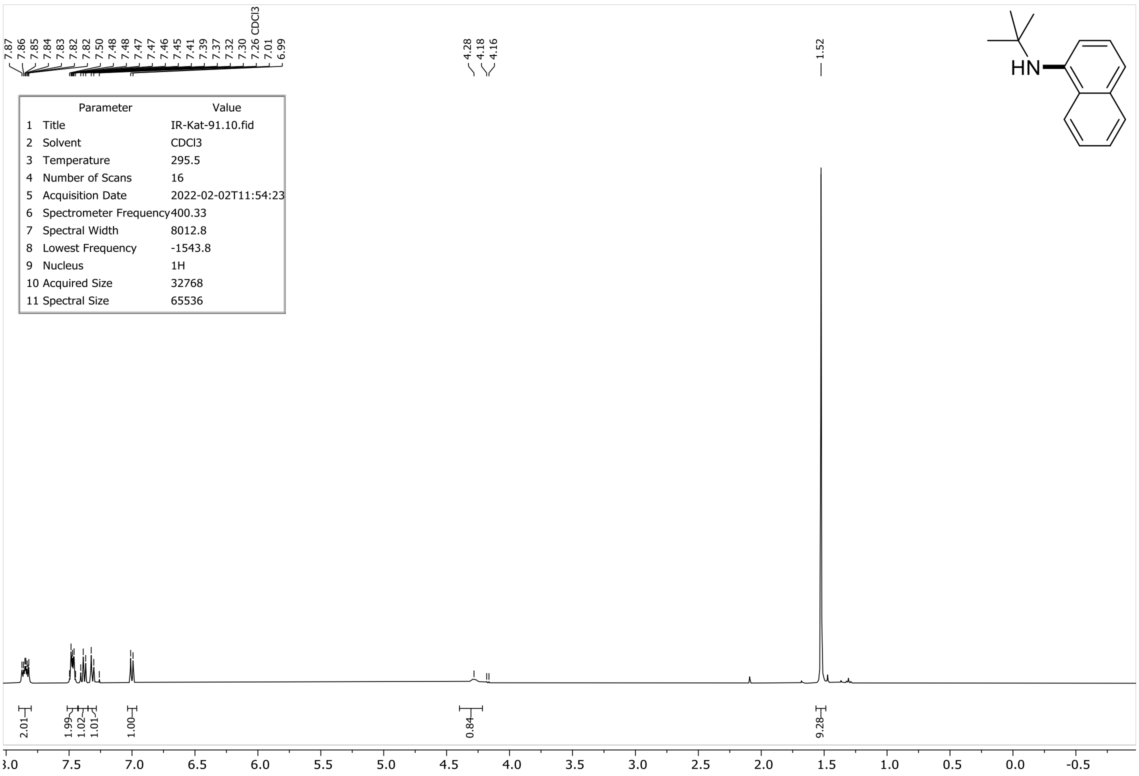

Figure S21. ^{1}H NMR spectrum of **Ab**.

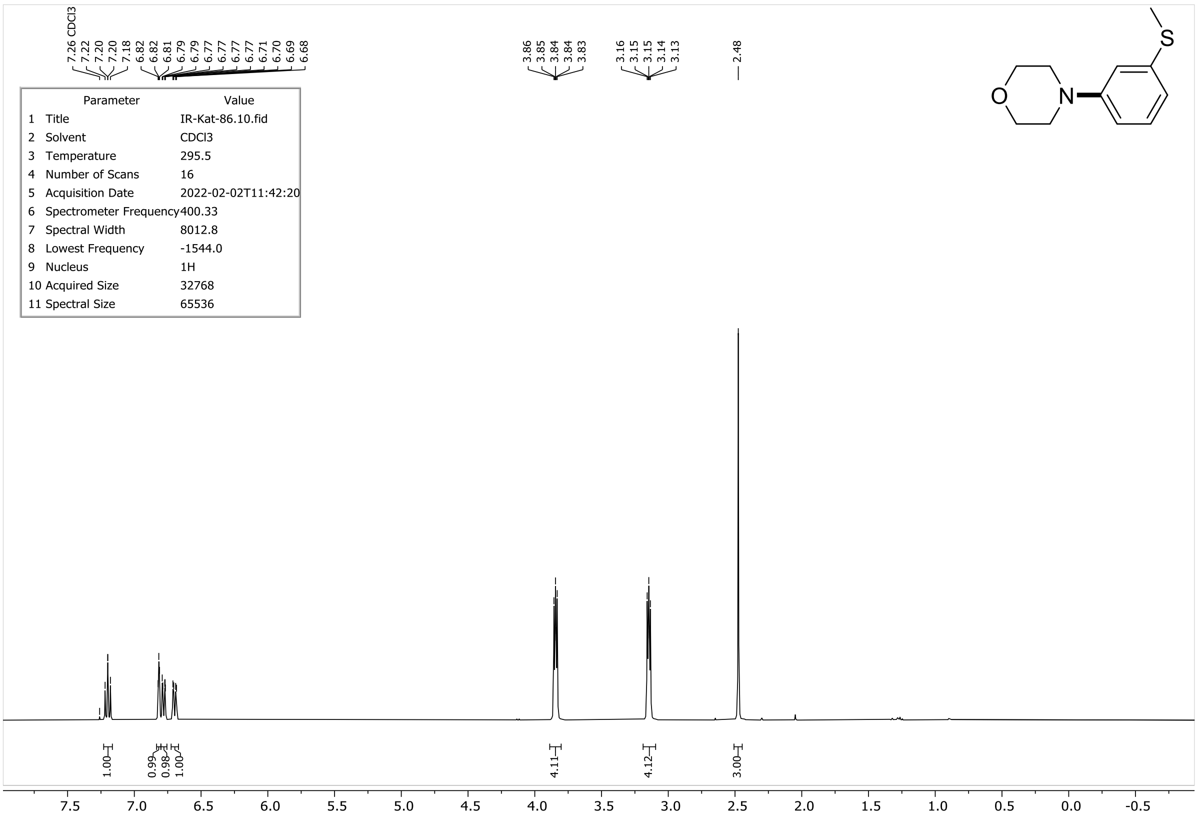

Figure S22. ^{1}H NMR spectrum of **Ac**.

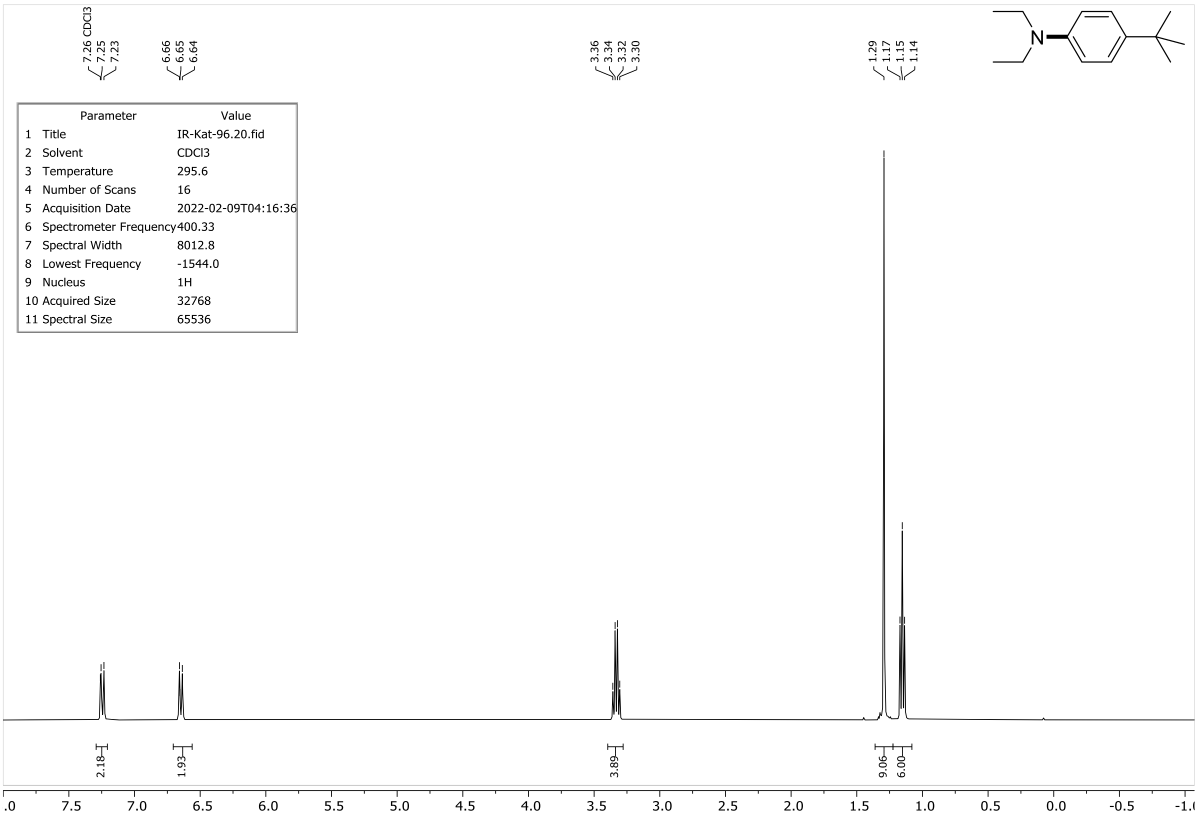

Figure S23. ^{1}H NMR spectrum of **Ad**.

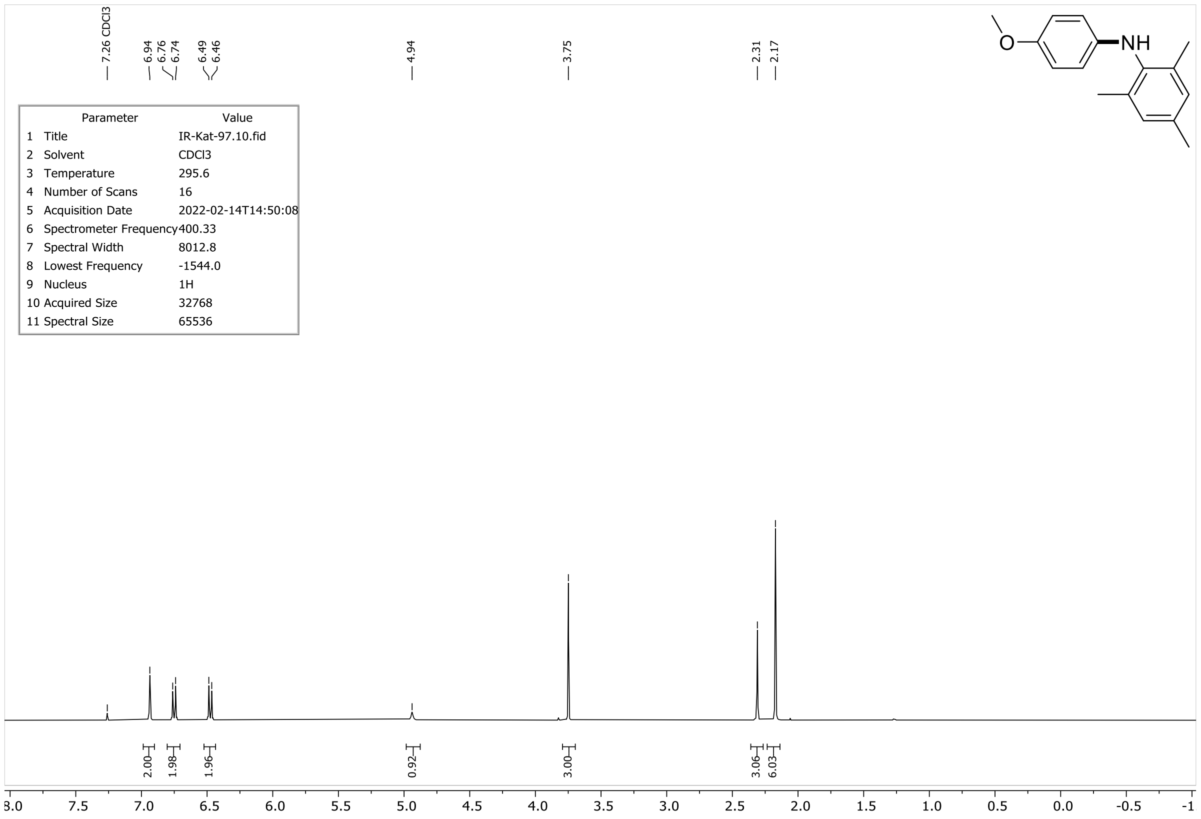

Figure S24. ^{1}H NMR spectrum of **Ae**.

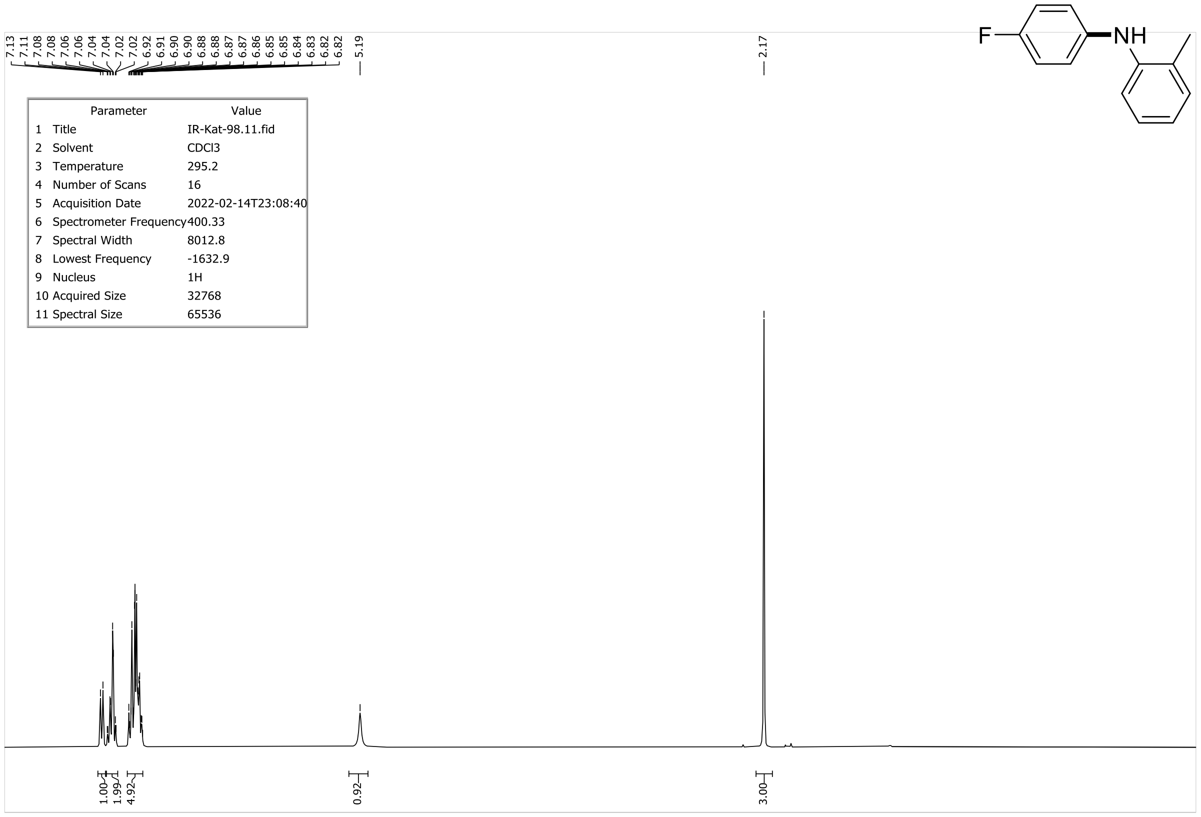

Figure S25a. ^{1}H NMR spectrum of **Af**.

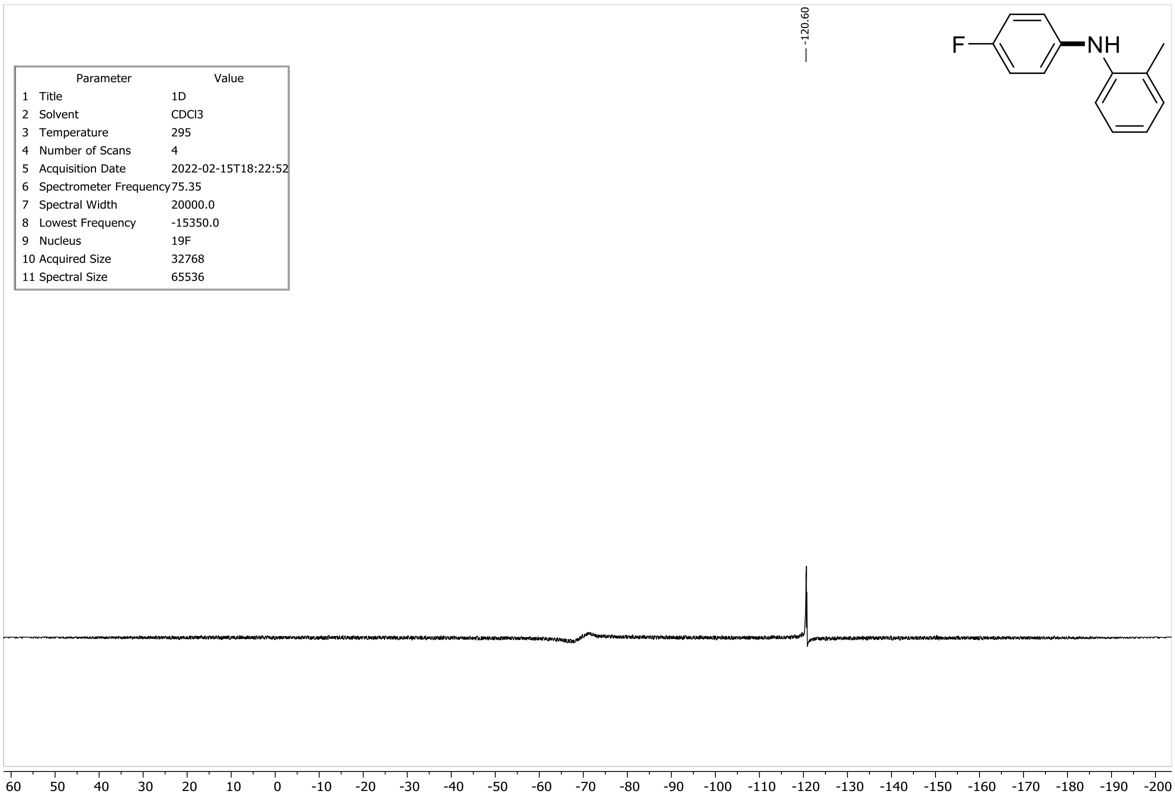

Figure S25b. $^{19}F\{^{1}H\}$ NMR spectrum of **Af**.

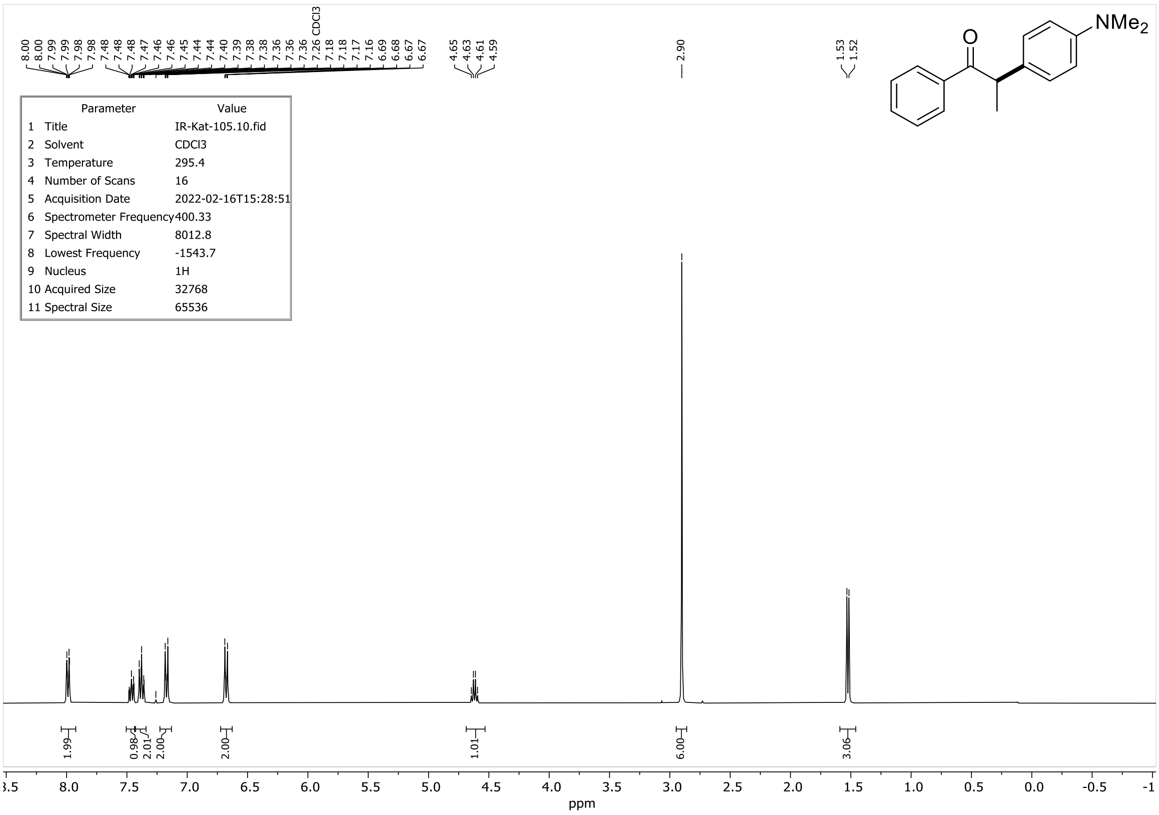

Figure S26. ^{1}H NMR spectrum of **Ba**.

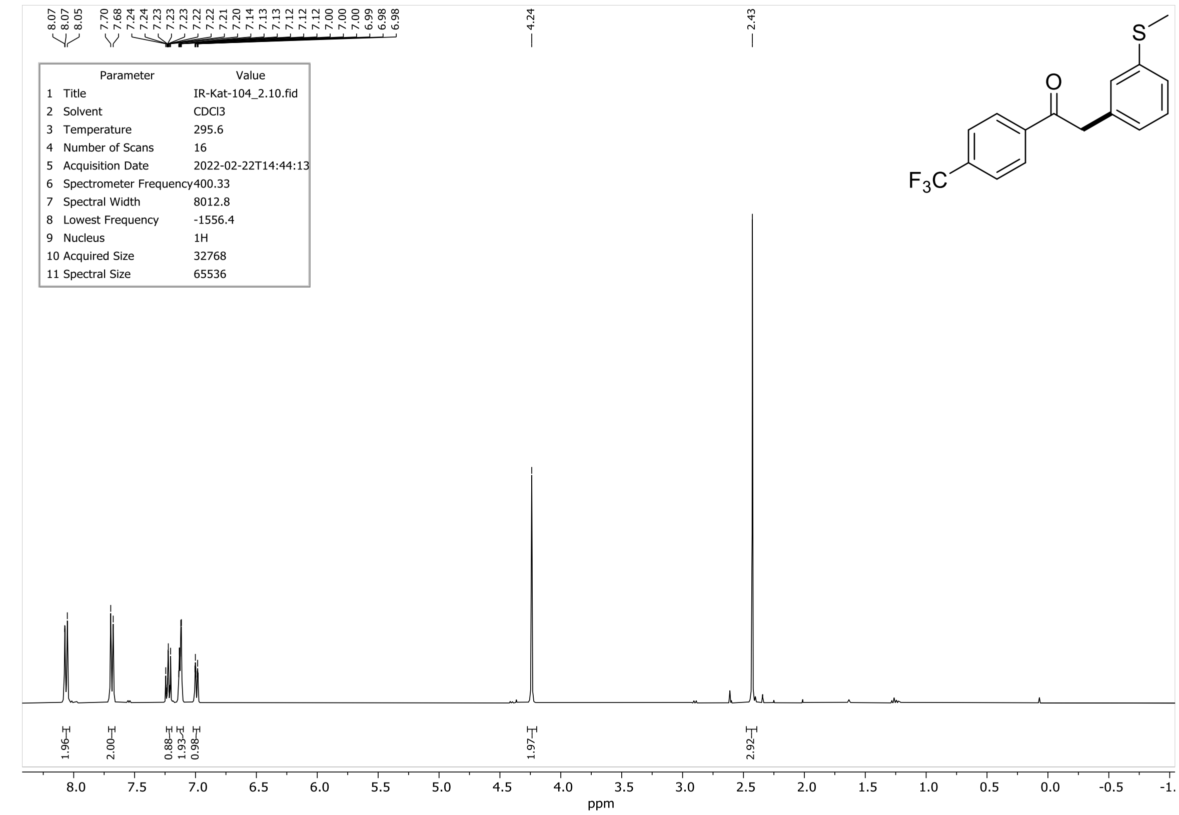

Figure S27a. ^{1}H NMR spectrum of **Bb**.

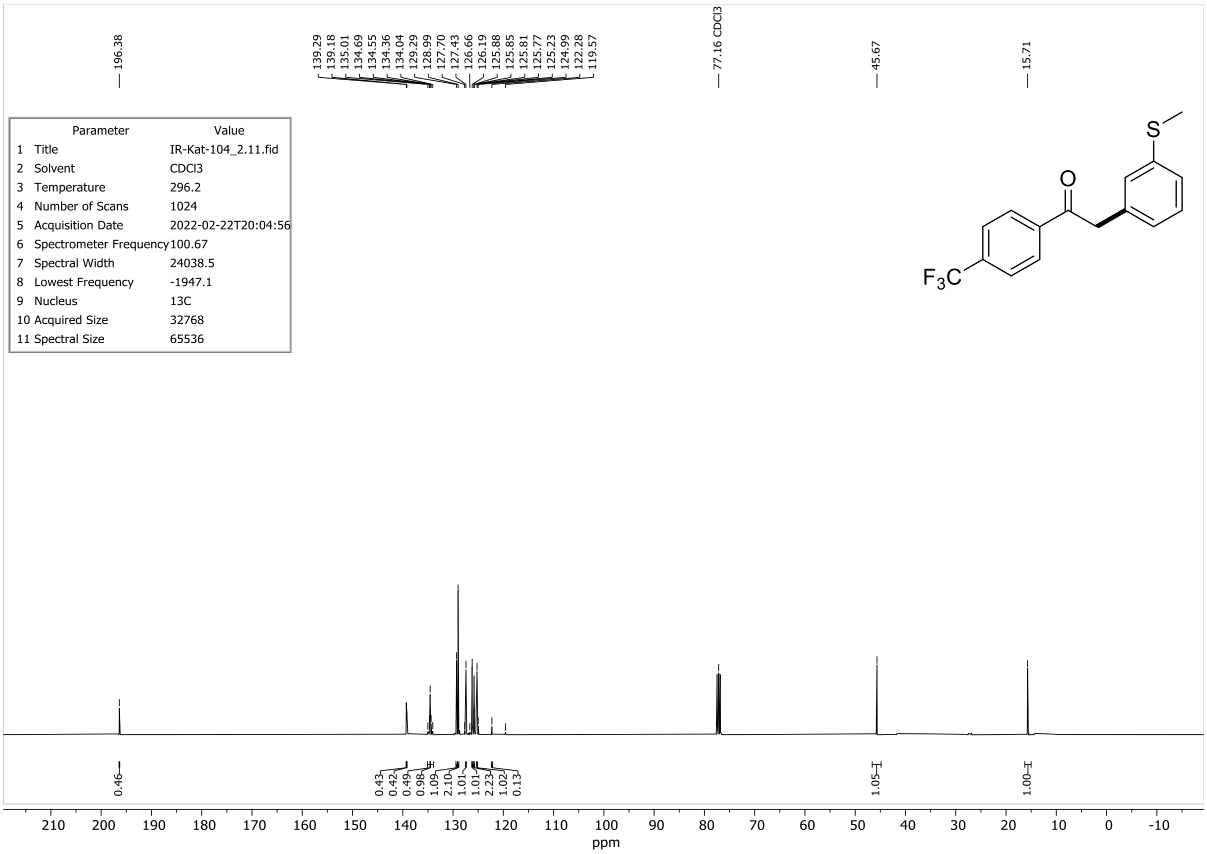

Figure S27b. $^{13}C\{^{1}H\}$ NMR spectrum of **Bb**.

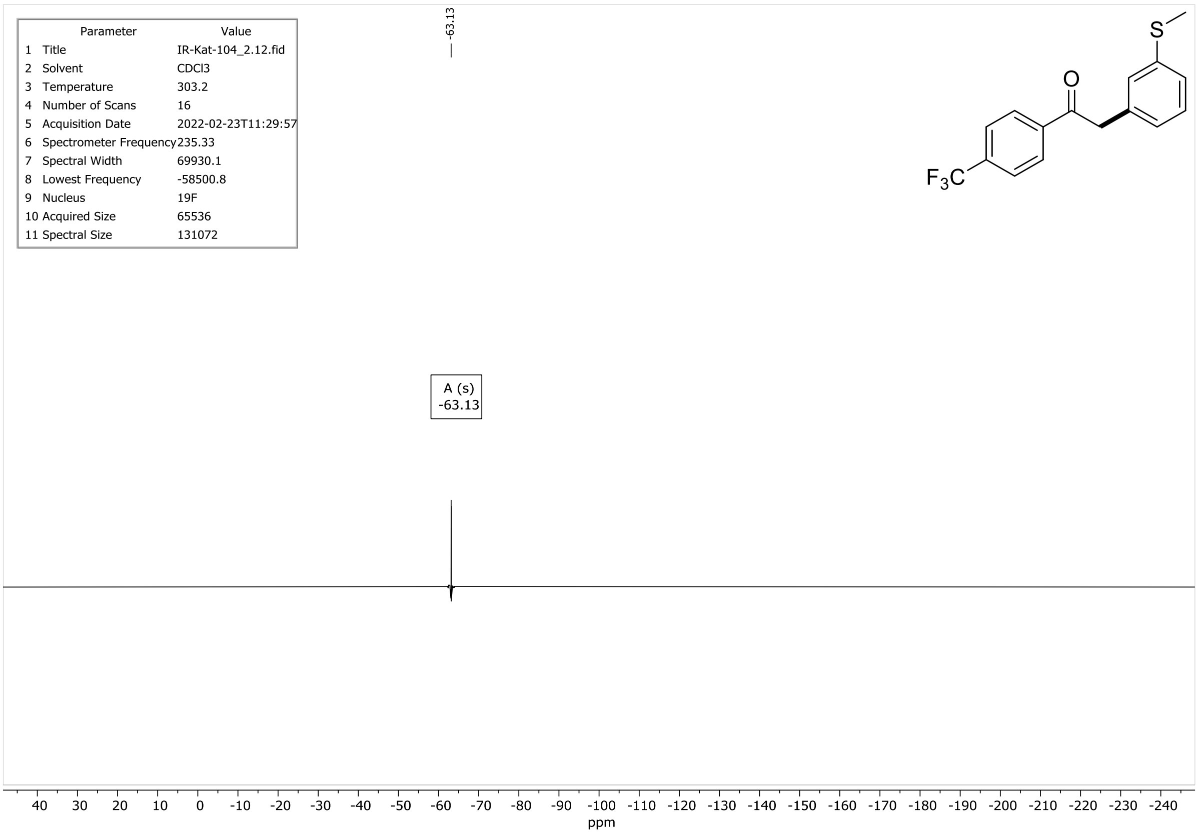

Figure S27c. ^{19}F{^{1}H} NMR spectrum of **Bb**.

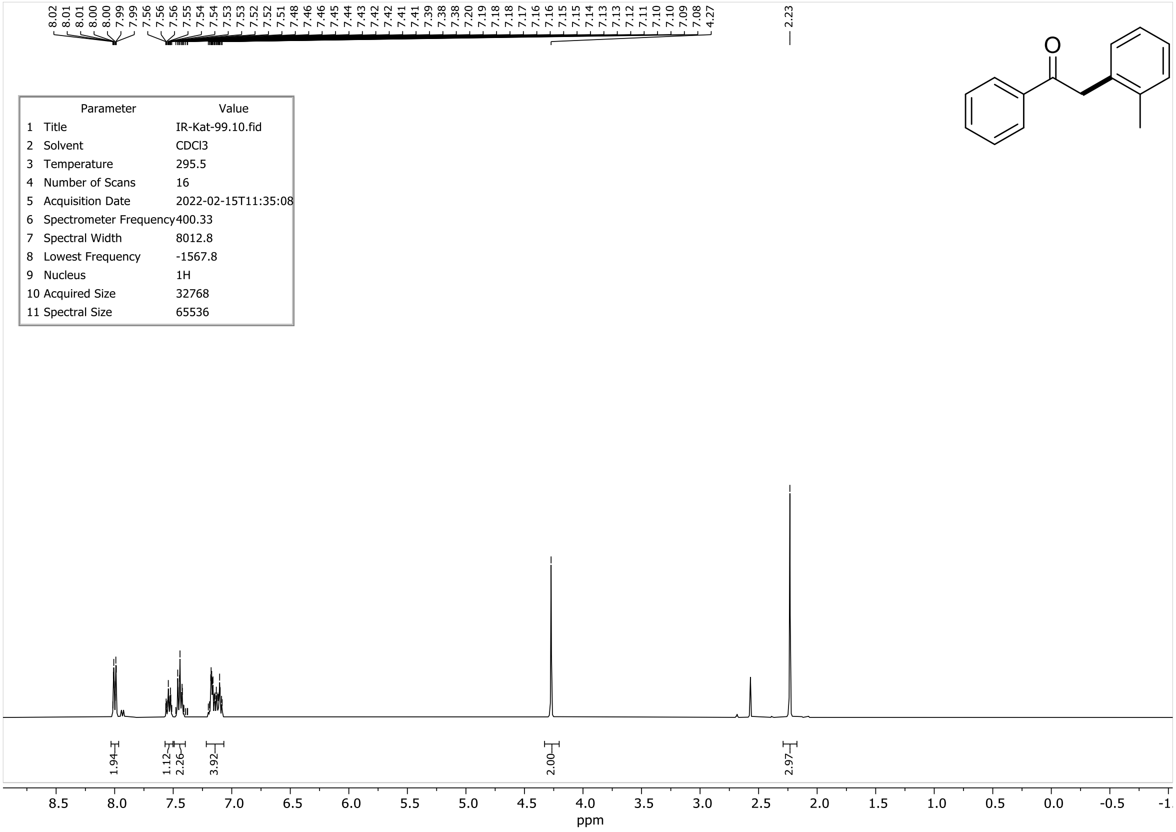

Figure S28. ^{1}H NMR spectrum of **Bc**.

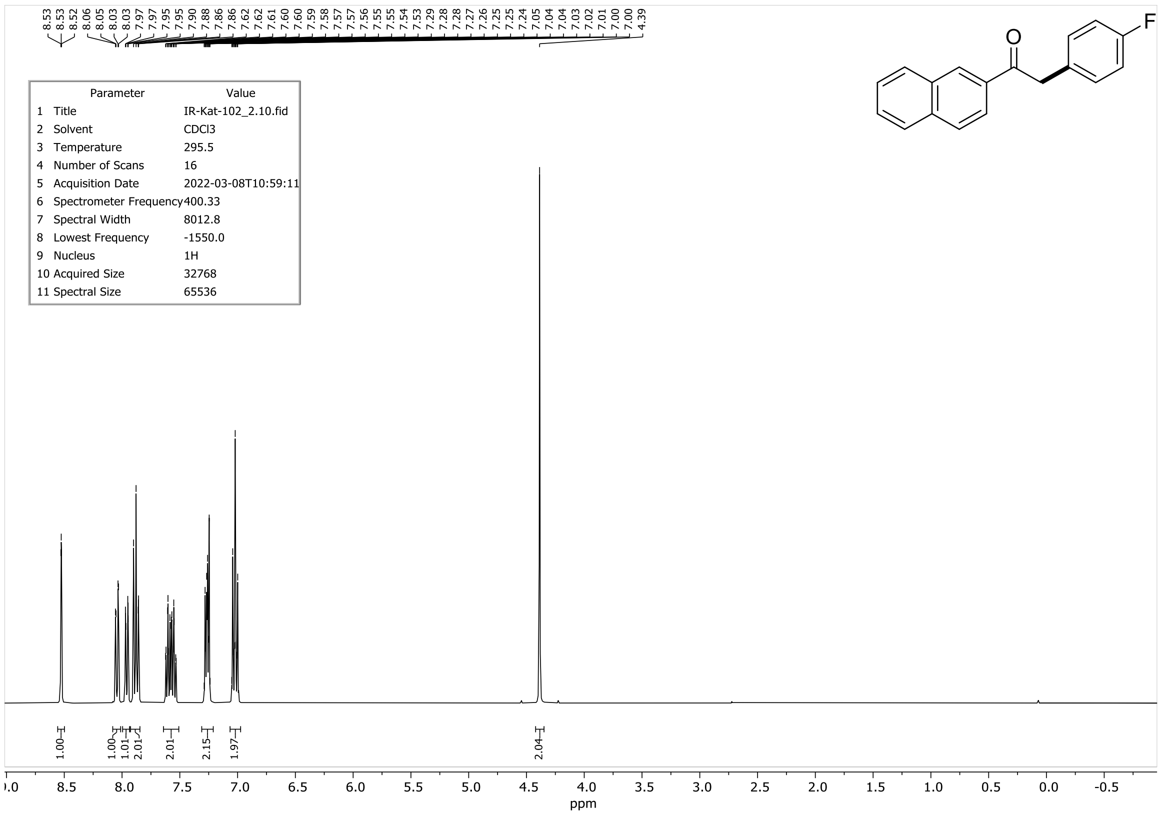

Figure S29a. ^{1}H NMR spectrum of **Bd**.

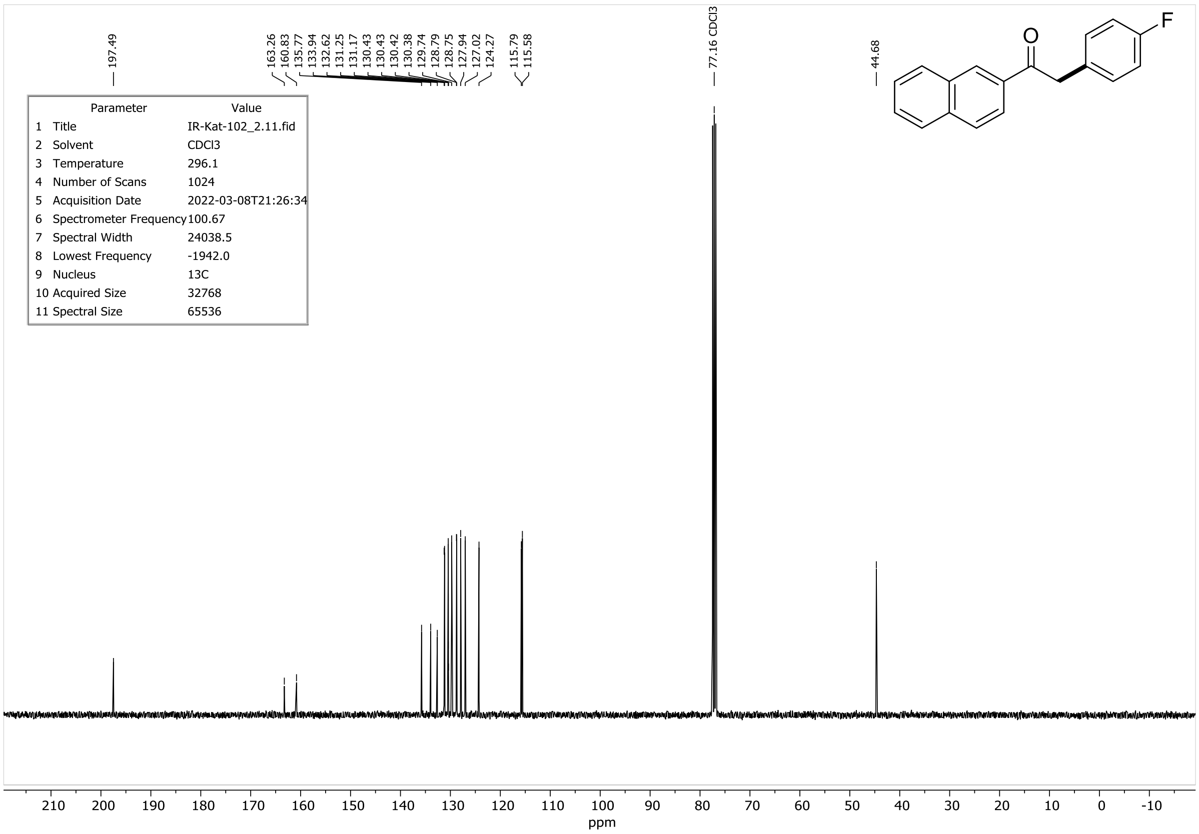

Figure S29b. $^{13}C\{^{1}H\}$ NMR spectrum of **Bd**.

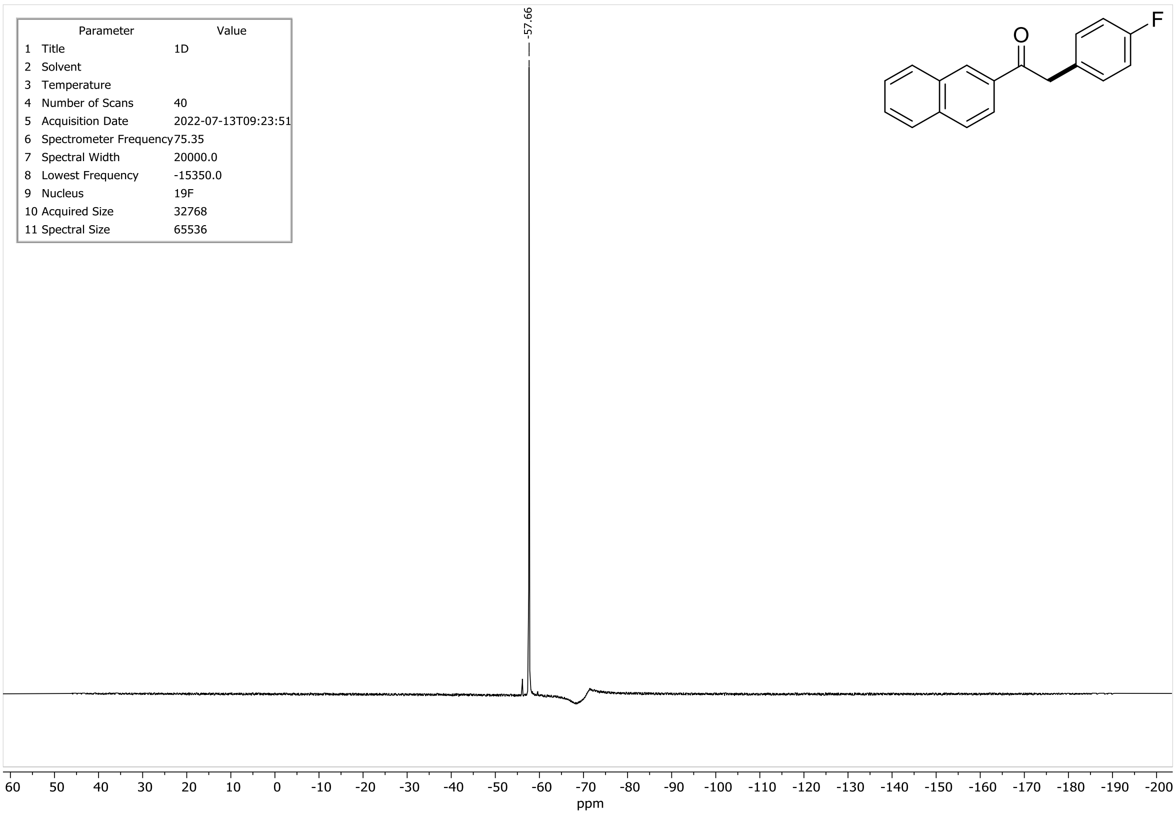

Figure S29c. $^{19}F\{^{1}H\}$ NMR spectrum of **Bd**.

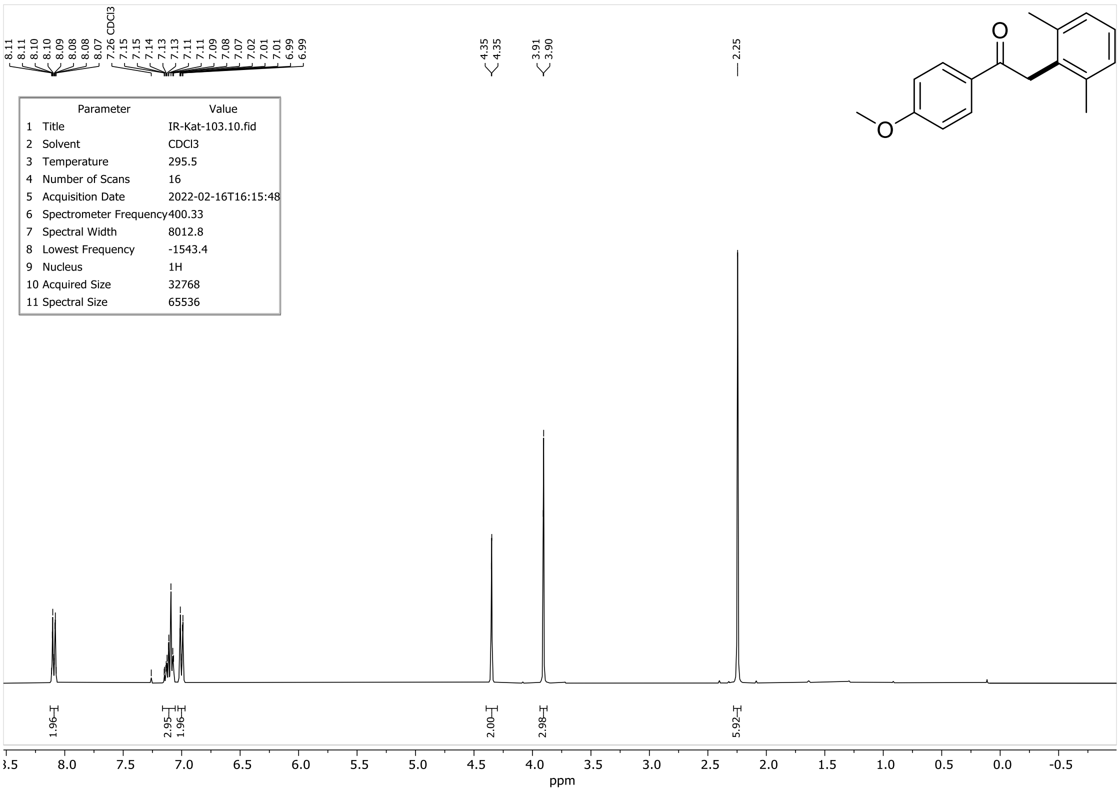

Figure S30. ^{1}H NMR spectrum of **Be**.

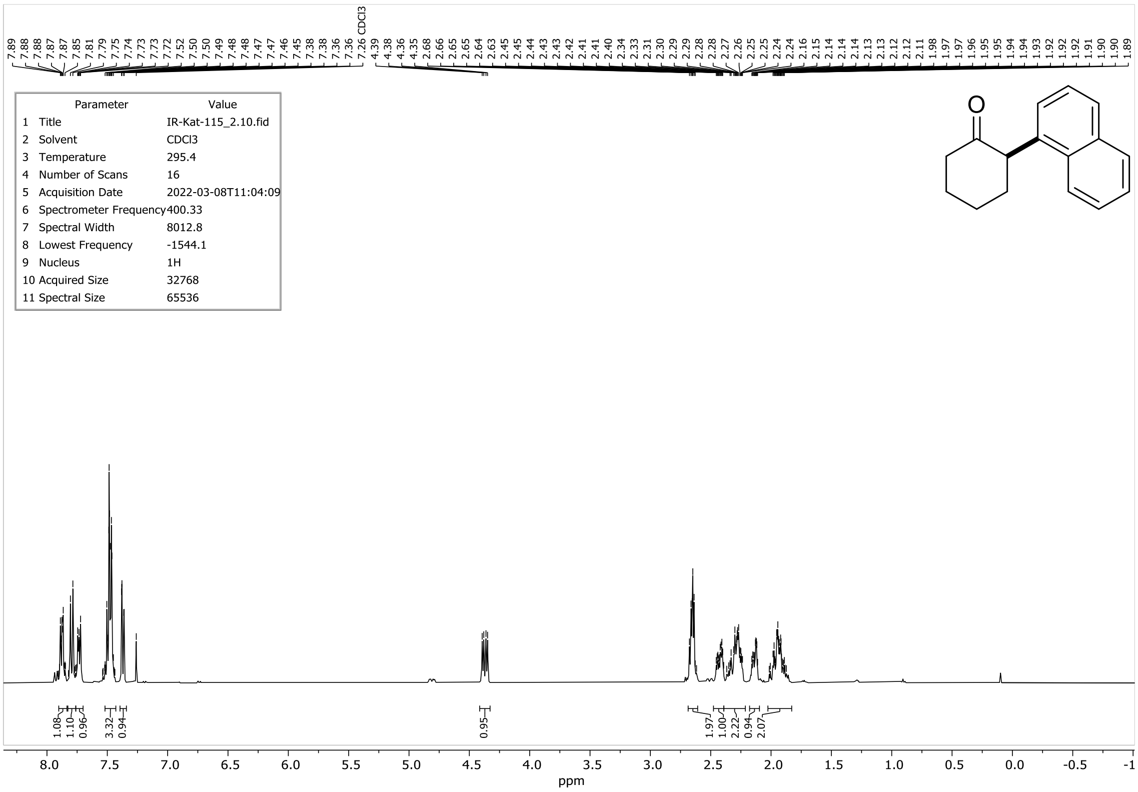

Figure S31. ^{1}H NMR spectrum of **Bf**.

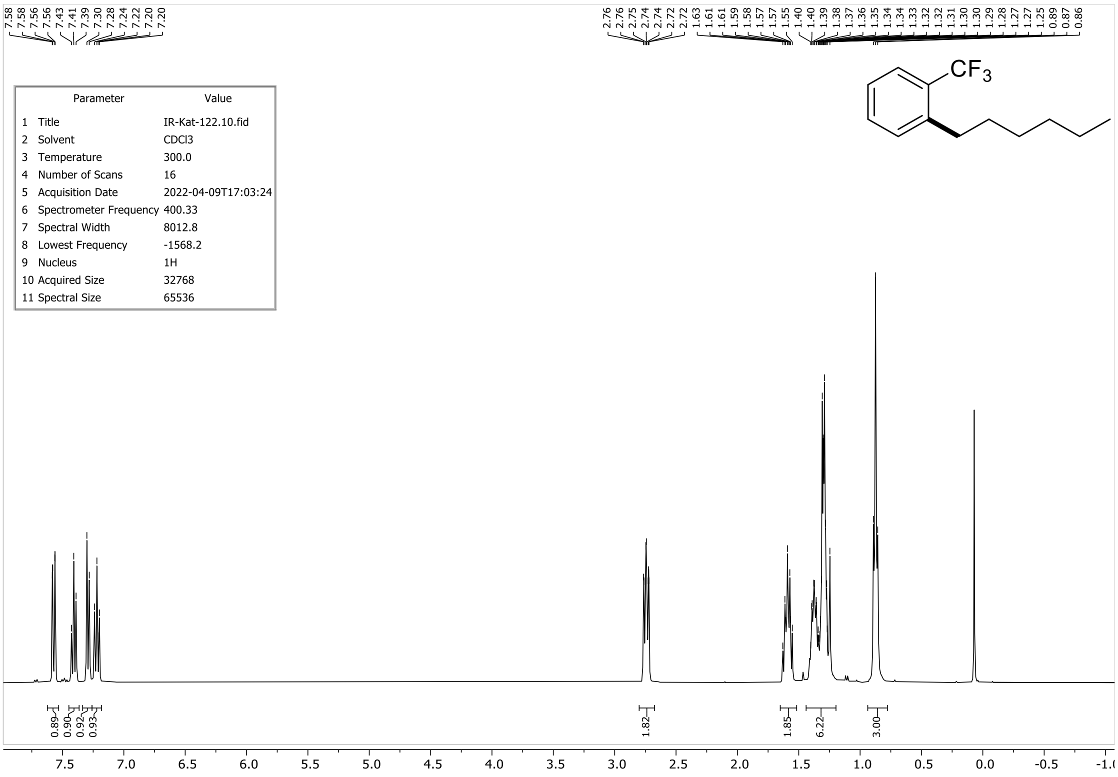

Figure S32a. ^{1}H NMR spectrum of **Ca**.

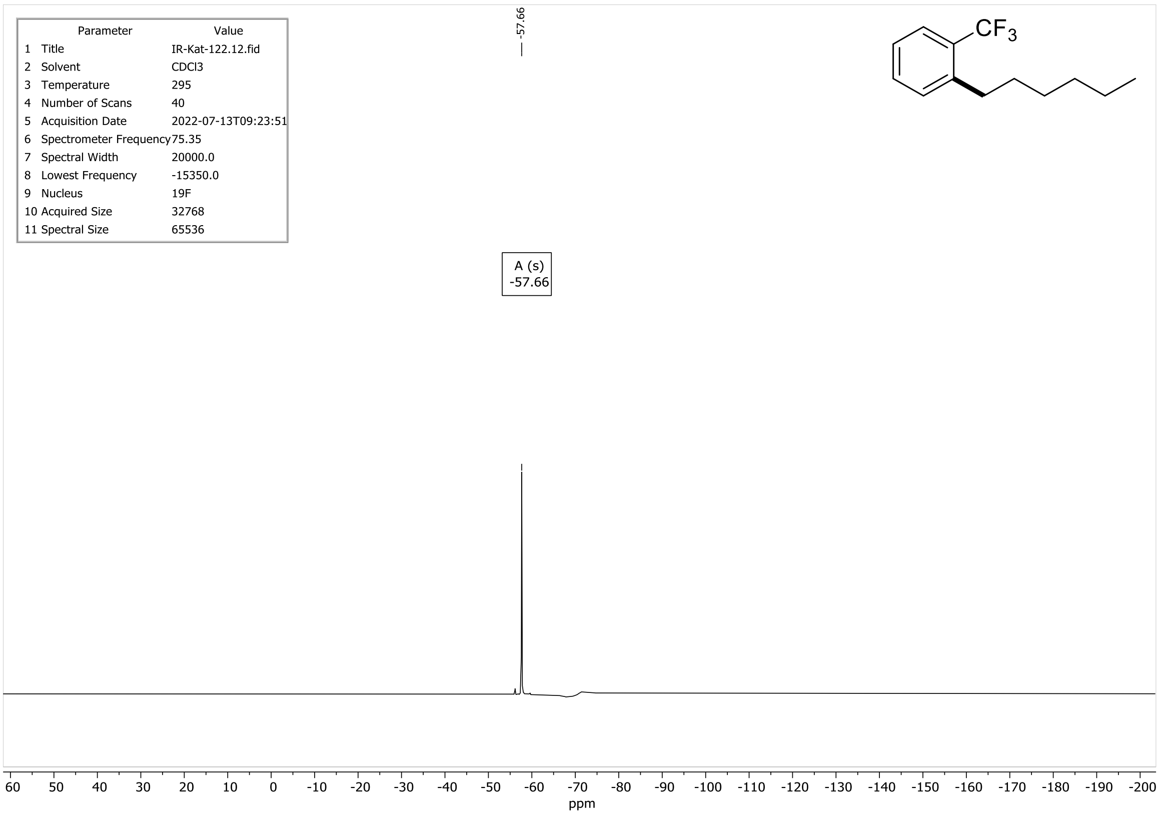

Figure S32b. $^{19}F\{^1H\}$ NMR spectrum of **Ca**.

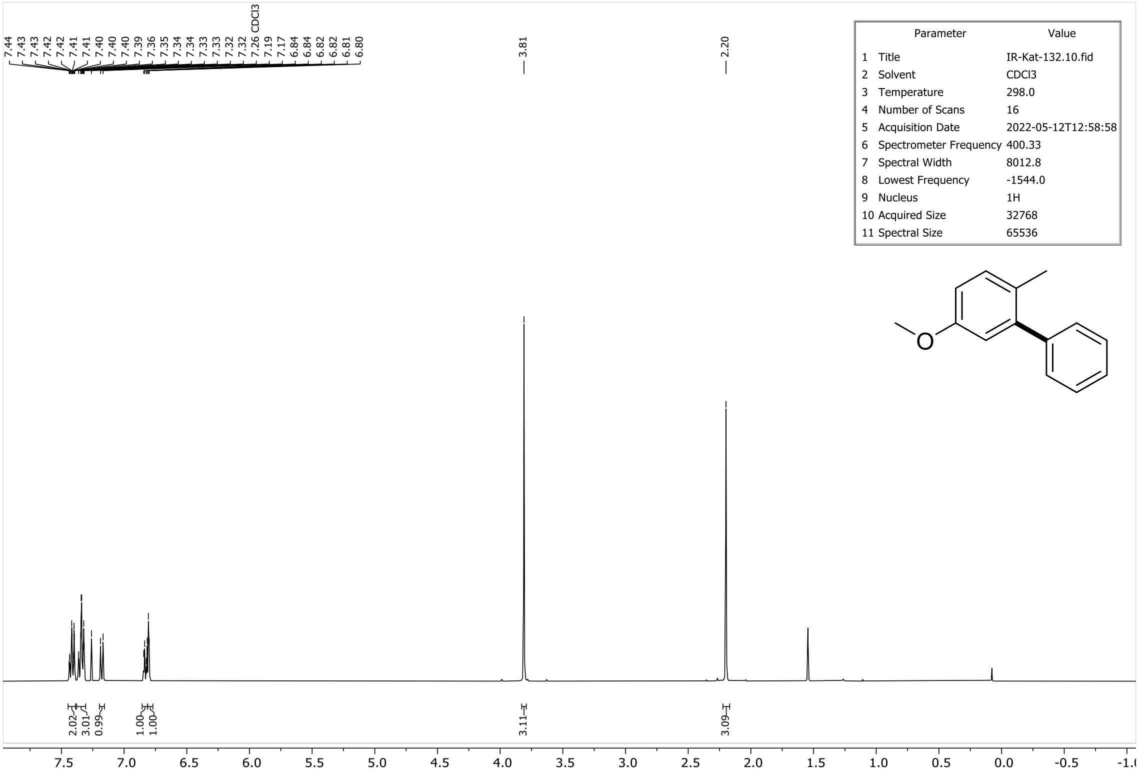

Figure S33a. ^1H NMR spectrum of **Cb**.

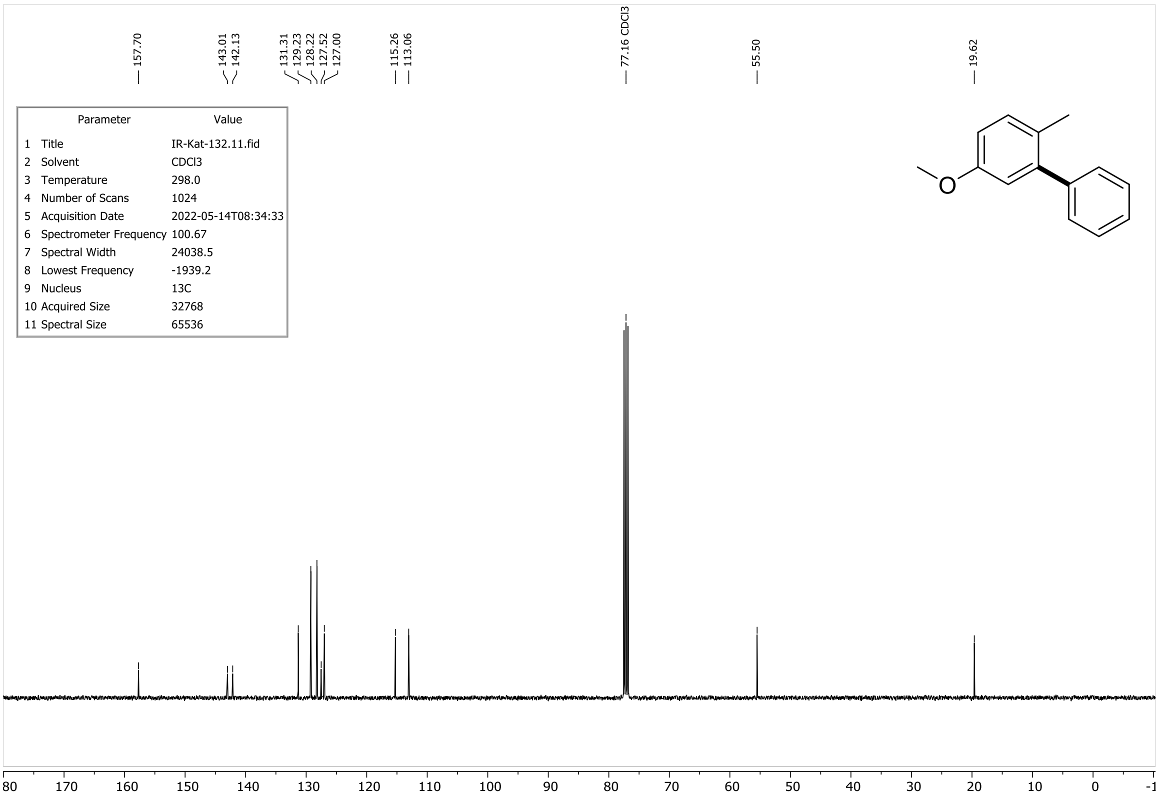

Figure S33b. $^{13}C\{^{1}H\}$ NMR spectrum of **Cb**.

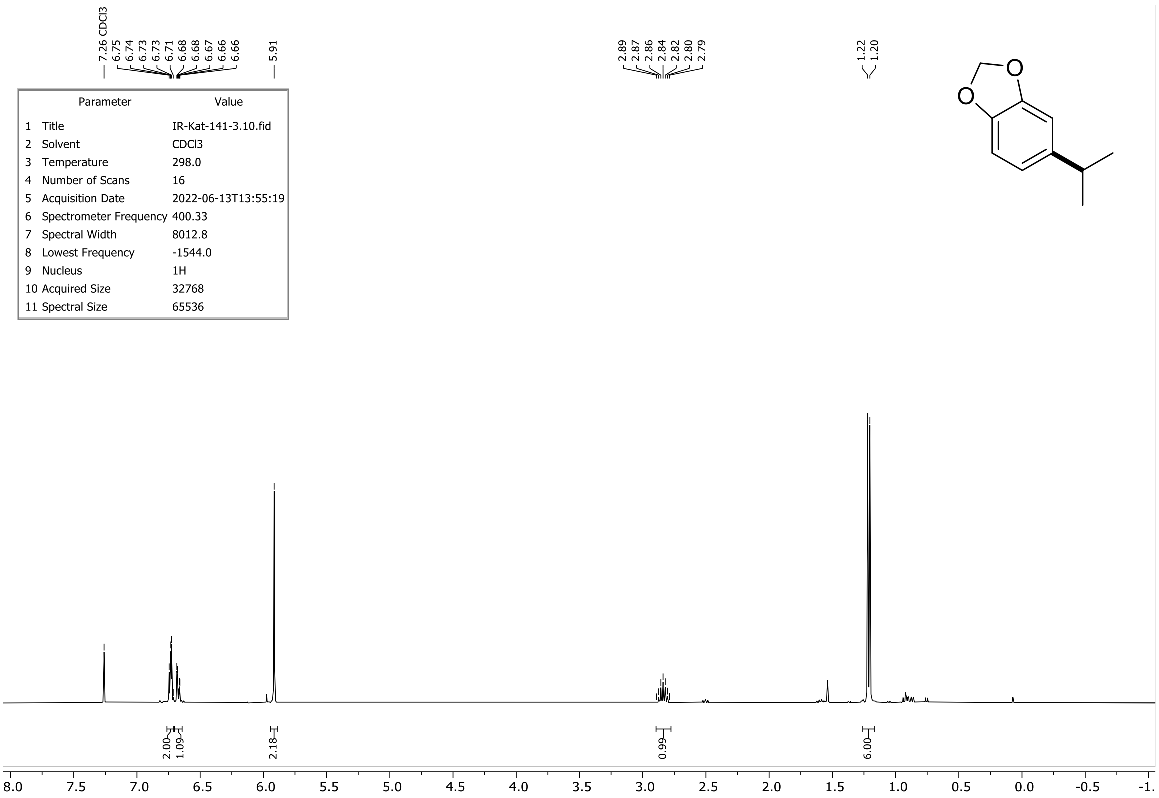

Figure S34. ^{1}H NMR spectrum of **Cc**.

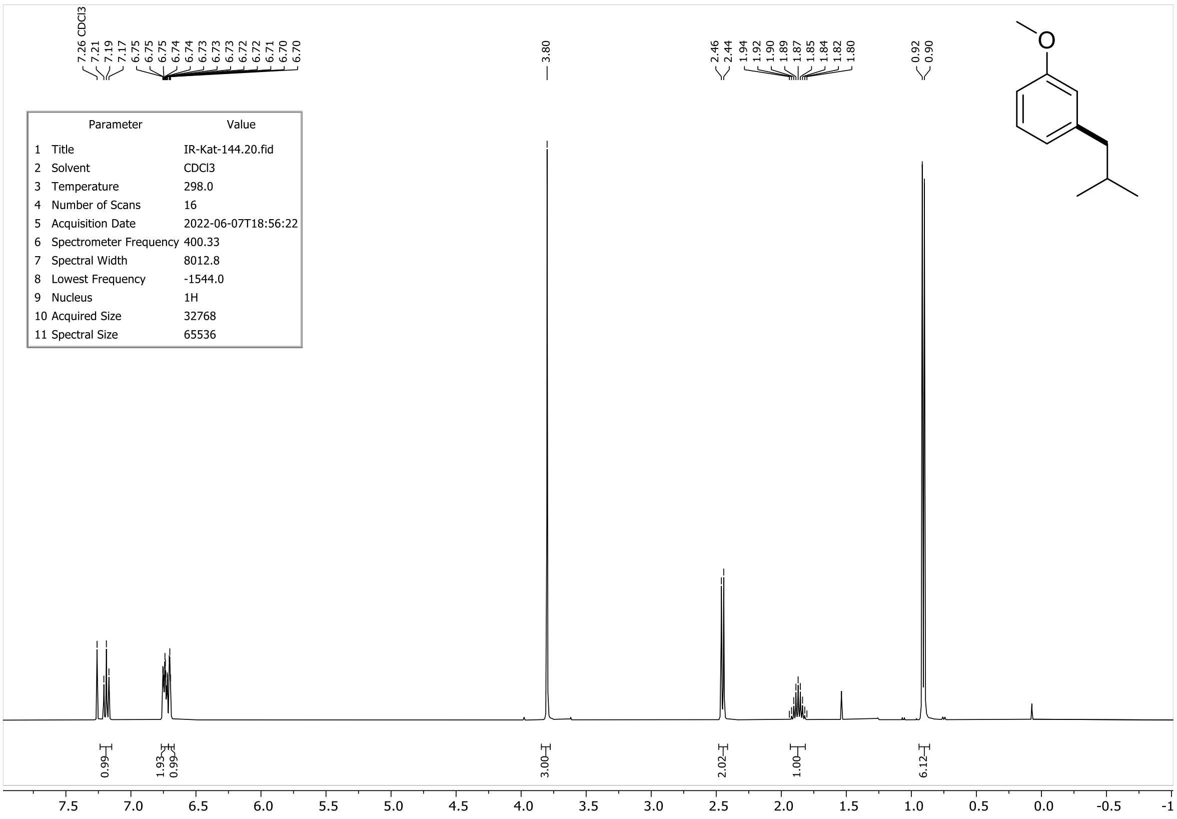

Figure S35. ^{1}H NMR spectrum of **Cd**.

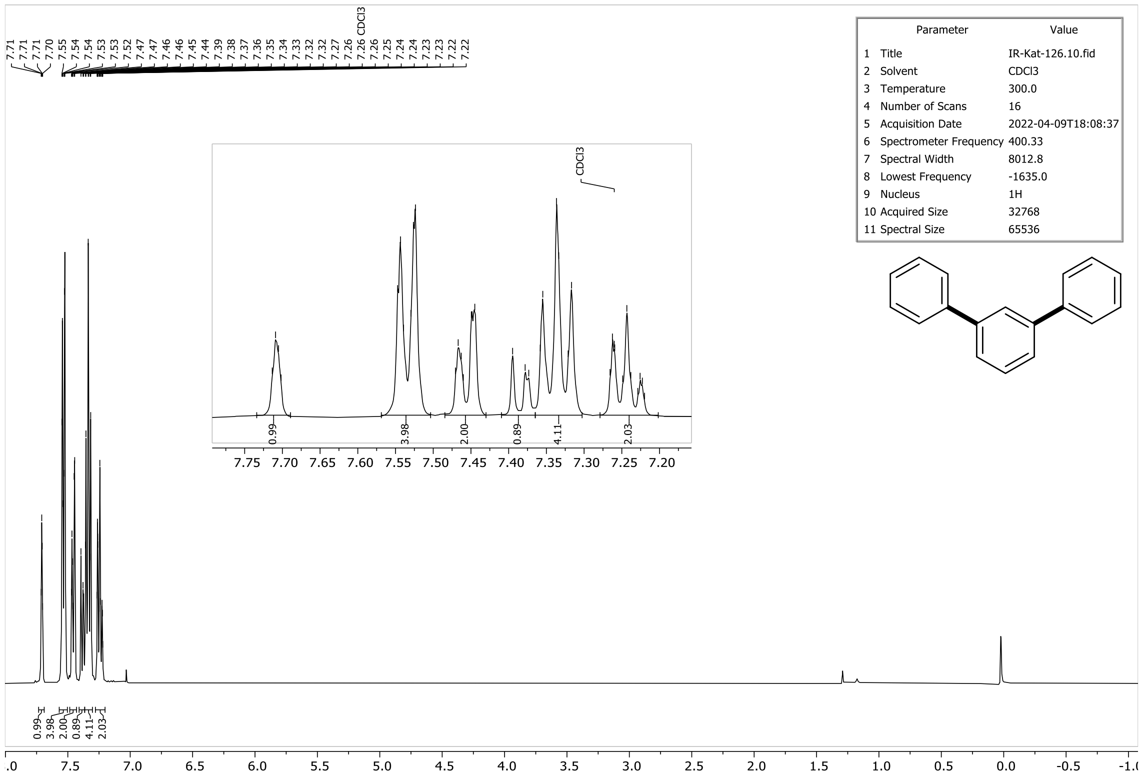

Figure S36. ^{1}H NMR spectrum of **Da**.

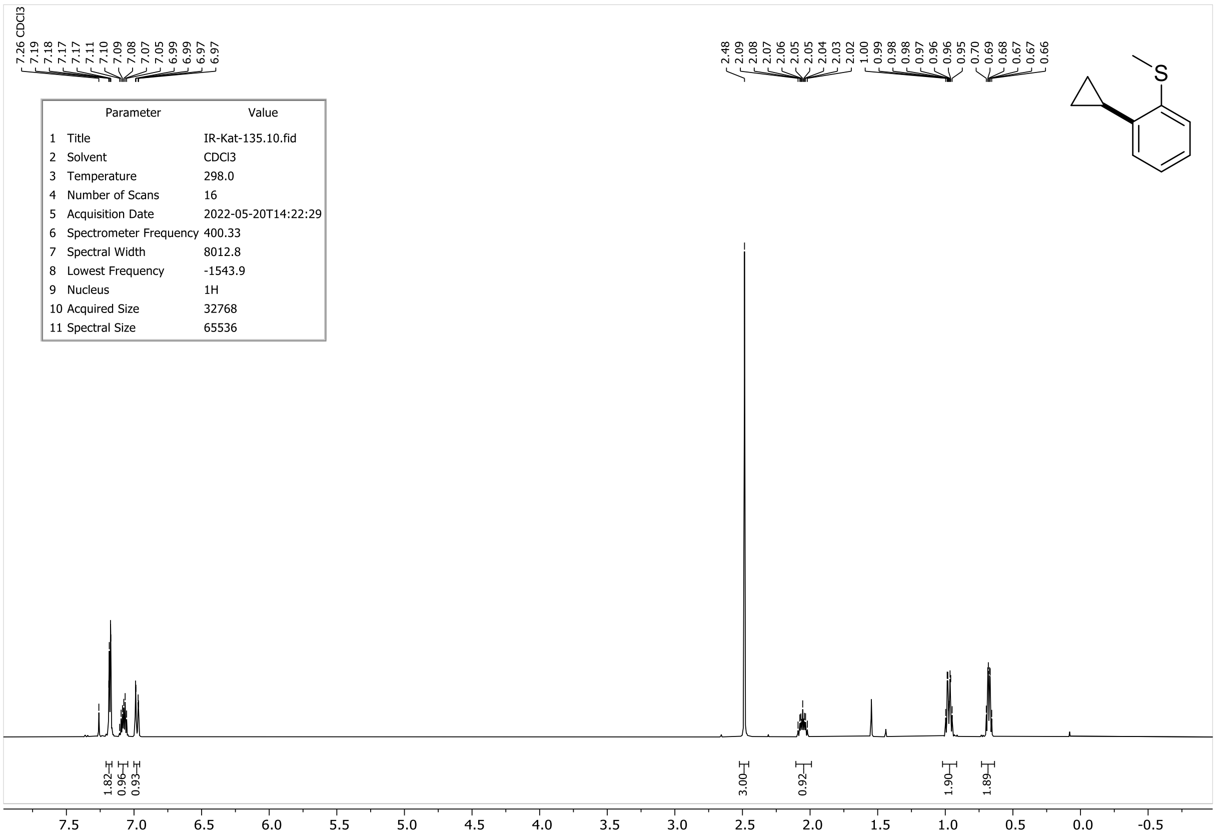

Figure S37a. ^{1}H NMR spectrum of **Db**.

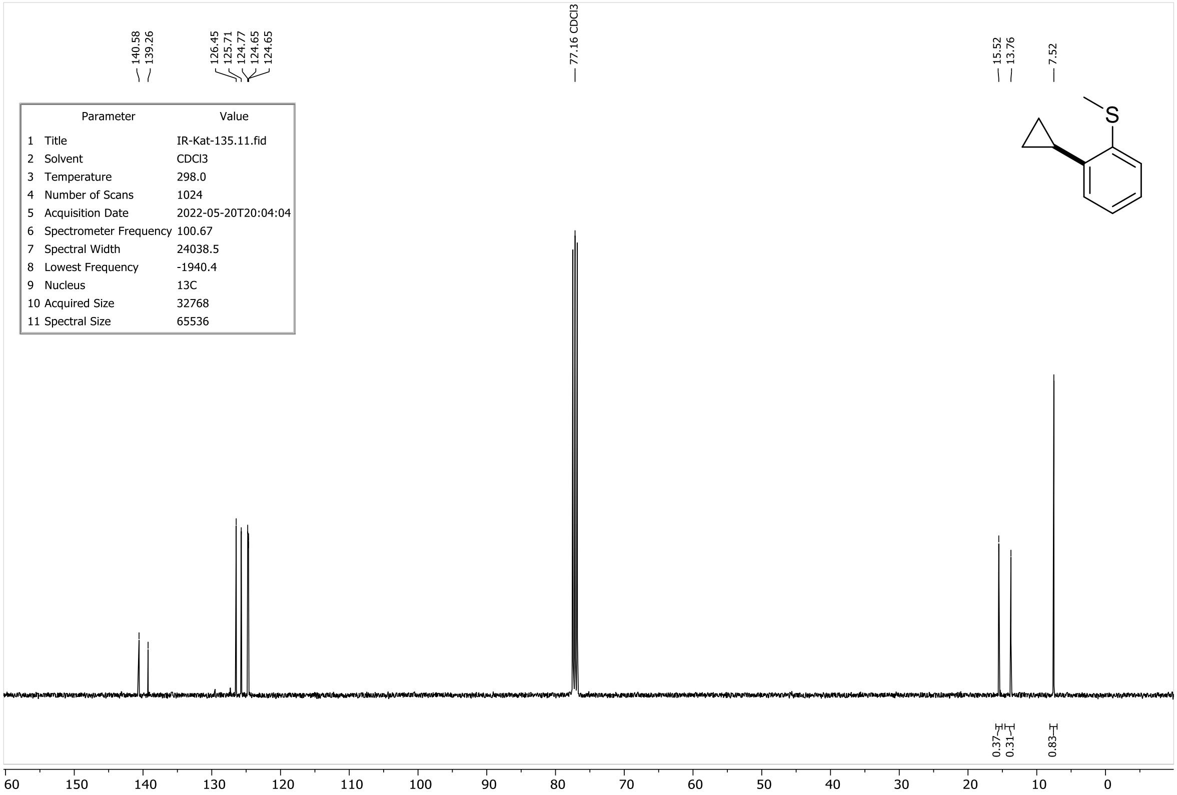

Figure S37b. $^{13}C\{^{1}H\}$ NMR spectrum of **Db**.

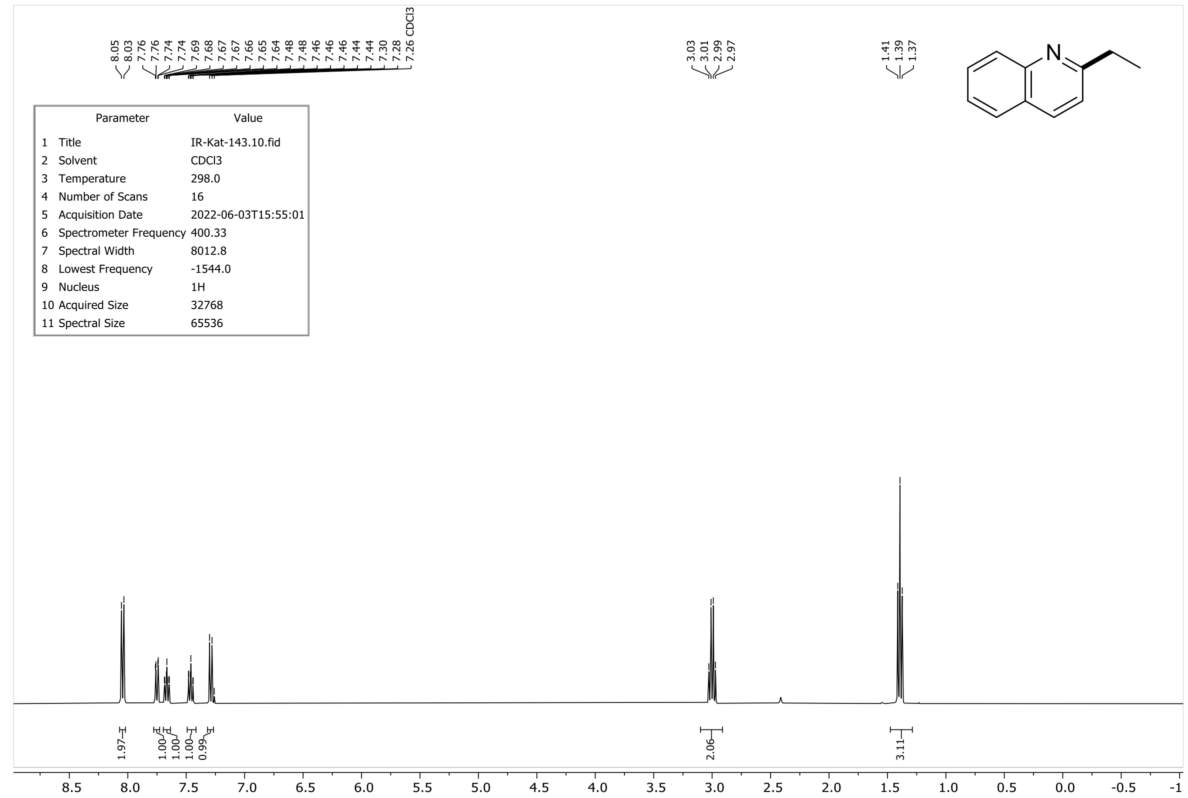

Figure S38. ^{1}H NMR spectrum of **Dc**.

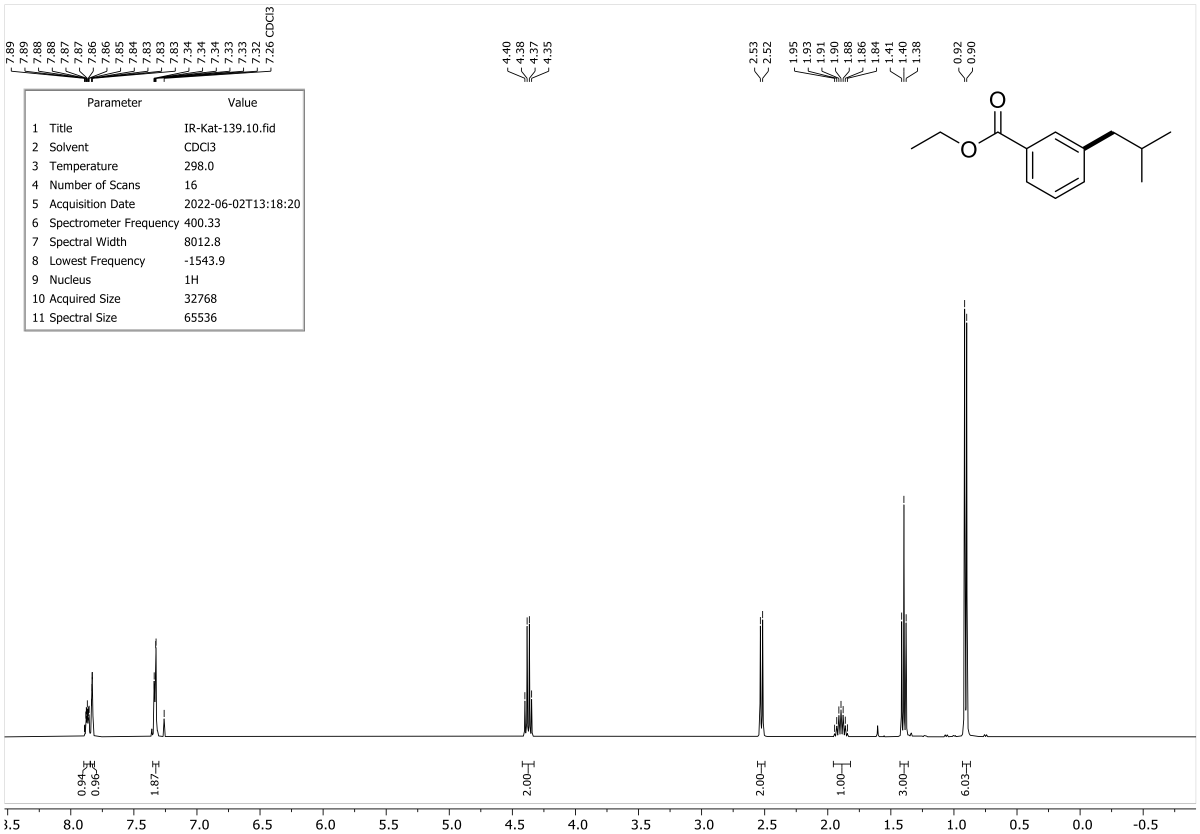

Figure S39. ^{1}H NMR spectrum of **Dd**.

4. Crystal Structure Determination

4.1 General Information

Data collection of all compounds was conducted on a Rigaku Synergy diffractometer. The structures were solved using direct methods, refined with the Shelx software package[21–23] and expanded using Fourier techniques. The crystals of all compounds were mounted in an inert oil (perfluoropolyalkylether). Crystal structure determinations were performed at 100 K. Crystallographic data (excluding structure factors) have been deposited with the Cambridge Crystallographic Data Centre as supplementary publication no. CCDC-2190326-2190335. Copies of the data can be obtained free of charge on application to Cambridge Crystallographic Data Centre, 12 Union Road, Cambridge CB2 1EZ, UK; [fax: (+44) 1223-336-033; email: deposit@ccdc.cam.ac.uk].

Details on the structure solution:

2 contained a highly disordered THF molecule, which was treated by using the PLATON/SQUEEZE routine.[24] The crystal contained a disordered THF molecule on a symmetrical center, which was modelled using a RIGU, SIMU and SAME restrain.

L1·$PdCl_2$ was refined as a two-component twin. In addition, the crystal contained two cyclohexyl-groups disordered over two positions with an occupancy of 0.74 and 0.26. This was modelled by using the EADP restrain.

L1H·$PdCl_3$ contained a highly disordered CH_2Cl_2 molecule, which was treated by using the PLATON/SQUEEZE routine. Two disagreeable reflections were flagged as unobserved by the OMIT restrain.

L2H·$PdCl_3$ was refined as a multi-component twin. It contained highly disordered CH_2Cl_2 molecules, which were treated by using the PLATON/SQUEEZE routine.

L4H·$PdCl_3$ contained a highly disordered THF molecule, which was treated by using the PLATON/SQUEEZE routine. The remaining THF molecule was disordered over two positions with an occupancy of 0.54:0.46. This was modelled by using the RIGU and SIMU restrain.

Table S1. Data collection and structure refinement details for the compounds **2**, **L1·PdBr₂** and **L1·PdI₂**.

Compound	**2**	**L1·PdBr$_2$**	**L1·PdI$_2$**
CCDC No.	2190326	2190328	2190330
Formula	$C_{45}H_{77}BrO_{1.5}P_2Pd$	$C_{32}H_{58}Br_2P_2Pd$	$C_{32}H_{58}I_2P_2Pd$
Formula weight [g·mol^{-1}]	890.31	770.94	864.92
Temperature [K]	100(2)	100(2)	101(2)
Wavelength [Å]	1.54184	1.54184	1.54184
Crystal system	Triclinic	Monoclinic	Monoclinic
Space group	$P\bar{1}$	$P2_1/c$	$P2_1/c$
a [Å]	11.8295(3)	10.53750(10)	10.58610(10)
b [Å]	15.3397(6)	17.5174(2)	17.7689(2)
c [Å]	15.9371(6)	18.2109(2)	18.6428(2)
α [°]	115.760(5)	90	90
β [°]	106.946(4)	92.9380(10)	92.1640(10)
γ [°]	97.999(3)	90	90
Volume [Å^3]	2369.90(18)	3357.12(6)	3504.27(6)
Z	2	4	4
Calc. density [Mg·m^{-3}]	1.248	1.525	1.639
μ [mm^{-1}]	5.005	8.278	19.094
F(000)	940	1584	1728
Crystal dimensions [mm]	0.105 x 0.066 x 0.047	0.189 x 0.095 x 0.071	0.150 x 0.070 x 0.030
Theta range [°]	3.352 to 78.682	3.503 to 77.093	3.473 to 76.521
Index ranges	$-14 \le h \le 14$	$-10 \le h \le 13$	$-12 \le h \le 12$
	$-19 \le k \le 19$	$-22 \le k \le 22$	$-21 \le k \le 8$
	$-20 \le l \le 19$	$-22 \le l \le 22$	$-23 \le l \le 22$
Reflections collected	30834	43386	25503
Independent reflections	9641 [R_{int} = 0.0396]	7005 [R_{int} = 0.0591]	6841 [R_{int} = 0.0440]
Data/Restraints/Parameter	9641 / 86 / 480	7005 / 0 / 335	6841 / 0 / 335
Goodness-of-fit on F^2	1.047	1.104	1.051
Final R indices [I>2sigma(I)]	*R*1 = 0.0420, w*R*2 = 0.1175	*R*1 = 0.0363, w*R*2 = 0.0925	*R*1 = 0.0388, w*R*2 = 0.0968
R indices (all data)	*R*1 = 0.0437, w*R*2 = 0.1191	*R*1 = 0.0410, w*R*2 = 0.0946	*R*1 = 0.0434, w*R*2 = 0.0996
Largest diff. peak and hole [e·Å^{-3}]	1.270 and -1.139	0.885 and -1.229	2.139 and -0.901

Table S2. Data collection and structure refinement details for the compounds **L1·PdCl2**, **L3·PdCl2** and **L4·PdCl2**.

Compound	**L1·PdCl2**	**L3·PdCl2**	**L4·PdCl2**
CCDC No.	2190329	2190333	2190335
Formula	$C_{32}H_{58}Cl_2P_2Pd$	$C_{37}H_{60}Cl_2P_2Pd$	$C_{38}H_{62}Cl_2P_2Pd$
Formula weight [g·mol^{-1}]	682.02	744.09	758.11
Temperature [K]	100(2)	100.00(10)	100(2)
Wavelength [Å]	1.54184	1.54184	1.54184
Crystal system	Monoclinic	Monoclinic	Monoclinic
Space group	$P2_1/c$	Cc	$P2_1/c$
a [Å]	9.84400(10)	19.1244(2)	12.8401(2)
b [Å]	16.99413(17)	10.50540(10)	15.1413(3)
c [Å]	19.4275(2)	17.9999(2)	20.3980(4)
α [°]	90	90	90
β [°]	98.2226(10)	94.9260(10)	106.091(2)
γ [°]	90	90	90
Volume [Å^3]	3216.61	3602.99(7)	3810.34(13)
Z	4	4	4
Calc. density [Mg·m^{-3}]	1.408	1.372	1.322
μ [mm^{-1}]	7.257	6.529	6.183
F(000)	1440	1568	1600
Crystal dimensions [mm]	0.150 x 0.085 x 0.073	0.211 x 0.085 x 0.065	0.176 x 0.098 x 0.050
Theta range [°]	3.471 to 77.009	4.641 to 77.524	3.583 to 76.993
Index ranges	–11 ≤ h ≤ 11	–22 ≤ h ≤ 24	–15 ≤ h ≤ 16
	–20 ≤ k ≤ 20	–13 ≤ k ≤ 13	–17 ≤ k ≤ 18
	–23 ≤ l ≤ 24	–22 ≤ l ≤ 20	–25 ≤ l ≤ 25
Reflections collected	13343	28402	28012
Independent reflections	13333 [R_{int} = n.d.]	5835 [R_{int} = 0.0242]	7756 [R_{int} = 0.0410]
Data/Restraints/Parameter	13333 / 0 / 374	5835 / 0 / 379	7756 / 0 / 389
Goodness-of-fit on F^2	1.091	1.079	1.126
Final R indices [I>2sigma(I)]	*R*1 = 0.0661, w*R*2 = 0.1848	*R*1 = 0.0244, w*R*2 = 0.0652	*R*1 = 0.0444, w*R*2 = 0.1230
R indices (all data)	*R*1 = 0.0760, w*R*2 = 0.1964	*R*1 = 0.0244, w*R*2 = 0.0653	*R*1 = 0.0489, w*R*2 = 0.1264
Largest diff. peak and hole [e·Å^{-3}]	2.818 and -1.779	0.436 and -0.575	1.537 and -1.649

Table S3. Data collection and structure refinement details for the compounds **L1H·PdCl₃** and **L2H·PdCl₃**.

Compound	**L1H·PdCl₃**	**L2H·PdCl₂**
CCDC No.	2190327	2190331
Formula	$C_{33}H_{61}Cl_5P_2Pd$	$C_{28}H_{55}Cl_3P_2Pd$
Formula weight [g·mol^{-1}]	803.40	666.41
Temperature [K]	100(2)	100(2)
Wavelength [Å]	1.54184	1.54184
Crystal system	Monoclinic	Monoclinic
Space group	$P2_1/c$	$P2_1/c$
a [Å]	9.83040(10)	17.0122(6)
b [Å]	16.81080(10)	14.9105(3)
c [Å]	24.1396(2)	18.1097(12))
α [°]	90	90
β [°]	93.6640(10)	109.673(5)
γ [°]	90	90
Volume [Å^3]	3981.08(6)	4325.6(4)
Z	4	4
Calc. density [Mg·m^{-3}]	1.340	1.023
μ [mm^{-1}]	7.753	5.940
F(000)	1680	1400
Crystal dimensions [mm]	0.200 x 0.100 x 0.060	0.298 x 0.197 x 0.088
Theta range [°]	3.670 to 76.483	2.758 to 77.634
Index ranges	–12 ≤ h ≤ 6	–21 ≤ h ≤ 20
	–21 ≤ k ≤ 20	–18 ≤ k ≤ 18
	–29 ≤ l ≤ 30	–22 ≤ l ≤ 22
Reflections collected	29158	8818
Independent reflections	9052 [R_{int} = 0.0175]	9052 [R_{int} = ?]
Data/Restraints/Parameter	7898 / 0 / 371	8818 / 0 / 316
Goodness-of-fit on F^2	1.052	1.065
Final R indices [I>2sigma(I)]	$R1$ = 0.0257, w$R2$ = 0.0621	$R1$ = 0.0588, w$R2$ = 0.1524
R indices (all data)	$R1$ = 0.0274, w$R2$ = 0.0631	$R1$ = 0.0642, w$R2$ = 0.1552
Largest diff. peak and hole [e·Å^{-3}]	0.637 and -0.602	1.121 and -1.057

Table S4. Data collection and structure refinement details for the compounds **L3H·PdCl3** and **L4H·PdCl3**.

Compound	**L3H·PdCl3**	**L4H·PdCl2**
CCDC No.	2190332	2190334
Formula	$C_{38}H_{63}Cl_5P_2Pd$	$C_{42}H_{71}Cl_3OP_2Pd$
Formula weight [g·mol^{-1}]	865.47	866.67
Temperature [K]	100(2)	100(2)
Wavelength [Å]	1.54184	1.54184
Crystal system	Orthorhombic	Monoclinic
Space group	$P2_12_12_1$	$P2_1/c$
a [Å]	12.62782(10)	22.1819(9)
b [Å]	17.78901(12)	9.9664(4)
c [Å]	18.10483(13)	20.8757(7)
α [°]	90	90
β [°]	90	94.071(4)
γ [°]	90	90
Volume [Å^3]	4067.00(5)	4603.4(3)
Z	4	4
Calc. density [Mg·m^{-3}]	1.413	1.251
μ [mm^{-1}]	7.634	5.718
F(000)	1808	1832
Crystal dimensions [mm]	0.144 x 0.076 x 0.056	0.140 x 0.060 x 0.020
Theta range [°]	3.483 to 77.064	3.996 to 77.803
Index ranges	–15 ≤ h ≤ 15	–27 ≤ h ≤ 26
	–14 ≤ k ≤ 22	–12 ≤ k ≤ 7
	–22 ≤ l ≤ 22	–23 ≤ l ≤ 26
Reflections collected	27029	30019
Independent reflections	8254 [R_{int} = 0.0253]	9113 [R_{int} = 0.0673]
Data/Restraints/Parameter	8254 / 0 / 415	9113 / 120 / 489
Goodness-of-fit on F^2	1.073	1.080
Final R indices [I>2sigma(I)]	*R*1 = 0.0197, w*R*2 = 0.0520	*R*1 = 0.0581, w*R*2 = 0.1497
R indices (all data)	*R*1 = 0.0203, w*R*2 = 0.0522	*R*1 = 0.0786, w*R*2 = 0.1586
Largest diff. peak and hole [e·Å^{-3}]	0.572 and -0.642	1.629 and -1.927

4.2 Details on the crystal structure analyses

4.2.1 Crystal Structure Determination of **2**

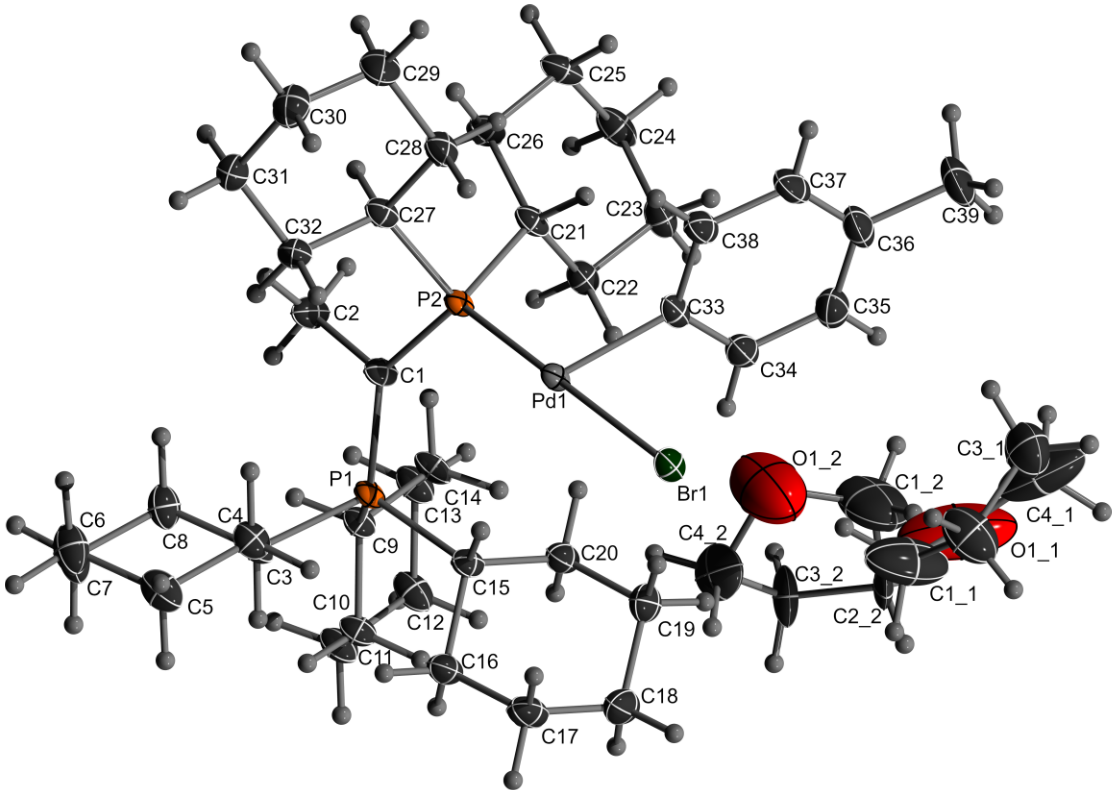

Figure S40. ORTEP Plot of **2**. Ellipsoids are drawn at the 50% probability level.

Table S5. Atomic coordinates (x 10^4) and equivalent isotropic displacement parameters (Å^2 x 10^3) for **2**. U(eq) is defined as one third of the trace of the orthogonalized Uij tensor.

	x	y	z	U(eq)
Pd(1)	5810(1)	5521(1)	1410(1)	16(1)
Br(1)	5720(1)	6135(1)	165(1)	21(1)
P(1)	3050(1)	4893(1)	2349(1)	17(1)
P(2)	5862(1)	5096(1)	2638(1)	15(1)
C(1)	4498(3)	4794(2)	2827(2)	19(1)
C(2)	4624(3)	4375(3)	3559(3)	26(1)
C(3)	1781(3)	3661(2)	1443(2)	20(1)
C(4)	1986(3)	3068(3)	469(2)	24(1)
C(5)	900(3)	2090(3)	-302(3)	31(1)
C(6)	722(4)	1407(3)	135(3)	35(1)
C(7)	521(4)	1981(3)	1118(3)	36(1)
C(8)	1594(3)	2973(3)	1896(3)	28(1)
C(9)	2504(3)	5466(2)	3391(2)	19(1)
C(10)	1263(3)	5690(2)	3087(2)	22(1)
C(11)	836(3)	6055(3)	3971(2)	25(1)
C(12)	1835(3)	6994(3)	4926(3)	28(1)

C(13)	3063(3)	6773(3)	5225(2)	27(1)
C(14)	3505(3)	6401(3)	4356(2)	23(1)
C(15)	2995(3)	5603(2)	1665(2)	19(1)
C(16)	1701(3)	5384(3)	883(2)	25(1)
C(17)	1836(3)	5906(3)	269(3)	31(1)
C(18)	2431(3)	7048(3)	970(3)	34(1)
C(19)	3701(3)	7295(3)	1770(3)	30(1)
C(20)	3590(3)	6758(2)	2375(2)	23(1)
C(21)	7143(3)	6082(2)	3897(2)	20(1)
C(22)	6743(3)	6998(2)	4429(2)	21(1)
C(23)	7850(3)	7903(3)	5321(3)	28(1)
C(24)	8501(3)	7611(3)	6083(2)	30(1)
C(25)	8890(3)	6670(3)	5574(2)	26(1)
C(26)	7789(3)	5773(3)	4667(2)	24(1)
C(27)	6446(3)	3981(2)	2378(2)	19(1)
C(28)	7627(3)	4134(3)	2167(3)	24(1)
C(29)	8190(3)	3280(3)	2086(3)	36(1)
C(30)	7253(4)	2243(3)	1270(3)	34(1)
C(31)	6056(3)	2092(3)	1456(3)	27(1)
C(32)	5502(3)	2954(2)	1539(2)	23(1)
C(33)	7258(3)	6785(2)	2352(2)	22(1)
C(34)	7169(3)	7755(3)	2846(2)	26(1)
C(35)	8211(3)	8612(3)	3323(3)	32(1)
C(36)	9345(3)	8528(3)	3313(3)	36(1)
C(37)	9448(3)	7556(3)	2817(3)	33(1)
C(38)	8418(3)	6691(3)	2343(3)	26(1)
C(39)	10472(4)	9460(4)	3804(4)	50(1)
O11	6517(6)	10102(6)	2607(4)	130(2)
C11	5972(6)	9224(7)	1561(5)	91(2)
C21	6774(5)	9407(4)	1086(4)	59(1)
C31	8042(5)	10021(4)	1924(4)	58(1)
C41	7744(8)	10676(5)	2754(5)	98(2)
O13	5464(12)	9452(10)	4671(13)	115(5)
C13	5931(14)	10593(12)	5061(18)	95(7)
C23	5003(8)	11089(5)	5256(7)	40(2)
C33	4210(11)	10361(7)	5359(9)	40(2)
C43	4151(12)	9319(7)	4616(10)	55(3)

Table S6. Anisotropic displacement parameters (Å^2 x 10^3) for **2**. The anisotropic displacement factor exponent takes the form: $-2\pi^2$ [$h^2a^{*2}U^{11}$ + ... + $2\ h\ k\ a^*b^*U^{12}$].

	U^{11}	U^{22}	U^{33}	U^{23}	U^{13}	U^{12}
Pd(1)	12(1)	23(1)	14(1)	11(1)	5(1)	3(1)
Br(1)	21(1)	28(1)	17(1)	14(1)	7(1)	2(1)
P(1)	12(1)	22(1)	17(1)	10(1)	7(1)	4(1)
P(2)	12(1)	21(1)	14(1)	10(1)	5(1)	4(1)

C(1)	14(1)	28(2)	19(1)	15(1)	9(1)	7(1)
C(2)	20(2)	42(2)	31(2)	27(2)	14(1)	12(1)
C(3)	13(1)	24(1)	19(1)	9(1)	7(1)	4(1)
C(4)	23(2)	28(2)	22(2)	11(1)	13(1)	7(1)
C(5)	27(2)	31(2)	24(2)	5(1)	10(1)	6(1)
C(6)	40(2)	22(2)	36(2)	7(1)	20(2)	4(1)
C(7)	40(2)	25(2)	37(2)	9(2)	23(2)	-1(2)
C(8)	31(2)	25(2)	28(2)	12(1)	16(1)	4(1)
C(9)	16(1)	24(1)	19(1)	10(1)	10(1)	5(1)
C(10)	16(1)	28(2)	23(2)	11(1)	10(1)	7(1)
C(11)	18(2)	29(2)	25(2)	9(1)	11(1)	6(1)
C(12)	22(2)	28(2)	28(2)	5(1)	14(1)	5(1)
C(13)	21(2)	30(2)	21(2)	7(1)	8(1)	3(1)
C(14)	14(1)	29(2)	21(1)	9(1)	8(1)	3(1)
C(15)	15(1)	27(2)	20(1)	14(1)	8(1)	6(1)
C(16)	17(2)	33(2)	24(2)	16(1)	5(1)	6(1)
C(17)	21(2)	45(2)	30(2)	26(2)	5(1)	7(1)
C(18)	28(2)	43(2)	43(2)	33(2)	10(2)	9(2)
C(19)	28(2)	33(2)	36(2)	25(2)	13(2)	6(1)
C(20)	18(1)	28(2)	24(2)	15(1)	7(1)	6(1)
C(21)	14(1)	27(2)	18(1)	11(1)	7(1)	3(1)
C(22)	19(1)	22(1)	19(1)	9(1)	7(1)	3(1)
C(23)	24(2)	25(2)	24(2)	9(1)	6(1)	-2(1)
C(24)	22(2)	36(2)	18(1)	7(1)	5(1)	-1(1)
C(25)	15(1)	42(2)	20(1)	16(1)	5(1)	6(1)
C(26)	20(2)	31(2)	17(1)	13(1)	4(1)	6(1)
C(27)	15(1)	25(2)	17(1)	11(1)	7(1)	4(1)
C(28)	19(2)	26(2)	32(2)	15(1)	15(1)	7(1)
C(29)	22(2)	37(2)	56(2)	25(2)	21(2)	14(2)
C(30)	37(2)	29(2)	47(2)	21(2)	26(2)	16(2)
C(31)	26(2)	27(2)	32(2)	18(1)	11(1)	8(1)
C(32)	19(1)	24(2)	22(1)	12(1)	5(1)	6(1)
C(33)	18(1)	29(2)	18(1)	16(1)	4(1)	-1(1)
C(34)	22(2)	30(2)	25(2)	17(1)	6(1)	4(1)
C(35)	32(2)	30(2)	32(2)	19(2)	7(2)	3(1)
C(36)	26(2)	37(2)	38(2)	24(2)	2(2)	-7(2)
C(37)	17(2)	45(2)	42(2)	29(2)	9(1)	2(1)
C(38)	20(2)	34(2)	31(2)	21(1)	10(1)	6(1)
C(39)	31(2)	45(2)	62(3)	29(2)	8(2)	-11(2)
O11	137(4)	238(7)	83(3)	87(3)	81(3)	146(4)
C11	52(3)	174(6)	92(3)	90(3)	42(3)	52(4)
C21	47(2)	63(3)	47(2)	15(2)	20(2)	6(2)
C31	57(3)	40(2)	56(2)	19(2)	10(2)	2(2)
C41	142(4)	88(4)	51(3)	26(3)	13(3)	83(4)
O13	82(7)	87(7)	139(12)	23(8)	46(7)	33(6)
C13	49(6)	88(7)	127(18)	33(8)	35(8)	35(5)
C23	33(4)	15(3)	69(5)	22(3)	18(4)	0(3)

C33	55(5)	16(3)	41(4)	8(3)	24(4)	-4(3)
C43	64(6)	23(3)	40(5)	-2(3)	6(4)	6(4)
Pd(1)	12(1)	23(1)	14(1)	11(1)	5(1)	3(1)

4.2.2 Crystal Structure Determination of **L1·PdBr$_2$**

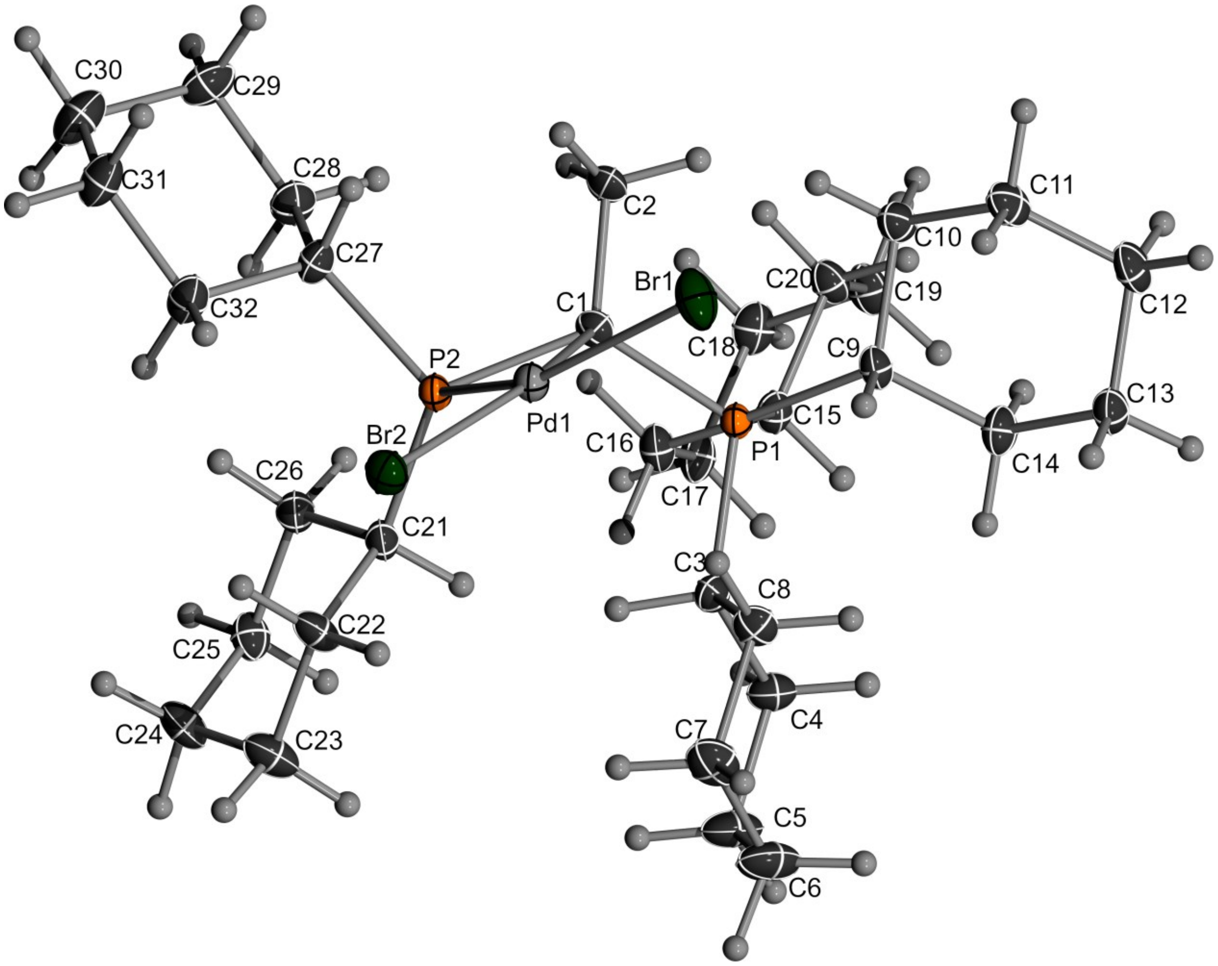

Figure S41. ORTEP Plot of **L1·PdBr$_2$**. Ellipsoids are drawn at the 50% probability level.

Table S7. Atomic coordinates (x 10^4) and equivalent isotropic displacement parameters (Å^2 x 10^3) for **L1·PdBr$_2$**. U(eq) is defined as one third of the trace of the orthogonalized Uij tensor.

	x	y	z	U(eq)
Pd(1)	3874(1)	7590(1)	6582(1)	15(1)
Br(1)	6174(1)	7317(1)	6327(1)	27(1)
Br(2)	3796(1)	8813(1)	5893(1)	21(1)
P(1)	3942(1)	6657(1)	8115(1)	14(1)
P(2)	1976(1)	7302(1)	6889(1)	14(1)
C(1)	3165(3)	6640(2)	7213(2)	16(1)
C(2)	3142(3)	5850(2)	6844(2)	22(1)
C(3)	3947(3)	7642(2)	8498(2)	17(1)

C(4)	3754(3)	7698(2)	9330(2)	22(1)
C(5)	3619(4)	8536(2)	9554(2)	29(1)
C(6)	4758(4)	9017(2)	9349(2)	33(1)
C(7)	4975(4)	8941(2)	8532(2)	28(1)
C(8)	5139(3)	8108(2)	8309(2)	19(1)
C(9)	5619(3)	6381(2)	8088(2)	16(1)
C(10)	5987(3)	5720(2)	7583(2)	20(1)
C(11)	7436(3)	5674(2)	7587(2)	22(1)
C(12)	8042(3)	5566(2)	8357(2)	23(1)
C(13)	7631(3)	6203(2)	8872(2)	20(1)
C(14)	6184(3)	6257(2)	8878(2)	20(1)
C(15)	3147(3)	6029(2)	8751(2)	17(1)
C(16)	1716(3)	6202(2)	8759(2)	19(1)
C(17)	1121(3)	5744(2)	9367(2)	21(1)
C(18)	1319(3)	4886(2)	9251(2)	22(1)
C(19)	2731(3)	4702(2)	9225(2)	22(1)
C(20)	3348(3)	5167(2)	8623(2)	19(1)
C(21)	950(3)	7851(2)	7503(2)	16(1)
C(22)	1013(3)	8702(2)	7300(2)	21(1)
C(23)	185(3)	9181(2)	7795(2)	27(1)
C(24)	-1183(3)	8892(2)	7769(2)	25(1)
C(25)	-1236(3)	8055(2)	7979(2)	20(1)
C(26)	-426(3)	7564(2)	7493(2)	19(1)
C(27)	1025(3)	6888(2)	6109(2)	19(1)
C(28)	28(3)	6269(2)	6255(2)	22(1)
C(29)	-471(4)	5929(2)	5523(2)	27(1)
C(30)	-1055(4)	6552(2)	5023(2)	31(1)
C(31)	-92(4)	7182(2)	4891(2)	28(1)
C(32)	446(3)	7528(2)	5616(2)	22(1)

Table S8. Anisotropic displacement parameters ($Å^2$ x 10^3) for **L1·PdBr₂**. The anisotropic displacement factor exponent takes the form: $-2\pi^2$ [$h^2a^{*2}U^{11}$ + ... + 2 h k $a^*b^*U^{12}$].

	U^{11}	U^{22}	U^{33}	U^{23}	U^{13}	U^{12}
Pd(1)	14(1)	15(1)	16(1)	1(1)	-1(1)	0(1)
Br(1)	19(1)	33(1)	31(1)	10(1)	5(1)	5(1)
Br(2)	24(1)	17(1)	23(1)	4(1)	-1(1)	-2(1)
P(1)	12(1)	13(1)	16(1)	1(1)	-2(1)	1(1)
P(2)	12(1)	13(1)	16(1)	1(1)	-2(1)	0(1)
C(1)	18(1)	12(1)	16(1)	2(1)	1(1)	-1(1)
C(2)	28(2)	11(1)	26(2)	-1(1)	-6(1)	3(1)
C(3)	14(1)	15(1)	21(2)	-2(1)	-3(1)	0(1)
C(4)	24(2)	21(2)	21(2)	-5(1)	4(1)	-1(1)
C(5)	32(2)	26(2)	30(2)	-13(2)	8(2)	-4(2)
C(6)	33(2)	26(2)	41(2)	-16(2)	4(2)	-7(2)

C(7)	27(2)	18(2)	39(2)	0(1)	2(2)	-4(1)
C(8)	21(2)	14(1)	23(2)	1(1)	2(1)	-3(1)
C(9)	12(1)	16(1)	19(1)	3(1)	-1(1)	1(1)
C(10)	16(1)	18(2)	25(2)	-2(1)	-1(1)	1(1)
C(11)	15(2)	22(2)	30(2)	-2(1)	3(1)	2(1)
C(12)	13(1)	22(2)	35(2)	3(1)	-1(1)	3(1)
C(13)	16(2)	20(2)	24(2)	3(1)	-4(1)	0(1)
C(14)	14(2)	25(2)	22(2)	3(1)	-2(1)	0(1)
C(15)	15(1)	16(1)	19(1)	3(1)	-2(1)	-2(1)
C(16)	15(2)	20(2)	22(2)	4(1)	-2(1)	1(1)
C(17)	14(1)	27(2)	21(2)	4(1)	-1(1)	-1(1)
C(18)	18(2)	26(2)	24(2)	5(1)	1(1)	-4(1)
C(19)	22(2)	18(2)	27(2)	7(1)	1(1)	1(1)
C(20)	17(2)	15(1)	24(2)	3(1)	1(1)	1(1)
C(21)	14(1)	16(1)	19(1)	2(1)	-2(1)	2(1)
C(22)	20(2)	13(1)	31(2)	-2(1)	5(1)	2(1)
C(23)	26(2)	17(2)	40(2)	-3(1)	9(2)	2(1)
C(24)	22(2)	20(2)	33(2)	1(1)	6(1)	7(1)
C(25)	16(2)	24(2)	21(2)	3(1)	2(1)	2(1)
C(26)	18(2)	18(2)	21(2)	1(1)	-2(1)	0(1)
C(27)	18(2)	21(2)	17(1)	0(1)	-3(1)	-3(1)
C(28)	21(2)	18(2)	26(2)	0(1)	-2(1)	-5(1)
C(29)	30(2)	25(2)	27(2)	-4(1)	-2(1)	-10(1)
C(30)	26(2)	43(2)	22(2)	-3(2)	-4(1)	-9(2)
C(31)	30(2)	34(2)	20(2)	3(1)	-6(1)	-2(2)
C(32)	23(2)	22(2)	22(2)	4(1)	-4(1)	-3(1)

4.2.3 Crystal Structure Determination of **L1·PdI₂**

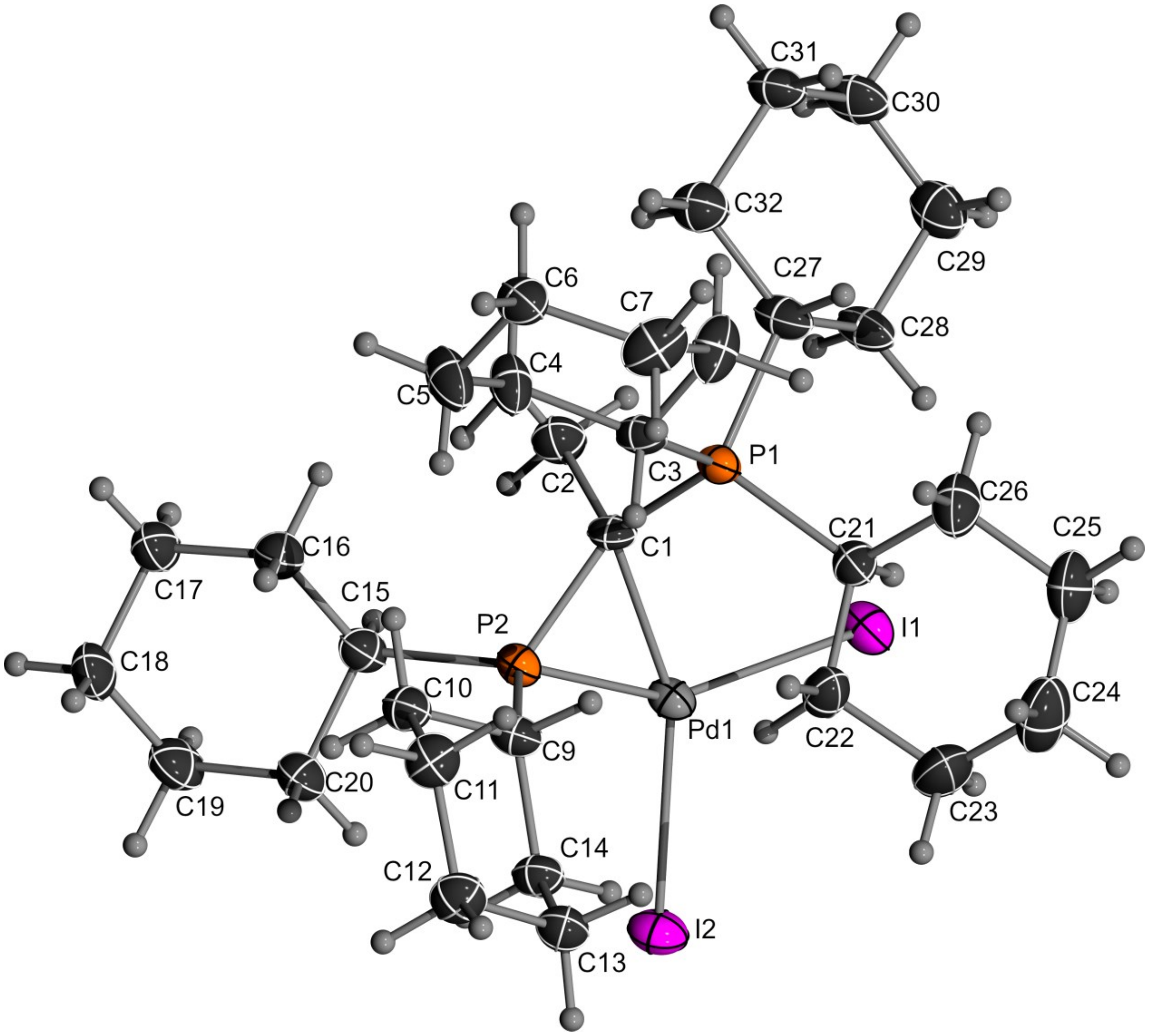

Figure S42. ORTEP Plot of **L1·PdI$_2$**. Ellipsoids are drawn at the 50% probability level.

Table S9. Atomic coordinates (x 10^4) and equivalent isotropic displacement parameters (Å^2 x 10^3) for **L1·PdI$_2$**. U(eq) is defined as one third of the trace of the orthogonalized Uij tensor.

	x	y	z	U(eq)
I(1)	3940(1)	8666(1)	7531(1)	32(1)
I(2)	7068(1)	9490(1)	8602(1)	34(1)
Pd(1)	6473(1)	8563(1)	7525(1)	24(1)
P(1)	6270(1)	6857(1)	6678(1)	23(1)
P(2)	8229(1)	8179(1)	7089(1)	23(1)
C(1)	6846(4)	7816(2)	6626(2)	23(1)
C(2)	6485(5)	8210(3)	5913(3)	34(1)
C(3)	7542(4)	6188(3)	6487(2)	27(1)

C(4)	8375(5)	6424(3)	5873(3)	35(1)
C(5)	9498(5)	5886(3)	5823(4)	43(1)
C(6)	9062(5)	5081(3)	5715(3)	33(1)
C(7)	8185(6)	4840(3)	6306(3)	41(1)
C(8)	7068(5)	5369(3)	6342(3)	37(1)
C(9)	9280(4)	7533(3)	7614(2)	25(1)
C(10)	10508(5)	7273(3)	7281(3)	28(1)
C(11)	11119(5)	6664(3)	7755(3)	33(1)
C(12)	11382(5)	6944(3)	8519(3)	34(1)
C(13)	10186(5)	7249(3)	8849(3)	32(1)
C(14)	9556(5)	7852(3)	8377(2)	28(1)
C(15)	9127(5)	8836(3)	6531(3)	29(1)
C(16)	9790(5)	8474(3)	5898(3)	31(1)
C(17)	10440(5)	9079(3)	5456(3)	35(1)
C(18)	11359(5)	9539(3)	5922(3)	38(1)
C(19)	10699(6)	9891(3)	6543(3)	38(1)
C(20)	10044(5)	9302(3)	7007(3)	32(1)
C(21)	5629(4)	6654(3)	7565(3)	26(1)
C(22)	6560(5)	6767(3)	8199(3)	36(1)
C(23)	5916(7)	6654(5)	8913(3)	56(2)
C(24)	5271(6)	5903(4)	8971(4)	56(2)
C(25)	4361(6)	5788(4)	8342(3)	46(1)
C(26)	4986(6)	5878(3)	7624(3)	42(1)
C(27)	4944(5)	6652(3)	6040(3)	33(1)
C(28)	3735(4)	7095(3)	6189(3)	32(1)
C(29)	2608(5)	6770(3)	5748(3)	40(1)
C(30)	2860(5)	6727(3)	4966(3)	40(1)
C(31)	4053(5)	6277(3)	4827(3)	34(1)
C(32)	5197(5)	6629(3)	5249(3)	38(1)

Table S10. Anisotropic displacement parameters ($Å^2$ x 10^3) for **L1·PdI$_2$**. The anisotropic displacement factor exponent takes the form: $-2\pi^2$ [$h^2a^{*2}U^{11}$ + ... + 2 h k a·b·U^{12}].

	U^{11}	U^{22}	U^{33}	U^{23}	U^{13}	U^{12}
I(1)	24(1)	26(1)	46(1)	-9(1)	0(1)	3(1)
I(2)	38(1)	28(1)	36(1)	-10(1)	-3(1)	2(1)
Pd(1)	22(1)	20(1)	28(1)	-3(1)	-1(1)	1(1)
P(1)	20(1)	24(1)	24(1)	-6(1)	-2(1)	0(1)
P(2)	22(1)	22(1)	26(1)	-3(1)	-2(1)	-1(1)
C(1)	26(2)	21(2)	20(2)	-7(2)	-1(2)	1(2)
C(2)	33(3)	39(3)	28(2)	5(2)	-6(2)	-2(2)
C(3)	20(2)	34(3)	25(2)	-8(2)	-4(2)	5(2)
C(4)	30(3)	26(2)	51(3)	-9(2)	17(2)	-5(2)
C(5)	28(3)	38(3)	64(4)	-18(3)	15(3)	-6(2)
C(6)	28(3)	34(3)	36(3)	-11(2)	4(2)	2(2)
C(7)	52(3)	36(3)	35(3)	5(2)	1(2)	14(3)

C(8)	43(3)	24(2)	45(3)	8(2)	17(2)	6(2)
C(9)	25(2)	25(2)	26(2)	-4(2)	-5(2)	1(2)
C(10)	26(2)	30(2)	28(2)	-4(2)	-1(2)	1(2)
C(11)	33(3)	31(3)	35(3)	2(2)	0(2)	7(2)
C(12)	32(3)	39(3)	32(3)	3(2)	-4(2)	5(2)
C(13)	29(3)	37(3)	29(2)	0(2)	-4(2)	-1(2)
C(14)	27(2)	33(3)	25(2)	-3(2)	-4(2)	2(2)
C(15)	29(3)	27(2)	29(2)	0(2)	-3(2)	-4(2)
C(16)	31(3)	31(3)	30(2)	-2(2)	1(2)	-2(2)
C(17)	36(3)	37(3)	32(3)	3(2)	-1(2)	-9(2)
C(18)	33(3)	39(3)	41(3)	6(2)	3(2)	-12(2)
C(19)	43(3)	30(3)	42(3)	0(2)	-4(2)	-11(2)
C(20)	32(3)	30(2)	33(3)	-3(2)	-1(2)	-5(2)
C(21)	23(2)	25(2)	30(2)	-7(2)	4(2)	1(2)
C(22)	27(3)	55(3)	26(2)	5(2)	1(2)	-1(2)
C(23)	54(4)	87(5)	27(3)	2(3)	1(3)	-14(4)
C(24)	46(4)	76(5)	48(4)	27(3)	8(3)	2(3)
C(25)	42(3)	45(3)	51(3)	12(3)	14(3)	-4(3)
C(26)	43(3)	34(3)	49(3)	-3(2)	17(3)	-6(2)
C(27)	25(2)	36(3)	38(3)	-16(2)	-6(2)	5(2)
C(28)	23(2)	34(3)	40(3)	-12(2)	-7(2)	4(2)
C(29)	28(3)	43(3)	49(3)	-5(2)	-1(2)	3(2)
C(30)	31(3)	48(3)	39(3)	-3(2)	-13(2)	4(2)
C(31)	31(3)	40(3)	30(2)	-9(2)	-8(2)	1(2)
C(32)	37(3)	41(3)	35(3)	-3(2)	-4(2)	-1(2)

4.2.4 Crystal Structure Determination of **L1·PdCl$_2$**

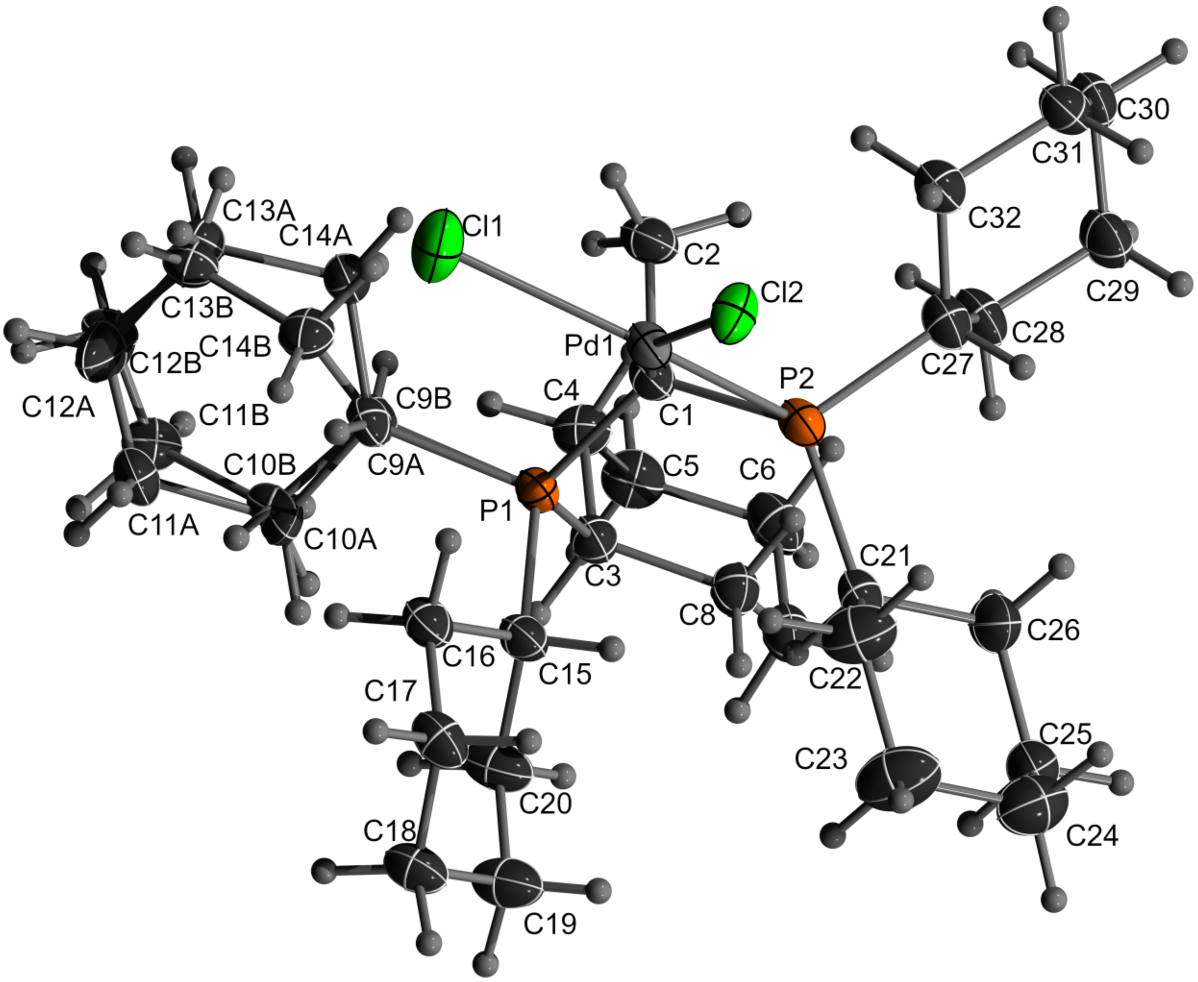

Figure S43. ORTEP Plot of **L1·PdCl$_2$**. Ellipsoids are drawn at the 50% probability level.

Table S11. Atomic coordinates (x 10^4) and equivalent isotropic displacement parameters ($Å^2$ x 10^3) for **L1·PdCl$_2$**. U(eq) is defined as one third of the trace of the orthogonalized Uij tensor.

	x	y	z	U(eq)
Pd(1)	4151(1)	5102(1)	6751(1)	25(1)
Cl(1)	3619(2)	6038(1)	7607(1)	43(1)
Cl(2)	5311(1)	5979(1)	6066(1)	29(1)
P(1)	3501(1)	3612(1)	7811(1)	24(1)
P(2)	3891(1)	3969(1)	6240(1)	26(1)
C(1)	3027(5)	4096(3)	6987(2)	25(1)
C(2)	1486(5)	4316(3)	6843(3)	34(1)
C(3)	2781(5)	2606(3)	7769(3)	26(1)
C(4)	1209(6)	2574(3)	7751(3)	33(1)
C(5)	703(6)	1725(3)	7705(3)	37(1)
C(6)	1090(6)	1314(4)	7063(3)	37(1)
C(7)	2645(6)	1328(3)	7084(3)	33(1)

C(8)	3199(6)	2165(3)	7142(3)	28(1)
C(9A)	3013(12)	4208(7)	8528(6)	23(2)
C(10A)	3290(13)	3714(8)	9219(8)	28(2)
C(11A)	3124(9)	4231(5)	9834(4)	35(2)
C(12A)	1726(11)	4610(7)	9769(5)	34(2)
C(13A)	1449(11)	5097(11)	9108(11)	29(3)
C(14A)	1590(7)	4609(4)	8446(3)	27(2)
C(9B)	2610(30)	3992(19)	8563(18)	29(6)
C(10B)	2960(40)	3560(30)	9200(30)	28(2)
C(11B)	2160(20)	3872(13)	9806(12)	35(5)
C(12B)	2190(30)	4760(20)	9876(17)	34(2)
C(13B)	1800(50)	5150(30)	9150(30)	29(3)
C(14B)	2670(30)	4890(11)	8650(12)	35(6)
C(15)	5381(5)	3544(3)	8018(3)	28(1)
C(16)	6064(6)	4301(3)	8354(3)	30(1)
C(17)	7623(6)	4244(3)	8394(3)	34(1)
C(18)	8177(6)	3508(3)	8795(3)	36(1)
C(19)	7491(6)	2768(4)	8477(4)	43(1)
C(20)	5931(6)	2811(3)	8424(4)	40(1)
C(21)	5357(5)	3271(3)	6215(3)	28(1)
C(22)	6633(6)	3759(4)	6145(4)	45(2)
C(23)	7903(7)	3226(4)	6166(5)	56(2)
C(24)	7683(7)	2598(4)	5603(4)	49(2)
C(25)	6416(6)	2115(4)	5680(3)	37(1)
C(26)	5134(6)	2635(4)	5652(3)	39(1)
C(27)	2768(6)	3866(3)	5396(3)	31(1)
C(28)	1737(6)	3176(3)	5364(3)	33(1)
C(29)	928(6)	3116(4)	4633(3)	37(1)
C(30)	196(6)	3888(4)	4422(3)	34(1)
C(31)	1250(5)	4551(3)	4427(3)	32(1)
C(32)	2105(6)	4646(3)	5146(3)	31(1)

Table S12. Anisotropic displacement parameters ($Å^2 \times 10^3$) for **L1·PdCl₂**. The anisotropic displacement factor exponent takes the form: $-2\pi^2 [h^2 a^{*2} U^{11} + ... + 2 h k a^* b^* U^{12}]$.

	U^{11}	U^{22}	U^{33}	U^{23}	U^{13}	U^{12}
Pd(1)	31(1)	22(1)	20(1)	1(1)	-2(1)	-2(1)
Cl(1)	46(1)	48(1)	38(1)	-16(1)	14(1)	-8(1)
Cl(2)	40(1)	21(1)	28(1)	0(1)	16(1)	-4(1)
P(1)	26(1)	25(1)	21(1)	-2(1)	-3(1)	2(1)
P(2)	30(1)	24(1)	21(1)	1(1)	-3(1)	-2(1)
C(1)	29(2)	22(2)	22(2)	1(2)	-1(2)	-1(2)
C(2)	30(3)	34(3)	34(3)	3(2)	-5(2)	4(2)
C(3)	30(3)	24(2)	23(2)	0(2)	0(2)	0(2)
C(4)	31(3)	31(3)	37(3)	1(2)	3(2)	1(2)
C(5)	34(3)	35(3)	41(3)	3(2)	5(2)	-3(2)

C(6)	43(3)	32(3)	31(3)	1(2)	-5(2)	-8(2)
C(7)	44(3)	28(3)	26(2)	-2(2)	2(2)	-1(2)
C(8)	34(3)	25(2)	26(2)	-1(2)	4(2)	-1(2)
C(9A)	28(6)	24(6)	17(3)	2(4)	0(4)	-1(4)
C(10A)	30(7)	24(6)	26(3)	-7(4)	-6(5)	-3(4)
C(11A)	44(5)	40(4)	19(3)	-2(3)	-1(3)	6(4)
C(12A)	40(7)	37(5)	25(4)	1(3)	6(4)	3(4)
C(13A)	28(8)	32(4)	28(4)	0(3)	5(7)	4(6)
C(14A)	30(4)	29(4)	22(3)	-1(3)	1(3)	2(3)
C(9B)	29(16)	25(16)	30(12)	-2(11)	-5(11)	-5(10)
C(10B)	30(7)	24(6)	26(3)	-7(4)	-6(5)	-3(4)
C(11B)	30(11)	36(11)	40(11)	9(8)	5(8)	-6(8)
C(12B)	40(7)	37(5)	25(4)	1(3)	6(4)	3(4)
C(13B)	28(8)	32(4)	28(4)	0(3)	5(7)	4(6)
C(14B)	52(14)	15(9)	38(11)	3(7)	4(10)	5(8)
C(15)	28(2)	28(3)	25(2)	0(2)	-4(2)	1(2)
C(16)	35(3)	28(3)	25(2)	3(2)	-2(2)	0(2)
C(17)	36(3)	33(3)	29(3)	2(2)	-4(2)	-6(2)
C(18)	30(3)	35(3)	38(3)	5(2)	-7(2)	-1(2)
C(19)	35(3)	33(3)	56(4)	3(3)	-10(3)	3(2)
C(20)	35(3)	27(3)	52(3)	6(2)	-11(3)	-1(2)
C(21)	34(3)	28(3)	19(2)	2(2)	-1(2)	-2(2)
C(22)	33(3)	34(3)	67(4)	-1(3)	8(3)	-3(2)
C(23)	35(3)	46(4)	85(6)	-6(4)	8(3)	-4(3)
C(24)	47(4)	45(4)	57(4)	7(3)	16(3)	8(3)
C(25)	42(3)	39(3)	30(3)	-3(2)	0(2)	8(3)
C(26)	41(3)	37(3)	35(3)	-10(2)	-3(2)	3(3)
C(27)	34(3)	33(3)	23(2)	-1(2)	-2(2)	-3(2)
C(28)	38(3)	33(3)	26(2)	2(2)	-6(2)	-6(2)
C(29)	39(3)	39(3)	30(3)	-4(2)	-6(2)	-2(2)
C(30)	33(3)	43(3)	25(2)	-5(2)	-2(2)	-1(2)
C(31)	31(3)	38(3)	25(2)	4(2)	-1(2)	1(2)
C(32)	33(3)	33(3)	27(3)	2(2)	0(2)	0(2)

4.2.5 Crystal Structure Determination of **L3·PdCl₂**

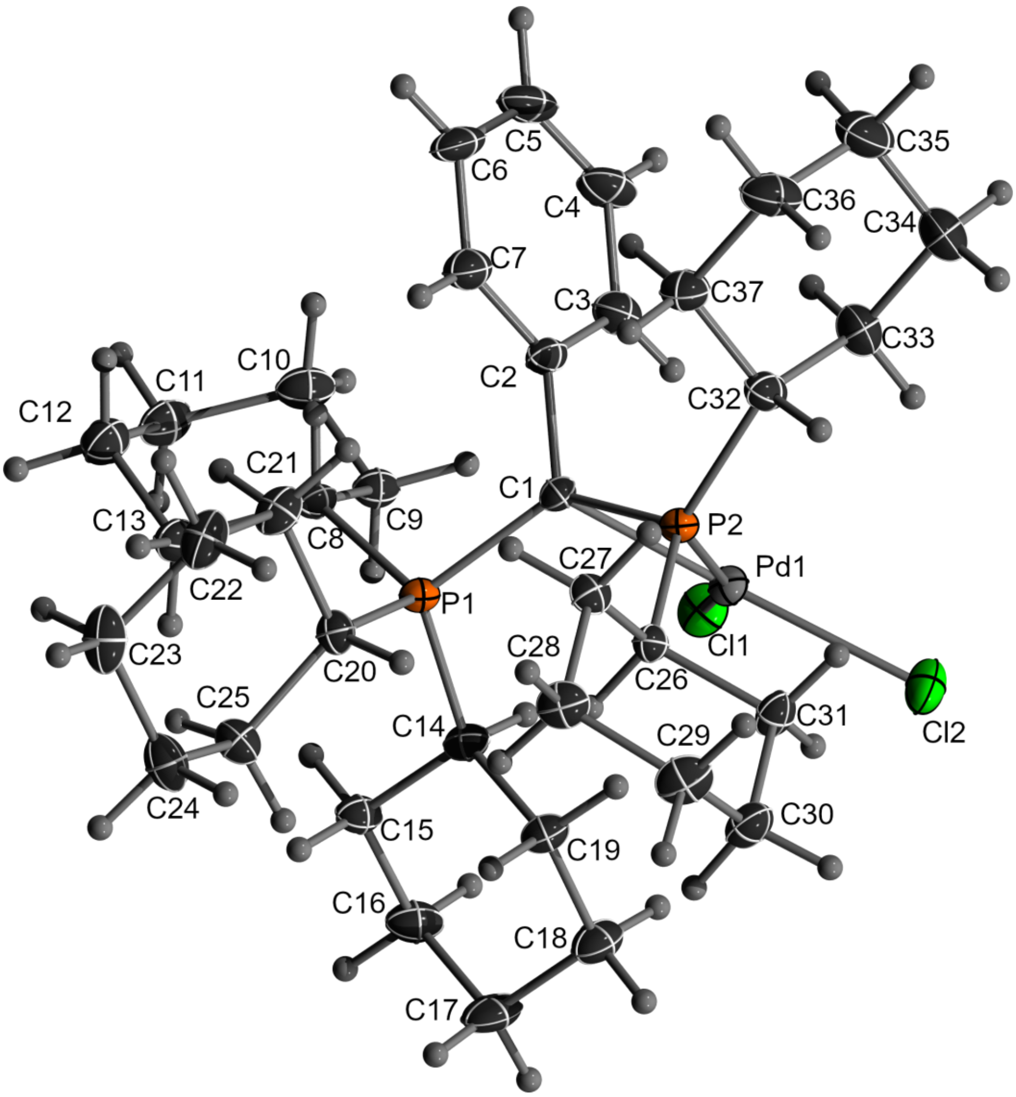

Figure S44. ORTEP Plot of **L3·$PdCl_2$**. Ellipsoids are drawn at the 50% probability level.

Table S13. Atomic coordinates (x 10^4) and equivalent isotropic displacement parameters (Å^2 x 10^3) for **L3·$PdCl_2$**. U(eq) is defined as one third of the trace of the orthogonalized Uij tensor.

	x	y	z	U(eq)
Pd(1)	4739(1)	586(1)	5739(1)	17(1)
Cl(1)	4823(1)	781(1)	7092(1)	26(1)
Cl(2)	4136(1)	-1351(1)	5717(1)	29(1)
P(1)	4720(1)	3701(1)	5517(1)	15(1)
P(2)	4908(1)	1112(1)	4614(1)	15(1)
C(1)	5215(2)	2283(3)	5290(2)	16(1)
C(2)	6007(2)	2331(4)	5470(2)	19(1)
C(3)	6321(2)	1574(4)	6049(2)	26(1)
C(4)	7044(2)	1614(5)	6228(3)	31(1)

C(5)	7462(2)	2395(5)	5837(3)	33(1)
C(6)	7167(2)	3143(4)	5263(3)	31(1)
C(7)	6443(2)	3105(4)	5081(3)	26(1)
C(8)	5355(2)	4670(4)	6106(2)	18(1)
C(9)	5531(2)	4021(4)	6864(2)	20(1)
C(10)	6166(2)	4670(4)	7273(2)	27(1)
C(11)	6022(2)	6083(4)	7382(2)	27(1)
C(12)	5813(2)	6736(4)	6636(2)	28(1)
C(13)	5185(2)	6084(4)	6211(2)	23(1)
C(14)	3982(2)	3263(4)	6055(2)	19(1)
C(15)	3676(2)	4353(3)	6506(2)	21(1)
C(16)	3149(2)	3810(4)	7014(2)	26(1)
C(17)	2560(2)	3099(5)	6573(3)	32(1)
C(18)	2860(2)	2032(4)	6117(2)	27(1)
C(19)	3389(2)	2538(4)	5603(2)	21(1)
C(20)	4401(2)	4602(3)	4675(2)	18(1)
C(21)	3841(2)	5617(4)	4818(2)	22(1)
C(22)	3629(3)	6386(4)	4111(3)	28(1)
C(23)	4274(3)	6967(4)	3794(2)	31(1)
C(24)	4774(3)	5934(4)	3605(2)	28(1)
C(25)	5025(2)	5145(4)	4291(2)	24(1)
C(26)	4156(2)	1482(3)	3945(2)	16(1)
C(27)	3714(2)	268(4)	3788(2)	22(1)
C(28)	3032(2)	602(4)	3319(3)	27(1)
C(29)	3183(2)	1268(4)	2595(2)	28(1)
C(30)	3646(2)	2449(4)	2742(2)	24(1)
C(31)	4328(2)	2102(3)	3211(2)	18(1)
C(32)	5553(2)	215(4)	4113(2)	22(1)
C(33)	6060(2)	1003(4)	3679(2)	26(1)
C(34)	6501(2)	122(5)	3228(3)	34(1)
C(35)	6890(3)	-869(5)	3721(3)	40(1)
C(36)	6388(3)	-1638(4)	4153(3)	38(1)
C(37)	5948(3)	-776(4)	4613(3)	30(1)

Table S14. Anisotropic displacement parameters ($Å^2 \times 10^3$) for **L3·PdCl$_2$**. The anisotropic displacement factor exponent takes the form: $-2\pi^2 [h^2a^{*2}U^{11} + ... + 2 h k a^* b^* U^{12}]$.

	U^{11}	U^{22}	U^{33}	U^{23}	U^{13}	U^{12}
Pd(1)	17(1)	19(1)	16(1)	2(1)	1(1)	-1(1)
Cl(1)	31(1)	29(1)	18(1)	6(1)	0(1)	-2(1)
Cl(2)	37(1)	21(1)	29(1)	1(1)	8(1)	-7(1)
P(1)	13(1)	18(1)	14(1)	-1(1)	2(1)	0(1)
P(2)	13(1)	16(1)	15(1)	0(1)	2(1)	0(1)
C(1)	15(2)	16(2)	16(2)	2(1)	3(1)	-2(1)
C(2)	14(2)	21(2)	22(2)	-5(2)	1(1)	-3(1)
C(3)	21(2)	29(2)	28(2)	-2(2)	1(2)	3(2)

C(4)	17(2)	40(2)	33(2)	-8(2)	-7(2)	5(2)
C(5)	15(2)	43(2)	41(2)	-16(2)	-2(2)	2(2)
C(6)	16(2)	37(2)	41(2)	-12(2)	10(2)	-8(2)
C(7)	20(2)	29(2)	32(2)	-5(2)	6(2)	-3(2)
C(8)	14(2)	21(2)	19(2)	-4(1)	2(1)	-3(1)
C(9)	18(2)	25(2)	18(2)	-1(2)	0(1)	0(1)
C(10)	20(2)	39(2)	22(2)	-4(2)	-2(2)	-2(2)
C(11)	24(2)	34(2)	24(2)	-11(2)	1(2)	-8(2)
C(12)	26(2)	28(2)	29(2)	-4(2)	3(2)	-6(2)
C(13)	20(2)	24(2)	25(2)	-7(2)	2(2)	-2(2)
C(14)	16(2)	24(2)	16(2)	4(1)	2(1)	-1(1)
C(15)	18(2)	25(2)	19(2)	-3(1)	4(2)	0(1)
C(16)	22(2)	37(2)	20(2)	-4(2)	5(2)	0(2)
C(17)	21(2)	43(2)	32(2)	-7(2)	13(2)	-8(2)
C(18)	24(2)	32(2)	27(2)	-3(2)	9(2)	-7(2)
C(19)	18(2)	25(2)	19(2)	1(2)	3(2)	-4(1)
C(20)	21(2)	18(2)	16(2)	3(1)	1(2)	1(1)
C(21)	24(2)	23(2)	19(2)	1(1)	2(2)	4(1)
C(22)	37(3)	23(2)	23(2)	3(2)	-3(2)	7(2)
C(23)	50(3)	23(2)	19(2)	3(2)	-2(2)	-1(2)
C(24)	40(3)	26(2)	18(2)	0(2)	5(2)	-9(2)
C(25)	29(2)	26(2)	17(2)	2(2)	6(2)	-6(2)
C(26)	16(2)	15(2)	18(2)	-2(1)	1(1)	0(1)
C(27)	21(2)	20(2)	23(2)	2(2)	-6(2)	-5(1)
C(28)	21(2)	28(2)	30(2)	4(2)	-3(2)	-6(2)
C(29)	26(2)	38(2)	20(2)	1(2)	-7(2)	-3(2)
C(30)	24(2)	31(2)	17(2)	4(2)	-1(2)	0(2)
C(31)	20(2)	20(2)	15(2)	-3(1)	4(1)	0(1)
C(32)	17(2)	23(2)	25(2)	-6(2)	3(2)	1(1)
C(33)	21(2)	29(2)	29(2)	-1(2)	6(2)	-1(2)
C(34)	23(2)	45(3)	34(2)	-7(2)	6(2)	4(2)
C(35)	27(2)	41(2)	54(3)	-11(2)	8(2)	8(2)
C(36)	33(3)	26(2)	57(3)	-1(2)	7(2)	8(2)
C(37)	30(2)	24(2)	37(2)	2(2)	3(2)	5(2)

4.2.6 Crystal Structure Determination of **L4·PdCl₂**

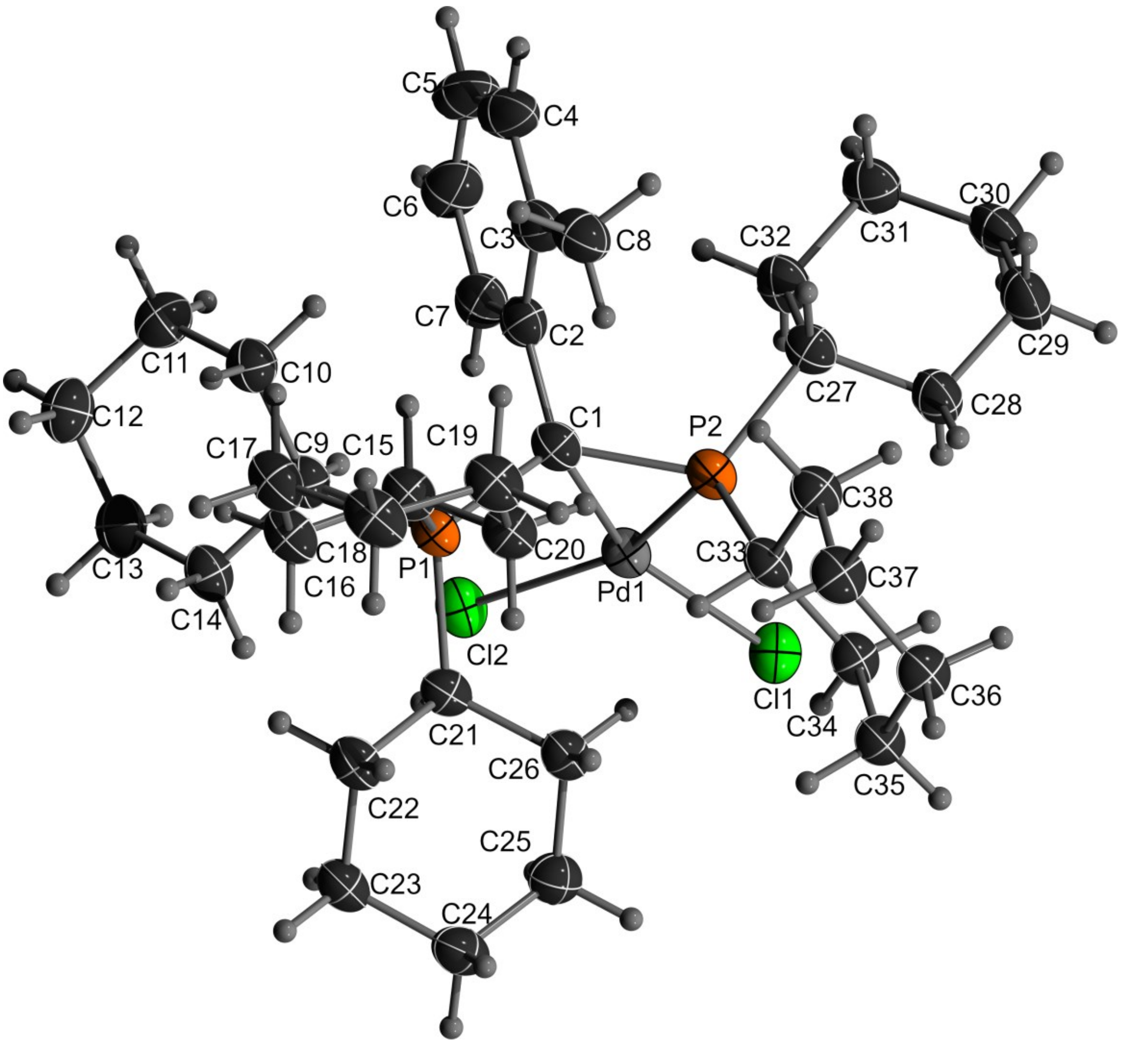

Figure S45. ORTEP Plot of **L4·PdCl$_2$**. Ellipsoids are drawn at the 50% probability level.

Table S15. Atomic coordinates (x 10^4) and equivalent isotropic displacement parameters (Å^2 x 10^3) for **L4·PdCl$_2$**. U(eq) is defined as one third of the trace of the orthogonalized Uij tensor.

	x	y	z	U(eq)
Pd(1)	5892(1)	5666(1)	2031(1)	29(1)
Cl(1)	4571(1)	6147(1)	1050(1)	37(1)
P(1)	6849(1)	4271(1)	3223(1)	29(1)
C(1)	7297(3)	4887(2)	2580(2)	31(1)
P(2)	6897(1)	4767(1)	1667(1)	30(1)
Cl(2)	5254(1)	6445(1)	2882(1)	35(1)
C(2)	8330(3)	5413(2)	2864(2)	35(1)
C(3)	9364(3)	5078(3)	2895(2)	41(1)
C(4)	10264(3)	5632(3)	3142(2)	51(1)
C(5)	10158(3)	6484(3)	3355(2)	55(1)
C(7)	8238(3)	6287(2)	3077(2)	40(1)
C(6)	9148(3)	6809(3)	3326(2)	49(1)

C(8)	9576(3)	4161(3)	2685(2)	44(1)
C(9)	7190(3)	5023(2)	3972(2)	32(1)
C(10)	8385(3)	4991(2)	4404(2)	36(1)
C(11)	8625(3)	5757(2)	4913(2)	44(1)
C(12)	7874(3)	5740(3)	5375(2)	46(1)
C(13)	6697(3)	5745(2)	4954(2)	42(1)
C(14)	6443(3)	4978(2)	4446(2)	36(1)
C(15)	7646(3)	3252(2)	3469(2)	34(1)
C(16)	7559(3)	2790(2)	4130(2)	36(1)
C(17)	8390(3)	2037(2)	4310(2)	42(1)
C(18)	8190(3)	1357(2)	3740(2)	43(1)
C(19)	8219(3)	1791(2)	3070(2)	40(1)
C(20)	7426(3)	2570(2)	2889(2)	36(1)
C(21)	5364(2)	4103(2)	3012(2)	30(1)
C(22)	5042(3)	3440(2)	3495(2)	34(1)
C(23)	3841(3)	3544(2)	3444(2)	37(1)
C(24)	3145(3)	3375(2)	2717(2)	41(1)
C(25)	3503(3)	3969(2)	2214(2)	39(1)
C(26)	4714(3)	3875(2)	2279(2)	33(1)
C(27)	7803(3)	5242(2)	1194(2)	34(1)
C(28)	7225(3)	5180(3)	426(2)	41(1)
C(29)	7925(4)	5548(3)	-6(2)	51(1)
C(30)	8238(3)	6505(3)	174(2)	44(1)
C(31)	8779(3)	6595(3)	933(2)	44(1)
C(32)	8083(3)	6218(2)	1364(2)	40(1)
C(33)	6406(3)	3673(2)	1317(2)	32(1)
C(34)	5366(3)	3723(2)	720(2)	32(1)
C(35)	4917(3)	2790(2)	547(2)	34(1)
C(36)	5755(3)	2169(2)	396(2)	37(1)
C(37)	6797(3)	2147(2)	983(2)	39(1)
C(38)	7260(3)	3078(2)	1142(2)	36(1)

Table S16. Anisotropic displacement parameters ($Å^2$ x 10^3) for **L4·PdCl$_2$**. The anisotropic displacement factor exponent takes the form: $-2\pi^2$ [$h^2a^{*2}U^{11}$ + ... + 2 h k a·b·U^{12}].

	U^{11}	U^{22}	U^{33}	U^{23}	U^{13}	U^{12}
Pd(1)	36(1)	20(1)	33(1)	0(1)	13(1)	1(1)
Cl(1)	45(1)	28(1)	36(1)	1(1)	11(1)	7(1)
P(1)	34(1)	22(1)	33(1)	2(1)	14(1)	1(1)
C(1)	36(2)	24(1)	35(2)	3(1)	13(1)	1(1)
P(2)	35(1)	23(1)	34(1)	1(1)	15(1)	2(1)
Cl(2)	48(1)	24(1)	37(1)	0(1)	17(1)	5(1)
C(2)	38(2)	35(2)	31(2)	6(1)	10(1)	-3(1)
C(3)	40(2)	45(2)	38(2)	13(2)	11(1)	-3(2)
C(4)	39(2)	60(3)	51(2)	19(2)	7(2)	-8(2)
C(5)	44(2)	57(2)	58(2)	12(2)	4(2)	-21(2)
C(7)	48(2)	33(2)	39(2)	7(1)	12(1)	-6(1)
C(6)	58(2)	37(2)	49(2)	4(2)	8(2)	-17(2)
C(8)	38(2)	49(2)	49(2)	12(2)	17(2)	6(2)

C(9)	39(2)	26(1)	32(2)	1(1)	12(1)	1(1)
C(10)	40(2)	32(2)	37(2)	4(1)	11(1)	1(1)
C(11)	43(2)	41(2)	45(2)	-1(2)	8(2)	-1(2)
C(12)	55(2)	43(2)	40(2)	-5(2)	11(2)	4(2)
C(13)	51(2)	40(2)	37(2)	-4(1)	15(2)	4(2)
C(14)	44(2)	31(2)	36(2)	1(1)	17(1)	2(1)
C(15)	38(2)	27(2)	40(2)	3(1)	16(1)	4(1)
C(16)	44(2)	26(2)	43(2)	6(1)	20(1)	5(1)
C(17)	46(2)	39(2)	46(2)	13(2)	21(2)	12(2)
C(18)	48(2)	30(2)	57(2)	9(2)	26(2)	10(1)
C(19)	44(2)	31(2)	49(2)	3(1)	20(2)	8(1)
C(20)	39(2)	27(2)	42(2)	1(1)	15(1)	4(1)
C(21)	33(2)	22(1)	37(2)	-2(1)	14(1)	0(1)
C(22)	44(2)	24(1)	40(2)	2(1)	19(1)	1(1)
C(23)	43(2)	32(2)	42(2)	-1(1)	23(1)	-2(1)
C(24)	39(2)	40(2)	50(2)	-7(2)	22(2)	-7(1)
C(25)	37(2)	39(2)	42(2)	-2(2)	12(1)	-1(1)
C(26)	39(2)	28(2)	36(2)	-2(1)	16(1)	-2(1)
C(27)	37(2)	30(2)	38(2)	4(1)	13(1)	1(1)
C(28)	49(2)	41(2)	38(2)	1(1)	18(2)	-8(2)
C(29)	66(3)	53(2)	40(2)	2(2)	25(2)	-10(2)
C(30)	47(2)	46(2)	45(2)	11(2)	22(2)	-4(2)
C(31)	48(2)	38(2)	48(2)	7(2)	16(2)	-5(2)
C(32)	52(2)	31(2)	40(2)	3(1)	18(2)	-4(2)
C(33)	38(2)	26(1)	36(2)	-1(1)	18(1)	0(1)
C(34)	40(2)	24(1)	34(2)	-2(1)	15(1)	2(1)
C(35)	38(2)	30(2)	37(2)	-2(1)	14(1)	-1(1)
C(36)	46(2)	26(2)	42(2)	-7(1)	17(2)	0(1)
C(37)	44(2)	30(2)	45(2)	-2(1)	16(1)	8(1)
C(38)	37(2)	34(2)	39(2)	-3(1)	15(1)	4(1)

4.2.7 Crystal Structure Determination of **L1H·PdCl₃**

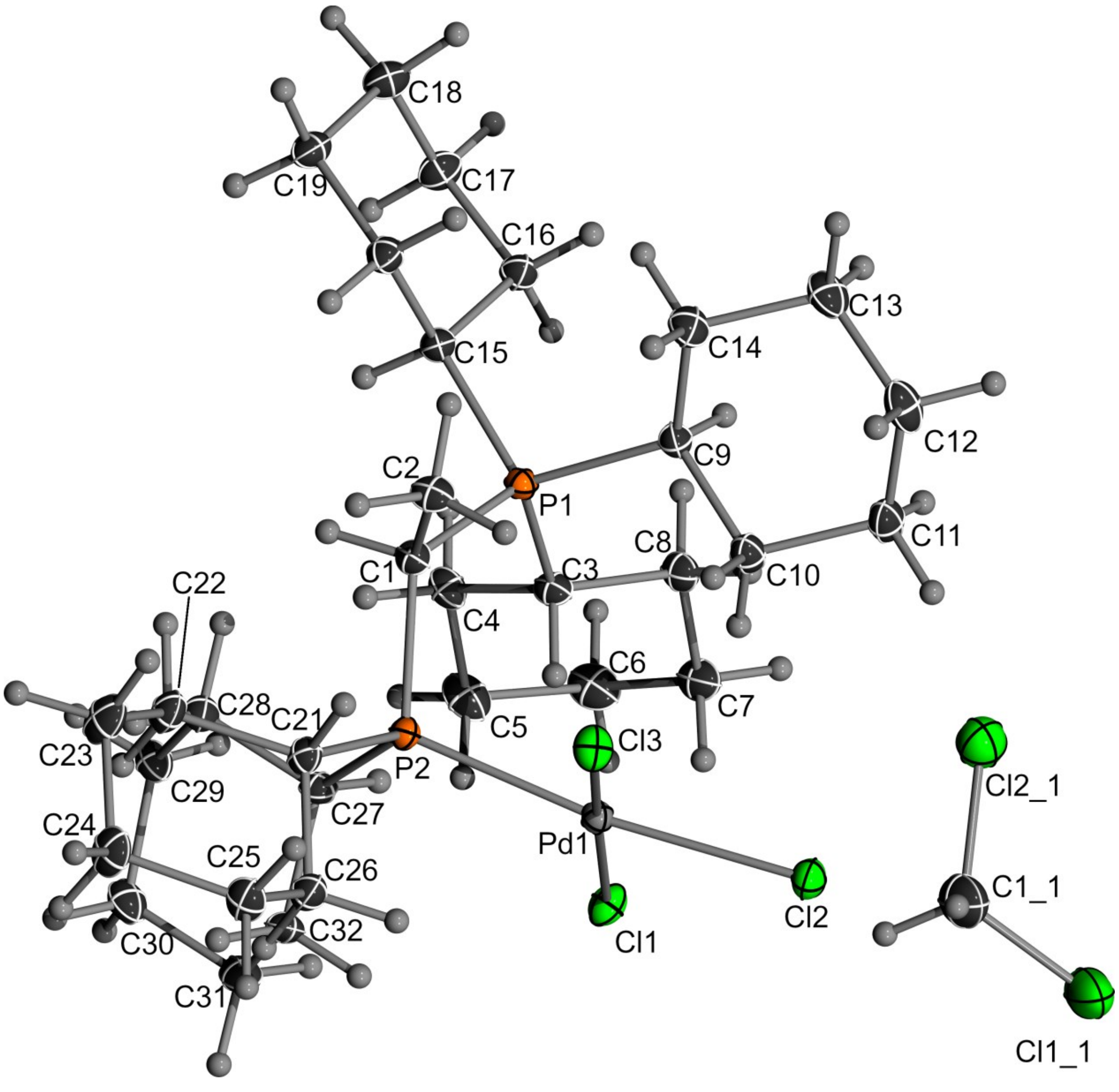

Figure S46. ORTEP Plot of **L1H·PdCl₃**. Ellipsoids are drawn at the 50% probability level.

Table S17. Atomic coordinates (x 10^4) and equivalent isotropic displacement parameters (Å^2 x 10^3) for **L1H·PdCl₃**. U(eq) is defined as one third of the trace of the orthogonalized Uij tensor.

	x	y	z	U(eq)
Pd(1)	3597(1)	5116(1)	7547(1)	13(1)
Cl(1)	1682(1)	4801(1)	8013(1)	19(1)
Cl(2)	3517(1)	6439(1)	7900(1)	18(1)
Cl(3)	5393(1)	5536(1)	7038(1)	19(1)
P(1)	5631(1)	3390(1)	8436(1)	12(1)
P(2)	3925(1)	3843(1)	7274(1)	13(1)
C(1)	5457(2)	3384(1)	7666(1)	14(1)
C(2)	6798(2)	3704(1)	7444(1)	18(1)

C(3)	3949(2)	3420(1)	8736(1)	15(1)
C(4)	3255(2)	2598(1)	8746(1)	18(1)
C(5)	1760(2)	2700(1)	8880(1)	24(1)
C(6)	1651(2)	3112(2)	9442(1)	24(1)
C(7)	2399(2)	3904(1)	9451(1)	22(1)
C(8)	3893(2)	3811(1)	9315(1)	18(1)
C(9)	6584(2)	4232(1)	8750(1)	14(1)
C(10)	5931(2)	5037(1)	8586(1)	18(1)
C(11)	6501(2)	5686(1)	8979(1)	21(1)
C(12)	8051(2)	5731(1)	8976(1)	24(1)
C(13)	8710(2)	4926(1)	9115(1)	23(1)
C(14)	8147(2)	4272(1)	8722(1)	21(1)
C(15)	6469(2)	2448(1)	8635(1)	14(1)
C(16)	6706(2)	2381(1)	9270(1)	16(1)
C(17)	7188(2)	1542(1)	9426(1)	20(1)
C(18)	8499(2)	1333(1)	9154(1)	21(1)
C(19)	8312(2)	1427(1)	8526(1)	19(1)
C(20)	7795(2)	2252(1)	8351(1)	17(1)
C(21)	4375(2)	3822(1)	6539(1)	15(1)
C(22)	4548(2)	2997(1)	6276(1)	19(1)
C(23)	5126(2)	3087(1)	5705(1)	25(1)
C(24)	4210(2)	3613(1)	5326(1)	23(1)
C(25)	3980(2)	4424(1)	5590(1)	20(1)
C(26)	3399(2)	4332(1)	6161(1)	18(1)
C(27)	2514(2)	3133(1)	7363(1)	15(1)
C(28)	2813(2)	2237(1)	7301(1)	17(1)
C(29)	1590(2)	1744(1)	7470(1)	19(1)
C(30)	279(2)	1978(1)	7136(1)	21(1)
C(31)	20(2)	2872(1)	7174(1)	21(1)
C(32)	1235(2)	3352(1)	6993(1)	17(1)
Cl11	990(1)	7155(1)	9437(1)	47(1)
Cl21	3193(1)	5997(1)	9576(1)	34(1)
C11	1956(3)	6444(2)	9112(1)	36(1)

Table S18. Anisotropic displacement parameters ($Å^2$ x 10^3) for **L1H·PdCl₃**. The anisotropic displacement factor exponent takes the form: $-2\pi^2$ [$h^2a^{*2}U^{11}$ + ... + 2 h k a·b·U^{12}].

	U^{11}	U^{22}	U^{33}	U^{23}	U^{13}	U^{12}
Pd(1)	15(1)	13(1)	12(1)	0(1)	1(1)	1(1)
Cl(1)	19(1)	18(1)	20(1)	-1(1)	7(1)	2(1)
Cl(2)	23(1)	14(1)	18(1)	-2(1)	1(1)	1(1)
Cl(3)	21(1)	18(1)	19(1)	0(1)	4(1)	-3(1)
P(1)	11(1)	14(1)	12(1)	0(1)	1(1)	0(1)
P(2)	12(1)	14(1)	12(1)	-1(1)	1(1)	1(1)
C(1)	13(1)	16(1)	13(1)	0(1)	2(1)	0(1)
C(2)	13(1)	24(1)	16(1)	1(1)	3(1)	0(1)

C(3)	12(1)	20(1)	13(1)	0(1)	2(1)	1(1)
C(4)	17(1)	21(1)	16(1)	-1(1)	1(1)	-4(1)
C(5)	17(1)	34(1)	22(1)	-2(1)	4(1)	-8(1)
C(6)	17(1)	38(1)	19(1)	0(1)	6(1)	-2(1)
C(7)	19(1)	32(1)	14(1)	-2(1)	3(1)	4(1)
C(8)	18(1)	23(1)	13(1)	-2(1)	2(1)	1(1)
C(9)	16(1)	14(1)	14(1)	-1(1)	0(1)	-1(1)
C(10)	22(1)	15(1)	16(1)	0(1)	-2(1)	-1(1)
C(11)	29(1)	14(1)	20(1)	-1(1)	-2(1)	-1(1)
C(12)	30(1)	20(1)	21(1)	0(1)	0(1)	-10(1)
C(13)	18(1)	26(1)	24(1)	-3(1)	0(1)	-7(1)
C(14)	16(1)	21(1)	25(1)	-3(1)	2(1)	-3(1)
C(15)	14(1)	14(1)	15(1)	-1(1)	0(1)	1(1)
C(16)	18(1)	16(1)	15(1)	1(1)	0(1)	2(1)
C(17)	22(1)	16(1)	21(1)	4(1)	2(1)	1(1)
C(18)	20(1)	20(1)	24(1)	3(1)	1(1)	4(1)
C(19)	18(1)	17(1)	23(1)	0(1)	2(1)	4(1)
C(20)	16(1)	17(1)	18(1)	-1(1)	2(1)	1(1)
C(21)	17(1)	17(1)	12(1)	-1(1)	3(1)	2(1)
C(22)	25(1)	17(1)	15(1)	-2(1)	3(1)	5(1)
C(23)	33(1)	26(1)	18(1)	-2(1)	8(1)	9(1)
C(24)	32(1)	24(1)	12(1)	-2(1)	6(1)	4(1)
C(25)	28(1)	20(1)	13(1)	2(1)	2(1)	2(1)
C(26)	22(1)	17(1)	15(1)	0(1)	1(1)	4(1)
C(27)	12(1)	17(1)	15(1)	0(1)	1(1)	1(1)
C(28)	14(1)	17(1)	18(1)	-2(1)	1(1)	-1(1)
C(29)	17(1)	20(1)	19(1)	-2(1)	1(1)	-3(1)
C(30)	16(1)	26(1)	21(1)	-5(1)	2(1)	-5(1)
C(31)	14(1)	28(1)	21(1)	-5(1)	2(1)	1(1)
C(32)	14(1)	21(1)	16(1)	-1(1)	-3(1)	4(1)
Cl11	39(1)	60(1)	40(1)	-24(1)	-11(1)	16(1)
Cl21	42(1)	29(1)	31(1)	-1(1)	-3(1)	4(1)
C11	36(1)	46(2)	24(1)	-9(1)	0(1)	6(1)

4.2.8 Crystal Structure Determination of **L2H·PdCl₃**

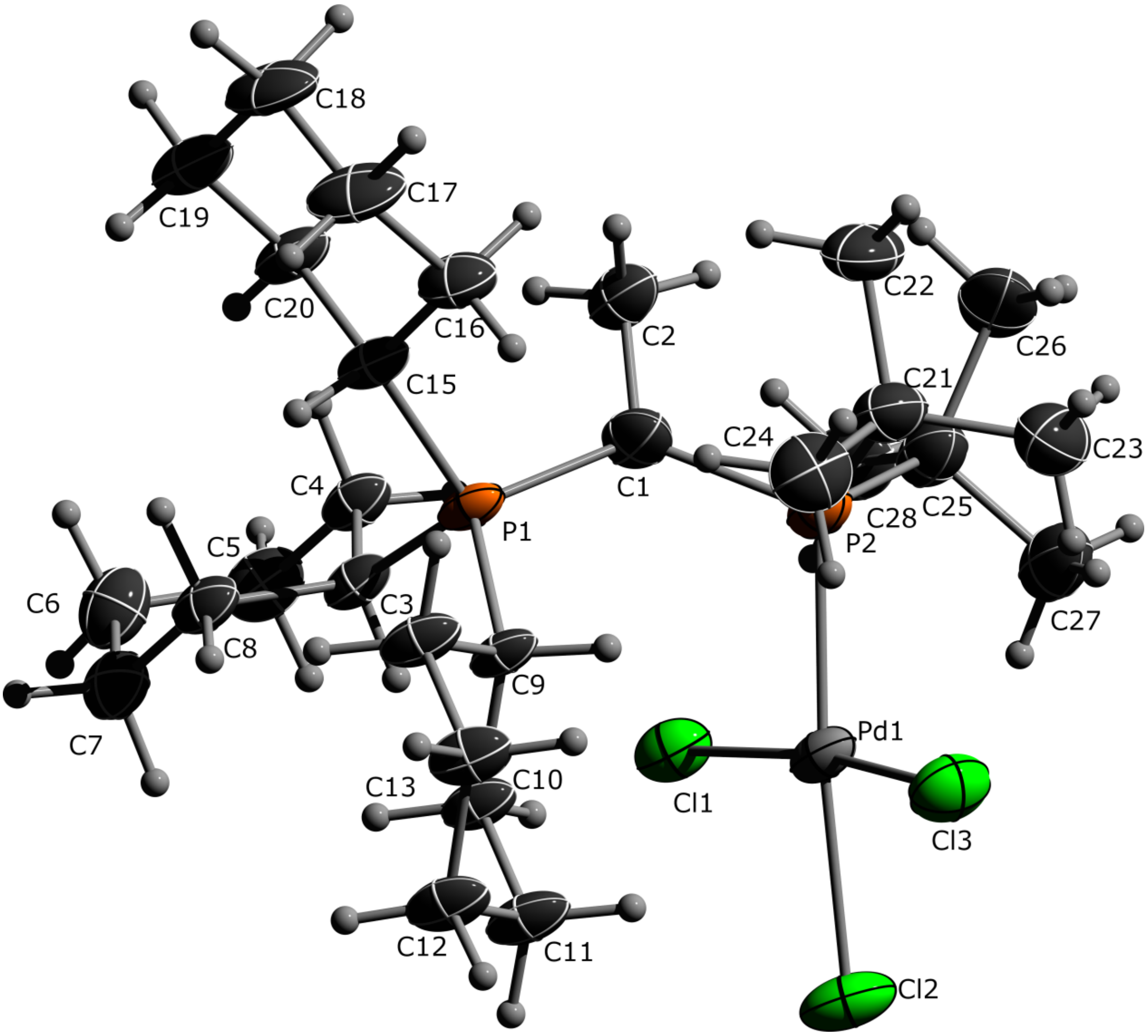

Figure S47. ORTEP Plot of **L2H·PdCl₃**. Ellipsoids are drawn at the 50% probability level.

Table S19. Atomic coordinates (x 10^4) and equivalent isotropic displacement parameters (Å^2 x 10^3) for **L2H·PdCl₃**. U(eq) is defined as one third of the trace of the orthogonalized Uij tensor.

	x	y	z	U(eq)
Pd(1)	7055(1)	6618(1)	3895(1)	27(1)
Cl(1)	5926(1)	6206(1)	2800(1)	34(1)
Cl(2)	6526(1)	5680(1)	4657(1)	44(1)
Cl(3)	8022(1)	7066(1)	5068(1)	45(1)
P(1)	6004(1)	8694(1)	2447(1)	22(1)
P(2)	7627(1)	7344(1)	3079(1)	30(1)
C(1)	6828(4)	7991(4)	2297(3)	36(1)
C(2)	6979(4)	8175(4)	1580(3)	37(1)
C(3)	4982(3)	8211(3)	1920(3)	26(1)

C(4)	4879(3)	7783(3)	1121(3)	28(1)
C(5)	4014(4)	7358(4)	792(3)	39(1)
C(6)	3324(4)	8028(4)	729(4)	45(1)
C(7)	3436(4)	8461(4)	1528(3)	37(1)
C(8)	4286(3)	8890(3)	1860(3)	30(1)
C(9)	6080(3)	8761(3)	3497(2)	23(1)
C(10)	5471(3)	8131(3)	3712(3)	28(1)
C(11)	5658(4)	8129(3)	4602(3)	35(1)
C(12)	5573(4)	9065(3)	4885(3)	34(1)
C(13)	6162(4)	9698(3)	4670(3)	34(1)
C(14)	5981(4)	9713(3)	3782(3)	30(1)
C(15)	6049(3)	9842(3)	2084(3)	27(1)
C(16)	6887(4)	10324(3)	2419(3)	38(1)
C(17)	6781(5)	11319(4)	2238(4)	52(2)
C(18)	6424(5)	11468(4)	1348(4)	55(2)
C(19)	5616(4)	10981(4)	998(3)	44(2)
C(20)	5711(4)	9981(3)	1187(3)	34(1)
C(21)	8535(4)	8144(4)	3520(3)	42(1)
C(22)	8780(4)	8681(5)	2911(4)	50(2)
C(23)	9316(4)	7635(5)	4055(4)	56(2)
C(24)	8270(4)	8829(5)	4031(4)	50(2)
C(25)	7996(4)	6406(4)	2549(3)	43(1)
C(26)	8695(4)	6661(5)	2225(4)	56(2)
C(27)	8339(5)	5671(5)	3179(4)	57(2)
C(28)	7294(4)	5996(4)	1875(4)	49(2)

Table S20. Anisotropic displacement parameters (Å^2 x 10^3) for **L2H·PdCl$_3$**. The anisotropic displacement factor exponent takes the form: $-2\pi^2$ [$h^2a^{*2}U^{11}$ + ... + 2 h k a·b·U^{12}].

	U^{11}	U^{22}	U^{33}	U^{23}	U^{13}	U^{12}
Pd(1)	44(1)	21(1)	21(1)	3(1)	15(1)	4(1)
Cl(1)	52(1)	25(1)	27(1)	-3(1)	16(1)	-1(1)
Cl(2)	76(1)	33(1)	28(1)	-1(1)	25(1)	-14(1)
Cl(3)	51(1)	54(1)	27(1)	4(1)	9(1)	-5(1)
P(1)	42(1)	15(1)	14(1)	1(1)	16(1)	3(1)
P(2)	41(1)	29(1)	24(1)	5(1)	16(1)	6(1)
C(1)	51(3)	37(3)	30(3)	4(2)	25(2)	13(3)
C(2)	54(3)	28(3)	35(3)	3(2)	20(3)	12(2)
C(3)	43(3)	17(2)	18(2)	2(2)	10(2)	4(2)
C(4)	48(3)	21(2)	18(2)	1(2)	15(2)	5(2)
C(5)	54(3)	29(3)	30(3)	-5(2)	8(2)	-5(2)
C(6)	48(3)	35(3)	48(3)	2(3)	8(3)	1(3)
C(7)	45(3)	26(3)	43(3)	4(2)	19(3)	8(2)
C(8)	47(3)	17(2)	32(3)	3(2)	20(2)	7(2)
C(9)	44(3)	17(2)	12(2)	0(2)	14(2)	2(2)
C(10)	51(3)	18(2)	19(2)	0(2)	19(2)	0(2)

C(11)	67(4)	24(2)	22(2)	1(2)	26(2)	1(2)
C(12)	66(4)	25(2)	20(2)	-2(2)	26(2)	1(2)
C(13)	60(3)	23(2)	23(2)	-7(2)	20(2)	-5(2)
C(14)	54(3)	22(2)	19(2)	-2(2)	17(2)	-4(2)
C(15)	52(3)	16(2)	20(2)	1(2)	20(2)	1(2)
C(16)	57(3)	24(3)	39(3)	5(2)	25(3)	-5(2)
C(17)	84(5)	26(3)	57(4)	5(3)	38(4)	-14(3)
C(18)	93(5)	24(3)	65(4)	18(3)	50(4)	2(3)
C(19)	79(4)	29(3)	34(3)	11(2)	32(3)	9(3)
C(20)	62(3)	24(2)	22(2)	4(2)	23(2)	4(2)
C(21)	44(3)	51(3)	33(3)	9(3)	16(2)	-4(3)
C(22)	48(3)	55(4)	52(4)	15(3)	21(3)	-14(3)
C(23)	42(3)	73(5)	52(4)	15(3)	17(3)	-1(3)
C(24)	47(3)	50(4)	46(3)	-5(3)	9(3)	-8(3)
C(25)	58(4)	42(3)	35(3)	6(2)	25(3)	16(3)
C(26)	65(4)	75(5)	42(3)	10(3)	37(3)	24(4)
C(27)	76(5)	49(4)	58(4)	17(3)	38(4)	32(3)
C(28)	76(4)	36(3)	48(3)	-11(3)	38(3)	10(3)

4.2.9 Crystal Structure Determination of **L3H·PdCl$_3$**

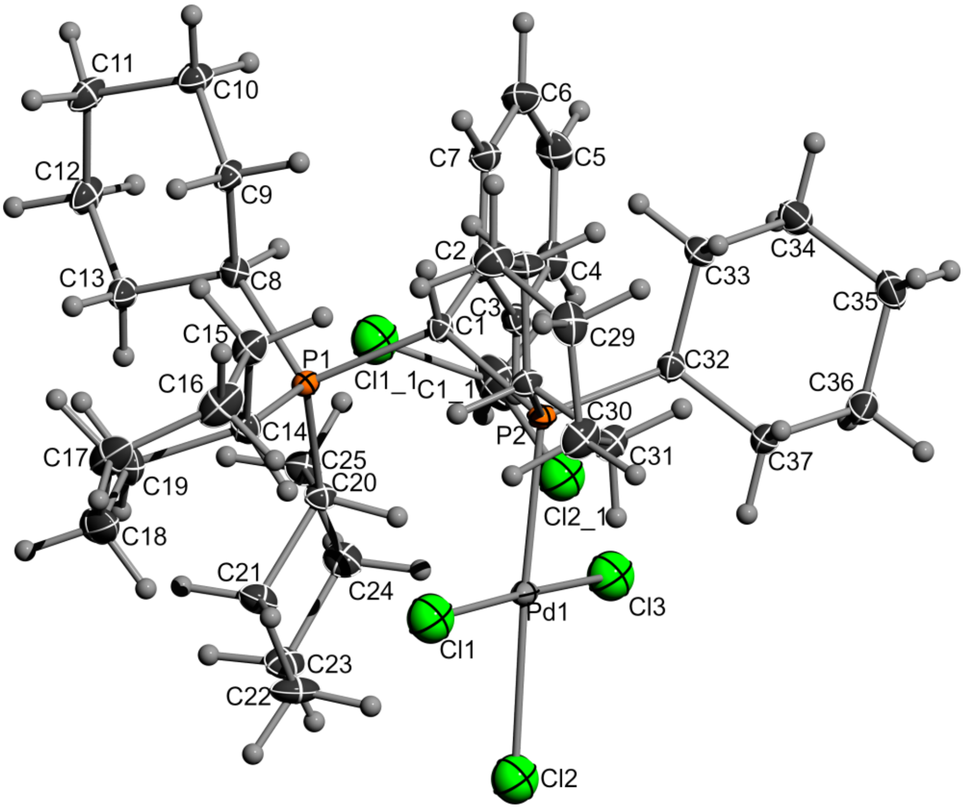

Figure S48. ORTEP Plot of **L3H·PdCl$_3$**. Ellipsoids are drawn at the 50% probability level.

Table S21. Atomic coordinates (x 10^4) and equivalent isotropic displacement parameters ($Å^2$ x 10^3) for **L3H·PdCl$_3$**. U(eq) is defined as one third of the trace of the orthogonalized Uij tensor.

	x	y	z	U(eq)
Pd(1)	2533(1)	5435(1)	2645(1)	13(1)
Cl(1)	1648(1)	6448(1)	3156(1)	20(1)
P(1)	4117(1)	5431(1)	4494(1)	11(1)
C(1)	3206(2)	4615(1)	4364(1)	12(1)
Cl(2)	2903(1)	6175(1)	1584(1)	21(1)
P(2)	2136(1)	4691(1)	3628(1)	11(1)
C(2)	3832(2)	3878(1)	4354(1)	12(1)
Cl(3)	3504(1)	4477(1)	2110(1)	20(1)
C(3)	4472(2)	3662(2)	3760(2)	16(1)
C(4)	5033(2)	2992(2)	3784(2)	20(1)
C(5)	4965(2)	2522(2)	4394(2)	23(1)
C(6)	4322(2)	2727(2)	4987(2)	22(1)
C(7)	3761(2)	3402(1)	4966(2)	16(1)
C(8)	4961(2)	5130(2)	5260(2)	14(1)
C(9)	4319(2)	4967(2)	5966(1)	15(1)
C(10)	5013(2)	4536(2)	6510(1)	18(1)
C(11)	5995(2)	4986(2)	6712(2)	20(1)
C(12)	6610(2)	5220(2)	6023(2)	21(1)
C(13)	5901(2)	5641(2)	5470(2)	18(1)
C(14)	3334(2)	6268(1)	4716(2)	16(1)
C(15)	2488(2)	6176(1)	5326(1)	18(1)
C(16)	1747(3)	6860(2)	5317(2)	26(1)
C(17)	2364(3)	7590(2)	5422(2)	27(1)
C(18)	3294(3)	7659(2)	4886(2)	30(1)
C(19)	4009(3)	6967(2)	4920(2)	23(1)
C(20)	4876(2)	5568(1)	3630(1)	14(1)
C(21)	4967(2)	6383(2)	3344(2)	19(1)
C(22)	5427(2)	6384(2)	2560(2)	24(1)
C(23)	6492(2)	5985(2)	2520(2)	22(1)
C(24)	6402(2)	5189(2)	2827(2)	21(1)
C(25)	5982(2)	5196(2)	3618(2)	18(1)
C(26)	966(2)	5031(1)	4144(1)	14(1)
C(27)	649(2)	4646(2)	4880(1)	18(1)
C(28)	-219(2)	5112(2)	5253(2)	22(1)
C(29)	-1184(2)	5212(2)	4754(2)	23(1)
C(30)	-878(2)	5530(2)	4001(2)	23(1)
C(31)	-12(2)	5061(2)	3634(2)	18(1)
C(32)	1911(2)	3706(1)	3332(1)	15(1)
C(33)	1506(2)	3157(1)	3923(2)	17(1)
C(34)	1615(2)	2354(2)	3626(2)	21(1)
C(35)	973(2)	2255(2)	2912(2)	22(1)
C(36)	1274(2)	2848(2)	2338(2)	21(1)

C(37)	1193(2)	3644(2)	2646(2)	18(1)
Cl11	7902(1)	3539(1)	3552(1)	36(1)
Cl21	6777(1)	3285(1)	2160(1)	43(1)
C11	7786(3)	2968(2)	2758(2)	38(1)

Table S22. Anisotropic displacement parameters (Å^2 x 10^3) for **L3H·PdCl$_3$**. The anisotropic displacement factor exponent takes the form: $-2\pi^2$ [$h^2a^{*2}U^{11}$ + ... + 2 h k $a^*b^*U^{12}$].

	U^{11}	U^{22}	U^{33}	U^{23}	U^{13}	U^{12}
Pd(1)	13(1)	16(1)	11(1)	3(1)	-1(1)	-3(1)
Cl(1)	25(1)	17(1)	18(1)	3(1)	-1(1)	1(1)
P(1)	11(1)	12(1)	11(1)	0(1)	-1(1)	0(1)
C(1)	10(1)	14(1)	13(1)	-1(1)	0(1)	-1(1)
Cl(2)	21(1)	25(1)	15(1)	8(1)	-2(1)	-6(1)
P(2)	11(1)	13(1)	10(1)	2(1)	1(1)	-1(1)
C(2)	11(1)	12(1)	14(1)	-1(1)	-2(1)	-1(1)
Cl(3)	21(1)	24(1)	15(1)	0(1)	4(1)	1(1)
C(3)	15(1)	16(1)	16(1)	-1(1)	0(1)	-2(1)
C(4)	17(1)	21(1)	22(1)	-5(1)	1(1)	1(1)
C(5)	22(1)	16(1)	31(2)	-1(1)	-4(1)	5(1)
C(6)	24(2)	20(1)	21(1)	5(1)	-2(1)	1(1)
C(7)	17(1)	16(1)	15(1)	1(1)	-1(1)	1(1)
C(8)	12(1)	14(1)	14(1)	-1(1)	0(1)	1(1)
C(9)	13(1)	21(1)	12(1)	-1(1)	1(1)	-2(1)
C(10)	16(1)	24(1)	15(1)	3(1)	1(1)	-1(1)
C(11)	14(1)	31(2)	14(1)	0(1)	-2(1)	2(1)
C(12)	13(1)	32(2)	18(1)	-1(1)	-3(1)	-4(1)
C(13)	17(1)	24(1)	15(1)	1(1)	-1(1)	-6(1)
C(14)	17(1)	14(1)	17(1)	-2(1)	0(1)	2(1)
C(15)	18(1)	20(1)	17(1)	-3(1)	0(1)	6(1)
C(16)	24(1)	32(2)	22(1)	-5(1)	-1(1)	15(1)
C(17)	41(2)	21(1)	20(1)	-5(1)	-3(1)	16(1)
C(18)	50(2)	16(1)	23(2)	-1(1)	0(1)	2(1)
C(19)	28(2)	17(1)	24(1)	-6(1)	0(1)	-1(1)
C(20)	12(1)	16(1)	14(1)	2(1)	2(1)	-1(1)
C(21)	16(1)	18(1)	23(1)	6(1)	1(1)	0(1)
C(22)	19(1)	32(2)	22(1)	14(1)	4(1)	2(1)
C(23)	18(1)	28(1)	21(1)	6(1)	4(1)	-4(1)
C(24)	19(1)	23(1)	21(1)	0(1)	6(1)	1(1)
C(25)	14(1)	19(1)	19(1)	1(1)	2(1)	1(1)
C(26)	13(1)	16(1)	13(1)	1(1)	1(1)	1(1)
C(27)	18(1)	22(1)	14(1)	5(1)	4(1)	1(1)
C(28)	19(2)	27(1)	19(1)	1(1)	6(1)	0(1)
C(29)	14(1)	27(1)	27(2)	-4(1)	5(1)	-2(1)
C(30)	13(1)	31(2)	24(1)	-1(1)	-1(1)	4(1)
C(31)	10(1)	27(1)	17(1)	-1(1)	1(1)	-2(1)

C(32)	14(1)	15(1)	15(1)	-1(1)	-2(1)	-1(1)
C(33)	17(1)	18(1)	16(1)	2(1)	0(1)	-3(1)
C(34)	22(1)	17(1)	25(1)	2(1)	-1(1)	-1(1)
C(35)	25(1)	17(1)	26(1)	-3(1)	1(1)	-5(1)
C(36)	24(1)	24(1)	16(1)	-3(1)	-1(1)	-4(1)
C(37)	21(1)	20(1)	14(1)	1(1)	-4(1)	-5(1)
Cl11	35(1)	27(1)	47(1)	-8(1)	-7(1)	2(1)
Cl21	74(1)	32(1)	24(1)	-3(1)	4(1)	-6(1)
C11	43(2)	24(2)	47(2)	-11(1)	11(2)	1(1)

4.2.10 Crystal Structure Determination of **L4H·PdCl$_3$**

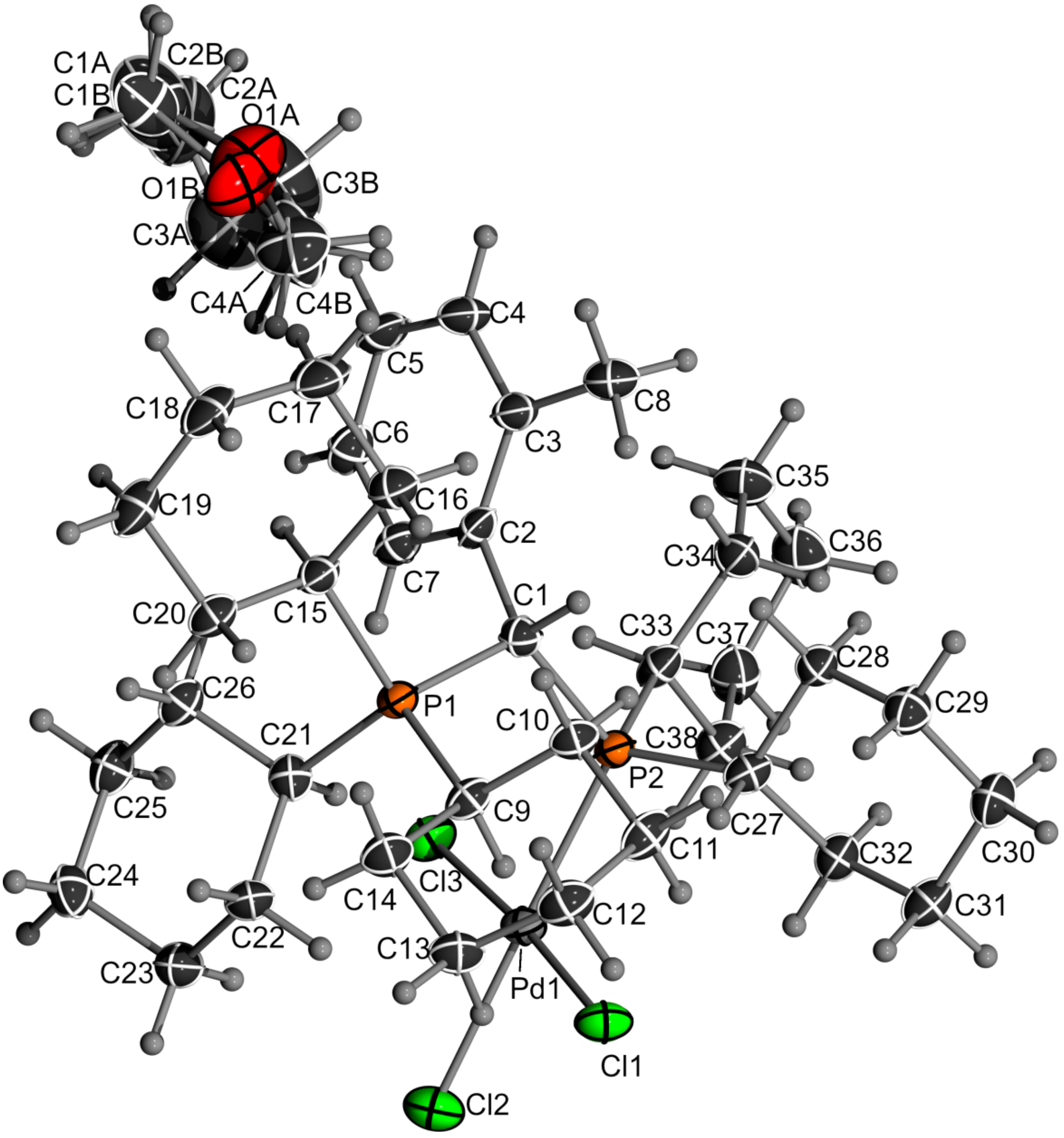

Figure S49. ORTEP Plot of **L4H·PdCl$_3$**. Ellipsoids are drawn at the 50% probability level.

Table S23. Atomic coordinates (x 10^4) and equivalent isotropic displacement parameters ($Å^2$ x 10^3) for **L4H·PdCl$_3$**. U(eq) is defined as one third of the trace of the orthogonalized Uij tensor.

	x	y	z	U(eq)
Pd(1)	2651(1)	4726(1)	4039(1)	22(1)
Cl(1)	3203(1)	3289(1)	3439(1)	30(1)
Cl(2)	2280(1)	5714(1)	3063(1)	34(1)
Cl(3)	2036(1)	6167(1)	4579(1)	28(1)
P(1)	2022(1)	1499(1)	4780(1)	20(1)
P(2)	3026(1)	3824(1)	4992(1)	20(1)
C(1)	2544(2)	2454(5)	5357(2)	22(1)
C(2)	2221(2)	2876(5)	5950(2)	23(1)
C(3)	2358(2)	2270(5)	6551(2)	26(1)
C(4)	2009(2)	2638(5)	7060(2)	30(1)
C(5)	1565(2)	3598(5)	6990(2)	32(1)
C(6)	1448(2)	4241(5)	6399(2)	29(1)
C(7)	1781(2)	3867(5)	5890(2)	26(1)
C(8)	2874(2)	1316(5)	6720(2)	30(1)
C(9)	2500(2)	656(5)	4221(2)	23(1)
C(10)	3030(2)	-140(5)	4565(2)	24(1)
C(11)	3484(2)	-539(5)	4071(2)	28(1)
C(12)	3181(2)	-1377(5)	3529(2)	32(1)
C(13)	2625(2)	-658(5)	3223(2)	29(1)
C(14)	2181(2)	-308(5)	3725(2)	29(1)
C(15)	1593(2)	322(5)	5245(2)	24(1)
C(16)	1963(2)	-655(5)	5691(2)	31(1)
C(17)	1543(2)	-1354(6)	6135(2)	39(1)
C(18)	1044(2)	-2100(6)	5751(2)	38(1)
C(19)	703(2)	-1230(5)	5248(2)	35(1)
C(20)	1138(2)	-543(5)	4819(2)	27(1)
C(21)	1475(2)	2679(5)	4377(2)	22(1)
C(22)	1371(2)	2488(5)	3646(2)	28(1)
C(23)	1000(2)	3661(5)	3353(2)	31(1)
C(24)	391(2)	3783(5)	3655(2)	32(1)
C(25)	498(2)	3961(5)	4387(2)	30(1)
C(26)	861(2)	2788(5)	4683(2)	25(1)
C(27)	3763(2)	2985(5)	4907(2)	24(1)
C(28)	4076(2)	2198(5)	5476(2)	25(1)
C(29)	4608(2)	1408(5)	5236(2)	31(1)
C(30)	5058(2)	2326(5)	4938(2)	32(1)
C(31)	4752(2)	3197(5)	4409(2)	31(1)
C(32)	4223(2)	3979(5)	4652(2)	26(1)
C(33)	3124(2)	5104(5)	5631(2)	25(1)
C(34)	3454(2)	4658(5)	6262(2)	31(1)
C(35)	3381(2)	5689(6)	6781(2)	40(1)
C(36)	3650(2)	7033(6)	6590(2)	42(1)

C(37)	3378(2)	7488(6)	5930(2)	36(1)
C(38)	3425(2)	6416(5)	5415(2)	28(1)
O1A1	652(19)	5450(40)	7998(17)	55(6)
C1A1	111(12)	5900(20)	8263(14)	50(5)
C2A1	170(11)	7430(20)	8359(14)	68(5)
C3A1	471(9)	7780(14)	7866(10)	70(5)
C4A1	880(17)	6570(20)	7698(15)	48(5)
O1B1	630(20)	5430(50)	7860(20)	54(6)
C1B1	98(13)	5460(30)	8197(15)	43(5)
C2B1	44(13)	6940(30)	8373(14)	73(6)
C3B1	718(10)	7620(20)	8187(13)	82(6)
C4B1	880(20)	6800(40)	7740(20)	65(6)

Table S24. Anisotropic displacement parameters (Å^2 x 10^3) for **L4H·PdCl$_3$**. The anisotropic displacement factor exponent takes the form: $-2\pi^2$ [$h^2a^{*2}U^{11}$ + ... + 2 h k a·b·U^{12}].

	U^{11}	U^{22}	U^{33}	U^{23}	U^{13}	U^{12}
Pd(1)	26(1)	21(1)	20(1)	1(1)	4(1)	-3(1)
Cl(1)	38(1)	32(1)	22(1)	-2(1)	7(1)	2(1)
Cl(2)	45(1)	32(1)	23(1)	3(1)	1(1)	1(1)
Cl(3)	31(1)	27(1)	28(1)	-1(1)	7(1)	0(1)
P(1)	21(1)	21(1)	19(1)	0(1)	5(1)	-3(1)
P(2)	22(1)	22(1)	18(1)	1(1)	5(1)	-2(1)
C(1)	22(2)	22(2)	21(2)	2(2)	2(1)	0(2)
C(2)	20(2)	27(3)	23(2)	-2(2)	9(2)	-3(2)
C(3)	29(2)	30(3)	19(2)	2(2)	5(2)	-4(2)
C(4)	37(2)	36(3)	19(2)	3(2)	8(2)	1(2)
C(5)	34(2)	40(3)	22(2)	-4(2)	14(2)	-6(2)
C(6)	26(2)	31(3)	31(2)	-6(2)	8(2)	1(2)
C(7)	29(2)	27(3)	23(2)	-3(2)	7(2)	-4(2)
C(8)	36(2)	32(3)	22(2)	4(2)	6(2)	-1(2)
C(9)	25(2)	17(2)	27(2)	1(2)	7(2)	-2(2)
C(10)	27(2)	22(2)	22(2)	-2(2)	6(2)	1(2)
C(11)	28(2)	23(3)	36(2)	-1(2)	13(2)	4(2)
C(12)	39(3)	29(3)	29(2)	-6(2)	10(2)	-1(2)
C(13)	36(2)	29(3)	23(2)	-1(2)	6(2)	2(2)
C(14)	31(2)	30(3)	27(2)	-2(2)	7(2)	2(2)
C(15)	23(2)	26(3)	23(2)	4(2)	7(2)	-2(2)
C(16)	32(2)	34(3)	29(2)	6(2)	6(2)	-2(2)
C(17)	45(3)	41(3)	34(2)	14(2)	13(2)	-2(2)
C(18)	36(3)	38(3)	44(3)	10(2)	16(2)	-8(2)
C(19)	29(2)	32(3)	47(3)	4(2)	12(2)	-6(2)
C(20)	28(2)	24(3)	29(2)	3(2)	7(2)	-4(2)
C(21)	21(2)	19(2)	25(2)	0(2)	1(2)	0(2)

C(22)	28(2)	35(3)	20(2)	1(2)	5(2)	-1(2)
C(23)	31(2)	38(3)	25(2)	4(2)	3(2)	2(2)
C(24)	26(2)	31(3)	37(2)	4(2)	-2(2)	2(2)
C(25)	25(2)	28(3)	38(2)	-2(2)	6(2)	1(2)
C(26)	25(2)	23(3)	29(2)	-3(2)	6(2)	1(2)
C(27)	26(2)	23(2)	23(2)	1(2)	7(2)	0(2)
C(28)	22(2)	27(3)	26(2)	4(2)	2(2)	-2(2)
C(29)	28(2)	30(3)	34(2)	3(2)	4(2)	1(2)
C(30)	24(2)	34(3)	38(2)	0(2)	4(2)	1(2)
C(31)	30(2)	28(3)	35(2)	2(2)	11(2)	-3(2)
C(32)	23(2)	27(3)	28(2)	0(2)	4(2)	-2(2)
C(33)	23(2)	26(3)	28(2)	0(2)	10(2)	-6(2)
C(34)	29(2)	40(3)	24(2)	-3(2)	2(2)	-7(2)
C(35)	44(3)	53(4)	25(2)	-6(2)	4(2)	0(3)
C(36)	42(3)	47(4)	37(2)	-20(2)	-1(2)	1(3)
C(37)	31(2)	38(3)	39(2)	-8(2)	2(2)	1(2)
C(38)	31(2)	24(3)	29(2)	-4(2)	6(2)	-2(2)
O1A1	64(8)	30(7)	74(15)	8(9)	19(9)	6(5)
C1A1	51(7)	48(11)	52(11)	13(10)	6(6)	13(8)
C2A1	60(9)	46(10)	99(11)	-10(9)	12(8)	11(8)
C3A1	85(11)	42(7)	80(9)	-4(6)	-6(7)	12(7)
C4A1	66(10)	27(8)	53(8)	5(7)	10(7)	-5(7)
O1B1	59(9)	49(9)	59(12)	-9(8)	23(9)	14(7)
C1B1	45(8)	48(11)	37(7)	-16(9)	3(5)	31(8)
C2B1	81(12)	56(13)	79(11)	-46(11)	-10(8)	40(9)
C3B1	84(11)	63(10)	93(12)	-20(9)	-35(9)	19(8)
C4B1	56(11)	50(11)	85(13)	-5(9)	-18(9)	11(9)

5. Computational Studies

5.1 Computational details

Geometry optimizations were performed using the Gaussian 16 optimizer[25] together with TurboMole V7.0.[26] energies and gradients. All geometry optimizations were computed using the functional PBE0[27,28] functional with Grimme dispersion corrections D3[29] and the Becke-Jonson damping function[30] in combination with the def2-SVP basis set.[31] The stationary points were located with the Berny algorithm[32] using redundant internal coordinates. Analytical Hessians were computed to determine the nature of stationary points (one and zero imaginary frequencies for transition states and minima, respectively)[33] and to calculate unscaled zero-point energies (ZPEs) as well as thermal corrections and entropy effects using the standard statistical-mechanics relationships for an ideal gas.

The atomic partial charges were estimated with the natural bond orbital (NBO)[34,35] method using NBO 7.0.[36] The topological quantum theory of atoms in molecules (QTAIM),[37] and Laplacian of the electron density analyses were carried out with AIMAll[38] All these analysis were performed at the PBE0-D3(BJ)/def2-TZVPP//PBE0-D3(BJ)/def2-SVP level of theory.

The nature of the chemical bonds were investigated by means of the Energy Decomposition Analysis (EDA) method, which was developed by Morokuma[39] and by Ziegler and Rauk.[40,41] The bonding analysis focuses on the instantaneous interaction energy ΔE_{int} of a bond A–B between two fragments A and B in the particular electronic reference state and in the frozen geometry AB. This energy is divided into four main components (Eq S1).

$$\Delta E_{\text{int}} = \Delta E_{elst} + \Delta E_{Pauli} + \Delta E_{orb} + \Delta E_{disp} \qquad \text{(S1)}$$

The term ΔE_{elst} corresponds to the quasiclassical electrostatic interaction between the unperturbed charge distributions of the prepared atoms (or fragments) and it is usually attractive. The Pauli repulsion ΔE_{Pauli} is the energy change associated with the transformation from the superposition of the unperturbed wave functions (Slater determinant of the Kohn-Sham orbitals) of the isolated fragments to the wave function $\Psi_0 = N\hat{A}[\Psi^A\Psi^B]$, which properly obeys the Pauli principle through explicit antisymmetrization ($\hat{A}$ operator) and renormalization (N = constant) of the product wave function. It comprises the destabilizing interactions between electrons of the same spin on either fragment. The orbital interaction ΔE_{orb} accounts for charge transfer and polarization effects.[42] In the case that the Grimme dispersion corrections[29,30] are computed the term ΔE_{disp} is added to equation S1. Further details on the EDA method can be found in the literature.[43,44] In the case of the dimers, relaxation of the fragments to their equilibrium geometries at the electronic ground state is termed ΔE_{prep}, because it may be

considered as preparation energy for chemical bonding. The addition of ΔE_{prep} to the intrinsic interaction energy ΔE_{int} gives the total energy ΔE, which is, by definition, the opposite sign of the bond dissociation energy D_e:

$$\Delta E(-D_e) = \Delta E_{\mathrm{int}} + \Delta E_{prep} \tag{S2}$$

The EDA–NOCV method combines the EDA with the natural orbitals for chemical valence (NOCV) to decompose the orbital interaction term ΔE_{orb} into pairwise contributions. The NOCVs Ψ_i are defined as the eigenvector of the valence operator, $\hat{V}$, given by Equation (S3).

$$\hat{V}\Psi_i = v_i \Psi_i \tag{S3}$$

In the EDA–NOCV scheme the orbital interaction term, ΔE_{orb}, is given by Equation (S4),

$$\Delta \mathrm{E}_{\mathrm{orb}} = \sum_{\mathrm{k}} \Delta \mathrm{E}_{\mathrm{k}} = \sum_{\mathrm{k}=1}^{\mathrm{N}/2} v_k \left[-\mathrm{F}_{\mathrm{-k,k}}^{\mathrm{TS}} + \mathrm{F}_{\mathrm{k,k}}^{\mathrm{TS}} \right] \tag{S4}$$

in which $\mathrm{F}_{-\mathrm{k},-\mathrm{k}}^{\mathrm{TS}}$ and $\mathrm{F}_{\mathrm{k,k}}^{\mathrm{TS}}$ are diagonal transition state Kohn–Sham matrix elements corresponding to NOCVs with the eigenvalues $-v_k$ and v_k, respectively. The ΔE_k^{orb} term for a particular type of bond is assigned by visual inspection of the shape of the deformation density $\Delta\rho_k$. The latter term is a measure of the size of the charge deformation and it provides a visual notion of the charge flow that is associated with the pairwise orbital interaction. The EDA–NOCV scheme thus provides both qualitative and quantitative information about the strength of orbital interactions in chemical bonds. The EDA-NOCV calculations were carried out with ADF2019.101. The basis sets for all elements have triple-ζ quality augmented by two sets of polarizations functions and one set of diffuse function. Core electrons were treated by the frozen-core approximation. This level of theory is denoted PBE0-D3(BJ)/TZ2P.[45] Scalar relativistic effects have been incorporated by applying the zeroth-order regular approximation (ZORA).[46]

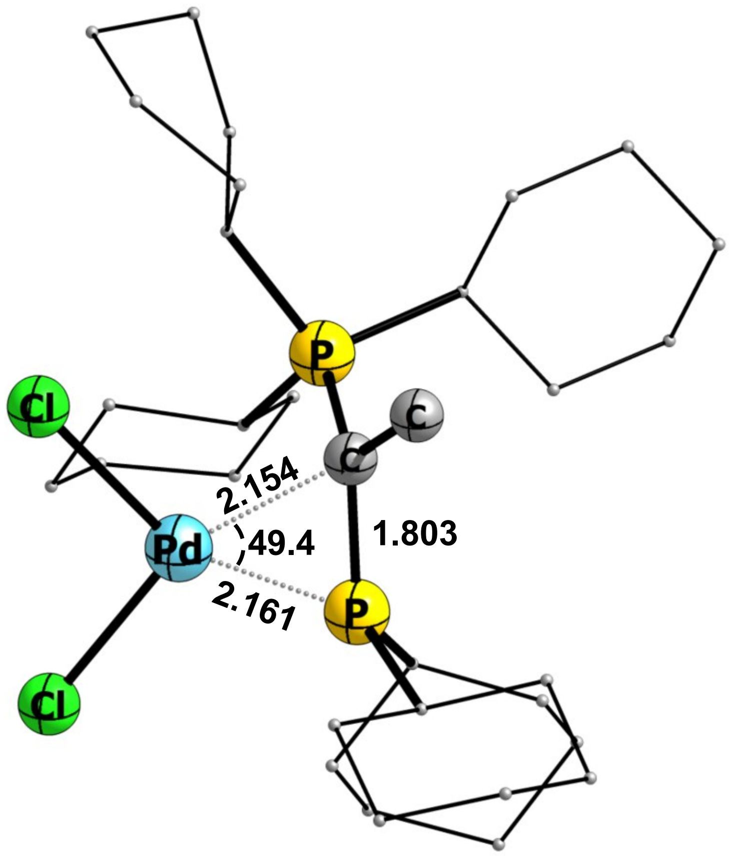

Figure S50. Optimized geometry of **L1PdCl₂** at the PBE0-D3(BJ)/def2-SVP level of theory. Selected computational bond length in [Å] and bond angles [°]. Hydrogen atoms were omitted for clarity.

	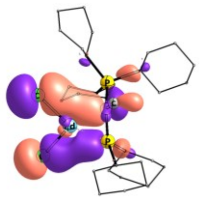	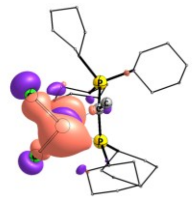
	**HOMO-5** (ε = -6.80 eV)	**HOMO-4** (ε = -6.39 eV)
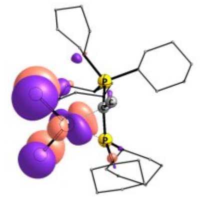	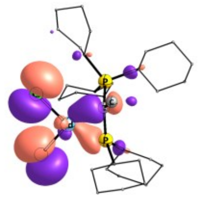	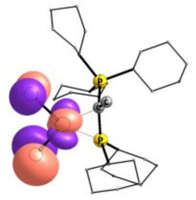
**HOMO-3** (ε = -6.28 eV)	**HOMO-2** (ε = -5.94 eV)	**HOMO-1** (ε = -5.78 eV)
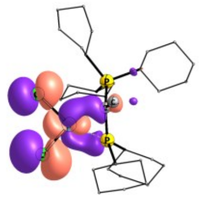	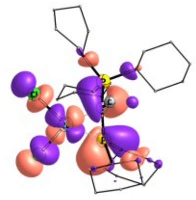	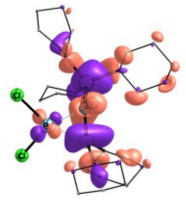
**HOMO** (ε = -5.45 eV)	**LUMO** (ε = -1.06 eV)	**LUMO+1** (ε =-0.11 eV)

Figure S51. Shape of frontier molecular orbitals (isocontour = 0.03 au) of **L1PdCl$_2$** and eigenvalues (in eV) at the PBE0-D3(BJ)/def2-TZVPP//PBE0-D3(BJ)/def2-SVP level of theory. Hydrogen atoms were omitted for clarity.

Table S25. NBO results calculated at PBE0-D3(BJ)/def2-TZVPP//PBE0-D3(BJ)/def2-SVP level of theory: partial charges, Q (in e), Wiberg bond order, P (in a.u.).

	**L1PdCl$_2$**
**Q(Pd)**	+0.40
**Q(Cl)**	-0.67
**Q(Cl)**	-0.63
**Q(C1)**	-0.95
**Q(P1)**	+1.74
**Q(P2)**	+1.22
**Q(PdCl$_2$)**	-0.90

**Q(L1)**	+0.90
**P(Pd−C1)**	0.34
**P(Pd-P2)**	0.54
**P(C1-P2)**	1.07

Table S26. NBO results at the PBE0-D3(BJ)/def2-TZVPP//PBE0-D3(BJ)/def2-SVP of compound **L1PdCl$_2$** using default resonance structure (Structures 2). Hydrogen atoms were omitted for clarity.

Orbital	Occ.	Contribution from atoms to the orb	Atomic orbitals
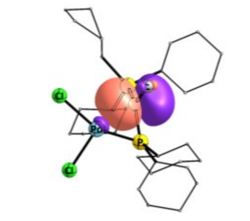	1.40	LP(C)	C: *s*(9.71%) *p* (90.07%) *d* (0.19%)
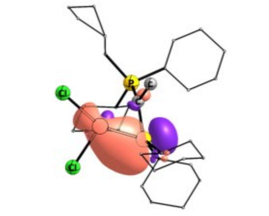	1.85	Pd (38.24%) - P (61.76%)	Pd: *s*(37.98%) *p* (0.39%) *d* (61.54%)<br>P: *s*(23.30%) *p* (76.31%) *d* (0.37%)
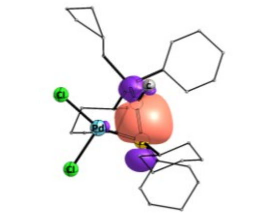	1.95	P (37.02%) - C (62.98%)	P: *s*(24.39%) *p*(75.05%) *d*(0.54%)<br>C: *s*(27.27%) *p*(72.48%) *d*(0.22%)
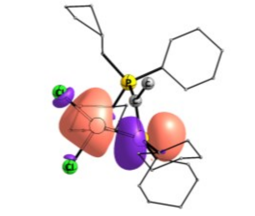	0.51	Pd (61.76%) - P (38.24%)	Pd: *s*(37.98%) *p*(0.39%) *d*(61.54%)<br>P: *s*(23.30%) *p* (76.31%) *d* (0.37%)
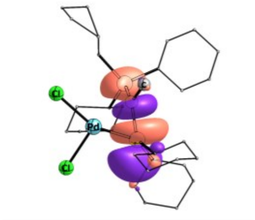	0.06	P (62.98%) - C (37.02%)	P: s(24.39%) p(75.05%) d(0.54%)<br>C: *s*(27.27%) *p*(72.48%) *d*(0.22%)

Table S27. NBO results at the PBE0-D3(BJ)/def2-TZVPP//PBE0-D3(BJ)/def2-SVP of compound **L1PdCl$_2$** using a chosen resonance structure (Structures 2). Hydrogen atoms were omitted for clarity.

Orbital	Occ.	Contribution from atoms to the orb	Atomic orbitals
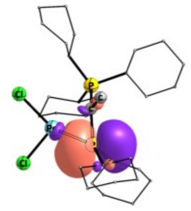	1.29	LP(P)	P: s (11.25%) p (88.48%) d (0.26%)
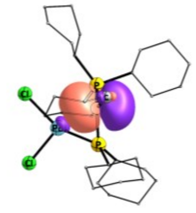	1.40	LP(C)	C: s (9.71%) p (90.07%) d (0.19%)
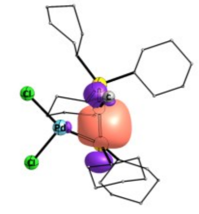	1.96	P (37.08%) - C (62.92%)	P: *s*(26.55%) *p* (72.91%) *d*(0.52%)<br>C: *s*(27.27%) *p* (72.48%) *d*(0.22%)
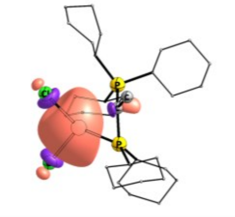	0.54	LV(Pd)	s(75.65%) p(0.42%) d(23.81%)
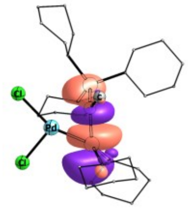	0.06	P (62.92%) - C (37.08%)	P: *s*(26.55%) *p* (72.91%) *d* (0.52%)<br>C: *s*(27.27%) *p* (72.48%) *d* (0.22%)

Table S28. NBO results for two selected Lewis structures at the PBE0-D3(BJ)/def2-TZVPP//PBE0-D3(BJ)/def2-SVP level of theory.

	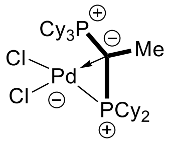	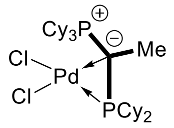
Effective core	28.00000	28.00000
Core	111.99917 (99.999% of 112)	111.99917 (99.999% of 112)
Valence Lewis	216.46839 (98.395% of 220)	215.89662 (98.135% of 220)
Total Lewis	356.46757 (99.019% of 360)	355.89580 (98.860% of 360)
Valence non-Lewis	3.04503 (0.846% of 360)	3.61768 (1.005% of 360)
Rydberg non-Lewis	0.48740 (0.135% of 360)	0.48652 (0.135% of 360)
Total non-Lewis	3.53243 (0.981% of 360)	4.10420 (1.140% of 360)

Conclusions from the NBO analysis

Natural population analysis (NPA) indicates an electron-rich C1 atom with -0.95 e and a positively charged P2 atom with +1.22 e. The complete moiety of $PdCl_2$ bears a negative charge of -0.90e, mainly located at the chlorine atoms, which are donated by the YPhos fragment. The Wiberg bond order values are consistent with a weak single bond character for P-Pd (P = 0.54 au) and C-Pd (P = 0.34 au) bonds. This suggests a dative bond for both the phosphine and the ylide donor. The NBO analysis leads to two plausible Lewis structures (Table S28). We have chosen the Lewis representation with a two lone pair at the YPhos ligand located at the P atom and C atom 89% and 90% *p*-character, respectively, since phosphines are in general not regarded as Z-type ligands. Yet, this Lewis structure expresses the strong donor properties of the ylide-substituted phosphines and clearly show that the ylide acts as a L-type ligand. Importantly, the second order perturbation interaction (ΔE^2) within the NBO method suggests a stabilization LP(P)→LV(Pd) is 55.9 kcal/mol and the LP(C) → LV(Pd) is 66.8 kcal/mol.

Table S29. EDA-NOCV at the PBE0-D3(BJ)/TZ2P level of theory.[a]

	L1(S); $PdCl_2$ (S)	$L1^{+1}$(D); $PdCl_2^{-1}$ (D)
ΔE_{int}	-142.1	-154.5
ΔE_{Pauli}	282.9	304.4
ΔE_{disp}[b]	-13.6 (3.2 %)	-13.6 (3.0 %)
ΔE_{elstat}[b]	-239.0 (56.2 %)	-296.7 (64.7 %)
$\Delta E_{orb\ total}$[b]	-172.3 (40.5 %)	-148.6 (32.4 %)
$\Delta E_{orb\ HF}$	0.3	0.0
ΔE_{orb}	-172.6	-148.6
$\Delta E_{orb\text{-}\sigma}$[c]	-103.1 (59.7 %)	-83.9 (56.5 %)
$\Delta E_{orb\text{-}\pi}$[c]	-15.2 (8.8 %)	-18.5 (12.5 %)
$\Delta E_{orb\text{-}\pi}$[c]	-13.9 (8.1 %)	-14.4 (9.7 %)
$\Delta E_{orb\text{-}pol}$[c]	-11.7 (6.8 %)	-7.7 (5.2 %)
$\Delta E_{orb\text{-}rest}$[c]	-40.4 (23.4 %)	-31.8 (21.4 %)
$\Delta E_{prep\ PdCl2}$	10.1	
$\Delta E_{prep\ L1}$	29.5	
$\Delta E_{prep\ total}$	39.9	52.3
D_e	102.2	102.2
Electron Flow	1.28e	1.33e

[a] Geometries optimized at the PBE0-D3(BJ)/def2-TZVP level of theory. [b] The value in parenthesis gives the percentage contribution to the total attractive interactions ΔE_{elstat} + ΔE_{orb} + ΔE_{disp}. [c] The values in parenthesis gives the percentage contribution to the total orbital interaction.

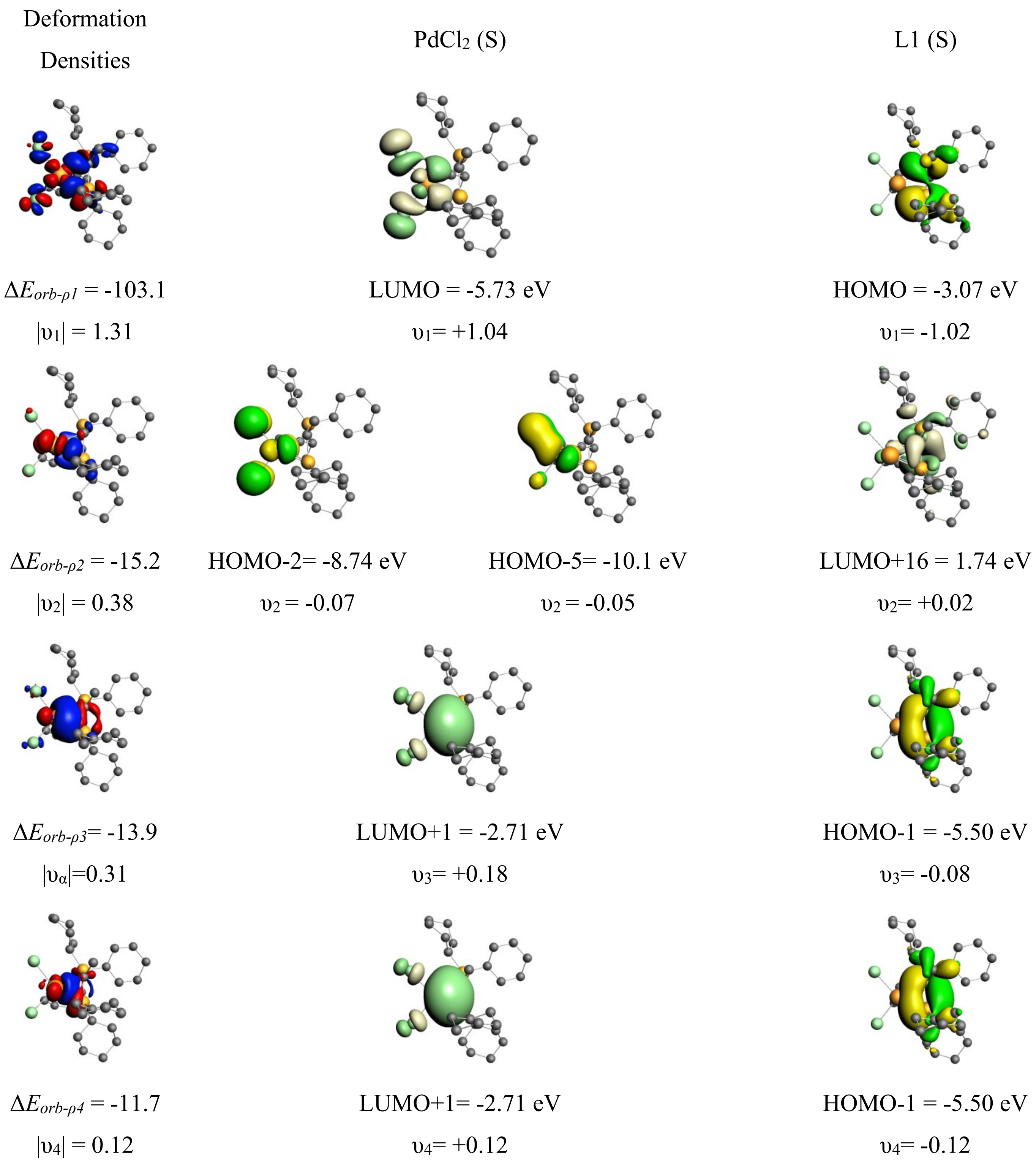

Figure S52. Plot of deformation densities Δρ (isovalue = 0.003) of the pairwise orbital interactions between L1(S) and PdCl$_2$(S), associated energies ΔE (in kcal/mol) and eigenvalues v (in a.u.). The red colour shows the charge outflow, whereas blue shows charge density accumulation. Shape of the most important interacting occupied and vacant orbitals (isovalue = 0.03) of the fragments. The fragment molecular orbitals the occupied orbitals are shown in blue and yellow, while virtual orbitals are in cyan and pale yellow. Hydrogen atoms are omitted for clarity.

Deformation Densities	$PdCl_2$ (D)		L1 (D)	
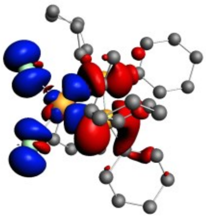	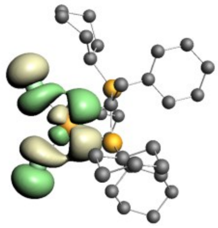		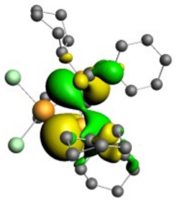	
$\Delta E_{orb-\rho 1}$ = -83.9<br>$\|\upsilon_{1\alpha}\|$=0.62<br>$\|\upsilon_{1\beta}\|$=0.62	SOMO = -0.08 eV<br>$\upsilon_{1\alpha}$=+0.51<br>$\upsilon_{1\beta}$=-0.48		SOMO = -6.84 eV<br>$\upsilon_{1\alpha}$=-0.52<br>$\upsilon_{1\beta}$=+0.50	
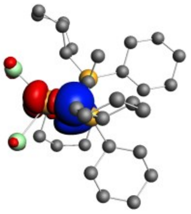	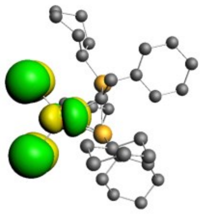		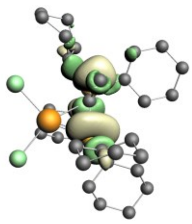	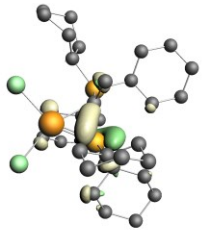
$\Delta E_{orb-\rho 2}$ = -18.5<br>$\|\upsilon_{2\alpha}\|$=0.18<br>$\|\upsilon_{2\beta}\|$=0.18	HOMO-3= -2.27 eV<br>$\upsilon_{2\alpha}$=-0.05<br>$\upsilon_{2\beta}$=-0.05		LUMO = -2.74 eV<br>$\upsilon_{2\alpha}$=+0.01<br>$\upsilon_{2\beta}$=+0.01	LUMO+2 = -2.36 eV<br>$\upsilon_{2\alpha}$=+0.01<br>$\upsilon_{2\beta}$=+0.01
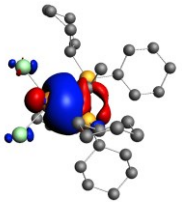	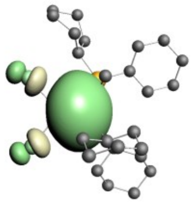	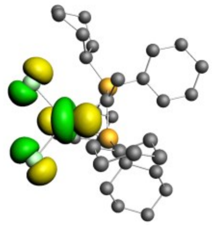	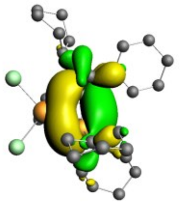	
$\Delta E_{orb-\rho 3}$= -14.4<br>$\|\upsilon_{3\alpha}\|$=0.16<br>$\|\upsilon_{3\beta}\|$=0.16	LUMO+1 = 2.44 eV<br>$\upsilon_{3\alpha}$=-0.04<br>$\upsilon_{3\beta}$=-0.04	LUMO+1 = -2.25 eV<br>$\upsilon_{3\alpha}$=+0.07<br>$\upsilon_{3\beta}$=+0.07	HOMO-1 = -9.84 eV<br>$\upsilon_{3\alpha}$=-0.03<br>$\upsilon_{3\beta}$=-0.03	
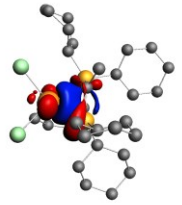	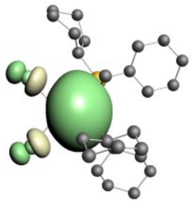	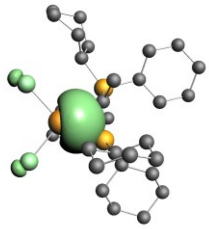	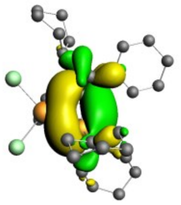	
$\Delta E_{orb-\rho 4}$ = -7.7	LUMO+1 = 2.44 eV	LUMO+1 = 5.11 eV	HOMO-1 = -9.84 eV	

$\lvert\upsilon_{4\alpha}\rvert$=0.10	$\upsilon_{4\alpha}$=-0.04	$\upsilon_{4\alpha}$=+0.02	$\upsilon_{4\alpha}$=-0.04
$\lvert\upsilon_{4\beta}\rvert$=0.10	$\upsilon_{4\beta}$=+0.04	$\upsilon_{4\beta}$=+0.02	$\upsilon_{4\beta}$=-0.04

Figure S53. Plot of deformation densities Δρ (isovalue = 0.003) of the pairwise orbital interactions between $L1^{+1}$(D) and $PdCl_2^{-1}$(D), associated energies ΔE (in kcal/mol) and eigenvalues v (in a.u.). The red colour shows the charge outflow, whereas blue shows charge density accumulation. Shape of the most important interacting occupied and vacant orbitals (isovalue = 0.03) of the fragments. The fragment molecular orbitals the occupied orbitals are shown in blue and yellow, while virtual orbitals are in cyan and pale yellow. Hydrogen atoms are omitted for clarity.

Table S30. QTAIM Local topological parameters [in a.u.] of the charge density at the main Bond Critical Points (BCP) calculated at PBE0-D3(BJ)/def2-TZVPP//PBE0-D3(BJ)/def2-SVP level of theory: Electron density (ρ(r)), Laplacian of the electron density ($\nabla^2\rho(r)$), local kinetic energy density (G), Local potential energy density (V), and local electronic energy density (H).

BCP	ρ(r)	$\nabla^2\rho(r)$	V	G	H
Pd-C	0.099 (0.67 e/Å^3)	0.230 (5.55 e/Å^5)	-0.1207	0.0891	-0.0316
Pd-P	0.125 (0.84 e/Å^3)	0.071 (1.72 e/Å^5)	-0.1542	0.0860	-0.0682
P-C	0.161	-0.081	-0.2767	0.1283	-0.1484

5.2 xyz coordinations (in Å) and Energies (in Hartree)

L1·$PdCl_2$
Energy(PBE0-D3(BJ)/def2-SVP) = -2982.166410164

```
Pd   0.399315000000   -0.112021000000    0.055048000000
Cl   1.641596000000    0.105384000000   -1.970292000000
Cl  -1.674924000000   -0.666812000000   -0.873446000000
P    2.842578000000   -1.355052000000    1.450007000000
P    0.108632000000   -0.011713000000    2.194059000000
C    1.825304000000    0.105660000000    1.654036000000
C    2.565578000000    1.408482000000    1.912673000000
H    3.457670000000    1.503055000000    1.285089000000
H    2.884793000000    1.514612000000    2.963401000000
H    1.927953000000    2.267562000000    1.666851000000
C    3.842046000000   -1.651530000000    2.985207000000
H    4.177200000000   -2.701822000000    2.925937000000
C    5.089979000000   -0.764522000000    3.079977000000
H    5.733499000000   -0.904178000000    2.199972000000
H    4.791867000000    0.296041000000    3.081470000000
C    5.877670000000   -1.068449000000    4.351168000000
H    6.757961000000   -0.408659000000    4.407837000000
H    6.266892000000   -2.101167000000    4.295954000000
C    5.015736000000   -0.920107000000    5.598717000000
H    4.716383000000    0.137843000000    5.709222000000
```

H	5.595390000000	-1.174104000000	6.500245000000
C	3.766300000000	-1.786717000000	5.507550000000
H	4.057019000000	-2.852584000000	5.516914000000
H	3.121413000000	-1.633837000000	6.387886000000
C	2.975933000000	-1.495164000000	4.236688000000
H	2.585922000000	-0.463470000000	4.271730000000
H	2.094887000000	-2.152185000000	4.185309000000
C	3.986580000000	-1.273356000000	0.011134000000
H	3.273513000000	-1.433162000000	-0.816721000000
C	5.002762000000	-2.440358000000	0.066184000000
H	5.274837000000	-2.712528000000	1.097335000000
H	4.535690000000	-3.339104000000	-0.366039000000
C	5.292652000000	-0.029095000000	-1.729265000000
H	4.517830000000	0.183719000000	-2.480526000000
H	6.045828000000	0.770694000000	-1.810830000000
C	4.636021000000	0.065209000000	-0.344532000000
H	5.365336000000	0.377185000000	0.419860000000
H	3.849324000000	0.826043000000	-0.414047000000
C	1.781390000000	-2.852454000000	1.136954000000
H	0.780046000000	-2.509997000000	1.435609000000
C	1.635526000000	-3.222438000000	-0.347061000000
H	1.406179000000	-2.334992000000	-0.956993000000
H	2.582903000000	-3.645165000000	-0.720696000000
C	0.526291000000	-4.253919000000	-0.507905000000
H	0.424457000000	-4.511975000000	-1.573136000000
H	-0.431745000000	-3.786438000000	-0.219512000000
C	0.796822000000	-5.500918000000	0.323920000000
H	-0.037617000000	-6.215342000000	0.239138000000
H	1.688457000000	-6.016957000000	-0.076664000000
C	1.040890000000	-5.155413000000	1.787697000000
H	1.308343000000	-6.056657000000	2.362595000000
H	0.103991000000	-4.771705000000	2.230642000000
C	2.127279000000	-4.094897000000	1.962355000000
H	3.100366000000	-4.499510000000	1.631941000000
H	2.231481000000	-3.858797000000	3.032023000000
C	-0.514522000000	-1.380573000000	3.276853000000
H	0.192370000000	-2.213018000000	3.137731000000
C	-1.878921000000	-1.825368000000	2.731551000000
H	-1.816826000000	-2.013105000000	1.647203000000
H	-2.597546000000	-0.992322000000	2.837595000000
C	-2.395086000000	-3.040245000000	3.493549000000
H	-3.391043000000	-3.320033000000	3.116240000000
H	-1.734068000000	-3.901405000000	3.285431000000
C	-2.439838000000	-2.791515000000	4.996939000000
H	-3.197289000000	-2.016515000000	5.213068000000
H	-2.765988000000	-3.699109000000	5.529666000000
C	-1.088500000000	-2.324338000000	5.526468000000
H	-0.348938000000	-3.138874000000	5.416107000000
H	-1.149219000000	-2.106007000000	6.604884000000
C	-0.586361000000	-1.091981000000	4.777016000000
H	-1.280669000000	-0.254674000000	4.961200000000
H	0.394934000000	-0.782043000000	5.167175000000
C	-0.633629000000	1.538841000000	2.868366000000
H	-1.601528000000	1.148208000000	3.239625000000
C	0.073285000000	2.216626000000	4.046242000000
H	0.288772000000	1.495115000000	4.848150000000

H	1.043693000000	2.612696000000	3.711553000000
C	-0.772855000000	3.369987000000	4.580042000000
H	-1.710986000000	2.966532000000	5.003058000000
H	-0.245643000000	3.865421000000	5.411401000000
C	-1.104020000000	4.373407000000	3.480577000000
H	-0.169105000000	4.851663000000	3.136057000000
H	-1.736241000000	5.182638000000	3.879909000000
C	-1.787169000000	3.697265000000	2.296880000000
H	-1.979060000000	4.424514000000	1.492430000000
H	-2.776387000000	3.319976000000	2.613803000000
C	-0.965120000000	2.534668000000	1.751129000000
H	-1.496560000000	2.015751000000	0.938571000000
H	-0.031881000000	2.916526000000	1.302206000000
C	6.275938000000	-2.115300000000	-0.716192000000
H	6.941858000000	-1.479546000000	-0.106531000000
H	6.832412000000	-3.046301000000	-0.907356000000
C	5.933815000000	-1.395862000000	-2.011507000000
H	5.235329000000	-2.026378000000	-2.588105000000
H	6.828532000000	-1.280380000000	-2.642144000000

6. References

1 L. T. Scharf, I. Rodstein, M. Schmidt, T. Scherpf and V. H. Gessner, *ACS Catal.*, 2020, **10**, 999–1009.
2 P. Weber, T. Scherpf, I. Rodstein, D. Lichte, L. T. Scharf, L. J. Gooßen and V. H. Gessner, *Angew. Chem. Int. Ed.*, 2019, **58**, 3203–3207.
3 X.-Q. Hu, D. Lichte, I. Rodstein, P. Weber, A.-K. Seitz, T. Scherpf, V. H. Gessner and L. J. Gooßen, *Org. Lett.*, 2019, **21**, 7558–7562.
4 J. Tappen, I. Rodstein, K. McGuire, A. Großjohann, J. Löffler, T. Scherpf and V. H. Gessner, *Chem. – Eur. J.*, 2020, **26**, 4281–4288.
5 I. Rodstein, D. S. Prendes, L. Wickert, M. Paaßen and V. H. Gessner, *J. Org. Chem.*, 2020, **85**, 14674–14683.
6 T. Nakayama, H. Hikawa, S. Kikkawa and I. Azumaya, *RSC Adv.*, 2021, **11**, 23144–23150.
7 J. P. Tassone, E. V. England, P. M. MacQueen, M. J. Ferguson and M. Stradiotto, *Angew. Chem. Int. Ed.*, 2019, **58**, 2485–2489.
8 H. L. Li, Y. Kuninobu and M. Kanai, *Angew. Chem.*, 2017, **129**, 1517–1521.
9 K. Kamikawa, S. Sugimoto and M. Uemura, *J. Org. Chem.*, 1998, **63**, 8407–8410.
10 K. Matsubara, K. Ueno, Y. Koga and K. Hara, *J. Org. Chem.*, 2007, **72**, 5069–5076.
11 G. A. Chesnokov, P. S. Gribanov, M. A. Topchiy, L. I. Minaeva, A. F. Asachenko, M. S. Nechaev, E. V. Bermesheva and M. V. Bermeshev, *Mendeleev Commun.*, 2017, **27**, 618–620.
12 G. Adjabeng, T. Brenstrum, C. S. Frampton, A. J. Robertson, J. Hillhouse, J. McNulty and A. Capretta, *J. Org. Chem.*, 2004, **69**, 5082–5086.
13 N. Marion, E. C. Ecarnot, O. Navarro, D. Amoroso, A. Bell and S. P. Nolan, *J. Org. Chem.*, 2006, **71**, 3816–3821.
14 G. A. Grasa and T. J. Colacot, *Org. Lett.*, 2007, **9**, 5489–5492.
15 C. H. Cheon, T. Imahori and H. Yamamoto, *Chem. Commun.*, 2010, **46**, 6980.
16 T. Scherpf, H. Steinert, A. Großjohann, K. Dilchert, J. Tappen, I. Rodstein and V. H. Gessner, *Angew. Chem. Int. Ed.*, 2020, **59**, 20596–20603.
17 H. Lebel and C. Ladjel, *J. Org. Chem.*, 2005, **70**, 10159–10161.
18 N. Yoshikai, H. Matsuda and E. Nakamura, *J. Am. Chem. Soc.*, 2009, **131**, 9590–9599.
19 B. Qian, S. Guo, J. Shao, Q. Zhu, L. Yang, C. Xia and H. Huang, *J. Am. Chem. Soc.*, 2010, **132**, 3650–3651.
20 Y. Gan and T. A. Spencer, *J. Org. Chem.*, 2006, **71**, 5870–5875.
21 G. M. Sheldrick, *Acta Crystallogr. A*, 2008, **64**, 112–122.
22 A. Thorn, B. Dittrich and G. M. Sheldrick, *Acta Crystallogr. A*, 2012, **68**, 448–451.
23 G. M. Sheldrick, *Acta Crystallogr. Sect. C Struct. Chem.*, 2015, **71**, 3–8.
24 A. L. Spek, *Acta Crystallogr. Sect. C Struct. Chem.*, 2015, **71**, 9–18.
25 M. J. Frisch; G. W. Trucks; H. B. Schlegel; G. E. Scuseria; M. A. Robb; J. R. Cheeseman; G. Scalmani; V. Barone; B. Mennucci; G. A. Petersson; H. Nakatsuji; M. Caricato; X. Li; H. P. Hratchian; A. F. Izmaylov; J. Bloino; G. Zheng; J. L. Sonnenberg; M. Hada; M. Ehara; K. Toyota; R. Fukuda; J. Hasegawa; M. Ishida; T. Nakajima; Y. Honda; O. Kitao; H. Nakai; T. Vreven; J. A. Montgomery, J.; J. E. Peralta; F. Ogliaro; M. Bearpark; J. J. Heyd; E. Brothers; K. N. Kudin; V. N. Staroverov; R. Kobayashi; J. Normand; K. Raghavachari; A. Rendell; J. C. Burant; S. S. Iyengar; J. Tomasi; M. Cossi; N. Rega; J. M. Millam; M. Klene; J. E. Knox; J. B. Cross; V. Bakken; C. Adamo; J. Jaramillo; R. Gomperts; R. E. Stratmann; O. Yazyev; A. J. Austin; R. Cammi; C. Pomelli; J. W. Ochterski; R. L. Martin; K. Morokuma; V. G. Zakrzewski; G. A. Voth; P. Salvador; J. J. Dannenberg; S. Dapprich; A. D. Daniels; O. Farkas; J. B. Foresman; J. V. Ortiz; J. Cioslowski; D. J. Fox Gaussian 09, Revision C.01. Gaussian, Inc.: Wallingford CT, 2009.
26 *TURBOMOLE V7.0 2015* a development of University of Karlsruhe and Forschungszentrum Karlsruhe GmbH, 1989-2007; TURBOMOLE GmbH, since 2007; available from http://www.turbomole.com: Karlsruhe.

27 C. Adamo and V. Barone, *J. Chem. Phys.*, 1999, **110**, 6158–6170.
28 M. Ernzerhof and G. E. Scuseria, *J. Chem. Phys.*, 1999, **110**, 5029–5036.
29 S. Grimme, J. Antony, S. Ehrlich and H. Krieg, *J. Chem. Phys.*, 2010, **132**, 154104.
30 S. Grimme, S. Ehrlich and L. Goerigk, *J. Comput. Chem.*, 2011, **32**, 1456–1465.
31 F. Weigend and R. Ahlrichs, *Phys. Chem. Chem. Phys.*, 2005, **7**, 3297–3305.
32 C. Peng, P. Y. Ayala, H. B. Schlegel and M. J. Frisch, *J. Comput. Chem.*, 1996, **17**, 49–56.
33 J. W. McIver and A. Komornicki, *J. Am. Chem. Soc.*, 1972, **94**, 2625–2633.
34 A. E. Reed, R. B. Weinstock and F. Weinhold, *J. Chem. Phys.*, 1985, **83**, 735–746.
35 A. E. Reed, L. A. Curtiss and F. Weinhold, *Chem. Rev.*, 1988, **88**, 899–926.
36 E. D. Glendening, C. R. Landis and F. Weinhold, *J. Comput. Chem.*, 2019, 2234–2241.
37 R. F. Bader, *Atoms in molecules: a quantum theory*, Clarendon Pr, Oxford, 1990.
38 Keith, T. A.; Gristmill, T. K. *AIMAll*, 19.02.13; Overland Park KS, USA (aim.tkgristmill.com), 2019.
39 K. Morokuma, *J. Chem. Phys.*, 1971, **55**, 1236–1244.
40 T. Ziegler and A. Rauk, *Inorg. Chem.*, 1979, **18**, 1558–1565.
41 T. Ziegler and A. Rauk, *Inorg. Chem.*, 1979, **18**, 1755–1759.
42 F. M. Bickelhaupt, N. M. M. Nibbering, E. M. Van Wezenbeek and E. J. Baerends, *J. Phys. Chem.*, 1992, **96**, 4864–4873.
43 F. M. Bickelhaupt and E. J. Baerends, in *Reviews in Computational Chemistry*, eds. K. B. Lipkowitz and D. B. Boyd, John Wiley & Sons, Inc., Hoboken, NJ, USA, 2007, pp. 1–86.
44 G. te Velde, F. M. Bickelhaupt, E. J. Baerends, C. Fonseca Guerra, S. J. A. van Gisbergen, J. G. Snijders and T. Ziegler, *J. Comput. Chem.*, 2001, **22**, 931–967.
45 J. Krijn and E. J. Baerends, *Fit Functions in the HFS-Method*, 1984.
46 E. van Lenthe, E. J. Baerends and J. G. Snijders, *J. Chem. Phys.*, 1993, **99**, 4597–4610.
